# Applying an Anti-Kasha Model Resolves Differences Between Photosynthetic and Artificial Pigments

**DOI:** 10.1021/acs.jpcb.5c02465

**Published:** 2025-07-23

**Authors:** Jan P. Götze, Simon Petry, Sebastian Reiter, Heiko Lokstein, Regina de Vivie-Riedle

**Affiliations:** † 54203 Freie Universität Berlin, Fachbereich Biologie Chemie Pharmazie, Physikalische und Theoretische Chemie, Arnimallee 22, Berlin 14195, Germany; ‡ 9183 Ludwig-Maximilians-Universität München, Department Chemie, Butenandtstr. 5-13, Munich 81377, Germany; § Department of Chemical Physics and Optics, Charles University in Prague, Ke Karlovu 3, Prague 121 16, Czech Republic

## Abstract

The current interpretation of excitation energy transfer (EET) processes in natural photosynthesis generally relies on Kasha’s rule, suggesting that internal conversion (IC) processes usually outpace any EET between higher excited states. It is, however, known from research on artificial systems that Kasha’s rule does not apply to many dyes, especially when found in assembled clusters analogous to photosynthetic chlorophyll (Chl)-protein complexes. In this contribution, a semiempirical Förster-type model is applied to otherwise well-investigated pigments of natural photosynthesis (Chls *a*, *b*, *c1* and various carotenoids). Strong potential for anti-Kasha processes is identified in all investigated pigments, based on their high Coulomb coupling elements, similar to compounds with already known anti-Kasha properties. The pigments are further found to form strongly delocalized excitons, especially between the higher excited states usually responsible for anti-Kasha pathways. Test calculations with different pigment compositions for various natural light harvesting complexes (LHCII, CP24, CP26, CP29, FCP) demonstrate how the higher band EET network and absorbance could be affected by the presence of accessory pigments: Chl *a*-only networks should perform anti-Kasha EET, but this is suppressed by the presence of accessory pigments via several mechanisms (exciton disruption, spectral competition, energy sinks and fast, non-Chl *a* IC). The apparent “special” behavior of photosynthetic systems is thus resolved as the result of pigment mixtures.

## Graphical abstract

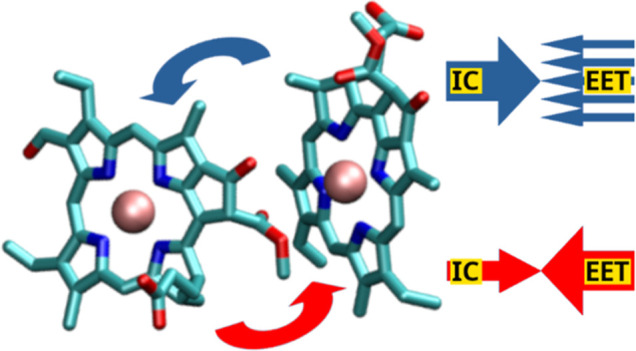

## 1 Introduction

### 1.1 Natural vs Artificial Photosynthesis

Recent decades have seen a surge of applications that mimic biological processes, among them the photosynthetic light reactions. This research promises an efficient conversion of solar into chemical energy, with the potential to be exclusively based on sustainable and cheap materials.^1,2^ Since natural photosynthesis also uses carbon dioxide as a direct input resource, artificial photosynthetic systems may help to solve two of the most pressing socio-environmental issues, namely clean energy production and climate change.

To expand the chemical scope of artificial systems, a wide variety of potentially highly effective organic compounds have been proposed and employed.^2,3^ They usually present large, conjugated π-systems, formed by hydrocarbons, often supplemented by nitrogen and oxygen. This mimics the key players in natural photosynthesis, chlorophylls (Chls) and carotenoids (Crts).^4^ Several groups have also gone so far as to use metalloporphyrins in their approaches, which is in structure and properties very close to the metallochlorin chromophore in Chls.^3^

Interestingly, the presented excited state energy pathways, such as excitation energy transfer (EET), internal conversion (IC) and emission (radiative decay), can sometimes differ between natural and artificial photosynthesis models. The characterization of artificial excited state energy pathways is in several cases impossible when strictly adhering to Kasha’s rule;^5^ a full understanding often requires inclusion of EET rates between higher energy states (S_n_–S_n_ EET).^6–8^ For natural photosynthesis systems, however, EET is interpreted almost always in a Kasha-like picture (only S_1_–S_1_ EET).^9,10^ A pictorial representation of the two perspectives, Kasha and anti-Kasha, is given in Figure 1. It should be noted that the original definition by Kasha refers only to emission from higher states, but has evolved nowadays to other processes as well, i.e., EET between higher excited states is also considered to be “anti-Kasha”.^8^ It refers to all processes that efficiently compete with IC, except emission from the lowest electronically excited state—and thus often involves two (or more) pigment molecules.

**Figure 1:**
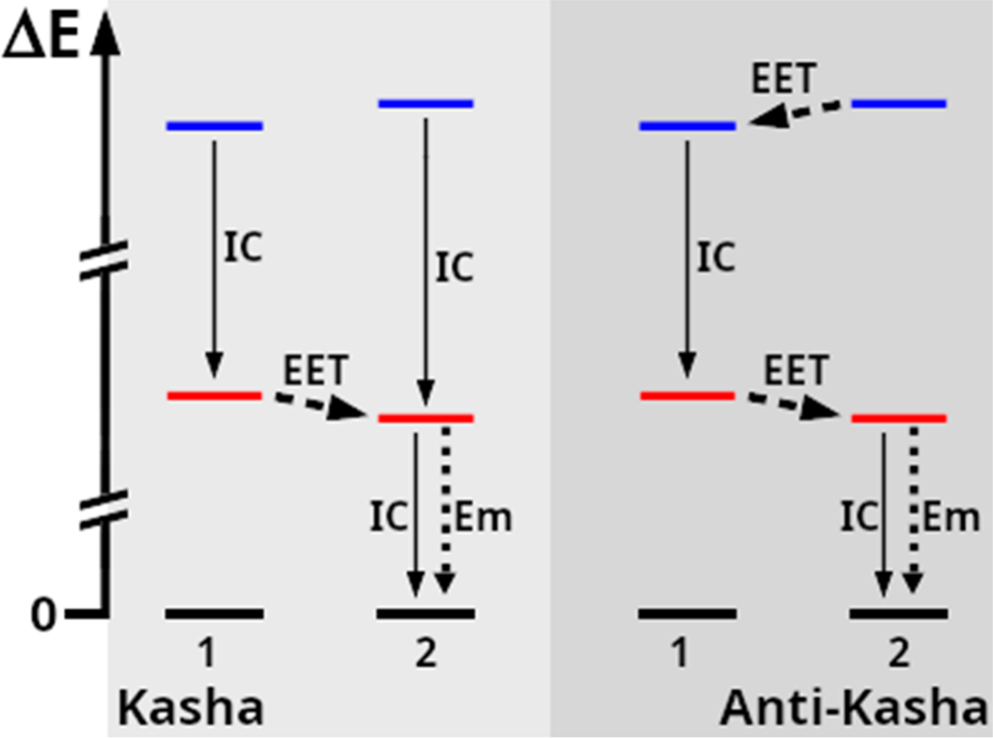
Comparison of excited state energy pathways between in an ideal Kasha-like situation (left) vs an ideal anti-Kasha arrangement (right). Shown are two pigments labeled “1” and “2” with different order of their excited states (or excitons, marked blue/red) compared to the ground state energy (black). The main Kasha/anti-Kasha difference is that blue-to-red internal conversion (IC, solid arrows) in the anti-Kasha case is most likely located at the pigment with the lowest “blue state” excitation energy (“1”), due to excitation energy transfer (EET, dashed arrows) between higher states. Radiative emission (Em, dotted arrows) at the terminal emitter (“2”) remains the same in both cases. Potential examples in the context of photosynthesis are Crts or Chl *b* (pigment 1) and Chl *a* (pigment 2), as suggested previously.^17,18,79^

A possible reason for the neglect of anti-Kasha processes in photosynthesis research might be that artificial models require systematic design, and research of natural assemblies relies on systematic decomposition. The decomposition approach, however, infers the properties of a complex system from its individual parts, and since IC between Chl excited states was found to be very fast,^11–13^ the Kasha picture seems to be appropriate. At the time of writing this paper, one of the most cited anti-Kasha reviews by Demchenko, Tomin and Chou (2017)^8^ had more than 300 citations, with only 8 of them explicitly mentioning photosynthesis, and solely 1 being concerned with natural pigments.^14^

To address the potential of anti-Kasha in Chl-based systems, we recently published a series of papers employing site-based Förster models.^15–18^ Despite our efforts, it remains unclear if anti-Kasha EET is actually relevant for photosynthetic pigments: Any related experimental evidence on their biologically assembled state is difficult to obtain, since the biological Chl-Crt assemblies are structurally very complex.^4^

To further incentivize experimental efforts, we aim in this contribution for a comparison between the natural photosynthetic pigments and experimentally well characterized anti-Kasha systems. This paper is thus to answer the following questions: (i) Do the natural photosynthetic pigments fall into the categories for which strong anti-Kasha effects are to be expected? (ii) If yes, can the roles of pigments, in the context of the anti-Kasha picture, be evaluated beyond the site-based Förster approach?

To answer the first question, solid criteria have been proposed in recent years; yet mostly focusing on anti-Kasha emission in individual pigments (i.e., not directly applicable to pigment assemblies as shown in Figure 1). A corresponding, quantum-chemical classification scheme was proposed by Veys and Escudero,^19^ stating three types of processes. Types I and II are based on strong electronic nonadiabatic coupling (NAC) with the ground state, while at the same time preventing processes between the S_1_ state and the ground state. Type III of their classification further directly introduces EET between higher energy states, for compounds with very weak NAC. The first assessment must thus be done on the basis of the individual pigment’s properties.

### 1.2 Properties of Photosynthetic Pigments

Chlorophylls and bacteriochlorophylls, (B)­Chls, are the pigments which perform the primary processes in natural photosynthesis: light absorption, EET and charge separation.^4,20^ Various other (B)­Chls have succeeded in finding their niches: Chl *b* acts as an accessory pigment in plant and green algal light-harvesting complexes (LHCs),^21,22^ similar to the family of Chl *c*-type pigments from various algae (incl. diatoms), dinoflagellates and other organisms.^23,24^ Furthermore, Chls *d* and *f* are far-red absorbing pigments, present under specific conditions in some cyanobacteria.^25,26^ (B)­Chls also provide a large range of chemical modifications, mostly leading to shifts of the lowest absorption band (called the Q band, between 550 and 850 nm, 1.5 to 2.3 eV). An overview of the spectra for several (B)­Chls is given in Figure 2.

**Figure 2:**
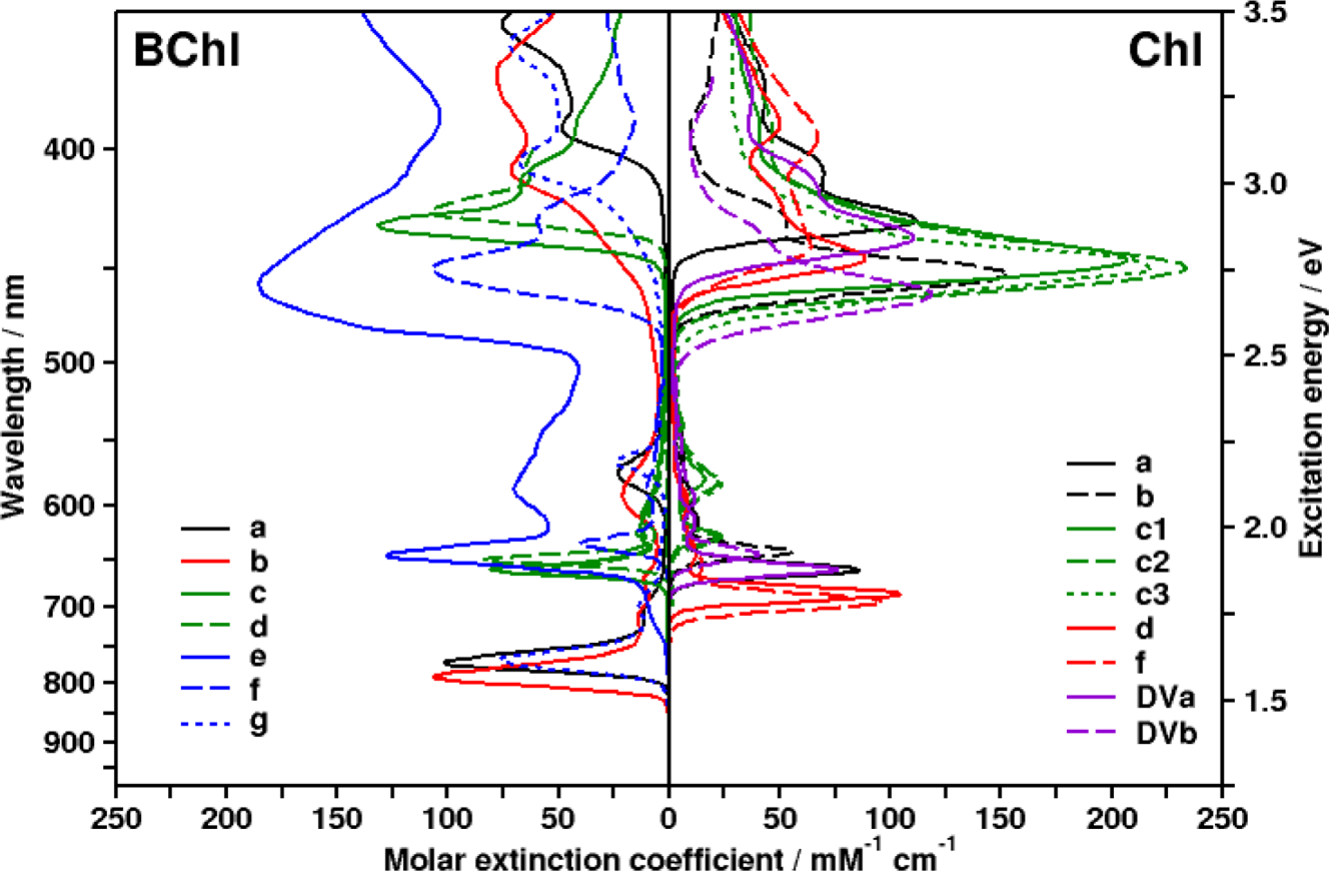
Overview of various BChl (left) and Chl (right) absorption spectra for the UV/visible range in diethyl ether (except for for BChl e, in acetone), collected from the PhotochemCAD database.^80^ References for spectra and extinction coefficients: BChl *a*,^81,82^ *b*,^83^ *c* and *d*,^84,85^ *e*,^86,87^ BChl *f* spectrum for BChl *f* [E, M],^88,89^ *g* (extinction in methanol).^90,91^ Chl *a* and *b*,^92–94^ *c*-types,^95–97^ *d* and *f*,^98^ divinyl (DV)­Chl *a* and *b*.^99,100^

**Figure 3:**
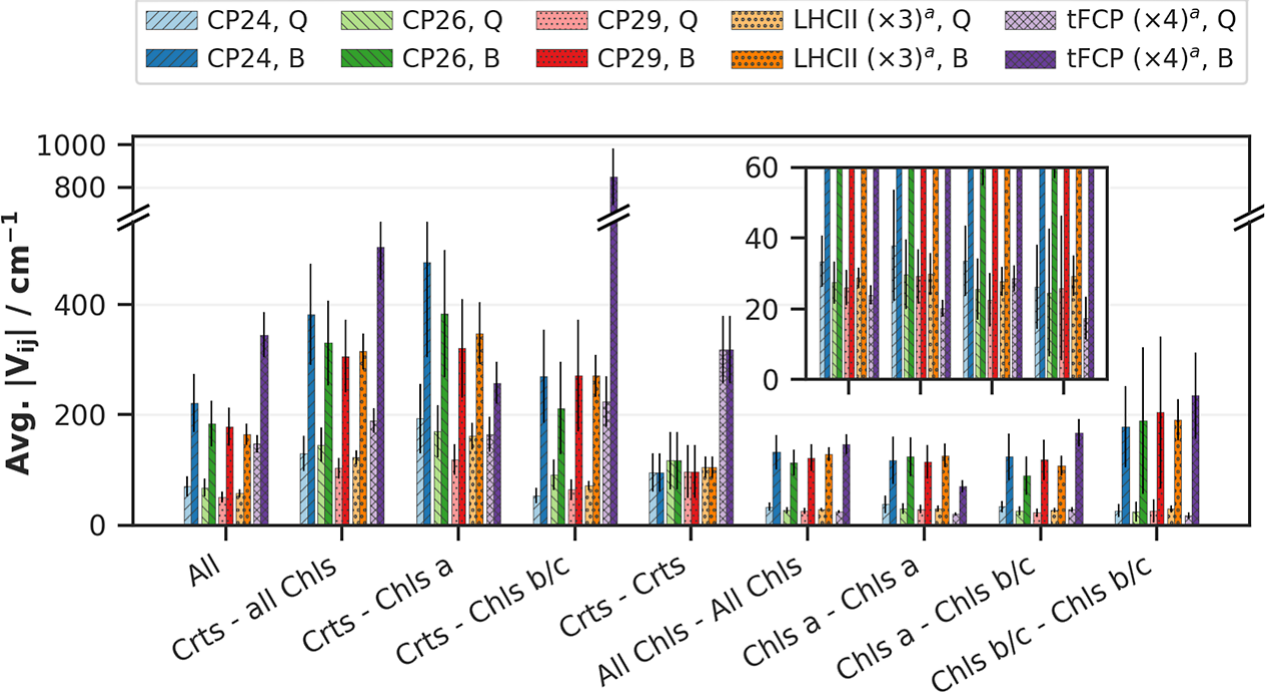
Average Förster coupling elements, summed over pairs of pigment classes, in the Q (light bars) and B (dark bars) bands of the investigated complexes. ^a^ Values for LHCII and tFCP were obtained by averaging the couplings over the whole multimer followed by multiplication with the multimeric order (3 and 4, respectively), to account for the high number of spatially distant, weakly coupled mirror images of each complex, which would otherwise artificially lower the average coupling compared to the monomeric LHC complexes. The inset shows a magnified view of the right half of the figure, starting with All Chls – All Chls, to magnify the comparatively weak Chl–Chl coupling in the Q band. Standard error of the mean indicated as gray bars.

Chl *a* and BChl *a* are the main pigments for natural photosynthetic productivity. They are responsible for charge separation in the photosynthetic reaction centers (RCs).^27,28^ The formation of an intermolecular charge transfer (CT) state seems to be extremely fine-tuned with the rest of the protein environment and the associated electron donors and acceptors,^29^ so that evolution rarely touches this basic concept (see also the highly conserved Chl *a* numbers in the PSII core complex indicated in Figure S1 of the Supporting Information, SI).^30^ Since the RC pigment binding sites are occupied by only a few (B)­Chl species, the other (B)­Chl pigments and the Crts are generally associated with different functions: Absorption of light and subsequent EET to the RCs.^9^

In the context of the proposed anti-Kasha pathways, (B)­Chls can be compared to other porphyrins which find application in artificial antenna models.^3^ The orbital structure between chlorins and porphyrins is not identical, but a recent quantum chemical study has demonstrated that the substituents found in Chl *a* revert these differences for the four frontier orbitals.^31^ As it turns out, the nondegeneracy of the Chl *a* frontier orbitals is also not affecting the quantum dynamics of the excited state too much: This leads to a strongly coupled pair of Q_*y*_ and Q_*x*_ states (which form the Q band), much stronger than could be assumed due to the difference in Q_*y*_ and Q_*x*_ absorption energies.^32^ The Gouterman porphyrin model thus applies surprisingly well to Chl *a*,^33^ via several effects that draws the asymmetric Chl *a* “back” to an ideal, porphyrin-like behavior. This may be the case for most (B)­Chls, as corroborated by the highly similar spectra shown for the various (B)­Chls in Figure 2.

For all natural (B)­Chls, the “B” (or “Soret”) band in the ultraviolet/blue (UV/blue) region is much more pronounced than the Q-band (see Figure 2, 500 nm and lower wavelengths, or higher than 2.5 eV). Computations of the B band present at least two, but generally three to five electronic B band states,^34,35^ with several theoretical approaches predicting at least one intramolecular charge transfer state close to the majorly absorbing states (B_*x*_ and B_*y*_).^36^ Theory therefore struggles with a clear assignment of the energetically lowest B band state, also since experiments are usually unable to excite these states individually due to their strongly overlapping peaks. The potential for dynamic state mixing, as mentioned above for the Q band, complicates matters further.^32^

Besides (B)­Chls, Crts are also abundantly present in natural photosynthetic pigment assemblies.^4^ Crts present a large, vibrationally strongly broadened absorption peak, corresponding to a single electronic transition (S_0_→S_2_). It is energetically located close to the B band absorption of the (B)­Chl compounds (see Figure S2 in the SI), starting at about 525 nm and higher wavelengths. In contrast to the (B)­Chls, Crts are already well-known to exhibit anti-Kasha behavior in terms of their emission, which occurs from the S_2_ state.^37^ They thus fall into the type I anti-Kasha category by Veys and Escudero,^19^ simply emitting directly back from the higher state by virtue of strong electronic NAC, and the virtually nonexisting emission from the (spectroscopically dark) Crt S_1_. The Crt S_2_ emission is weak, since the IC between the S_2_ and S_1_ states is very fast (subps range),^37,38^ forcing most of the excited population to undergo an S_2_→ S_1_→ S_0_ IC process. For Crts, the S_2_ state is known to be a potential source of EET to (B)­Chls,^14,38^ which is already an anti-Kasha process,^8^ yet not commonly designated as such.

Before we start comparing the natural pigment networks to systems with known anti-Kasha behavior, it should be noted that the experiments by Leupold and co-workers have shown that anti-Kasha behavior can be shown in Chls *a* and *b*.^39,40^ Their experiments enforced a large initial B band population, using a two-photon process (which can be considered best practice to investigate anti-Kasha effects).^8,41^ Consequently, emission from the B band was detected, with a yield comparable to that of Crts. Thus, there are parallels between Chls and Crts, with the difference that a dominant S_1_ (Q_*y*_) emission is found for Chls. The Chls are thus also, but less obvious than Crts, anti-Kasha type I emitters, in agreement with theoretical predictions.^17^

This indicates that Chls (possibly also BChls) and Crts have the potential for weak anti-Kasha type I emission. To understand how this affects EET, in this contribution, we analyze the corresponding coupling networks. After presenting the employed methods, we will follow with a direct comparison of the involved Förster coupling elements. Förster theory tends to underestimate the coupling between natural photosynthetic pigments,^9^ due to missing effects that are only incorporated when modeling excitonic behavior, e.g., via Generalized or multichromophoric Förster theory.^42,43^ It is thus helpful to use standard Förster theory with the aim to not overestimate EET.^44,45^ We will then continue with presenting excitons and the effects of pigment deletion on them. The remaining article will be concerned with the roles of Chl *b* or *c*-type (Chls *b*/*c* in the following) pigments and Crts, focusing on their effect on the B band vs their supposed supporting role in sunlight absorption. We will close with a summary and an outlook.

## 2 Methods

### 2.1 EET Network Model Systems

We chose a variety of well-studied light harvesting antenna complexes as the targets for studying their EET networks in the context of anti-Kasha potential: the peripheral antenna complexes of plant photosystem II (PSII), namely CP24, CP26, CP29 and the trimeric major light harvesting complex II, LHCII (all from PDB ID: 5XNL),^46^ and a fucoxanthin Chl binding protein, FCP, in its tetrameric form (tFCP) found for PSII of diatoms (from PDB ID: 7VD5).^47^ A pictorial representation of the PSII superstructures is provided in Figure S1 of the SI, focusing on the ratios of Chl *a* vs Chls *b*/*c*.

### 2.2 Förster Coupling Elements

To illustrate the coupling between the states in the Q and B bands, we computed Förster coupling elements^44,45^ V_ij_ between pairs of pigments *i* and *j*

1 $$V_{ij}=\kappa_{ij}\frac{\left|{\mathrm{\mu ⃗}}_{i}\right|\cdot \left|{\mathrm{\mu ⃗}}_{j}\right|}{n^{2}{\left|r_{ij}\right|}^{3}}$$

2 $$\kappa_{ij}=\left(\frac{{\mathrm{\mu ⃗}}_{i}}{\left|{\mathrm{\mu ⃗}}_{i}\right|}\cdot \frac{{\mathrm{\mu ⃗}}_{j}}{\left|{\mathrm{\mu ⃗}}_{j}\right|}\right)-3\left(\frac{{\mathrm{\mu ⃗}}_{i}}{\left|{\mathrm{\mu ⃗}}_{i}\right|}\cdot \frac{{\mathrm{r⃗}}_{ij}}{\left|{\mathrm{r⃗}}_{ij}\right|}\right)\left(\frac{{\mathrm{\mu ⃗}}_{j}}{\left|{\mathrm{\mu ⃗}}_{j}\right|}\cdot \frac{{\mathrm{r⃗}}_{ij}}{\left|{\mathrm{r⃗}}_{ij}\right|}\right)$$

with *n* being the refractive index of the medium (1.4 for all purposes here),^48^ κ_*ij*_ being the corresponding orientation factor of the transition dipole moments ${\mathrm{\mu ⃗}}_{i}$ , and ${\mathrm{r⃗}}_{ij}$ being the interpigment distance vector. For obtaining this distance, we used either the center of mass of the conjugated carbon atoms (for Crts) or the Mg coordinates (for Chls). It has been argued that this is not a good approximation for spatially close pigments, especially Crts,^49,50^ but our recent study has shown that for whole classes of pigments, the errors will likely cancel each other.^16^ Note that the “simple” Förster approach (site-based and involving point dipoles) provides a lower coupling between Chls than more elaborate models,^9^ which makes it ideal to see if an effect such as anti-Kasha EET exists at all. ${\mathrm{\mu ⃗}}_{i}$ was always oriented along the axis of two atoms in the structure, mimicking the Q_*y*_ or B_*x*_ state orientation from our earlier study.^34^ This approximation allows us to avoid recomputing each ${\mathrm{\mu ⃗}}_{i}$ orientation. The exact list of axes for each pigment and band can be found in the SI, Table S7. This table also contains the corresponding dipole strengths, which were reverse engineered from experimental spectra, using eq 1, Fermi’s Golden Rule (eq 3) and the empirical expressions for Förster EET rates (see SI for the full procedure).

It should be noted that we treat the various electronic states within the Q or B bands as a single-state entity. This is simply due to our recent studies indicating a strong dynamic population exchange between these states after they are excited,^32,51^ which simplifies the model substantially. As an additional practical advantage, the main absorbing/emitting state in the B band is unknown. For eq 1, we had to choose a singular value for each $\left|{\mathrm{\mu ⃗}}_{i}\right|$ , and the reverse-engineering process outlined in the Supporting Information produces effective values without needing an explicitly assigned electronic state. Further, the employed values are also very close to those experimentally reported (e.g., for Chl *a*, $\left|{\mathrm{\mu ⃗}}_{Q}\right|$ was computed as 6.45 D, with a reported value^52^ of 5.8 D at *n* = 1.35).

### 2.3 Spectra and Site Energies

We built our Förster model in a semiempirical way, using experimental spectra (see Figure S2 or sec. 4 of the Supporting Information for the corresponding sources). For the individual Chls, site energy shifts were applied, derived from computational literature or our own calculations (see below and sec. 4 of the Supporting Information). These shifts effectively reflect the individual protein environment of each Chl binding site. Crts were not energetically shifted. Aside from one exception, all shifts are less than ±0.1 eV and do not affect the qualitative picture; only the order within each pigment class changes slightly if the shifts are neglected (data not shown).

A detailed list of the sources and values for the site energy shifts can be found in the Supporting Information, Table S8. The shift values were acquired from earlier publications (CP29),^15^ used in analogy to CP29 (CP24 and CP26) or newly computed for this contribution (LHCII) following our previously^15^ established protocol: Calculations were performed using the (time-dependent) CAM-B3LYP/6–31G* level of theory, employing a hybrid quantum mechanics/molecular mechanics (QM/MM) preoptimization scheme (Gaussian16 and Gromacs software, Amber99 force field).^53–61^ Q-band shifts of FCP were obtained from the recent contribution of Maity and co-workers,^62^ and B band shifts were assumed to be identical to Q band shifts for the lack of any respective source. For more details on the QM/MM protocol, see sec. 4 of the Supporting Information.

### 2.4 EET Rates and Efficiencies

For an efficient EET process, *V_ij_* (eq 1) is not the only decisive factor. Instead, the rate of Förster EET (Förster resonance energy transfer, FRET) via Fermi’s Golden Rule is^63^

3 $$k_{FRET}=\frac{2\pi }{\hbar }{\left|V_{ij}\right|}^{2}\rho =\frac{2\pi }{\hbar }{\left|V_{ij}\right|}^{2}\frac{\int_{0}^{\infty }a_{i}\left(\lambda \right)F_{j}\left(\lambda \right)\lambda^{4}d\lambda }{\int_{0}^{\infty }a_{i}\left(\lambda \right)F_{j}\left(\lambda \right)d\lambda }$$

with *ℏ* being the reduced Planck constant and ρ being the density of interacting states between pigments *i* and *j* (i.e., the λ^4^-weighted integral over the absorption *a* of the acceptor *i* and the emission *F* of the donor *j* at wavelength λ; note that the corresponding units are of no concern as the spectra are become normalized here). The emission of the donor is however energetically shifted compared to its absorption, which is especially important for the Crts (Figure S1 in the Supporting Information), but also for the Chl B band.^39^ Quantum mechanical models, including our own, often neglect this aspect, making V_ij_ the seemingly decisive entity for *k*_FRET_, which may not be appropriate here. Unfortunately, the common, experimental definition of the overlap as used in our earlier studies does not normalize the absorption spectrum of the acceptor,^17,18^ and thus we need to compute ρ here anew, using the expressions outlined elsewhere.^16^ The computed values for ρ can be found in Table 1 of the results section.

**Table 1:** Density of States ρ, in 10^–6^ cm, and EET Rate *k*_FRET_, in ns^–1^, for Various Chl Donor (rows)/Acceptor (Columns) EET Pairs^a^

	acceptor		
Q band	Chl *a*	Chl *b*	Chl *c1*
donor	ρ	ρ	ρ
Chl *a*	450.05	77.93	8.86
Chl *b*	356.60	451.69	87.26^b^
Chl *c1* ^c^	228.30	372.45^b^	260.67
	acceptor		
B band	Chl *a*	Chl *b*	Chl *c1*
donor	ρ	ρ	ρ
Chl *a*	113.05	221.58	225.20
Chl *b*	16.76	174.08	106.84^b^
Chl *c1* ^c^	16.85	261.74^b^	193.46

a The *V_ij_* values used for each chromophore class were taken from Figure 3, either from CP24 or (for Chl *c1*-containing pairs) from tFCP.

b Chl *b*-*c* pairs are not found in vivo, hence no avg. V_ij_ available for *k*_FRET_ calculation.

c No experimental spectra for B band emission available, approximated, see Supporting Information.

Further, we compute EET efficiencies, as the ratio of all EET rates vs all EET and IC rates

4 $$E_{EET}=\frac{\sum k_{FRET}}{\sum k_{FRET}+\sum k_{IC}}=\frac{\sum k_{FRET}}{\sum k_{FRET}+\sum \tau_{IC}^{-1}}$$

### 2.5 Excitonic Interactions

Excitonic Hamiltonians **H** were constructed from the site energies ε_i_ and coupling elements V_ij_

$$H=\left(\begin{array}{llll} \varepsilon_{1} & V_{12} & \cdot \cdot \cdot & V_{1n}\\ V_{21} & \varepsilon_{2} & \cdot \cdot \cdot & V_{2n}\\ \vdots & \vdots & \ddots & \vdots \\ V_{n1} & V_{n2} & \cdot \cdot \cdot & \varepsilon_{n} \end{array}\right)$$ 5

Diagonalization of **H** yields excitonic energies as eigenvalues and the contribution coefficients of the pigments to each exciton as eigenvectors. The squared coefficients give the weight with which each pigment participates in a particular exciton in percent. Different from the Förster rate model, individual excitation energy values have to be provided for each of the pigments in **H**, enforcing the need for a separate B band Hamiltonian **H**_B_ and a Q band Hamiltonian **H**_Q_. For those, Chl energy values were determined by the respective experimental maxima plus the corresponding site shift value. For Crts, the energy values for **H**_B_ were taken from the maximum of the energetically lowest peak in the broad, bright Crt signal (which roughly corresponds to the 0–0 excitation). For **H**_Q_, we approximated the potentially lowest Crt emission energy by subtracting 0.8 eV, following experimental and computational evidence for the strong relaxation potential of this state before emission.^64,65^ The resulting eigenvectors were analyzed with a variant of the (inverse) participation ratio representing the delocalization of the state^66^

6 $$IPR^{-1}={\left(\sum_{N}c_{i}^{4}\right)}^{-1}$$

IPR^–1^ represents how much the eigenvector is distributed over its *N* elements (the pigments), based on the eigenvector coefficients *c*_*i*_.

#### 2.5.1 Other Methods

We further computed contributions for each pigment group for the initial sunlight absorption process of each target system investigated, using a Lambert–Beer approach; corresponding details can be found in secs. 5 and 6 of the Supporting Information, along with how to compute the individual pigment group contributions.

## 3 Results and Discussion

### 3.1 Förster Coupling Elements

Due to the differences in absorbance between Chl Q and B bands, the V_ij_ values involving the Chl B band can be expected to be generally larger for than for Q. Figure 7 illustrates the corresponding average V_ij_ elements in our model Hamiltonians **H**_**B**_ and **H**_**Q**_. Note that we applied a scaling factor of 3 (LHCII) or 4 (tFCP) in Figure 7 to make the averaged V_ij_ elements comparable to the monomeric cases (CP24, CP26, CP29). Without scaling, the averaged LHCII/tFCP coupling elements would seem much weaker, since LHCII and tFCP contain several near-zero V_ij_ values simply due to the distance between several pigment pairs *ij* located in different monomeric subunits of LHCII and tFCP.

**Figure 4:**
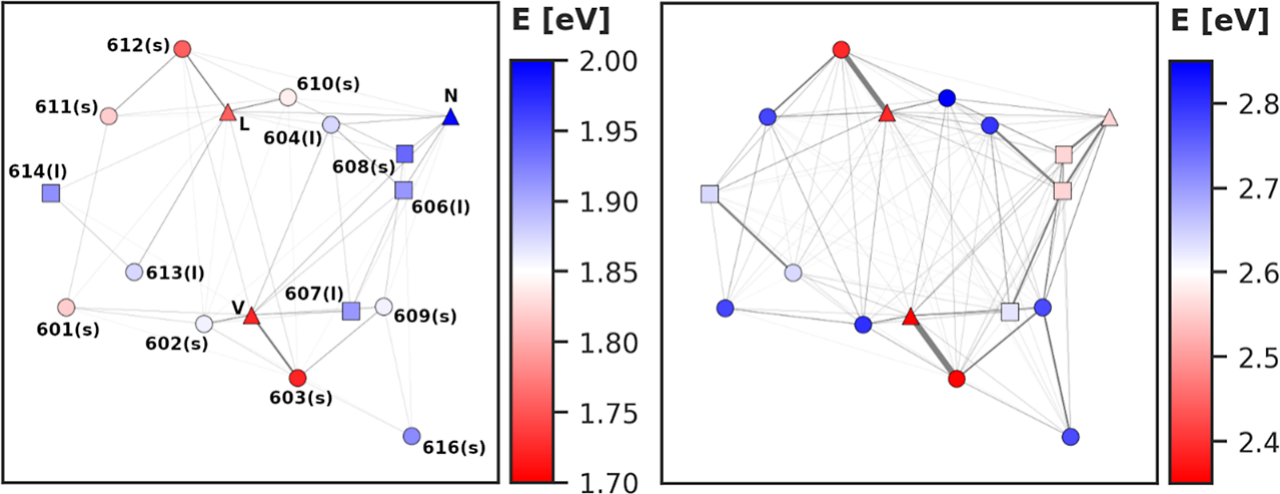
CP29 coupling network for Q (left) and B (right) bands. Pigments are indicated by symbols, Chl *a* (○), Chl *b* (□) and Crts (Δ). Color code indicates energy of the lowest exciton on each pigment *i*with *c*_*i*_^2^ of 0.1 or more. The thickness of connecting lines represents the strength of the corresponding coupling elements. Visualized from the stromal side of the thylakoid membrane, projected to an *X*/*Y* plane. Chls labeled by their PDB structure number with (s) and (l) indicating the stromal or lumenal positioning. Crts abbreviated as (L)­utein, (V)­iolaxanthin and (N)­eoxanthin.

**Figure 5:**
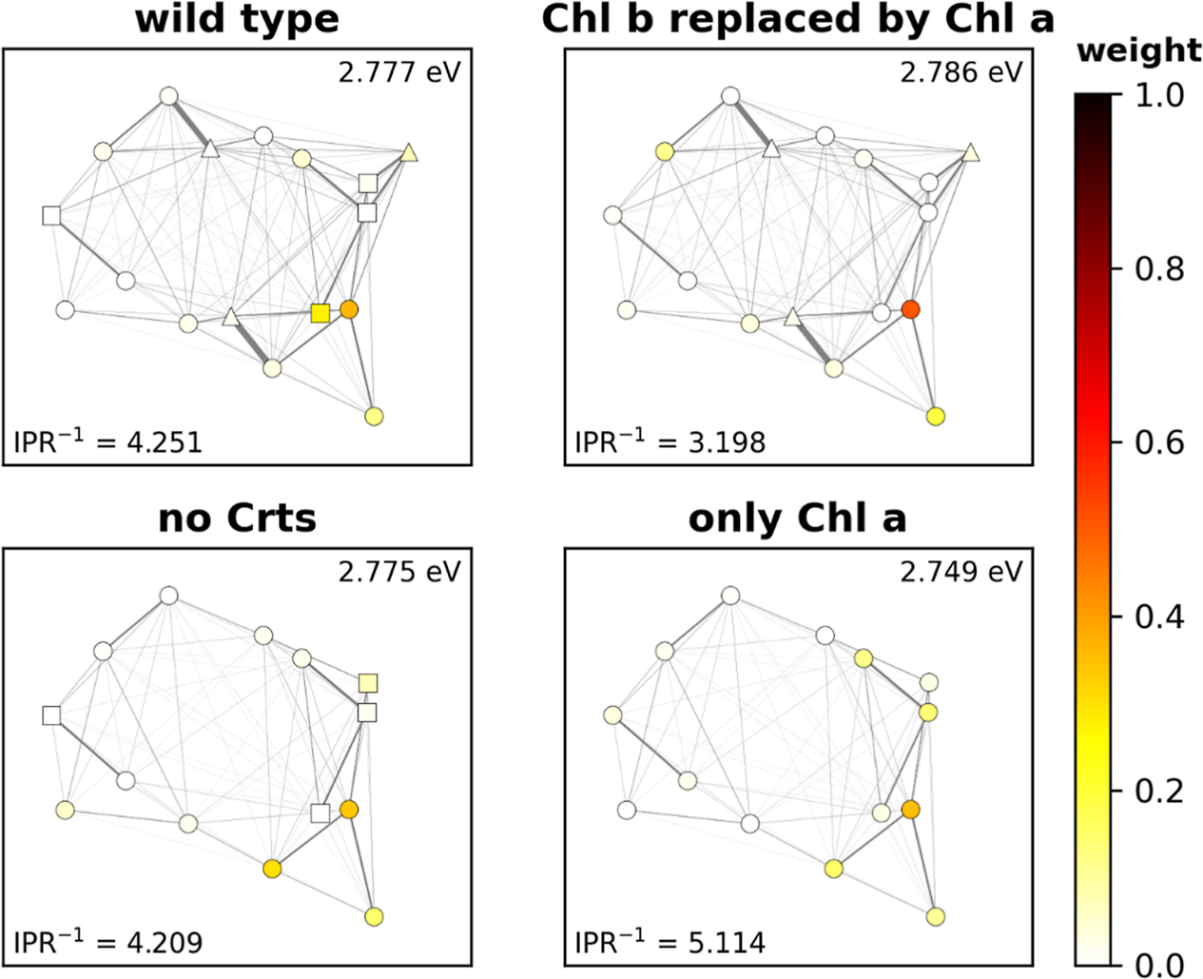
Detailed distribution of the B band excitons related to mainly Chl 609, in CP29 using different pigment configurations. The color code indicates the contribution of the pigments to the exciton. Energy of the excitons listed in the upper right corners, delocalization criteria in the lower left corners. See Figure 4 for pigment labels and details on the representation.

**Figure 6:**
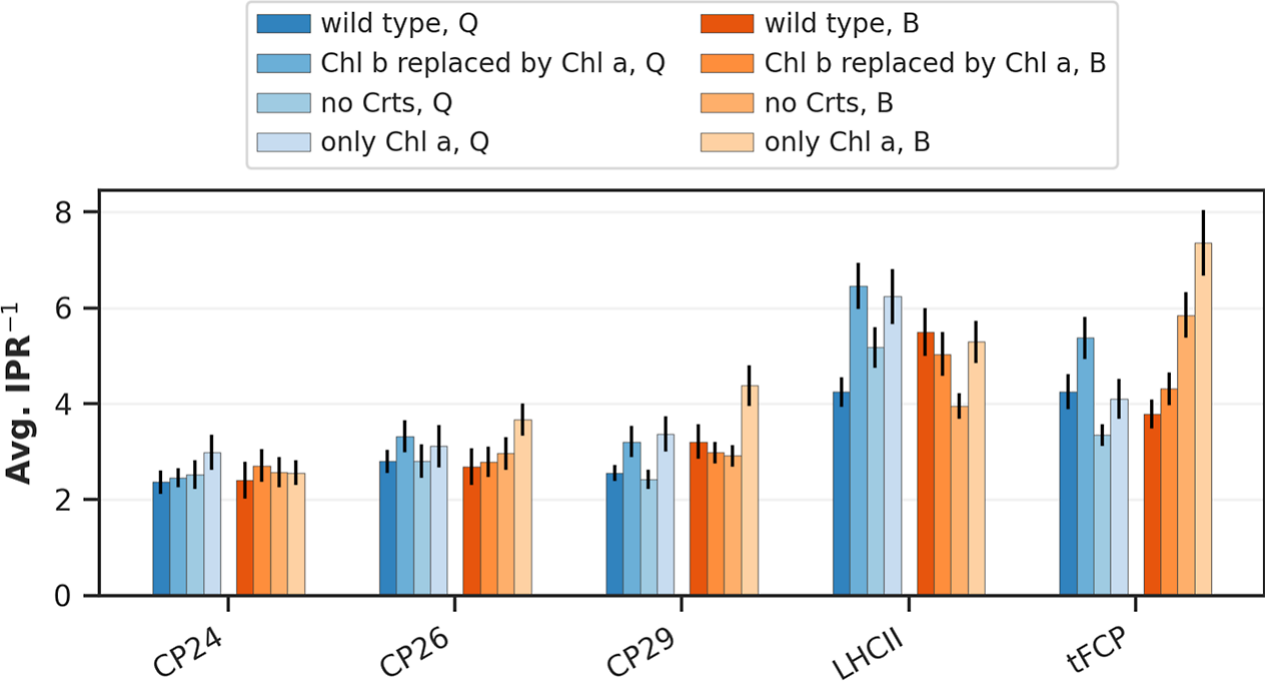
Exciton delocalization, for all investigated systems and pigment configurations; for Q band (blue bars) and B band (red/orange bars). For the Q band, the effect of Crts is likely exaggerated, as we allowed “relaxed” Crt states to participate in the corresponding networks; the constant intermixing of Crt states in the Q band energy range is not likely. Standard error of the mean indicated as black bars.

The average couplings shown in Figure 7 between Chls *a* in the Q-band is in all investigated cases ranging from 20 to 38.0 cm^–1^, indicating that this strength of coupling suffices for efficient EET between the long-lived (τ_*Q*_ = 6.3 ns)^67^ Chl *a* Q band excitations. Our Q band couplings agree well with those reported previously for CP29 and LHCII (with exceptionally strong coupled pairs starting at 40 cm^–1^ and higher),^68^ the photosystem I case,^69^ as well as for both bands when computed with the transition density cube approach that accounts for the spatial complexity of the Crt pigments.^16^

Concerning the potential for anti-Kasha EET processes along the short-lived B band (τ_*B*_ ≈ 100–250 fs),^11–13^ Figure 7 shows that B–B or B–S_2_ coupling elements are in most cases about 3 to 4 times larger than for the Q band. Especially the interaction between the Crts and the Chl *c1* B band is strong (849 cm^–1^ after scaling). This high value may have to be taken with care, but our comparison to the Zn-porphyrin system (see below) indicates that it should be indeed trustworthy. Between pairs of Chl *a* and Chls *b*/*c* in the B band, values range from 70 to 235 cm^–1^. The lowest value arises from Chl *a* pairs in tFCP, where Chls *a* are spatially more distant in comparison to the other complexes; the highest value is also found in tFCP, for interactions between Chls *c1*; this is due to their comparatively strong B band absorption.

For anti-Kasha potential, we can compare to a structurally very similar case, namely the windmill Zn-porphyrin arrangements of Nakano and coworkers.^7^ The comparison might seem flawed at first, since Zn-porphyrins have longer B band lifetimes (10–20 times more) than Chls.^13,70^ Still, the authors report EET also from a donor with B band lifetime similar to that of Chl *a* (octaethylporphyrin, 150 fs), noting that this is likely an effect of the short donor–acceptor distance.^7^ We compute the corresponding V_ij_ (see Supporting Information for details) for a CP24-like network using their weaker Soret coupling Zn-porphyrin compounds, giving an average V_ij_ of 216 cm^–1^. When only considering V_ij_, these values indicate that at least Chls *b*/*c* (and likely Crts as well) have potential for anti-Kasha behavior, even when not involving the potentially much faster Dexter-type EET.^71^

### 3.2 EET Rates

Despite this strong analogy between the Zn-porphyrins and Chls, the averaged V_ij_ values on their own provide no clear evidence for or against anti-Kasha behavior. High coupling may (for Crts-Chls and Chls *b*/*c* pairs) or may not (Chl *a*) apply. Therefore, we used Fermi’s Golden rule (eq 3) to calculate Förster transition rates *k*_*FRET*_ from the coupling V_ij_ and the density of interacting states ρ, providing a more balanced perspective (Table 1).

The order of ρ values found for the Chl *a* and Chl *b* pairs (Table 1) is identical to that found before for the respective spectral overlaps,^18^ validating the approach. The ρ values are mostly within 1 order of magnitude, aside from a few spectroscopically unfavorable pairs such as, e.g., donation from Chl *a* to Chl *c1* in the Q band. B band ρ values tend to be lower than Q-band ρ values, which can be attributed to the involved λ^4^ scaling.^16^ Overall, however, ρ does not vary so much as that one could immediately exclude any of the donor/acceptor pairs in terms of their EET capability. One must thus explicitly consider *k*_*FRET*_ and compare them to the corresponding IC rates (Q→S_0_ or B→Q). The corresponding *k*_*FRET*_ values are found in Table 1 as well.

To put the corresponding rates into perspective, we conduct two example calculations. First, for the Q-band, the case of coupling between pairs of Chl *a*, for which the average *k*_FRET_ in CP24 is found to be about 768 ns^–1^. The resulting EET efficiency (eq 4) for a single pair of Chls *a* is larger than 0.999, corroborating the strong EET efficiency known for the Q band. Performing the same calculation for the Chl *a* B band (1855 ns^–1^) “only” yields an *E*_EET,B_ of 0.156. However, as noted in our earlier contributions,^17,18^ all EET pairs compete with a single IC process. Including all 10 acceptor Chls (i.e., not even accounting for the Crts) in CP24 yields a B band *E*_EET,10,*B*_ of 0.650. For these examples, we only used the rates given in this paragraph, 6.3 ns Q band lifetime^67^ and a B band lifetime of 100 fs, which is on the lower end of the reported values, biasing against EET.^11–13,51^ For similarly computed efficiencies in the CP29 antenna system, please refer to our previous contributions.^17,18^ The semiempirical Förster model thus indicates a strong potential for anti-Kasha processes in these systems, since an absorbing Chl site would be more likely to perform B band EET before IC, just as shown in Figure 1, right.

### 3.3 Exciton Formation

In this section, we expand our perspective beyond the purely site-based approach. Anti-Kasha processes are, at their core, resulting from relocating the excited state population after absorption (Figure 1). We therefore check for the potential of both Q and B band exciton formation. The corresponding exemplary eigenvalues of **H**_**Q**_ and **H**_**B**_ are shown for the CP29 case in Figure 4 (other systems are presented in the SI). It is found for the Q band that the highest energy excitons are unsurprisingly located at Chls *b* and neoxanthin, since those have comparatively high **H**_**Q**_ eigenenergies. In the B band, the situation is mostly inverted; again, this follows the energetic order of states and is therefore expected. The prominent outliers in the B band are two Chls *a* (603 and 612), which are strongly coupled to nearby Crts and thus found in the lowest B band excitons.

A detailed discussion of each system’s excitons would go far beyond the limits of this article. Instead, we compare the overall properties of the Q and B band excitonic networks using an estimate of the delocalization (IPR^–1^, eq 6). IPR^–1^ ranges between 1 for an exciton localized entirely on one pigment, to N (number of pigments in the complex) for an exciton distributed equally over all pigments. The corresponding results can be found in Figure 6. Though IPR^–1^ may rise with larger system size (as the upper limit increases), many excitons are localized at only a few dominant pigments (see Supporting Information for visualization of each exciton for all systems). The potential for larger IPR^–1^ values is nicely seen when comparing small (CP24) to large (tFCP) systems in Figure 6.

Overall, the Q and the B band show similar IPR^–1^ values and trends (Figure 6). This indicates that both bands should exhibit a similarly strong exciton network. Figure 6 also shows the effects of varying the pigment configuration, in analogy to our previous contributions.^17,18^ For this, we exchanged all Chl *a* pigments by Chl *b*, removed the Crts from the network or performed both changes. The changes in the average IPR^–1^ show that a pure Chl *a*-based network is in nearly all cases more delocalized, especially in the B bands of CP26, CP29 and tFCP. To explain this, it must be noted that a purely Chl *a*-based network is energetically more homogeneous than a mixed pigment configuration. An energetically homogeneous network is then more likely to result in many highly similar excitons (as a trivial result of the matrix diagonalization) with an evenly distributed population. Introducing other pigments such as Chls *b*/*c* or Crts will thus likely perturb this ideal picture, leading to the observations in Figure 6.

To illustrate this further, we select analogous excitons between the different CP29 models and show how they are affected by the pigment configuration changes. The example excitons must be of similar character between the four configurations, and ideally they contain only minor Crt site coefficients. We chose the exciton that is characterized by the highest Chl *a* 609 coefficient, as the corresponding Crt population is only 8% (WT) and 5% (Chl *b* replaced by Chl *a*). The result is shown in Figure 5; the excitons become gradually more delocalized when going from the WT to the “only Chl *a*” configuration. However, it also becomes obvious that the simple IPR^–1^ criterion does not suffice to capture the full spatial character of delocalization (e.g., IPR^–1^ is low for the case when Chls *b* are replaced by Chl *a*, despite a clear, spatially distant contribution by residue 611; this will be addressed in a future contribution).

For anti-Kasha processes, the above analysis first and foremost shows that the B and Q bands have the same potential for exciton formation. It further suggests that the presence of Chls *b*/*c* and Crts reduces delocalization of the excitons, which is counterintuitive at first. This effect is more pronounced for the B band; in this regard, we have already discussed before that the accessory pigments may also serve as B band energy sinks.^17,18^ This is also nicely illustrated by the energetic order of the computed full exciton sets (see Supporting Information) which displays the lowest B band excitons to be mainly located at Crts and Chls *b*/*c*.

The above analysis allows us to quantify the effect of accessory pigments for “breaking” the B band excitons, which would be a new, photoprotective role. The last part of this results section will therefore be dedicated to weighting the so-far acknowledged main role – absorption increase–against the effect discussed above.

### 3.4 Impact of Crts and Chls *b*/*c* on Total Absorbance

One of the main acknowledged roles of accessory pigments, such as Crts and Chls *b*/*c*, is a broadening of the spectral range for effective photon absorption. It needs to be pointed out, however, that this is a handwaving argument, as *any* additional pigments (even simply adding more Chl *a*) will yield an increase in total absorbance. While this is likely a beneficial effect under low light conditions,^72^ it does not mean that it warrants biosynthesis of Chls *b*/*c* over Chl *a*.^22,73^ For the Crts, on the other hand, important functions beyond absorption are long known (e.g., Chl triplet quenching).^74^ Judging by the detrimental potential of Chl triplets, the improved absorbance inferred by Crts actually appears to be a beneficial side effect, unless in specialized light harvesting systems such as peridinin-Chl *a*-proteins.^75^

To assess the actual impact of mixing different numbers of pigments for the total photon absorbance, we present the corresponding contributions according to a Lambert–Beer model (see Supporting Information for details and absolute values in Table S9). Additional analyses to check the validity of our Lambert–Beer model can be found in the Supporting Information (Tables S10 and S11).

The differences induced by each pigment class with respect to the WT pigment configuration are plotted once for the full spectrum and once only for the B band in Figure 7. All investigated Chl *b*-containing complexes (i.e., all except tFCP) gain only about 0.05 in relative absorbance by the inclusion of Chl *b*, regardless of the Chl *a*:*b* ratio or the total Chl count. This is in all cases 10% or less of their absolute photon absorption (see Table S9). The inclusion of the stronger absorbing Chl *c1* in tFCP induces an absorbance gain of 0.07 at sea level, which is also less than 10% of the respective total WT absorbance. As FCPs are known to be especially interesting for deep sea levels,^76^ where FCP-producing diatoms largely have to thrive on blue light,^24^ we also compare to a deep-sea solar irradiation spectrum (see also Figure S4 in the SI). We find that the Chl *c1*-induded absorbance remains insignificant (except for the hypothetical tFCP without Crts, for which inclusion of Chl *c1* improves absorption by about 70%). Figure 7 further shows that the net gain in initial absorption by adding Chls *b*/*c* for a single complex does not depend on the presence of Crts (dark vs light green bars, see sec. 5 in the Supporting Information for the detailed expressions behind the bar heights). Difference spectra between varied pigment configurations of LHCII and tFCP (Figure S4, Supporting Information) rationalize this point, since absorbance gains induced by Chl *b*/*c* in some spectral regions are offset by net losses in other regions.

**Figure 7:**
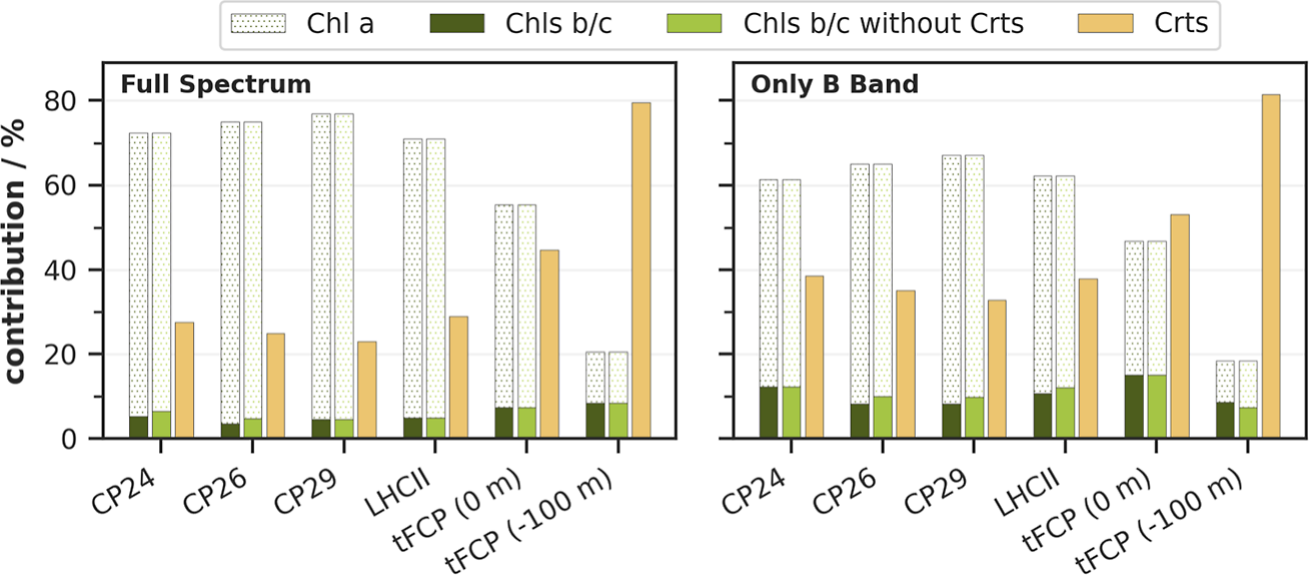
Contribution of different pigments to the total absorbed photons of various LHC variants in the range of 350 to 800 nm (full spectrum, left) or to 500 nm (B band, right) under irradiation with sunlight at sea level (0 m), 21 June noon, 50° N latitude.^101^ For tFCP, the irradiation at 100 m below sea level was tested as well (see also Figure S4 in the Supporting Information).^102^

In contrast, Crts provide much larger gains in relative absorbance; especially in the Crt-rich tFCP, even more so at deeper oceanic levels. The actual effect of Chls *b*/*c* for total absorbance is thus comparatively weak. Only considering the B band slightly elevates the relative contributions of the accessory pigments, since this region is characterized by a comparatively weak Chl *a* absorbance. Crts are found to outcompete the Chls *b*/*c* in this regard by approximately *a* factor of 5 for plant LHCs, up to *a* factor of 10 for tFCP at 100 m oceanic depth. This might be considered an unfair comparison, since Chls *b*/*c* are replacing Chl *a*, while Crts are simply added. Still, it needs to be remembered that only 3–4 Crts are added per LHC monomer (or 4–7 per FCP monomer). Comparing the WT to a hypothetical pigment configuration without Crts (Table S9, Supporting Information), it becomes apparent that adding Crts increases the total absorbance by about 50% in LHC monomers, nearly 100% in tFCP at sea level and more than 300% at deeper oceanic levels. This clearly underlines the much higher efficacy of Crts in terms of increasing absorbance compared to the Chls *b*/*c*.

In summary, this section shows that the actual gain in absorbance by introducing Chls *b*/*c* is only in the range of 5%–10%. It can be expected to be even less when considering the realistic case of a PSII supercomplex, for which the RC contains usually only Chl *a* (or pheophytin) and β-carotene as main pigments.

## 4 Summary and Conclusions

In this work, we tested Kasha and anti-Kasha models for some of the most important pigments and several pigment-protein complexes in natural photosynthesis. To do so, we calculated various parameters relevant for EET, not only for the commonly investigated Q band, but also for the often-neglected B band. We compared the computed parameters to those known for established anti-Kasha pigments, and found that the natural photosynthetic pigments should exhibit B band EET.

(i) Experimentally, it is already known that Chls and Crts are potential anti-Kasha emitters,^14,39^ when investigated with the appropriate spectroscopic methods. This is easily explained as the rate of spontaneous emission scales with absorbance, and the corresponding states have very strong extinction coefficients.
(ii) The resulting B band Coulombic coupling elements V_ij_ are found to be 3-to 4 times larger than those found in the Q band.
(iii) The spectral density ρ tends to be higher for the Q band (due to the λ^4^ scaling), but remains within 1 order of magnitude between Q and B.
(iv) The computed EET rates *k*_FRET_ depend on the pigment types but are typically larger for B than for Q.
(v) The resulting *k*_FRET,*Q*_ are strong enough to perform efficient Q band EET vs a comparatively slow IC to the ground state (as found in experiment). *k*_FRET,*B*_ can however also maintain EET processes, which become efficient when considering a network of multiple acceptors vs an ultrafast, but singular IC process.

The above insights are based on a site-based Förster approach, which neglects several aspects which would contribute to an anti-Kasha behavior, most notably, Dexter-type energy transfer^71^ and exciton formation. When including the latter, we found similar delocalization in both spectroscopic bands (Q and B), suggesting that the excitations might be much more connected than already found with Förster coupling. They become more delocalized without the accessory pigments, as expected from a more homogeneous network. This effect is more pronounced for the B band than for Q. We further find that absorption gains and delocalization are affected by Crts and Chls *b*/*c* in a similar fashion, namely strong changes when removing Crts vs. small changes when exchanging Chl *a* for Chls *b*/*c*. In a future contribution, however, more elaborate analysis of this effect is required, including the spatial information to assess the changes in exciton delocalization.

Overall, the benefit of Chls *b*/*c* for the overall absorbance is low, with less than 10% total gain. Even though this gain is surely beneficial under low-light conditions, we suggest here that Chls *b*/*c* might primarily serve other functions: Disrupting the B band delocalization outlined above, or other roles, resulting from spectral competition with Chl *a* and the overall lower B band energies of Chls *b*/*c*.^17,18^ This interpretation would also explain the large spectral overlap between the pigments (which would be counterproductive in an antenna) and the remarkable identity of Chl *a* B band emission and Chl *b*/*c* B band absorption.^39^

As noted in previous work, we suppose that photosynthetic organisms have evolved these mechanisms to prevent potentially harmful IC processes *inside the RCs*, e.g., close to the special pair or the oxygen evolving complex (for PSII).^77,78^ We have illustrated this hypothesis based on our present data in Figure 8. IC localized near the RCs would expose the fragile machinery of electron transport to increased heat stress. Since photosystems are in constant need for repair, alleviating this issue by supporting “peripheral” IC appears to be a viable, yet so far neglected strategy of photoprotection. The strategy may have yet eluded experiment simply due to the involved time scales as well as the pigments being assigned other, more intuitive roles. This work thus removes the implicit contradictions between the acknowledged EET pathways for natural and artificial light harvesting systems: The apparent lack of anti-Kasha EET in natural systems is likely due to the presence of accessory pigments. Via even faster IC, exciton disruption and lower B band energies, Crts and Chls *b*/*c* might turn the highly coupled Chl *a* network back into an apparently Kasha-like system.

**Figure 8:**
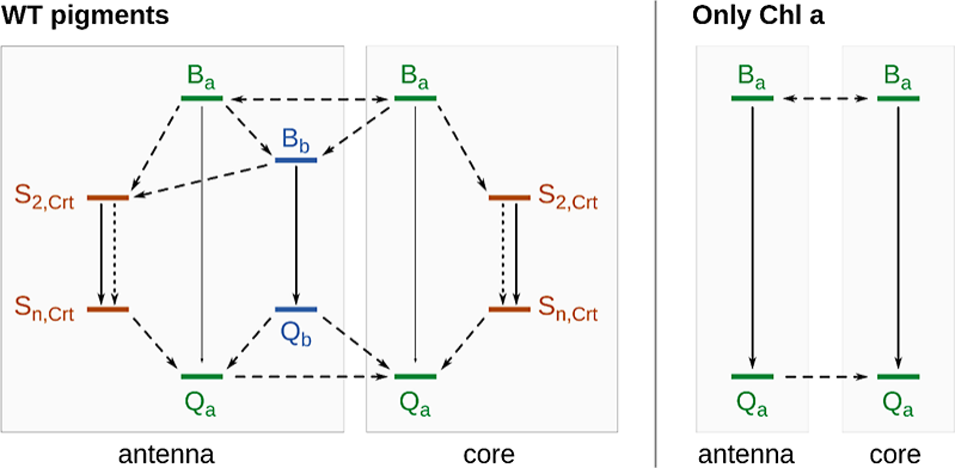
Schematic representation of energy transport events in an anti-Kasha photosystem complex. Dashed arrows: EET, Solid arrows: IC; Dotted arrows: vibrational relaxation. S_n_: Relaxed Crt state of unclear electronic character. Other states belong to the Chl Q or B band, and to Chls *a* or *b*, as indicated. A purely Chl *a* network (right side) would likely experience higher IC (and thus heat stress) at the core complexes due to energetic funneling; the WT (left side) configuration exhibits less Chl *a* absorbance of blue light as well as competing elements that redirect IC/heat to the antenna complexes.

## Supplementary Material





## Notes

Additional resources/data sets are available at 10.17169/refubium-47543. The Supporting Information is available free of charge at https://pubs.acs.org/doi/10.1021/acs.jpcb.5c02465.

Structural overview and Chl pigment ratios. Spectra of several naturally occurring carotenoids. FRET parameters. Details on site energy calculations and sources. Relative absorption, details on Figure 7 and Zn-porphyrin coupling elements. Testing the Lambert–Beer model parameters. Difference spectra for LHCII and tFCP. Eigenvectors and eigenvalues (excitons) of all investigated complexes. Additional references (PDF)
(ZIP)

## Notes

This paper is published in memory of our valued friend and colleague Prof. Regina de Vivie-Riedle, whose insight, mentorship and passion for science continues to inspire our research.
