# Supplementary material for: Applying an Anti-Kasha Model Resolves Differences Between Photosynthetic and Artificial Pigments
Source: J Phys Chem B. 2025 Jul 23;129(31):7884–95. doi: 10.1021/acs.jpcb.5c02465 (PMC12337091; doi:10.1021/acs.jpcb.5c02465)
Supplement: Supplementary file 1 [file jp5c02465_si_001.pdf]

Applying an Anti-Kasha Model Resolves Differences Between Photosynthetic and Artificial Pigments

Jan P. Götze,[1,*] Simon Petry,[1] Sebastian Reiter,[2] Heiko Lokstein[3] and Regina de Vivie-Riedle[2]

[1]Freie Universität Berlin, Fachbereich Biologie Chemie Pharmazie, Physikalische und Theoretische Chemie, Arnimallee 22, 14195 Berlin, Germany

[2]Ludwig-Maximilians-Universität München, Department Chemie, Butenandtstr. 5-13, 81377 Munich

[3]Department of Chemical Physics and Optics, Charles University in Prague, Ke Karlovu 3, 121 16 Prague, Czech Republic; current address: Luckauer Str. 12, 15907 Lübben, Germany

**Corresponding author, email: jan.goetze@fu-berlin.de*

Supporting information

Table of contents:

1. Structural overview and Chl pigment ratios
2. Spectra of several naturally occurring carotenoids
3. FRET parameters
4. Details on site energy calculations and sources
5. Relative absorption, details on Figure 7 and Zn-porphyrin coupling elements
6. Testing the Lambert-Beer model parameters
7. Difference spectra for LHCII and tFCP
8. Eigenvectors and eigenvalues (excitons) of all investigated complexes
9. References

1. Structural overview and Chl pigment ratios

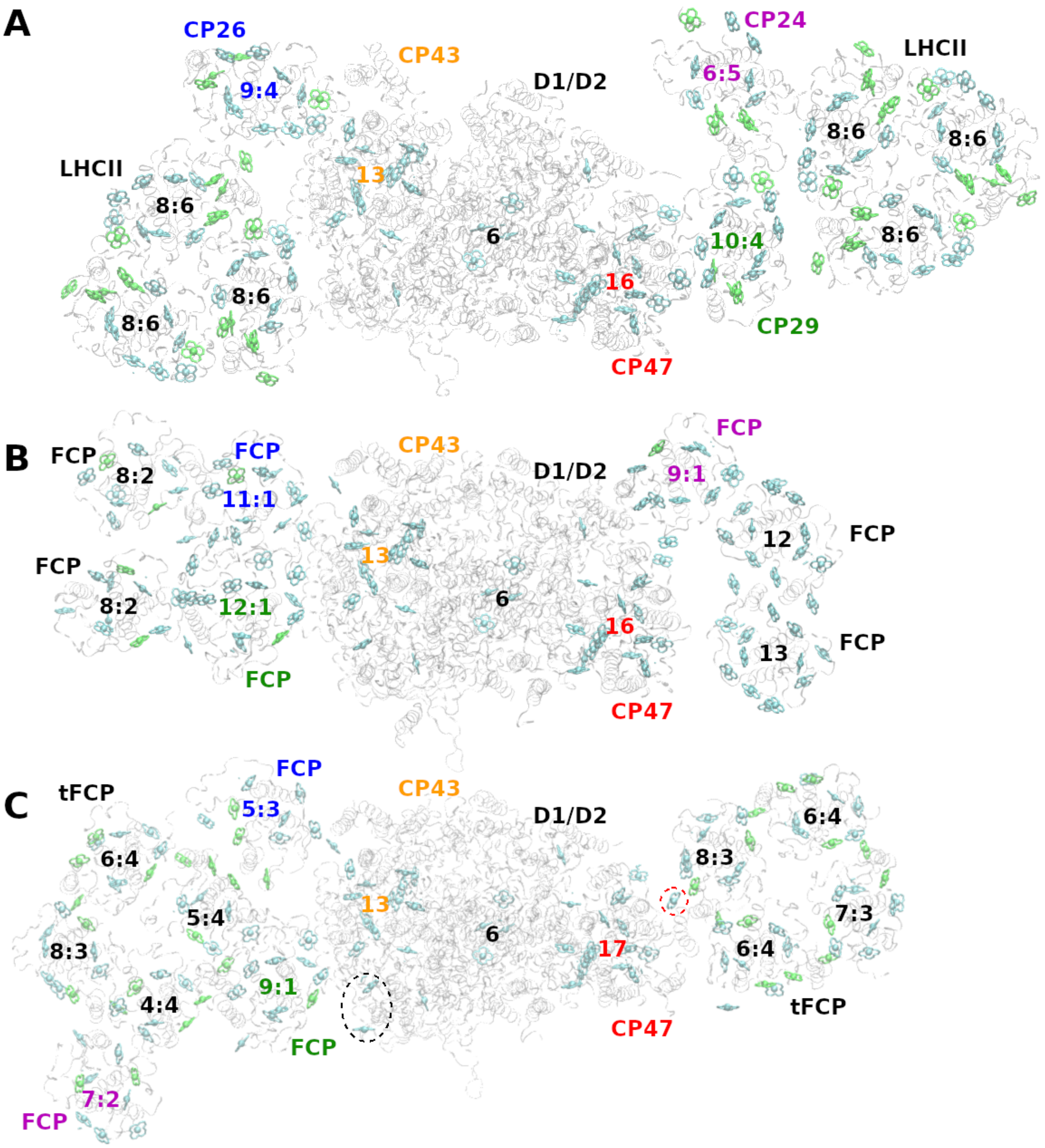

Figure S1: Chl a : Chls b/c ratios of different photosystem II assemblies, visualized using the examples of a plant (A, Pisum sativum) and two diatoms (B, Thalassiosira pseudonana, and C, Chaetoceros gracilis). Visualized regions were cut from full structures, found in PDB entries 5XNL, 8IWH and 7VD5, respectively.[15,16,40] *Chl a shown as cyan, Chls b/c as green Mg-porphyrins. Color code of subcomplex labels and corresponding numbers is a visual aid, not an indicator of functional equality/analogy. The red circle in (C) shows the additional Chl a in CP47; the black circle two, otherwise omitted, Chl a in the PSII core complex. The left tetrameric FCP (tFCP) complex of (C) has various chains that structurally contain Chls of adjacent subunits; individual subunit Chl ratios for this specific region must therefore be taken with care.*

2. Spectra of several naturally occurring carotenoids

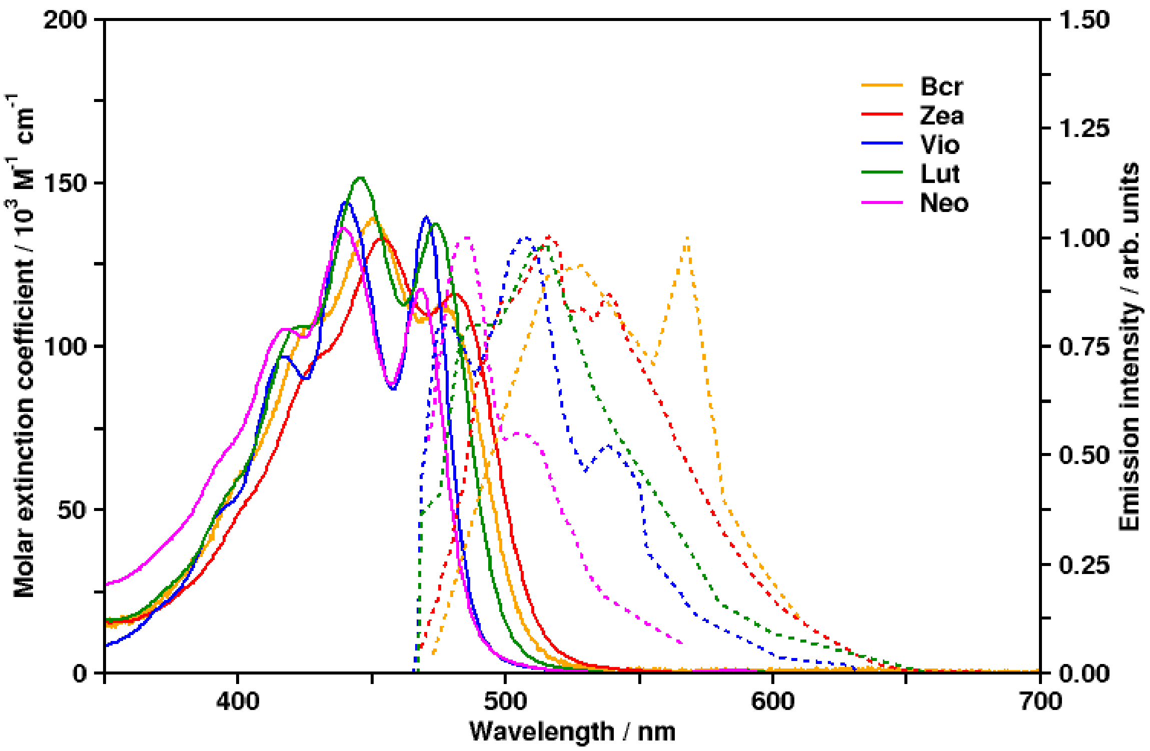

Figure S2: Overview of various carotenoid absorption (solid lines) and emission (dashed lines) spectra. The emission spectra are weak and thus normalized (right vertical axis) for better visibility. Absorption data mostly from the PhotoChemCAD database,[41] where applicable, other sources and abbreviations are β-carotene[13] (Bcr), zeaxanthin[42] (Zea), violaxanthin[42] (Vio), lutein[43] (Lut) and neoxanthin[13,44] (Neo). Emission for Neo is taken from octaene (same length of conjugated system as Neo), since no Neo emission spectrum was available.

3. FRET parameters

FCP pigment emission spectra

B band emission of Chl *c1* is not reported to our knowledge, requiring an approximated spectrum. We created it by mirroring the B band absorption spectrum and then applying a Stokes shift of -0.05 eV. Note that the Stokes shift affects the spectral overlap J (see our earlier work for the employed expressions),[1] so we restrict our model to the experimentally known values in all cases, if applicable. For the case of Chl *c1-c1* B band coupling, changing the shift to -0.10 eV would drop the J value by about 40% (from 39.0, see Table S1, to 23.8, test calculation). Hence, we use the FCP case only for qualitative comparison where explicit rates are concerned. For the FCP-Crt pigments (fucoxanthin and diadinoxanthin), we used the same approximation as for neoxanthin, due to the structural similarity of the pigments; we thus substituted the missing emission by the spectrum of octaene.[2]

Dipole moment strengths – Chls

To obtain the Förster coupling elements V_{ij}, the effective $|\vec{\mu}_i||\vec{\mu}_j|$ are required. Unfortunately, there is a wide range of experimentally and theoretically reported corresponding values for Chls.[3–5] Further, since we model the Q and B bands not via their individual electronic states, but as united entities, the effective $|\vec{\mu}_i|$ must also include that of higher states (Q_x or B_y): Also, the experimentally observed $|\vec{\mu}_i|$ of the donor band does not only result from population absorbed by the lowest state, but from all states in the band, which relax into the donor state prior to EET via IC. Our strategy to obtain the $|\vec{\mu}_i|$ values first requires the experimental approach to compute EET rates[6]

$$k_{FRET,exp} = \frac{9\ln(10)}{128\pi^5 N_A}\kappa^2 \Phi_D \tau_D^{-1} n^{-4} \frac{J}{|r_{ij}|^6} \quad \text{(S1)}$$

Eq. (S1) provides k_{FRET} in units of the reciprocal donor lifetime τ_D^{-1}, further requiring Avogadro's number N_A, the spectral overlap J and the quantum yield Φ_D of the donor. Note that $\Phi_D\tau_D^{-1}$ is identical to the donor fluorescence rate k_f. For comparison to experimental data, we used eq. (S1) to compute empirical $k_f = \Phi_D\tau_D^{-1}$ and k_{FRET} for each Chl pigment pair in our study, using an arbitrary model, which we choose to be r_{ij} = 10 Å, κ^2 = 1, n = 1.4 (Table S1). Since Chl *c1* lifetimes are, to our knowledge, unavailable, we had to compute the corresponding values, taking the respective parameters from the literature.[1,7–10]

Table S1: k_f fluorescence rates (model-independent) and k_{FRET} EET rates (based on an arbitrary model, see text for details) in ns^{-}, computed from eq. (S1) for the Chl-Chl pairs of our study. Spectral overlap J in 10^{14} M^{-1} cm^{-1} nm^4; pairs in italics are not found in vivo. Emp.: Empirical, $= \Phi_D\tau_D^{-1}$, Semi-emp.: semi-empirical, see eq. (4) of previous work,[10] "Used" k_f is empirical value, unless unavailable, then semi-empirical value with an average scaling factor of 0.631, derived from the Chl Q band cases. For τ_D, 100 fs (Chl a) or 58 fs (Chl b) were used.[9]

**Q band**	k_f			$k_{FRET,exp}$, (J), acceptors		
**Donors**	**Emp.**[7]	**Semi-emp.**	**Used**	**Chl *a***	**Chl *b***	**Chl *c1***
**Chl *a***	0.051	0.072	0.051	6433.3 (55.1)	903.2 (7.7)	60.7 (0.52)
**Chl *b***	0.037	0.065	0.037	3473.6 (41.0)	3526.9 (41.7)	*396.2 (4.68)*
**Chl *c1***	n/a	0.041	0.026	1507.5 (25.3)	*1975.1 (33.2)*	800.2 (13.5)

**B band**	k_f			$k_{FRET,exp}$, (J), acceptors		
**Donors**	**Emp.**[11]	**Semi-emp.**	**Used**	**Chl *a***	**Chl *b***	**Chl *c1***
**Chl *a***	< 1.0	1.431	0.903	24752.6 (12.0)	56922.8 (27.6)	87006.1 (42.1)
**Chl *b***	< 1.724	1.002	0.632	2776.5 (1.9)	34648.2 (24.0)	*31958.5 (22.1)*
**Chl *c1***	n/a	1.239	0.782	3524.9 (2.0)	*62357.2 (34.9)*	69711.2 (39.0)

The theoretical analogue to eq. (S1) is provided by Fermi's Golden rule (eq. (3) of the main article). Using the ρ values from Table 1 (after conversion to units of energy^{-1} by multiplying with $(hc)^{-1}$) in the main paper, and the J values from Table S1, we can now compute the strength of the dipole-dipole interaction by combining eqs. (1), (3) and (S1) to

$$|\vec{\mu}_i||\vec{\mu}_j| = \sqrt{k_{FRET,exp}\frac{\hbar |r_{ij}|^6 n^4 8\pi\epsilon_0^2}{\rho\kappa^2}} = \frac{3\epsilon_0}{4\pi^2}\sqrt{\frac{\hbar k_f \ln(10) J}{\rho N_A}} \tag{S2}$$

$$J = \frac{\int_0^\infty a_A(\lambda)F_D(\lambda)\lambda^4 d\lambda}{\int_0^\infty F_D(\lambda)d\lambda} \tag{S2a}$$

with $a_A(\lambda)$ as molar extinction coefficient at wavelength λ of the acceptor, and likewise F_D as the fluorescence of the donor. The resulting values are listed in Table S2 (which do not depend on the arbitrary model parameters, as seen in the second part of eq. (S2)).

Table S2: Effective coupling dipole strengths $|\vec{\mu}_i||\vec{\mu}_j|$ for the Chl-Chl pairs of our study in Debye², and the resulting $|\vec{\mu}_i|$ in Debye, when assuming that emission and absorption have nearly the same $|\vec{\mu}_i|$ ($\approx \sqrt{|\vec{\mu}_i||\vec{\mu}_j|}$, for $i = j$) or are derived from dividing by this value (for $i \neq j$). Note that Chl b-c1 interactions are not corresponding to a known in vivo case.

**Q band**	**Acceptors**			**$\lvert\vec{\mu}_i\rvert$ (same)**
**Donors**	**Chl *a***	**Chl *b***	**Chl *c1***	
**Chl *a***	42.8	38.5	29.5	6.54
**Chl *b***	35.3	31.6	24.1	5.62
**Chl *c1***	29.1	26.1	19.8	4.45
**$\lvert\vec{\mu}_i\rvert$ (mixed)**	6.28 (b), 6.53 (c1)	5.89 (a), 5.85 (c1)	4.53 (a), 4.29 (b)	/

**B band**	**Acceptors**			**$\lvert\vec{\mu}_i\rvert$ (same)**
**Donors**	**Chl *a***	**Chl *b***	**Chl *c1***	
**Chl *a***	167.4	181.3	222.4	12.9
**Chl *b***	145.6	159.6	195.7	12.6
**Chl *c1***	163.6	174.6	214.8	14.7
**$\lvert\vec{\mu}_i\rvert$ (mixed)**	11.3 (b), 13.0 (c1)	14.1 (a), 11.9 (c1)	17.2 (a), 15.5 (b)	/

For the Q band, all retrieved $|\vec{\mu}_i|$ values fall into a very close range, suggesting that a corresponding average (6.45 D, 5.79 D or 4.42 D for *a*, *b* or *c1*, respectively) is trustworthy. As shown in eq. (S2), this should be independent of the refractive index n; yet experimental sources still report a corresponding dependence.[5] This is because our J parameter (due to the non-normalized extinction) still depends on n. Our $|\vec{\mu}_i||\vec{\mu}_j|$ for the Q band of Chls a and b are thus higher than those reported at n = 1.4 previously (30.24 and 20.8 for a and b, respectively).[5] Our computed values are however the values to use in a context of Förster theory, as they are derived directly from the appropriate experimental spectra at n ≈ 1.4. Intriguingly, our computed values coincide well with those reported from multireference calculations (a: 6.6; b: 5.2),[12] further supporting the validity of the $|\mu_i|$ values in Table S2.

For the B band, the range of computed $|\vec{\mu}_i|$ is larger for the individual pigments, and the source of the corresponding uncertainties is not fully clear. The data quality of the input B band emission is, however, much lower than that for the Q bands, with Chl *c1* being a complete approximation (see above). We thus opt to ignore the *c1*-derived B band values for Chls *a* and *b*, resulting in employed $|\vec{\mu}_{i,B}|$ values of 12.6 (*a*) and 13.4 (*b*). This is higher than those reported by multireference calculations (about 50%), but the B band consists of multiple strong absorbing states, hence the effective $|\vec{\mu}_{i,B}|$ must be higher

than the $|\vec{\mu}_i|$ for an individual electronic transition. For Chl *c1*, we chose the value of 16.4, the average of the two *c1* absorption cases, to avoid using the approximated *c1* emission. The employed values are listed below in Table S8.

Dipole moment strengths – Crts

The data situation for the Crts is sparser than that of the Chls, as the measurement of Crt S_2 emission spectra is similarly challenging as measuring Chl B band fluorescence. In contrast to the Chls, however, Crts do not provide an easily accessible lower band signal for comparison. Still, we can apply the same analysis as above to the Crts, also Chls for coupling targets. The corresponding results can be found in Table S3, Table S4 and Table S5.

Table S3: k_f fluorescence rates (model-independent) and k_{FRET} EET rates (based on an arbitrary model, see text for details) in ns^{-1}, computed from eq. (S1) for the Crt-Chl pairs of our study. Spectral overlap J in 10^{14} M^{-1} cm^{-1} nm^4. Emp.: Empirical, $= \Phi_D \tau_D^{-1}$, Semi-emp.: semi-empirical, see eq. (4) of previous work,[10] "Used" k_f is empirical value, unless unavailable, then semi-empirical value with an average scaling factor of 0.98, derived from the β-carotene case. For τ_D, 163 fs was used.[13]

**Q band**		k_f		$k_{FRET,exp}$, (J), acceptors		
**Donors**	**Emp.**[13,14]	**Semi-emp.**	**Used**	**Chl *a***	**Chl *b***	**Chl *c1***
**Violaxanthin**	n/a	0.985	0.965	4703.1 (2.1)	6844.9 (3.1)	5498.0 (2.5)
**Lutein**	n/a	1.101	1.079	7209.1 (2.9)	10591.5 (4.3)	8492.9 (3.4)
**Neoxanthin**	n/a	1.155	1.093	3451.3 (1.4)	4401.6 (1.8)	2726.0 (1.1)
**β-carotene**	0.920	1.005	0.920	7430.8 (3.7)	11598.9 (5.5)	11704.1 (5.6)
**Fucoxanthin**	n/a	1.074	1.052	3321.8 (1.4)	4236.5 (1.8)	2623.7 (1.1)
**Diadinoxanthin**	n/a	1.105	1.083	3419.7 (1.4)	4361.3 (1.8)	2701.0 (1.1)

**B band**		k_f		$k_{FRET,exp}$, (J), acceptors		
**Donors**	**Emp.**[13,14]	**Semi-emp.**	**Used**	**Chl *a***	**Chl *b***	**Chl *c1***
**Violaxanthin**	n/a	0.985	0.965	< 1.0 (< 0.1)	3532.8 (1.6)	1550.0 (0.7)
**Lutein**	n/a	1.101	1.079	< 1.0 (< 0.1)	2567.6 (1.0)	1167.8 (0.5)
**Neoxanthin**	n/a	1.155	1.093	0.0 (0.0)	4476.6 (1.8)	2145.8 (1.1)
**β-carotene**	0.920	1.005	0.920	0.0 (0.0)	423.1 (0.2)	227.4 (0.1)
**Fucoxanthin**	n/a	1.074	1.052	0.0 (0.0)	4308.7 (1.8)	2065.3 (1.1)
**Diadinoxanthin**	n/a	1.105	1.083	0.0 (0.0)	4435.7 (1.8)	2126.1 (1.1)

Table S4: k_{FRET} EET rates (based on an arbitrary model, see text for details) in ns^{-}, computed from eq. (S1) for the Chl-Crt pairs of our study. Spectral overlap J in 10^{14} M^{-1} cm^{-1} nm^4.

**Q band**	$k_{FRET,exp}$, (J), acceptors					
**Donors**	**Vio**	**Lut**	**Neo**	**Bcr**	**Fuc**	**Dia**
**Chl *a***	0.0 (0.0)	0.0 (0.0)	0.0 (0.0)	85.8 (0.7)	0.0 (0.0)	0.0 (0.0)
**Chl *b***	< 1.0 (< 0.1)	< 1.0 (< 0.1)	< 1.0 (< 0.1)	110.9 (1.3)	< 1.0 (< 0.1)	< 1.0 (< 0.1)
**Chl *c1***	< 1.0 (< 0.1)	< 1.0 (< 0.1)	0.0 (0.0)	82.7 (1.4)	0.0 (0.0)	0.0 (0.0)

**B band**	$k_{FRET,exp}$, (J), acceptors					
**Donors**	**Vio**	**Lut**	**Neo**	**Bcr**	**Fuc**	**Dia**
**Chl *a***	83349.0 (40.3)	93741.8 (45.4)	81510.1 (39.5)	88411.1 (42.8)	74216.6 (35.9)	82522.5 (39.9)
**Chl *b***	54343.9 (37.6)	68052.8 (47.1)	48950.0 (33.9)	66693.4 (46.1)	55963.5 (38.7)	59058.1 (40.8)
**Chl *c1***	79051.3 (44.2)	97803.2 (54.7)	72592.0 (40.6)	94153.0 (52.6)	76689.5 (42.9)	85296.0 (47.7)

Table S5: k_{FRET} EET rates (based on an arbitrary model, see text for details) in ns-, computed from eq. (S1) for the Crt-Crt pairs of our study. Spectral overlap J in 10^{14} M^{-1} cm^{-1} nm^4.

	$k_{FRET,exp}$, (J), acceptors					
**Donors**	**Vio**	**Lut**	**Neo**	**Bcr**	**Fuc**	**Dia**
**Violaxanthin**	23603.8 (10.7)	38949.6 (17.6)	18790.3 (8.5)	43939.7 (19.9)	41223.8 (18.7)	33539.9 (15.2)
**Lutein**	17208.0 (7.0)	31947.2 (12.9)	13924.4 (5.6)	38440.3 (15.6)	36786.2 (14.9)	27750.1 (11.2)
**Neoxanthin**	35189.7 (14.1)	65523.7 (26.2)	27209.8 (10.9)	72876.3 (29.1)	63497.9 (25.4)	57145.6 (22.9)
**β-carotene**	3599.7 (1.7)	9241.2 (4.4)	3283.9 (1.6)	13830.2 (6.6)	15135.4 (7.2)	8104.5 (3.9)
**Fucoxanthin**	33867.8 (14.1)	63065.8 (26.2)	26189.1 (10.9)	70142.6 (29.1)	61116.0 (25.4)	55002.0 (22.9)
**Diadinoxanthin**	34865.8 (14.1)	64924.2 (26.2)	26960.9 (10.9)	72209.6 (29.1)	62917.0 (25.4)	56622.8 (22.9)

Applying eq. (S2) to Table S5 the yields the corresponding $|\vec{\mu}_i||\vec{\mu}_j|$ values, Table S6.

Table S6: Effective coupling dipole strengths $|\vec{\mu}_i||\vec{\mu}_j|$ for the Crt-Crt pairs of our study in Debye², and the resulting $|\vec{\mu}_i|$ in Debye, when assuming that emission and absorption have nearly the same $|\vec{\mu}_i|$ ($\approx \sqrt{|\vec{\mu}_i||\vec{\mu}_j|}$, for $i = j$) or are derived from dividing by this value (for $i \neq j$).

	Acceptors						$|\vec{\mu}_i|$ (same)
**Donors**	**Vio**	**Lut**	**Neo**	**Bcr**	**Fuc**	**Dia**	
**Violaxanthin**	279.38	305.34	292.63	308.21	288.44	288.26	16.71
**Lutein**	298.75	326.81	313.38	329.99	309.12	308.52	18.08
**Neoxanthin**	294.89	321.58	308.62	323.70	302.57	303.59	17.57
**β-carotene**	285.33	311.52	302.30	315.11	295.55	293.66	17.75
**Fucoxanthin**	289.30	315.49	302.78	317.57	296.84	297.84	17.23
**Diadinoxanthin**	293.53	320.10	307.20	322.22	301.18	302.20	17.38
**Avg. $|\vec{\mu}_i| \pm \Delta|\vec{\mu}_i|$**	16.63 ±0.22	18.15 ±0.23	17.45 ±0.18	18.31 ±0.20	17.13 ±0.17	17.13 ±0.22	

The resulting values already fall into a very close range for each pigment, although for β-carotene, the range as acceptor is slightly higher than that resulting from only self-interaction. The last row of Table S6 displays the corresponding averages and their standard deviation, which is low, so we used the averages for our models, as listed in Table S7.

Final list of parameters

This section contains all general data to quickly reconstruct our FRET model, starting with the spectroscopic parameters derived from the previous paragraphs. Analogous to our previous[12] analysis on the $\vec{\mu}_i$ orientations, we defined $\vec{\mu}_i$ to be oriented along the axis formed by to atoms of the compound structure. Table S7 provides the labels of these atoms according to the experimental structure. Only the lowest state within each band was used for this study, thereby biasing against a strong coupling due to κ being more restricted than in reality.

Table S7: Orientation axes for $\vec{\mu}_i$ associated either to the Q_y state of each pigment (representing the Q band as a whole) or the B_x/S_2 states (with B_x representing the B band as a whole). The axes are formed by the two atoms given. Orientations chosen to mimic earlier orientations found for CAM-B3LYP/6-31G calculations of the pigments, in an acetone continuum model.*[12]

**Pigment**	**Q**				**B (Chls) or S_2 (Crts)**			
	**Atom 1**	**Atom 2**	**ΔE / eV**	$\lvert\vec{\mu}_i\rvert$**/Debye**	**Atom 1**	**Atom 2**	**ΔE / eV**	$\lvert\vec{\mu}_i\rvert$**/Debye**
Chl *a*	NB	ND	1.878	6.45	C4A	C4C	2.892	12.6
Chl *b*	NB	ND	1.928	5.79	C4A	C4C	2.737	13.4
Chl *c1*	CHB	CHD	1.975	4.42	C4C	CHB	2.782	16.6
Violaxanthin	See S_2	See S_2	1.835	See S_2	C12	C32	2.635	16.6
Lutein	See S_2	See S_2	1.816	See S_2	C12	C32	2.616	18.2
Neoxanthin	See S_2	See S_2	2.018	See S_2	C12	C32	2.818	17.5
β-carotene	See S_2	See S_2	1.795	See S_2	C12	C19	2.595	18.3
Fucoxanthin	See S_2	See S_2	1.852	See S_2	C8	C24	2.652	17.1
Diadinoxanthin	See S_2	See S_2	1.802	See S_2	C8	C24	2.602	17.1

Table S7 provides the excitation energies ΔE used for each band before shifting; shown values (plus shifts from Table S8, see sec. 4 below) are the diagonal elements of the Hamiltonians $\mathbf{H_Q}$ and $\mathbf{H_B}$ of the main paper. It also lists the $|\vec{\mu}_i|$ used in our Förster model for the calculation of the coupling elements, as derived in the previous parts of this section. The resulting order of dipole strengths reflects the expectation from the experimental data (Chl *c1*(Q) < Chl *b*(Q) < Chl *a*(Q) < Chl *a*(B) < Chl *b*(B) < Chl *c1*(B) < Crts).

The structural parameters (i.e., the positions of the atoms listed in Table S7) were read for each pigment of the following PDB entries: Entry 5XNL,[15] chains "4" (for CP24), "s" (for CP26), "R" (for CP29) and the trimer of chains "1", "2" and "3" (for LHCII); and entry 7VD5,[16] the tetramer of chains "y", "z", "0" and "1" (for tFCP). The distance vectors between the pigments $|r_{ij}|$ were computed as the vector between the Mg ions (for Chls) or the center of mass of the conjugated carbon atoms (for Crts).

4. Details on site energy calculations and sources

For site energies (Table S8), we relied mostly on our earlier work based hybrid quantum mechanics/molecular mechanics (QM/MM): Chl site energies for Q and B band states were obtained from our earlier work for the CP29 complex.[17] For CP24 and CP26, the shifts were assigned in analogy to CP29, using the site shifts from CP29 for the corresponding sites in the smaller LHCs.

For LHCII, the site shifts were computed using the same protocol: After repairing and embedding the experimentally determined LHCII trimer structure (PDB entry 5XNL,[15] chains 1-3) in a DOPC membrane,

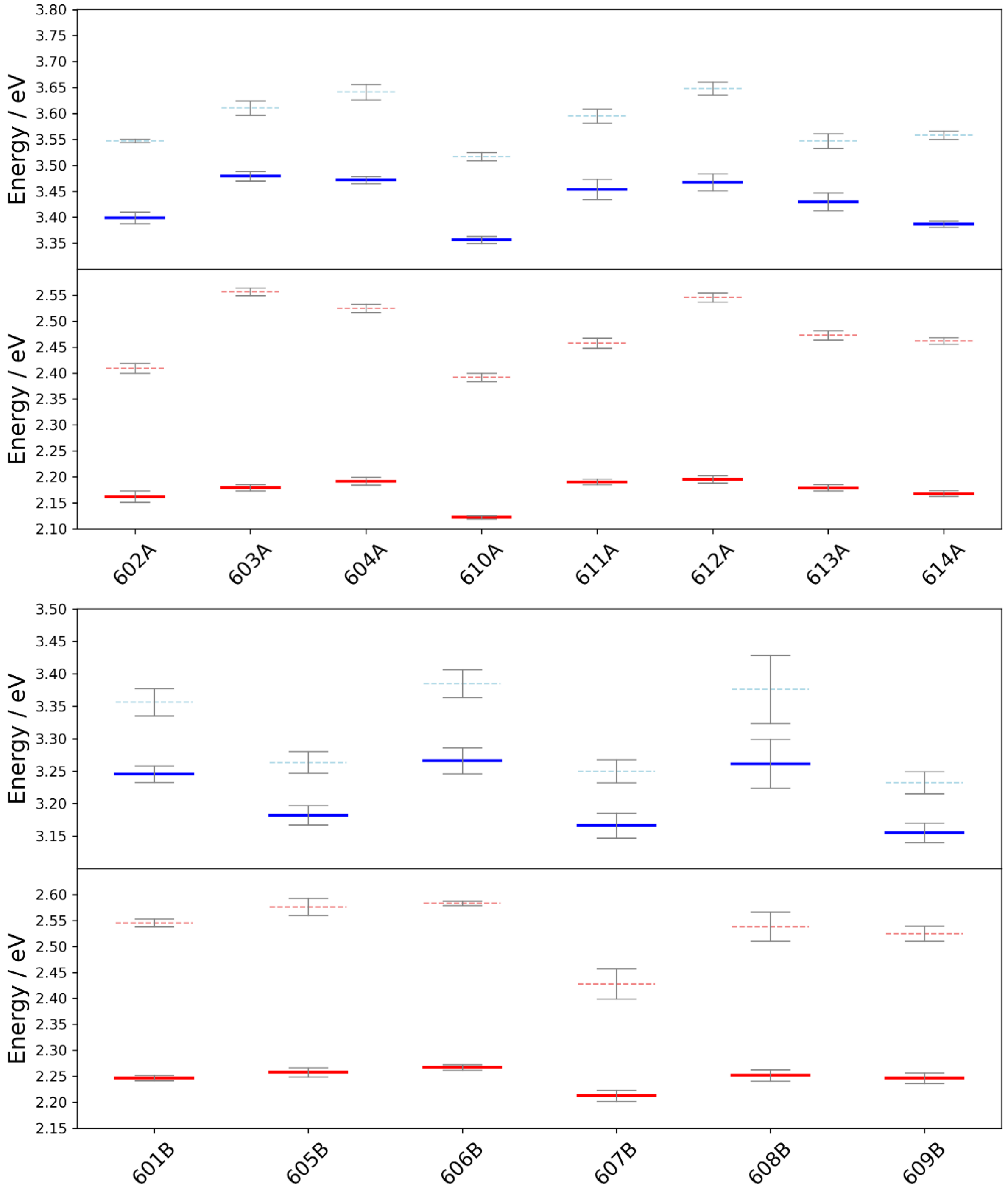

Figure S3: QM/MM vertical excitation energies of LHCII (time-dependent CAM-B3LYP/6-31G). Shown are averages (colored bars, Q band red, B band blue) over the depicted Chl pigments with standard errors of the mean indicated as horizontal bars. Dotted horizontal bars are states that are not used in our models (Q_x and B_y states) but were also computed as results of the calculations. Top graph shows the energies of Chl a pigments, bottom graph of Chl b.*

a 100 ps NVT (2 fs timestep for this and all other steps, modified Berendsen thermostat) was performed using the Gromacs 2019 software,[18] followed by a 1 ns NPT, followed by a 120 ns dynamics simulation (NPT equilibration and dynamics using the leap-frog integrator, Parinello-Rahman pressure coupling using a reference pressure of 1.0 bar in x, y and z dimension and a time constant of 5 ps, Nose-Hoover thermostat with T = 300 K and a time constant of 0.5 ps). The site energies were calculated as the statistical average of a total of 8 frames (every 10 ns starting at 50 ns up to 120 ns) of the individual chromophores of one LHCII monomer. The data were calculated equivalent to the CP29-related articles,[4,17] where first each chromophore was optimized using QM/MM[19] and then the excited state properties were determined by a time-dependent density functional theory calculation[20–22] (functional/basis: CAM-B3LYP/6-31G*) embedded in the point charge field of the QM/MM model.[23,24] Quantum mechanical calculations were performed using the Gaussian16 software.[25] The corresponding results are shown in Figure S3.

For tFCP, we used the recently published site energies by Maity and coworkers as basis.[26] As they restricted their analysis to the Q band, we used the same shifts for both bands. Shifts were computed by averaging over the provided site energies and then using the deviation from the average energy as the shift values. The tFCP contains two Chl sites for which the earlier work does not provide corresponding values; those sites were not considered in the model. The detailed shifts are listed in Table S8.

Table S8: Site energy shifts applied to each pigment in the model systems. Minor LHC shifts in accordance to CP29, from earlier work.[17] LHCII shifts computed analogously to CP29. The tFCP shifts obtained from the work of Maity and coworkers,[26] by averaging over their absolute energies and taking the deviation of the average for the listed shift. The tFCP structure is not as systematically labeled as the LHC cases, therefore site numbers may differ for each chain and are thus listed in detail.

	Minor LHCs		LHCII (monomer)		tFCP monomers	
**LHC site**	**ΔE_Q / eV**	**ΔE_B / eV**	**ΔE_Q / eV**	**ΔE_B / eV**	**FCP Site (chain)**	**ΔE / eV**
601	-0.051	-0.076	0.000	0.063	202 (1), 301 (y, z), 302 (0)	0.021
602	-0.011	-0.032	-0.011	-0.095	203 (1), 302 (y, z), 303 (0)	0.000
603	0.001	0.022	0.006	0.091	204 (1), 303 (y, z), 304 (0)	-0.024
604	0.007	-0.001	0.018	0.082	205 (1), 304 (y, z), 305 (0)	0.004
605 (LHCII)	n/a	n/a	0.011	-0.040	207 (1), 305 (z), 306 (0, y)	-0.006
606	0.017	0.040	-0.020	0.092	208 (1), 306 (z), 307 (0, y)	-0.012
607	-0.008	0.055	-0.035	-0.089	201 (1), 209 (1), 307 (z), 308 (y)	0.027
608	0.008	-0.054	0.005	0.035	210 (1), 308 (0, z), 309 (y)	0.000
609	0.021	0.019	0.000	-0.061	211 (1), 309 (0, z), 310 (y)	-0.012
610	0.003	-0.004	-0.051	-0.070		
611	-0.024	-0.065	0.017	-0.065		
612	-0.018	-0.015	0.022	0.109		
613	-0.001	0.032	0.006	-0.089		
614	-0.017	-0.043	-0.006	0.039		
616 (CP29)	0.040	0.093	n/a	n/a		

5. Relative absorption, details on Figure 7 and Zn-porphyrin coupling elements

Relative absorption

The Lambert-Beer[27,28] law was used to assess the relative absorption of the investigated model pigment mixtures

$$\ln\left(\frac{I_0(\lambda)}{I(\lambda)}\right) = d \sum_{i}^{Chromophores} \sigma_i(\lambda) n_i \quad \text{(S3)}$$

Here, $I_0(\lambda)$ and $I(\lambda)$ are the initially irradiated or transmitted number of photons for wavelength λ, d is the pathlength through the medium (see below, Table S9), and with σ_i being the pigment's absorption cross section at a given λ (derived from experimental molar extinction coefficients, ϵ, as $\sigma(\lambda) = \ln(10)\epsilon(\lambda)/N_A$) and n_i the number of pigments in the model. n_i is here always equal to 1, as each pigment was modelled individually: Each site was shifted by its site energy, see Table S8, making pigment different in our models. Chl *a*, Chl *b* and Crt spectra employed here are identical to earlier work for consistency, differing only slightly from those depicted in Figure 2 of the main paper (in acetone vs. in diethyl ether).[1,10] The employed Chl c1 spectrum is as shown in Figure 2 of the main paper,[29–31] spectra of fucoxanthin (in CS_2,[32] extinction from spectrum in acetone[33]) and diadinoxanthin (in ethanol,[34] extinction from spectrum in methanol[35]) were obtained from the given sources.

To obtain a path length d for a structurally oriented system like an LHC, one would have to know the orientation of the light beam with respect to the LHC target complex. As this is also unknown in (most) experiments, we can approximate and still stay true to the experimental situation. This further has the advantage that the orientation of the $\vec{\mu}$ towards the irradiated field becomes random as well. Consequently, the path length was approximated by taking an estimated volume of the target LHC and considering the random rotation by transforming the volume into an equally large sphere. The sphere diameters are given in Table S9, and are also the path lengths used for eq. (S4). The absorbed photons were then computed via

$$\Delta I(\lambda) = I_0(\lambda) * (1 - e^{-6/(\pi d^2)\sum \sigma_i(\lambda)}) \quad \text{(S4)}$$

Individual pigment contributions to the sum of absorbed intensities are then computed by linearly decomposing $\Delta I(\lambda)$ according to the weight of each pigment's σ_i to the total sum.

Table S9: Total absorbed photons of various LHC variants from eq. (2), summed over the range of 350 to 800 nm or to 500 nm (Soret band) under irradiation with sunlight at sea level (0 m), 21 June noon, 50° N latitude.[36] Values shown are relative to the LHCII case, full spectral range. For tFCP, the irradiation at 100 m below sea level was also tested (see also Figure S4).[37] Values relative to LHCII absorbance over the 350 to 800 nm range. The configurations replchla and onlya also include Chls a at binding sites originally filled with Chls b/c.

**Complex**	**d/ Å**	**Σ ΔI: 350-800 nm (350-550 nm), normalized to LHCII 350-800 nm**			
		**WT**	**replchla**	**noCrts**	**onlya**
CP24	50	0.76 (0.57)	0.72 (0.50)	0.55 (0.35)	0.50 (0.28)
CP26	50	0.84 (0.60)	0.81 (0.55)	0.63 (0.39)	0.59 (0.33)
CP29	50	0.87 (0.61)	0.83 (0.56)	0.67 (0.41)	0.63 (0.35)
LHCII	85	1.00 (0.74)	0.95 (0.66)	0.71 (0.46)	0.66 (0.37)
tFCP (0 m)	95	0.94 (0.79)	0.87 (0.67)	0.52 (0.37)	0.45 (0.25)
tFCP (-100 m)	95	8.3e-8 (8.1e-8)	7.6e-8 (7.4e-8)	1.7e-8 (1.5e-8)	1.0e-8 (0.9e-8)

Calculation of data in Figure 7

The contributions *c* in Figure 7 of the main manuscript were calculated according to eqs. (S5)-(S8) from the data in Table S9.

$$c_{\mathrm{Chl\ b,c}} = \frac{1}{\sum^{All} \Delta I} \left(\sum^{\mathrm{All}} \Delta I - \sum^{\substack{\mathrm{Chl\ a^*,} \\ \mathrm{Crts}}} \Delta I \right) * 100 \tag{S5}$$

$$c_{\mathrm{Chls\ b,c\ w/o\ Crts.}} = \frac{1}{\sum^{All} \Delta I} \left(\sum^{\substack{\mathrm{Chl\ a,} \\ \mathrm{Chls\ b/c}}} \Delta I - \sum^{\mathrm{Chl\ a^*}} \Delta I \right) * 100 \tag{S6}$$

$$c_{\mathrm{Crts.}} = \frac{1}{\sum^{All} \Delta I} \left(\sum^{All} \Delta I - \sum^{\substack{\mathrm{Chl\ a,} \\ \mathrm{Chls\ b/c}}} \Delta I \right) * 100 \tag{S7}$$

$$c_{\mathrm{Chl\ a}} = 100 - c_{\mathrm{Chl\ b,c}} - c_{\mathrm{Crts.}} \tag{S8}$$

Zn-porphyrin coupling elements

The compounds Nakano and coworkers labeled **5** and **6** in their work[38] are of most relevance for comparison here, as **5** shows only weak excitonic coupling between the Zn-porphyrin B states, while **6** shows an intensity splitting between the two experimental B band peaks. Both **5** and **6** are linear, yet orthogonal, Zn-porphyrin dimers.

When using eq. (1) of the main paper to obtain the coupling element $\mathrm{V_{ij}}$, we require the distance between the Zn-porphyrin centers r_{ab}, the length of the transition dipole moments $|\vec{\mu}|_a$ = $|\vec{\mu}|_b$ and the orientation factor κ_{ab}. From the original work, we know that the orientation of the two $\vec{\mu}$ of interest is parallel, hence $|\kappa_{ab}|$ = 2. r_{ab} can be reasonably estimated to be a porphyrin diameter (as is two times half a diameter) plus the length of the linker connecting the two units; taking these values from a structure like 5XNL[15] results in 6.7 Å (lower bound, from Chl *a*) plus 2.5 Å (lower bound, from neoxanthin) to a total of 9.2 Å. These parameters are identical for **5** and **6**.

For $|\vec{\mu}|_{a/b}$, we need to compare the values of the reported spectra to those of Chls and the computed values in Table S7. For **5**, the lowest B band molar extinction is reported with a maximum of about 0.21 10^6 M^{-1} cm^{-1}. For **6**, it is not as simple, as the measured intensities are likely affected by the excitonic splitting, and not the "pure" $|\vec{\mu}|_{a/b}$ absorption. We can thus only estimate the "pure" $|\vec{\mu}|_{a/b}$ absorption for **6** as the average of both B band peaks, which is about 0.32 10^6 M^{-1} cm^{-1}. Though converting extinction into transition dipole moments is possible,[39] it seems more prudent to compare to the spectra we have already used in our models. It can be seen in Figure 2 of the main paper that the intensity of **5** corresponds well to that of Chl c-type pigments, hence we use the corresponding μ of Table S7 (16.6 D), scaling this value by 1.5 for compound **6**. The resulting $\mathrm{V_{ij}}$ values for the pair of Zn-porphyrins in the experiments by Nakano and coworkers are thus about 909 cm^{-1} for **5** and 2045 cm^{-1} for **6**, indicating a coupling far beyond the weak regime, leading to the observed anti-Kasha effects.

In realistic networks of pigments, like those discussed in the main paper, these ideal values are clearly unachievable for most pairs in the network, foremost because not all pigments are less than 10 Å apart from each other. We therefore used the weaker coupled case (compound **5**) to mimic a corresponding CP24 network, with the μ orientations and pigment distances as found for CP24. The resulting average coupling for this Zn-porphyrin CP24-like network (11 Zn-porphyrins replacing all Chls, no other pigments) is 216 cm^{-1}. This value is about twice as high as the computed B-B Chl *a*-Chl *a* interactions, and nearly identical to the B-B interactions between Chls *b*/*c* (Figure 3 of the main paper). This would indicate that at least Chls *b*/*c* have strong potential for anti-Kasha behavior.

6. Testing the Lambert-Beer model parameters

Table S10 shows the change in absorption with different numbers of LHCII in a constant volume (1145 Å diameter sphere, approximately corresponding to the volume of a cylinder of 100 nm height, 100 nm diameter), normalized to the absorption of the lowest concentration (one LHCII trimer). It can be seen that the results are nearly independent of LHCII concentration, indicating that the relative absorption shown in the main article is nearly unaffected by packing effects. Table S11 indicates that the pigment concentration in the LHCII sphere is about half the size of the respective concentrations of the minor LHC cases, which is alleviated by d being about twice as long, making the LHCII and minor LHC cases comparable again; the same logic applies to the tFCP case.

Table S10: Relative absorbance of a 1145 Å diameter sphere containing the pigments of N_{LHCII} LHCII variants, summed over the range of 350 to 550 nm (or 350 to 800 nm in parentheses) under irradiation with sunlight at sea level, 21 June noon, 50° N latitude.[36] Values shown per LHCII complex and normalized to N_{LHCII} = 1 absorbance over 350 to 800 nm range. Chl a also includes Chls a at binding sites originally filled with Chls b/c.*

N_{LHCII}	Σ $\Delta I/N_{LHCII}$: 350-800 nm (350-550 nm)			
	All pigments	Chl a*, Crts	Chl a, Chls b/c	Chl a*
1	1.00 (0.74)	0.95 (0.66)	0.71 (0.45)	0.66 (0.37)
2	>0.99 (0.74)	0.95 (0.66)	0.71 (0.45)	0.66 (0.37)
4	>0.99 (0.74)	0.95 (0.66)	0.71 (0.45)	0.66 (0.37)
8	>0.99 (0.74)	0.95 (0.66)	0.71 (0.45)	0.66 (0.37)
16	>0.99 (0.74)	0.95 (0.66)	0.71 (0.45)	0.66 (0.37)
32	>0.99 (0.74)	0.94 (0.66)	0.71 (0.45)	0.66 (0.37)
64	>0.99 (0.74)	0.94 (0.65)	0.71 (0.45)	0.66 (0.37)
128	>0.99 (0.73)	0.94 (0.65)	0.71 (0.45)	0.65 (0.37)
256	0.98 (0.73)	0.93 (0.64)	0.70 (0.45)	0.65 (0.36)
512	0.97 (0.71)	0.91 (0.63)	0.69 (0.44)	0.64 (0.36)

Table S11: Absorption model parameters, comparing the resulting concentrations of the different present pigment classes to the volumes and path lengths.

Complex	d/ Å	Volume / nm^3	$c_{Chl\ a}$ / µm^{-3}	$c_{Chls\ b/c}$ / µm^{-3}	c_{Crts} / µm^{-3}
CP24	50	65.45	91.67	76.36	45.84
CP26	50	65.45	137.51	61.12	45.84
CP29	50	65.45	152.79	61.12	45.84
LHCII	85	321.56	75.64	55.98	37.32
tFCP	95	448.92	51.23	33.41	51.23

7. Difference spectra for LHCII and tFCP

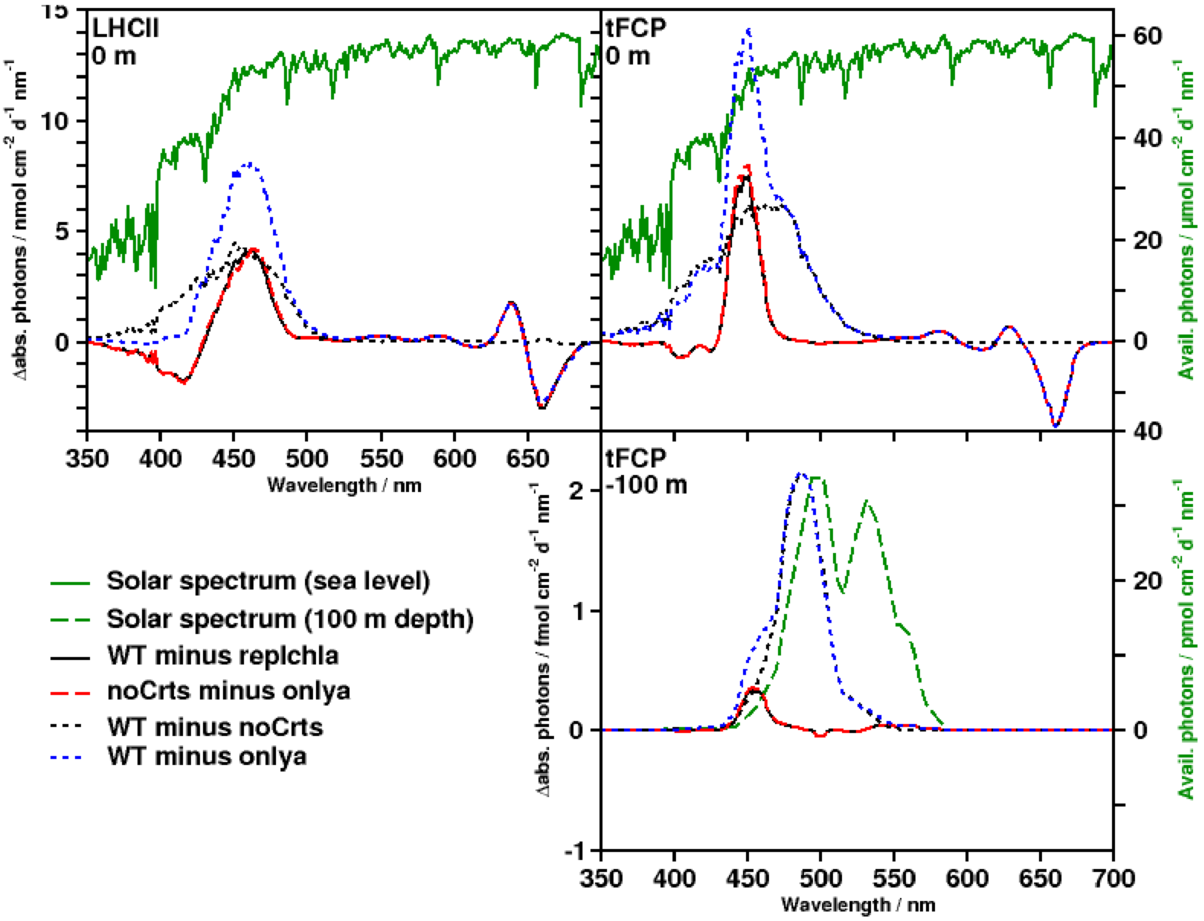

Figure S4: Difference in photon absorption of LHCII or tFCP variants under irradiation with sunlight at sea level, 21 June noon, 50° N latitude[36] or 100 m oceanic depth (at noon, undisclosed day, eastern mediterranean).[37] Models based on a spherical N = 512 system with 1145 Å diameter and path length (see Table S10 for details on the LHCII system; analogous setup for tFCP). The configurations replchla and onlya also include Chls a at binding sites originally filled with Chls b/c.

The spectra shown in Figure S4 indicate solar irradiation spectra (green lines) together with the differences in absorbed photons upon variation of the pigment composition for LHCII and tFCP. The upper left plot, LHCII at sea level, indicates that the increases in Soret and Q band absorption facilitated by Chl *b* are accompanied by losses in absorbance in the higher energy region of the Soret band (350-425 nm) as well as the lower energy region of the Q band (650-680 nm). This does not depend on the presence of the Crts, which was already shown by Table S9, as the difference peak at about 450 nm is only minutely smaller with Crts present ("WT minus replchla" vs. "noCrts minus onlya"). While simply adding Chl *b* would likely be beneficial for overall absorbance, exchanging Chl *a* for Chl *b* results in a pattern of gains and losses which, summed up, lead to the minor absorption improvements found above. For marine organisms, however, this might be different, as the higher penetration depth of UV/blue light results in a drastic change of the available photons.[37] For tFCP, in combination with different sets of irradiation spectra, the results are shown on the right side of Figure S4. At sea level (upper right graph), the pattern is similar to the LHCII case, though the peak resulting from including Chl *c1* is larger than the analogous Chl *b* peak of the LHCII case. Due to the sharper absorbance profile of Chl *c1*, the loss in the upper UV/vis region is also smaller in tFCP. The absorption loss in the Q band region at about 660 nm is however more pronounced, with much smaller gains due to the minute Chl *c1* Q band absorption as compared to Chl *a*.

Turning to the absorption profile at -100 m, for which Chl *c1* could possibly improve absorbance more significantly due to the prevalence of blue light, the observed impact is almost negligible (Figure S4, lower right plot). Indeed, the key players for absorbance here are the Crts (fucoxanthin and diadinoxanthin), not the Chls. Chl *c1* is only responsible for a small shoulder at about 450 nm in the overall absorption difference profile. There are simply not enough photons in the sub-470 nm region available at larger depths, and the graph clearly shows that the Crts become the main absorbers.

8. Eigenvectors and eigenvalues (excitons) of all investigated complexes

The figures analogous to Figures 4 and 6 of the main manuscript for other systems and excitons are available as a separate archive file of the supporting information.

9. References

1. Götze, J. P. & Lokstein, H. Excitation Energy Transfer between Higher Excited States of Photosynthetic Pigments: 2. Chlorophyll b is a B Band Excitation Trap. *ACS Omega* **8**, 40015–40023 (2023).
2. Frank, H. A. *et al.* Spectroscopic and Photochemical Properties of Open-Chain Carotenoids. *J. Phys. Chem. B* **106**, 2083–2092 (2002).
3. Reiter, S., Kiss, F. L., Hauer, J. & de Vivie-Riedle, R. Thermal site energy fluctuations in photosystem I: new insights from MD/QM/MM calculations. *Chem. Sci.* **14**, 3117–3131 (2023).
4. Petry, S., Tremblay, J. C. & Götze, J. P. Impact of Structure, Coupling Scheme, and State of Interest on the Energy Transfer in CP29. *J. Phys. Chem. B* **127**, 7207–7219 (2023).
5. Knox, R. S. & Spring, B. Q. Dipole Strengths in the Chlorophylls. *Photochem. Photobiol.* **77**, 497 (2003).
6. Qi, Q., Taniguchi, M. & Lindsey, J. S. Heuristics from Modeling of Spectral Overlap in Förster Resonance Energy Transfer (FRET). *J. Chem. Inf. Model.* **59**, 652–667 (2019).
7. Niedzwiedzki, D. M. & Blankenship, R. E. Singlet and triplet excited state properties of natural chlorophylls and bacteriochlorophylls. *Photosynth. Res.* **106**, 227–238 (2010).
8. Götze, J. P. & Lokstein, H. Correction to "Excitation Energy Transfer between Higher Excited States of Photosynthetic Pigments: 1. Carotenoids Intercept and Remove B Band Excitations". *ACS Omega* **9**, 22506–22507 (2024).
9. Bricker, W. P. *et al.* Non-radiative relaxation of photoexcited chlorophylls: theoretical and experimental study. *Sci. Rep.* **5**, 13625 (2015).
10. Götze, J. P. & Lokstein, H. Excitation Energy Transfer between Higher Excited States of Photosynthetic Pigments: 1. Carotenoids Intercept and Remove B Band Excitations. *ACS Omega* **8**, 40005–40014 (2023).
11. Leupold, D. *et al.* Two-Photon Excited Fluorescence from Higher Electronic States of Chlorophylls in Photosynthetic Antenna Complexes: A New Approach to Detect Strong Excitonic Chlorophyll a/b Coupling. *Biophys. J.* **82**, 1580–1585 (2002).
12. Götze, J. P., Anders, F., Petry, S., Witte, J. F. & Lokstein, H. Spectral characterization of the main pigments in the plant photosynthetic apparatus by theory and experiment. *Chem. Phys.* **559**, 111517 (2022).
13. Macpherson, A. N. & Gillbro, T. Solvent Dependence of the Ultrafast S 2 −S 1 Internal Conversion Rate of β-Carotene. *J. Phys. Chem. A* **102**, 5049–5058 (1998).
14. Polívka, T. & Sundström, V. Ultrafast Dynamics of Carotenoid Excited States−From Solution to Natural and Artificial Systems. *Chem. Rev.* **104**, 2021–2072 (2004).
15. Su, X. *et al.* Structure and assembly mechanism of plant C 2 S 2 M 2 -type PSII-LHCII supercomplex. *Science* **357**, 815–820 (2017).
16. Nagao, R. *et al.* Structural basis for different types of hetero-tetrameric light-harvesting complexes in a diatom PSII-FCPII supercomplex. *Nat. Commun.* **13**, 1764 (2022).
17. Petry, S. & Götze, J. P. Effect of protein matrix on CP29 spectra and energy transfer pathways. *Biochim. Biophys. Acta - Bioenerg.* **1863**, 148521 (2022).
18. Abraham, M. J. *et al.* GROMACS: High performance molecular simulations through multi-level

parallelism from laptops to supercomputers. *SoftwareX* **1–2**, 19–25 (2015).

19. Götze, J. P. *et al.* A user-friendly, Python-based quantum mechanics/Gromacs interface: gmx2qmmm. *Int. J. Quantum Chem.* **123**, e26486 (2021).

20. Jamorski, C., Casida, M. E. & Salahub, D. R. Dynamic polarizabilities and excitation spectra from a molecular implementation of time-dependent density-functional response theory: N 2 as a case study. *J. Chem. Phys.* **104**, 5134–5147 (1996).

21. Casida, M. E. & Salahub, D. R. Asymptotic correction approach to improving approximate exchange–correlation potentials: Time-dependent density-functional theory calculations of molecular excitation spectra. *J. Chem. Phys.* **113**, 8918–8935 (2000).

22. Casida, M. E., Jamorski, C., Casida, K. C. & Salahub, D. R. Molecular excitation energies to high-lying bound states from time-dependent density-functional response theory: Characterization and correction of the time-dependent local density approximation ionization threshold. *J. Chem. Phys.* **108**, 4439–4449 (1998).

23. Yanai, T., Tew, D. P. & Handy, N. C. A new hybrid exchange–correlation functional using the Coulomb-attenuating method (CAM-B3LYP). *Chem. Phys. Lett.* **393**, 51–57 (2004).

24. Hariharan, P. C. & Pople, J. A. The influence of polarization functions on molecular orbital hydrogenation energies. *Theor. Chim. Acta* **28**, 213–222 (1973).

25. Frisch, M. J. *et al.* Gaussian16 Revision A.03. (2016).

26. Maity, S., Daskalakis, V., Jansen, T. L. C. & Kleinekathöfer, U. Electric Field Susceptibility of Chlorophyll c Leads to Unexpected Excitation Dynamics in the Major Light-Harvesting Complex of Diatoms. *J. Phys. Chem. Lett.* **15**, 2499–2510 (2024).

27. Lambert, J. H. *Photometria sive de mensura et gradibus luminis, colorum et umbrae*. (Eberhard Klett, 1760).

28. Beer, A. Bestimmung der Absorption des rothen Lichts in farbigen Flüssigkeiten. *Ann. der Phys. und Chemie* **162**, 78–88 (1852).

29. Jeffrey, S. W. Preparation and some properties of crystalline chlorophyll c1 and c2 from marine algae. *Biochim. Biophys. Acta - Gen. Subj.* **279**, 15–33 (1972).

30. Jeffrey, S. W., Mantoura, R. F. C. & Bjørnland, T. Data for the identification of 47 key phytoplankton pigments. in *Phytoplankton Pigments in Oceanography: Guidelines to Modern Methods* (eds. Jeffrey, S. W., Mantoura, R. F. C. & Bjørnland, T.) 449–559 (UNESCO Publishing, 1997).

31. Jeffrey, S. W. & Wright, S. W. A new spectrally distinct component in preparations of chlorophyll c from the micro-alga Emiliania huxleyi (Prymnesiophycease). *Biochim. Biophys. Acta - Bioenerg.* **894**, 180–188 (1987).

32. Katoh, T., Nagashima, U. & Mimuro, M. Fluorescence properties of the allenic carotenoid fucoxanthin: Implication for energy transfer in photosynthetic pigment systems. *Photosynth. Res.* **27**, 221–226 (1991).

33. André Haugan, J. & Liaaen-Jensen, S. Improved isolation procedure for fucoxanthin. *Phytochemistry* **28**, 2797–2798 (1989).

34. Kagatani, K. *et al.* Excitation relaxation dynamics of carotenoids constituting the diadinoxanthin cycle. *Photosynth. Res.* **154**, 13–19 (2022).

35. Johansen, J. E., Svec, W. A., Liaaen-Jensen, S. & Haxo, F. T. Carotenoids of the dinophyceae.

Phytochemistry **13**, 2261–2271 (1974).

36. Apell, J. N. & McNeill, K. Updated and validated solar irradiance reference spectra for estimating environmental photodegradation rates. *Environ. Sci. Process. Impacts* **21**, 427–437 (2019).

37. Jaubert, M., Bouly, J.-P., Ribera d'Alcalà, M. & Falciatore, A. Light sensing and responses in marine microalgae. *Curr. Opin. Plant Biol.* **37**, 70–77 (2017).

38. Nakano, A. *et al.* Modified windmill porphyrin arrays: Coupled light-harvesting and charge separation, conformational relaxation in the S1 state, and S2-S2 energy transfer. *Chem. - A Eur. J.* **7**, 3134–3151 (2001).

39. Götze, J. & Saalfrank, P. Serine in BLUF domains displays spectral importance in computational models. *J. Photochem. Photobiol. B Biol.* **94**, 87–95 (2009).

40. Feng, Y. *et al.* Structure of a diatom photosystem II supercomplex containing a member of Lhcx family and dimeric FCPII. *Sci. Adv.* **9**, (2023).

41. Du, H., Fuh, R.-C. A., Li, J., Corkan, L. A. & Lindsey, J. S. PhotochemCAD: A Computer-Aided Design and Research Tool in Photochemistry. *Photochem. Photobiol.* **68**, 141–142 (1998).

42. Frank, H. A., Bautista, J. A., Josue, J. S. & Young, A. J. Mechanism of Nonphotochemical Quenching in Green Plants: Energies of the Lowest Excited Singlet States of Violaxanthin and Zeaxanthin. *Biochemistry* **39**, 2831–2837 (2000).

43. Akimoto, S. *et al.* Ultrafast Excitation Relaxation Dynamics of Lutein in Solution and in the Light-Harvesting Complexes II Isolated from Arabidopsis t haliana. *J. Phys. Chem. B* **109**, 12612–12619 (2005).

44. Frank, H. a *et al.* Effect of the Solvent Environment on the Spectroscopic Properties and Dynamics of the Lowest Excited States of Carotenoids. *J. Phys. Chem. B* **104**, 4569–4577 (2000).
