# Supplementary figures and images for: Applying an Anti-Kasha Model Resolves Differences Between Photosynthetic and Artificial Pigments (part 2 of 2)
Source: J Phys Chem B. 2025 Jul 23;129(31):7884–95. doi: 10.1021/acs.jpcb.5c02465 (PMC12337091; doi:10.1021/acs.jpcb.5c02465)

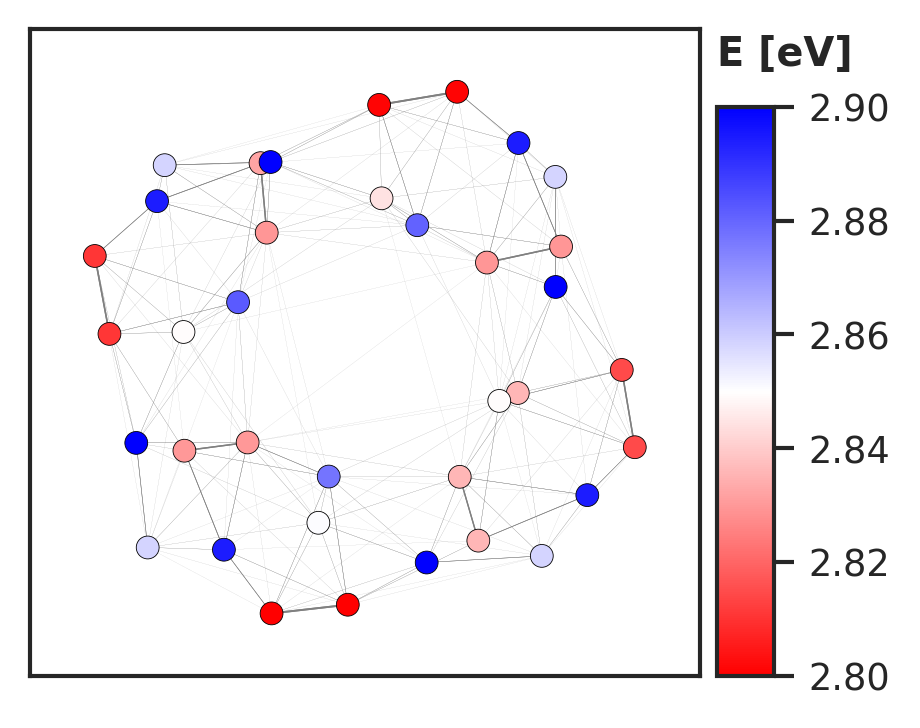

Supplement: Supplementary file 2 [file jp5c02465_si_002.zip › Fig4Analogues/tFCP/tFCP_onlyChla_B.png]

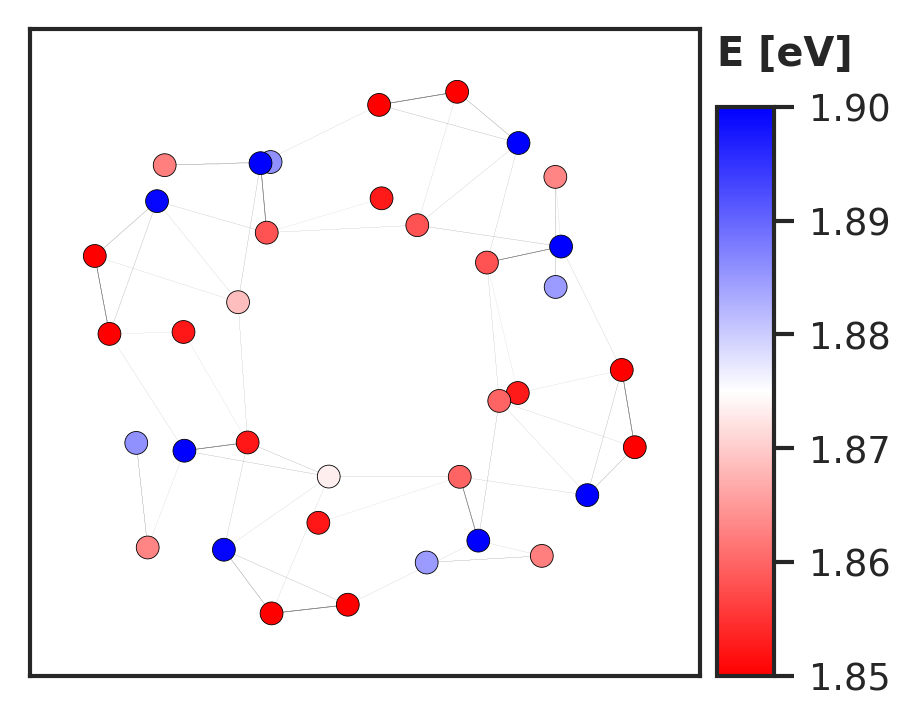

Supplement: Supplementary file 2 [file jp5c02465_si_002.zip › Fig4Analogues/tFCP/tFCP_onlyChla_Q.png]

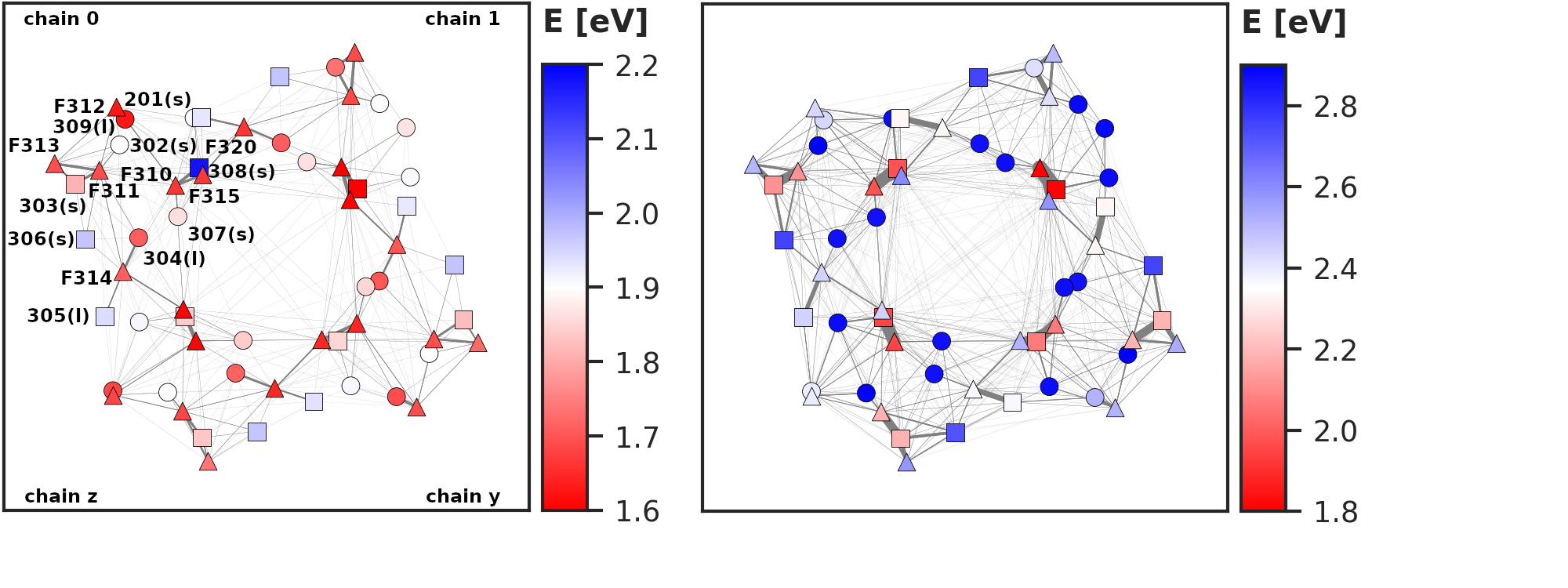

Supplement: Supplementary file 2 [file jp5c02465_si_002.zip › Fig4Analogues/tFCP/tFCP_WT_QandB.png]
