# Supplementary figures and images for: Applying an Anti-Kasha Model Resolves Differences Between Photosynthetic and Artificial Pigments (part 1 of 2)
Source: J Phys Chem B. 2025 Jul 23;129(31):7884–95. doi: 10.1021/acs.jpcb.5c02465 (PMC12337091; doi:10.1021/acs.jpcb.5c02465)

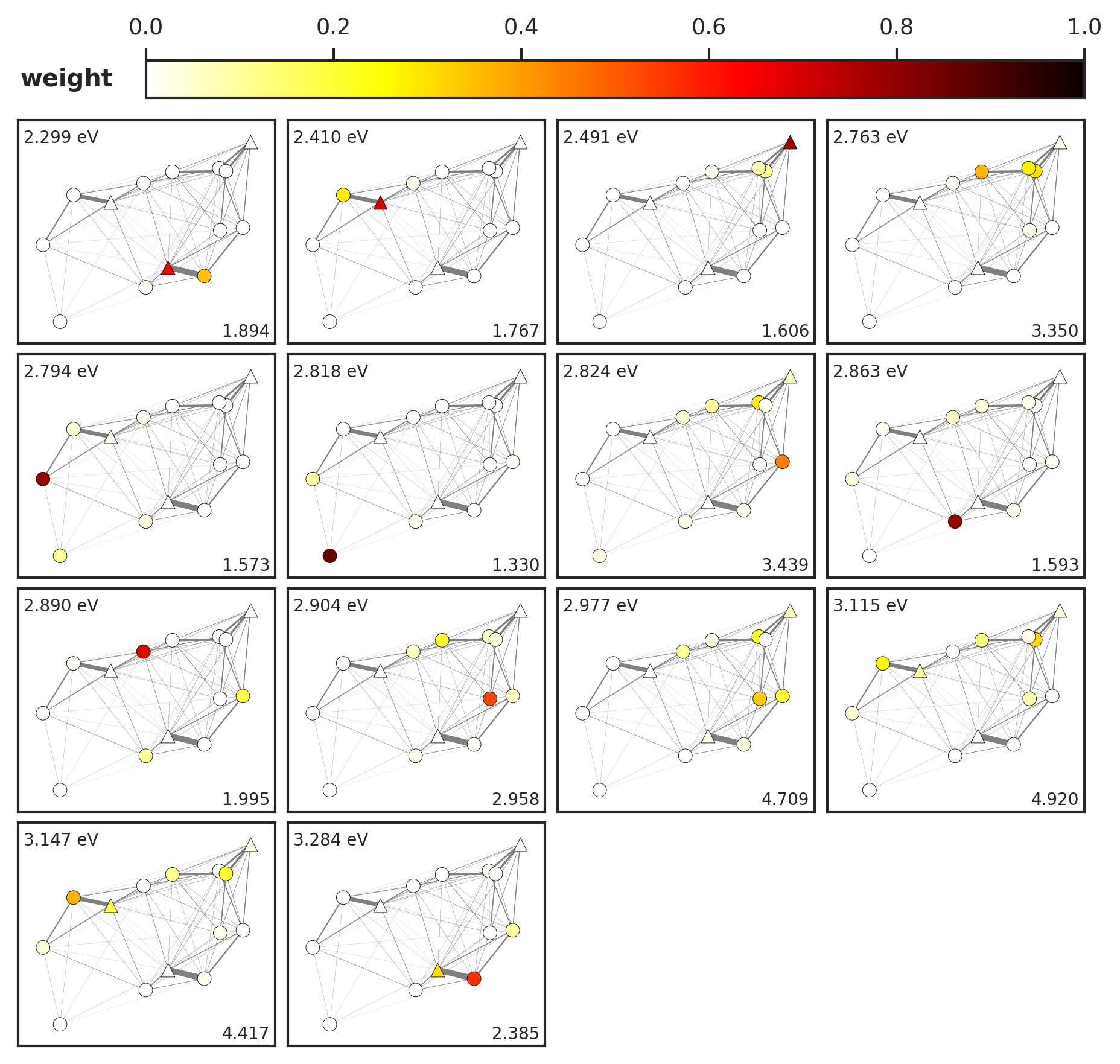

Supplement: Supplementary file 2 [file jp5c02465_si_002.zip › Fig6Analogues/CP24/CP24_ChlbreplacedbyChla_B.png]

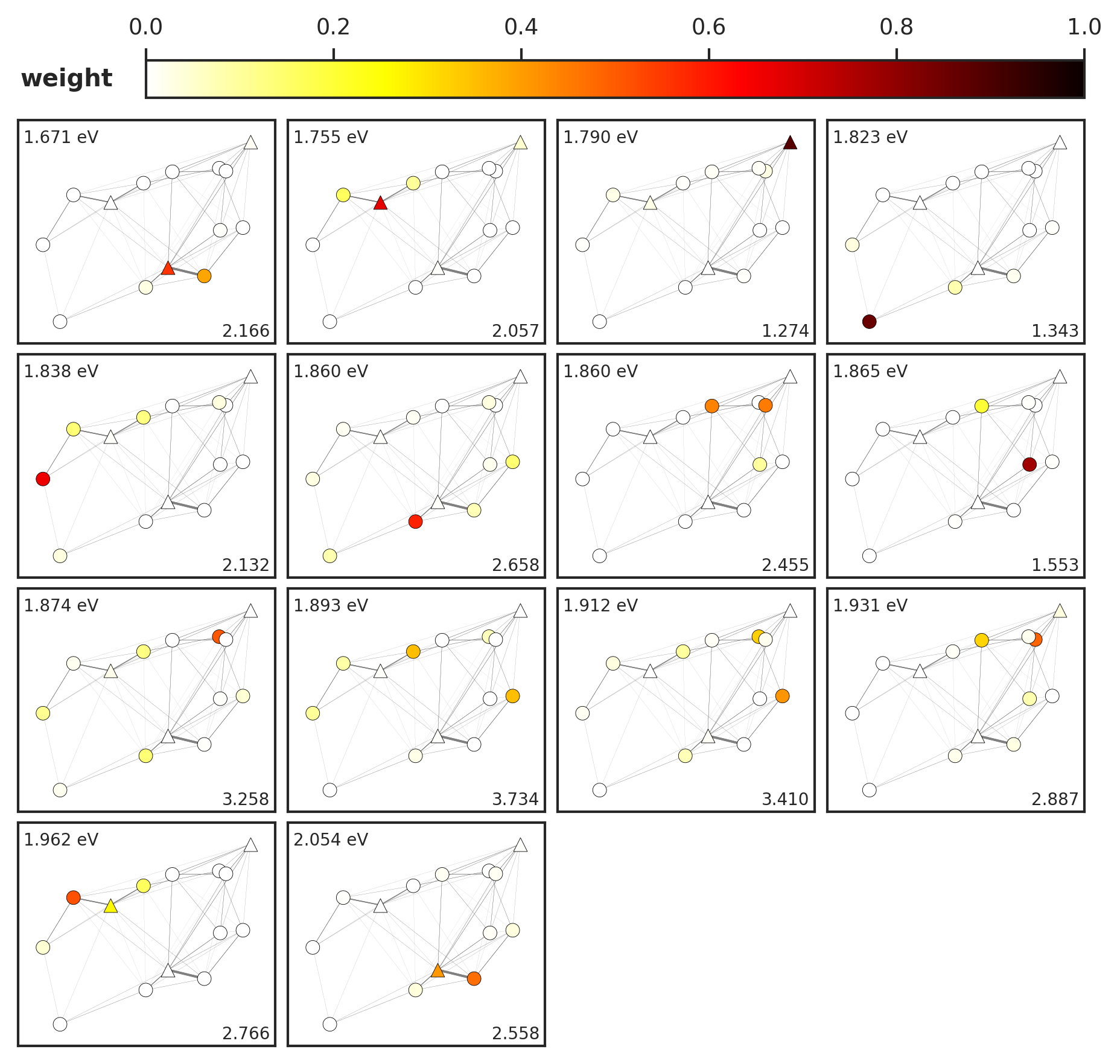

Supplement: Supplementary file 2 [file jp5c02465_si_002.zip › Fig6Analogues/CP24/CP24_ChlbreplacedbyChla_Q.png]

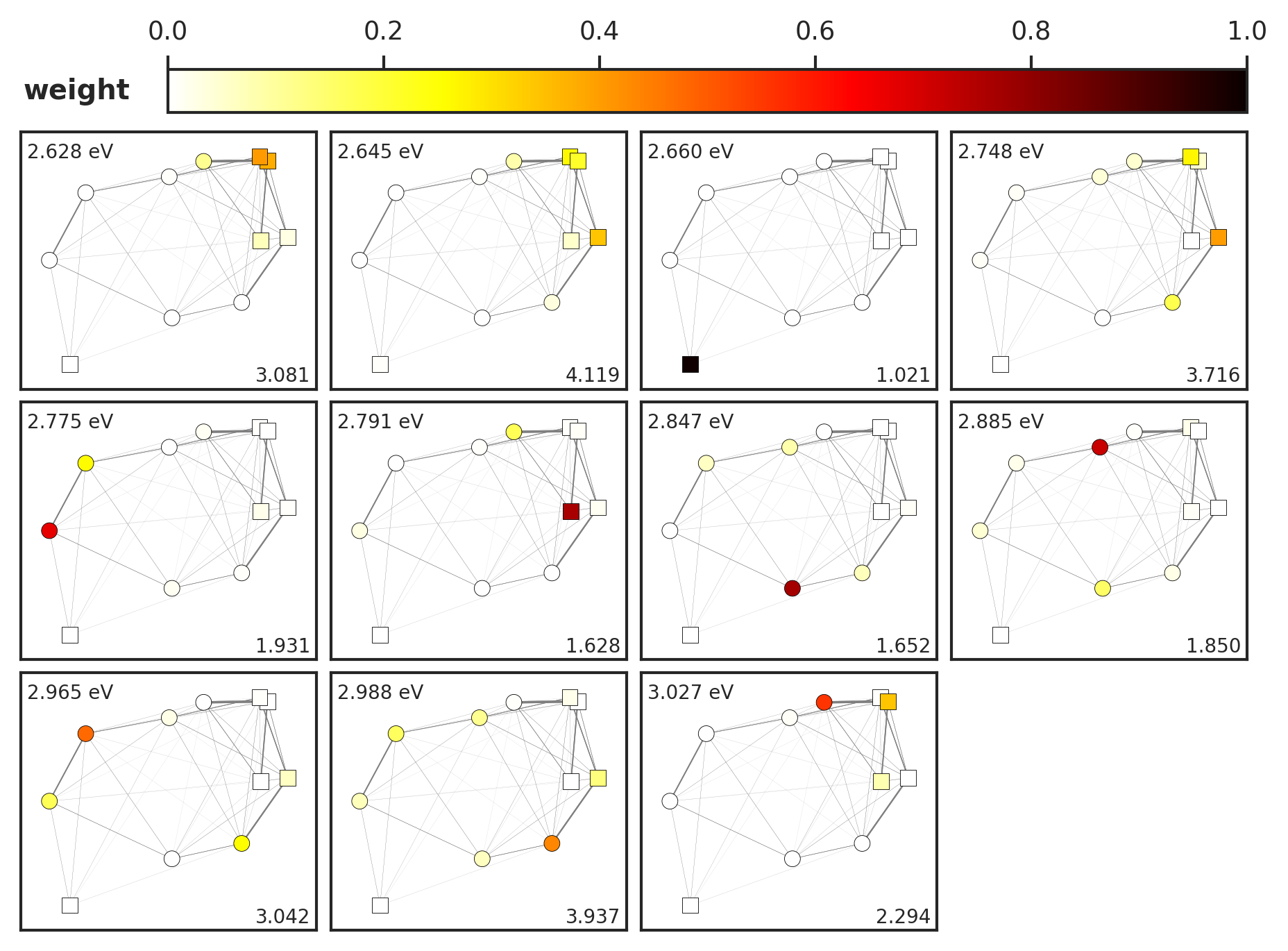

Supplement: Supplementary file 2 [file jp5c02465_si_002.zip › Fig6Analogues/CP24/CP24_noCrts_B.png]

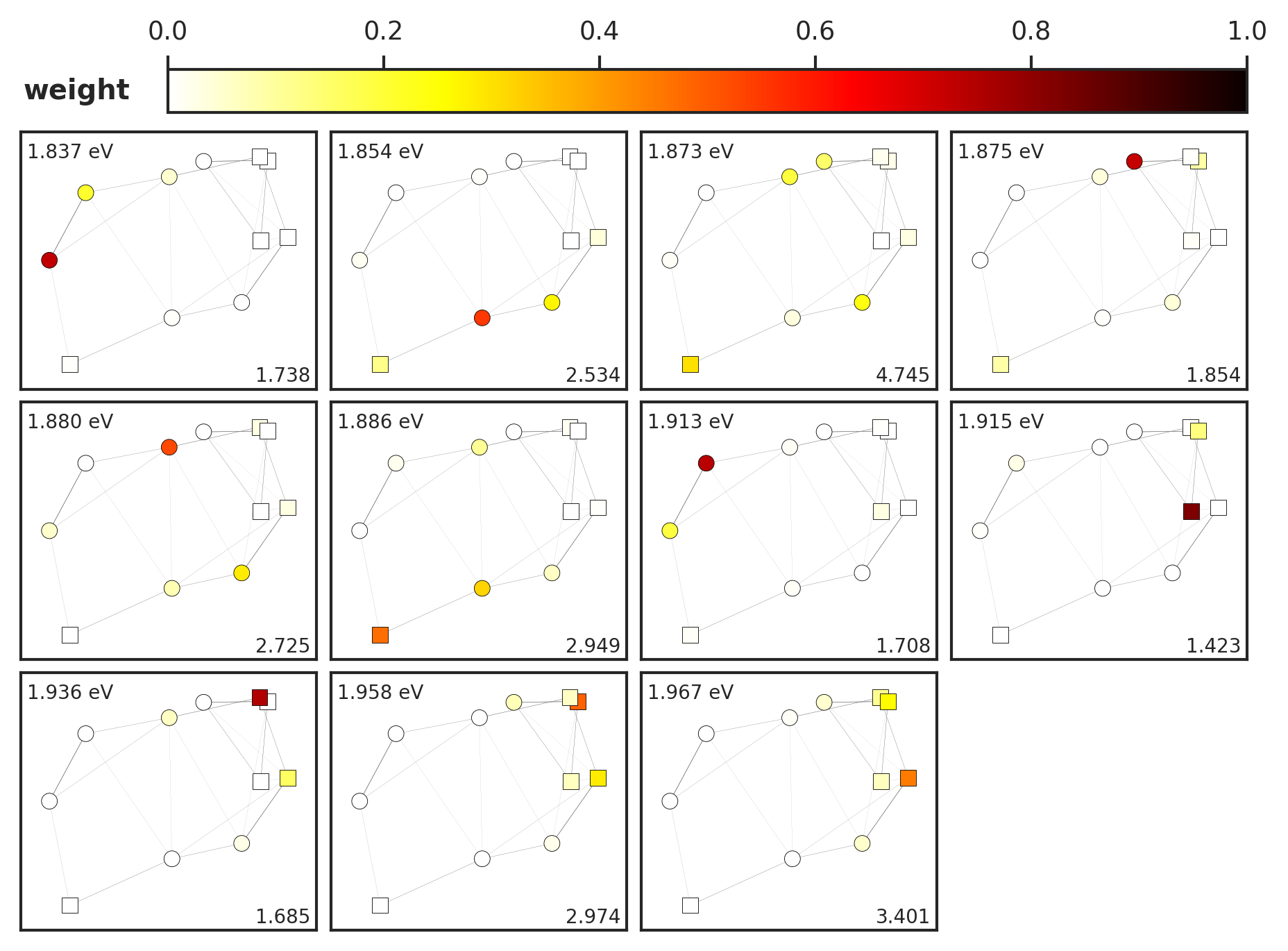

Supplement: Supplementary file 2 [file jp5c02465_si_002.zip › Fig6Analogues/CP24/CP24_noCrts_Q.png]

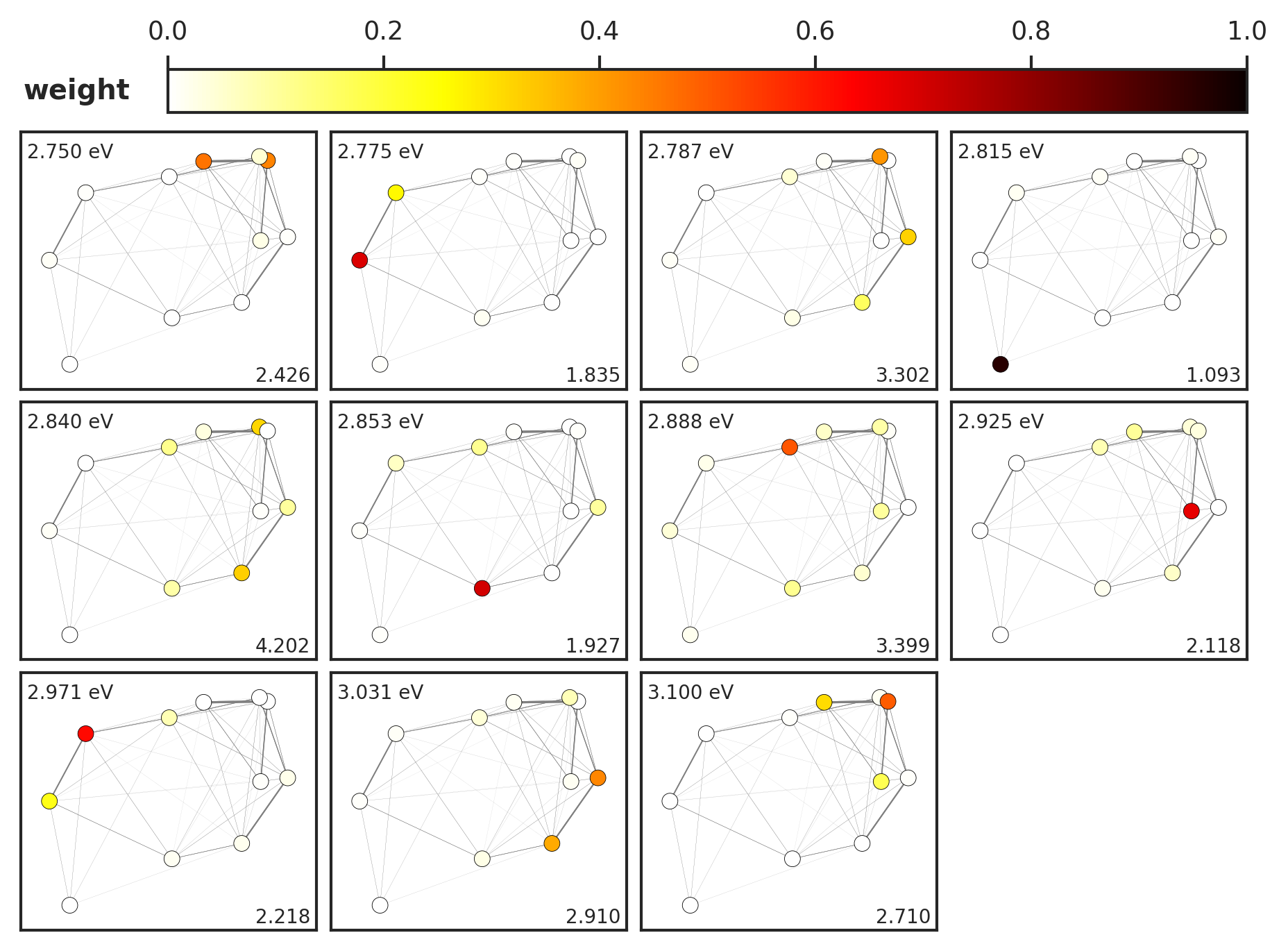

Supplement: Supplementary file 2 [file jp5c02465_si_002.zip › Fig6Analogues/CP24/CP24_onlyChla_B.png]

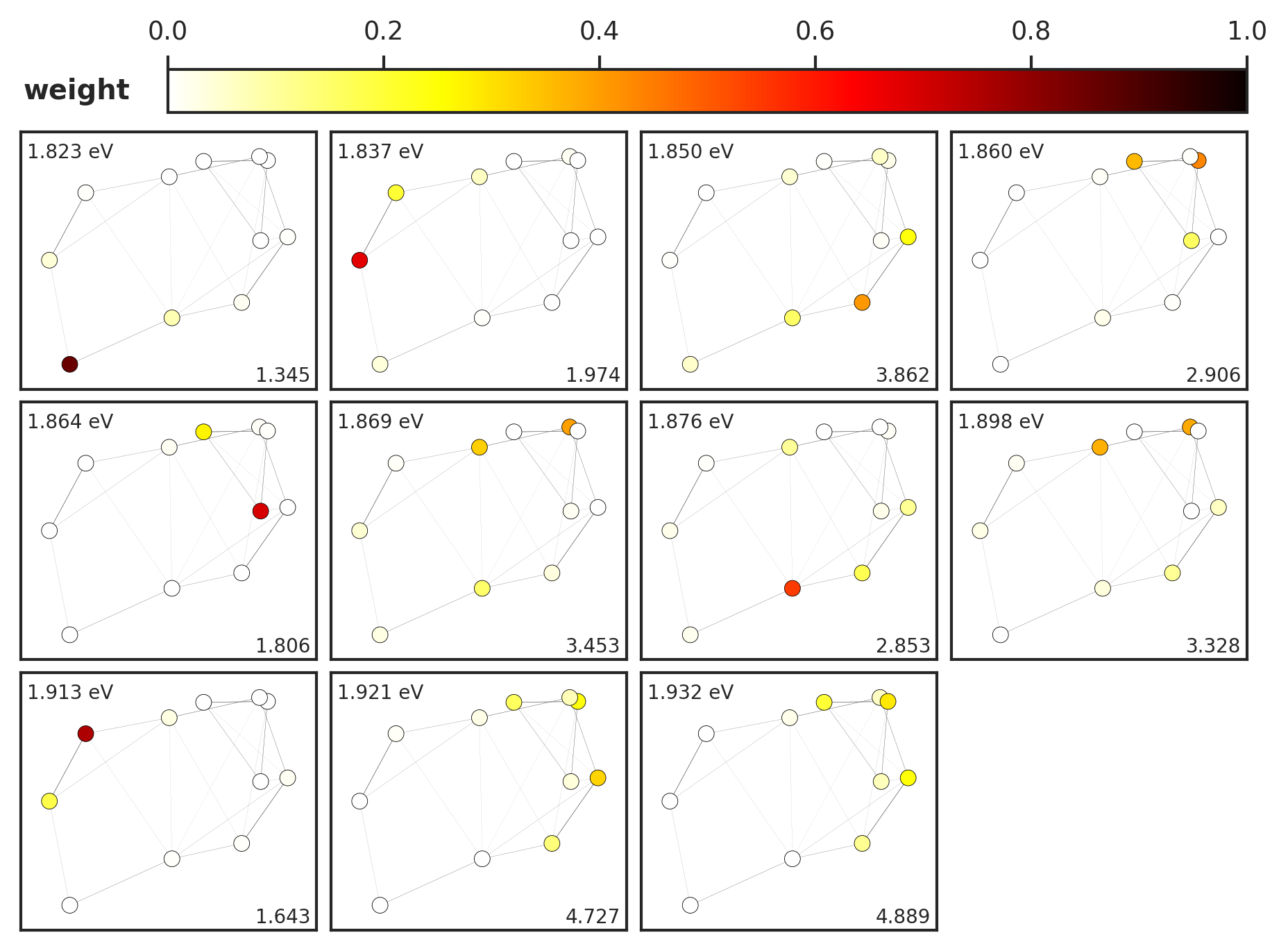

Supplement: Supplementary file 2 [file jp5c02465_si_002.zip › Fig6Analogues/CP24/CP24_onlyChla_Q.png]

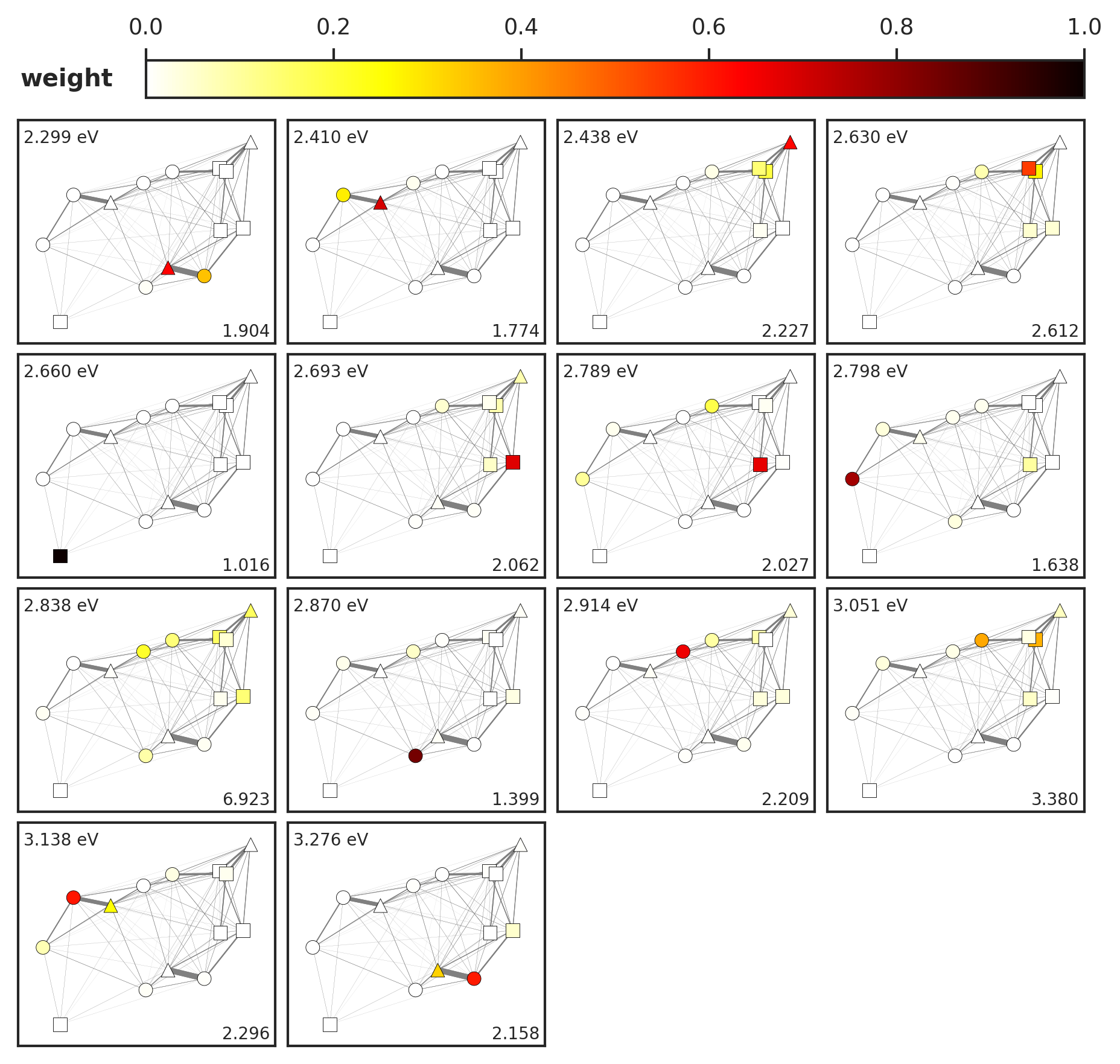

Supplement: Supplementary file 2 [file jp5c02465_si_002.zip › Fig6Analogues/CP24/CP24_WT_B.png]

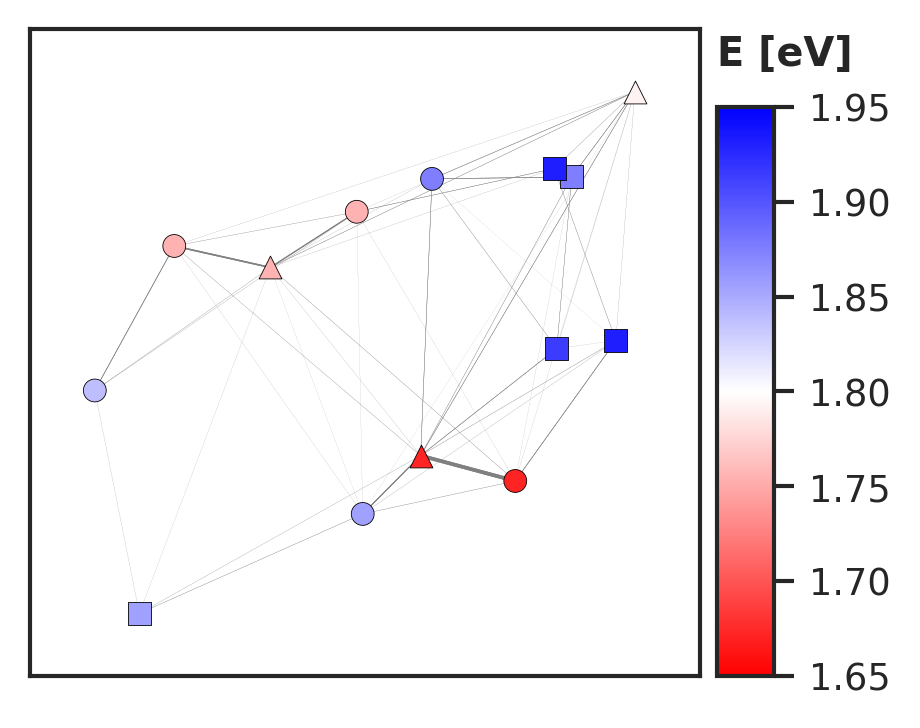

Supplement: Supplementary file 2 [file jp5c02465_si_002.zip › Fig6Analogues/CP24/CP24_WT_Q.png]

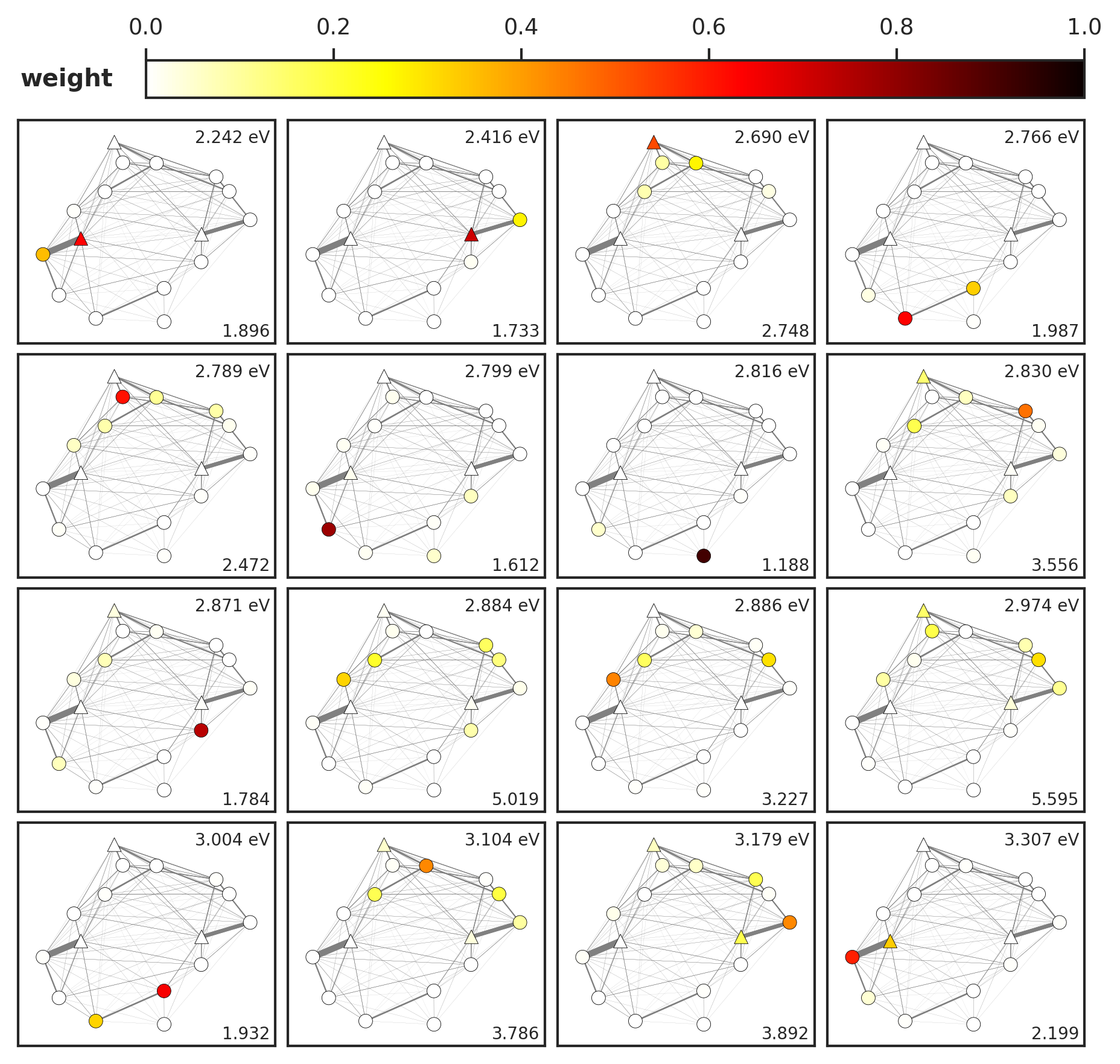

Supplement: Supplementary file 2 [file jp5c02465_si_002.zip › Fig6Analogues/CP26/CP26_ChlbreplacedbyChla_B.png]

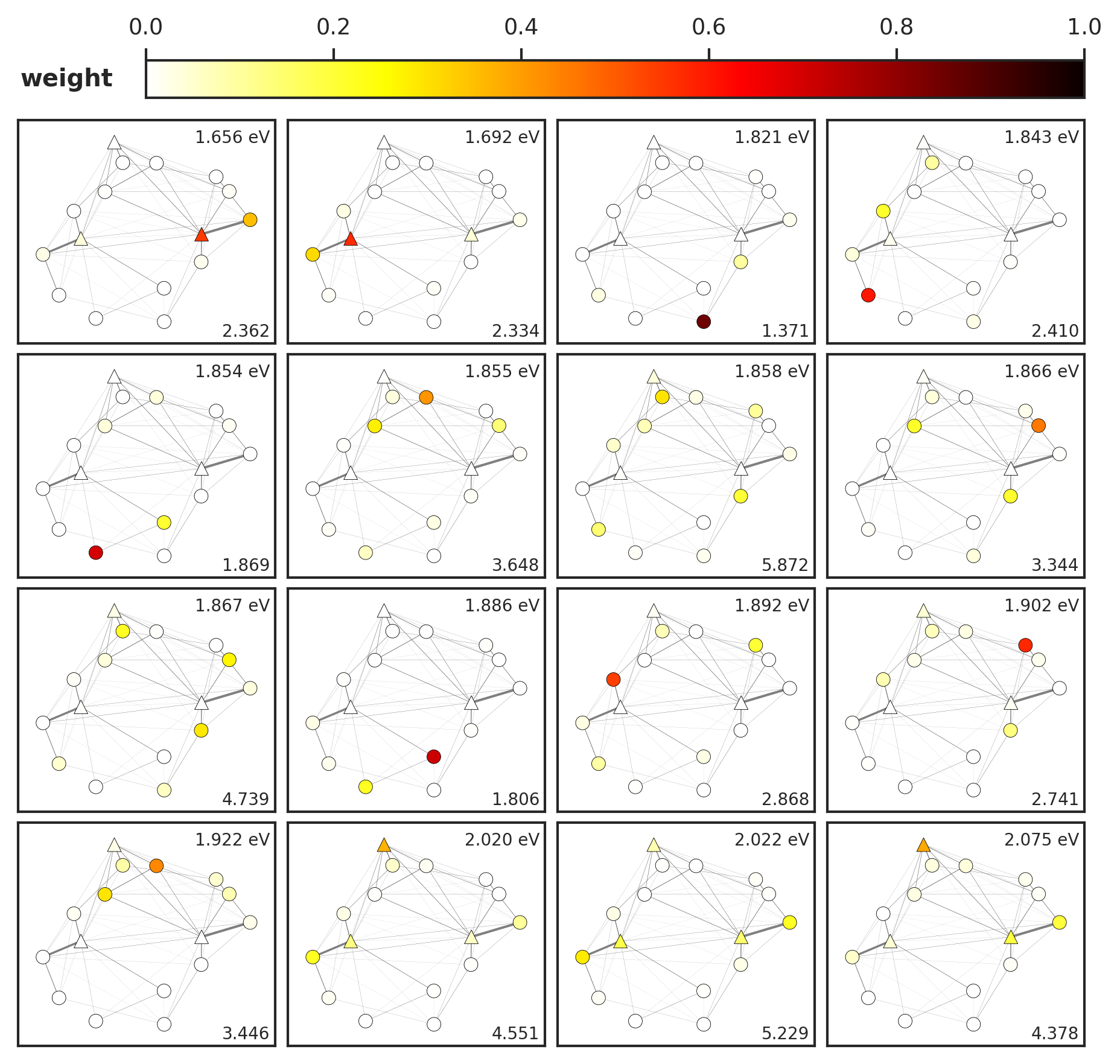

Supplement: Supplementary file 2 [file jp5c02465_si_002.zip › Fig6Analogues/CP26/CP26_ChlbreplacedbyChla_Q.png]

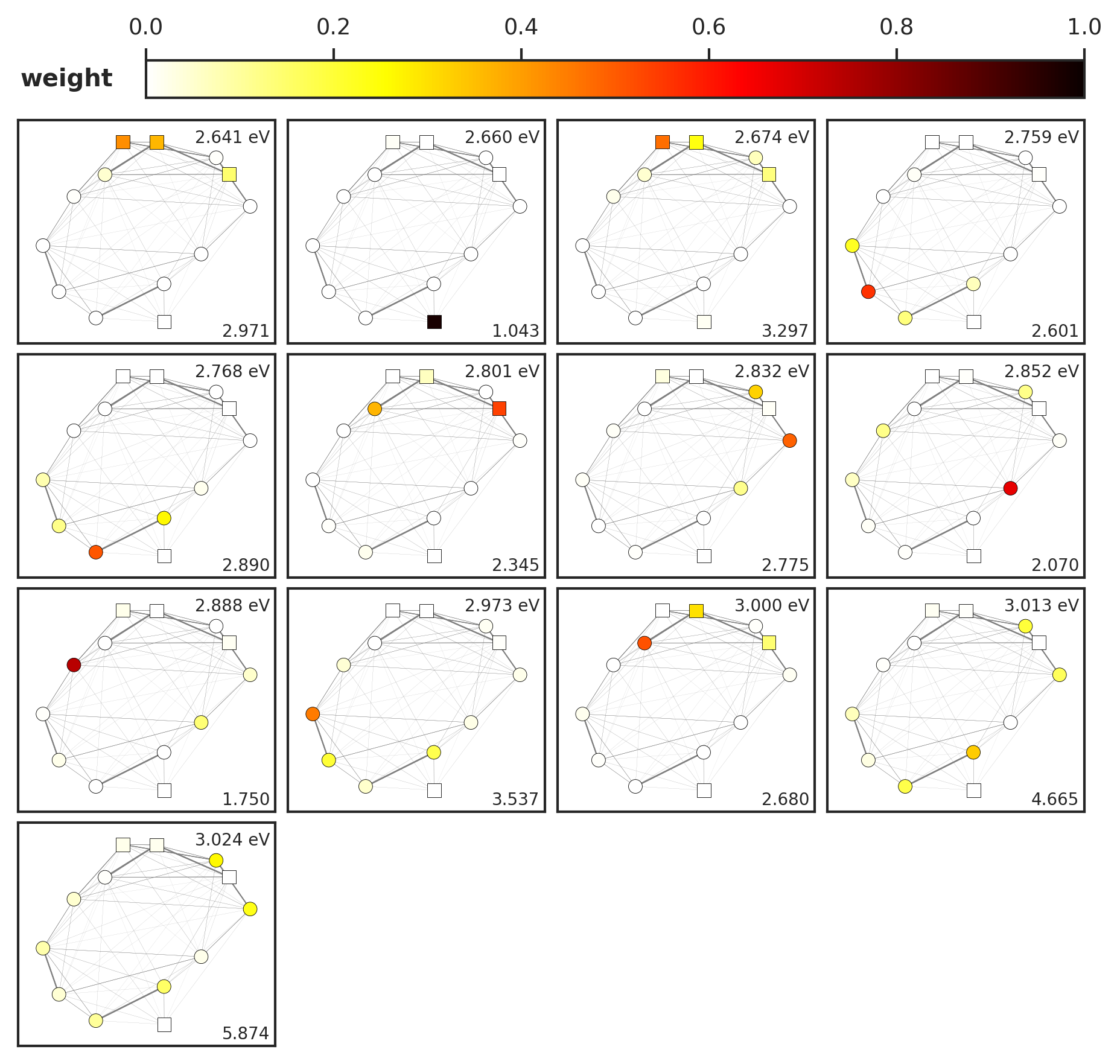

Supplement: Supplementary file 2 [file jp5c02465_si_002.zip › Fig6Analogues/CP26/CP26_noCrts_B.png]

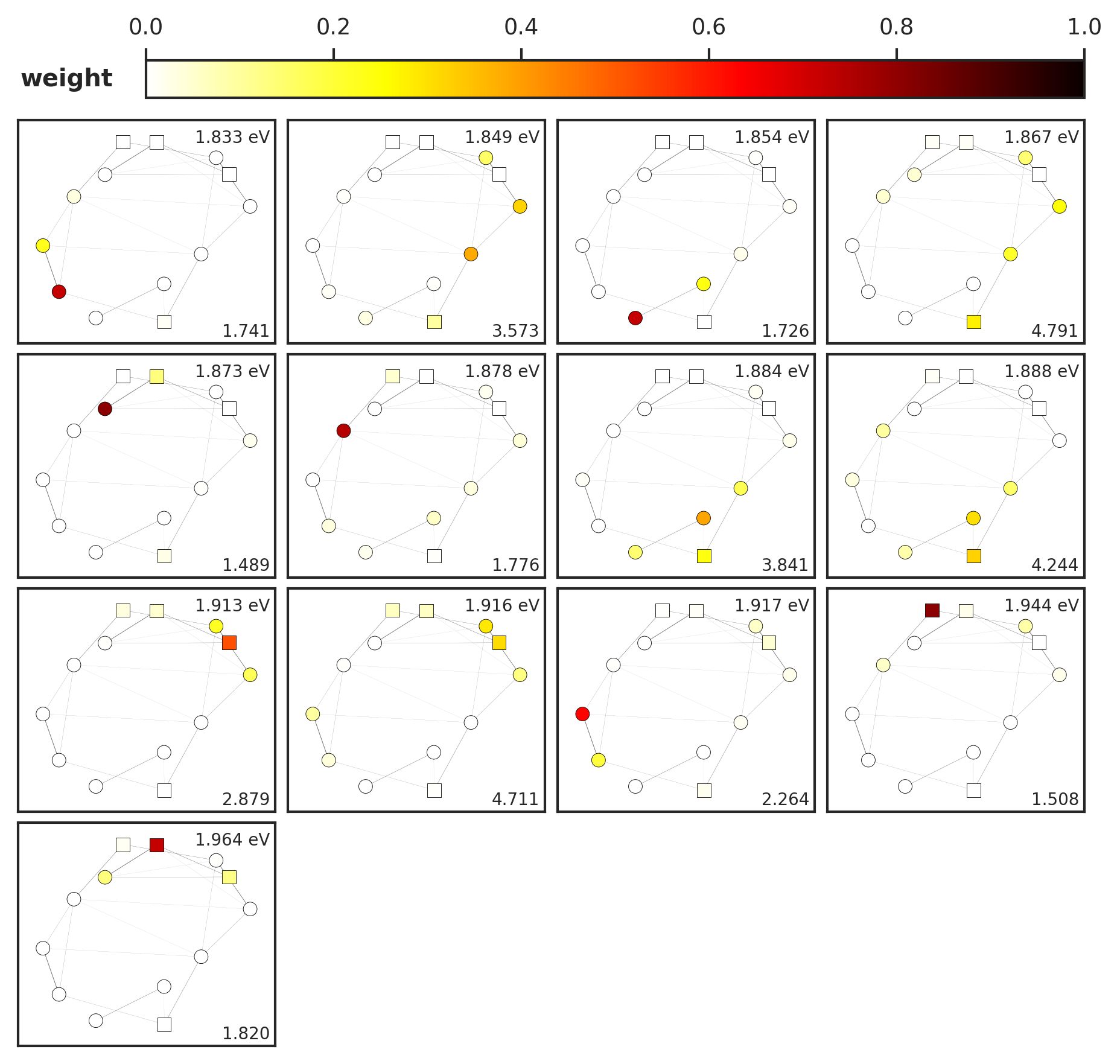

Supplement: Supplementary file 2 [file jp5c02465_si_002.zip › Fig6Analogues/CP26/CP26_noCrts_Q.png]

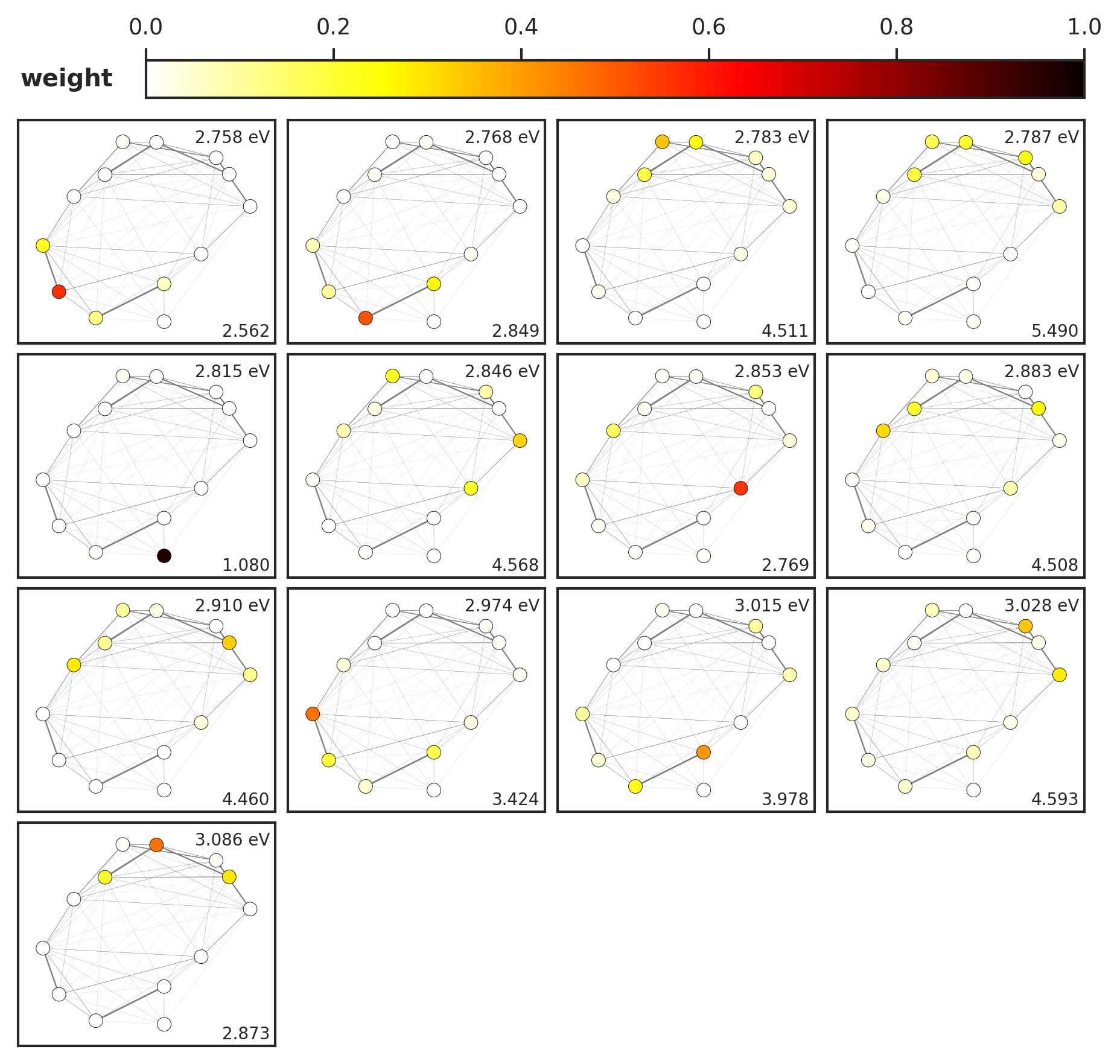

Supplement: Supplementary file 2 [file jp5c02465_si_002.zip › Fig6Analogues/CP26/CP26_onlyChla_B.png]

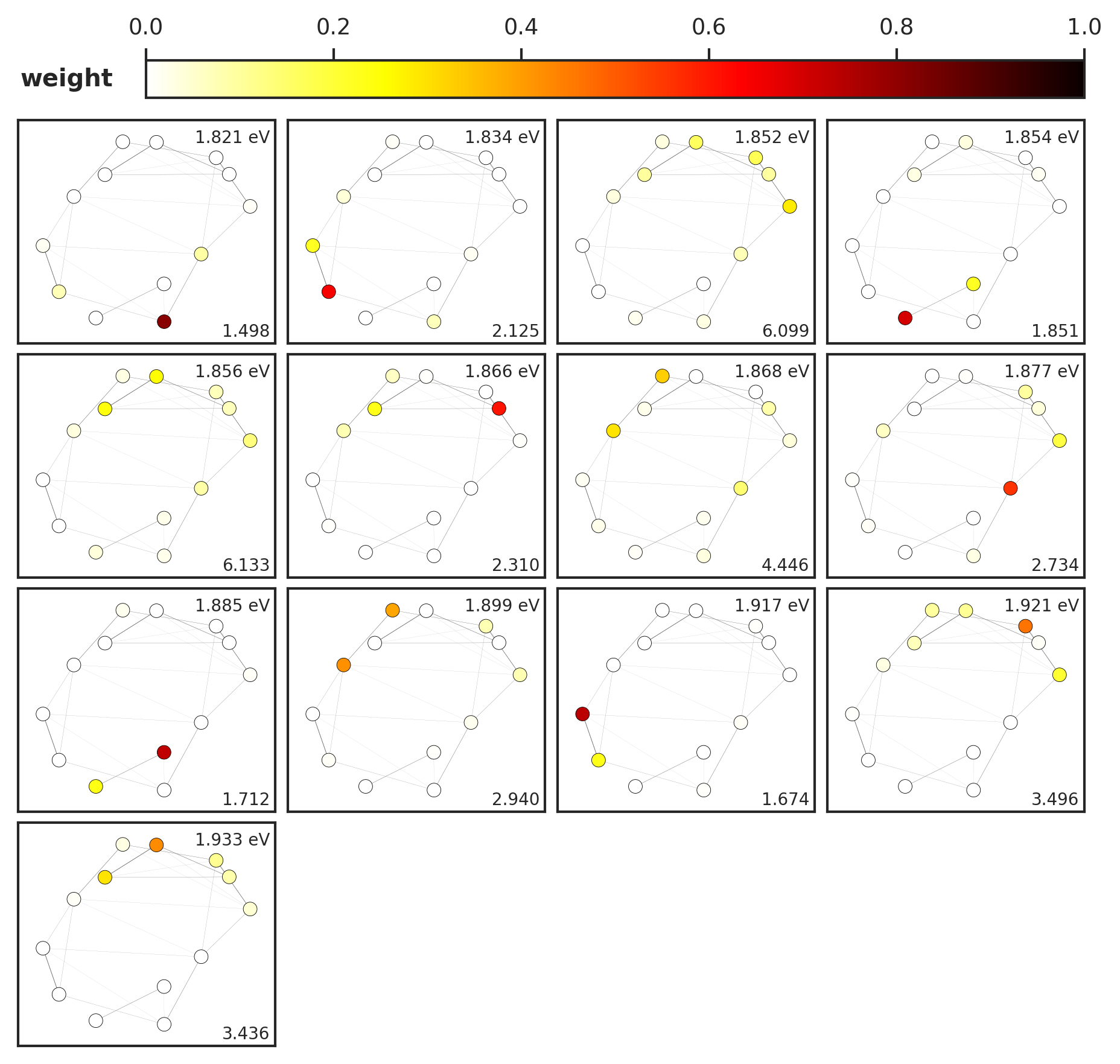

Supplement: Supplementary file 2 [file jp5c02465_si_002.zip › Fig6Analogues/CP26/CP26_onlyChla_Q.png]

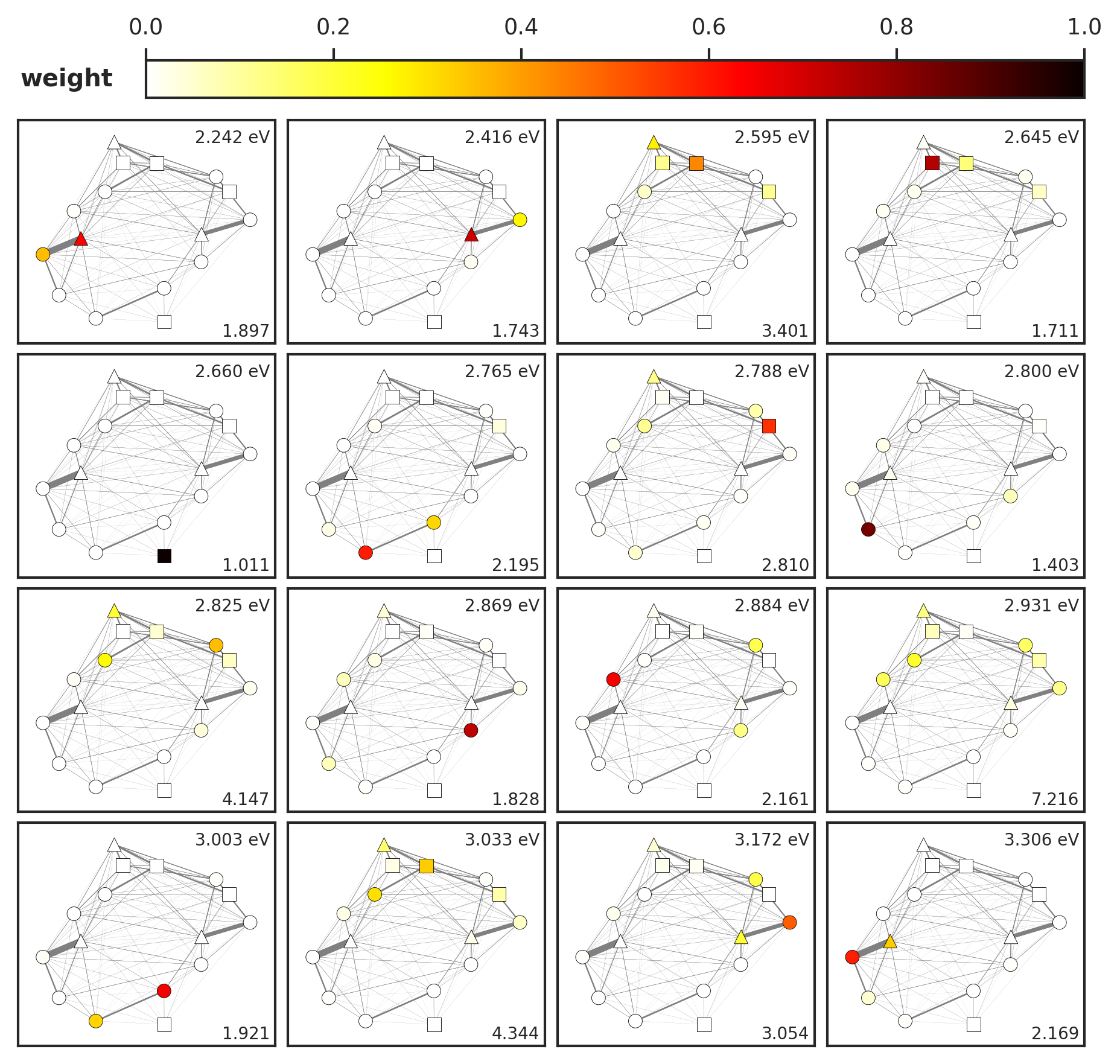

Supplement: Supplementary file 2 [file jp5c02465_si_002.zip › Fig6Analogues/CP26/CP26_WT_B.png]

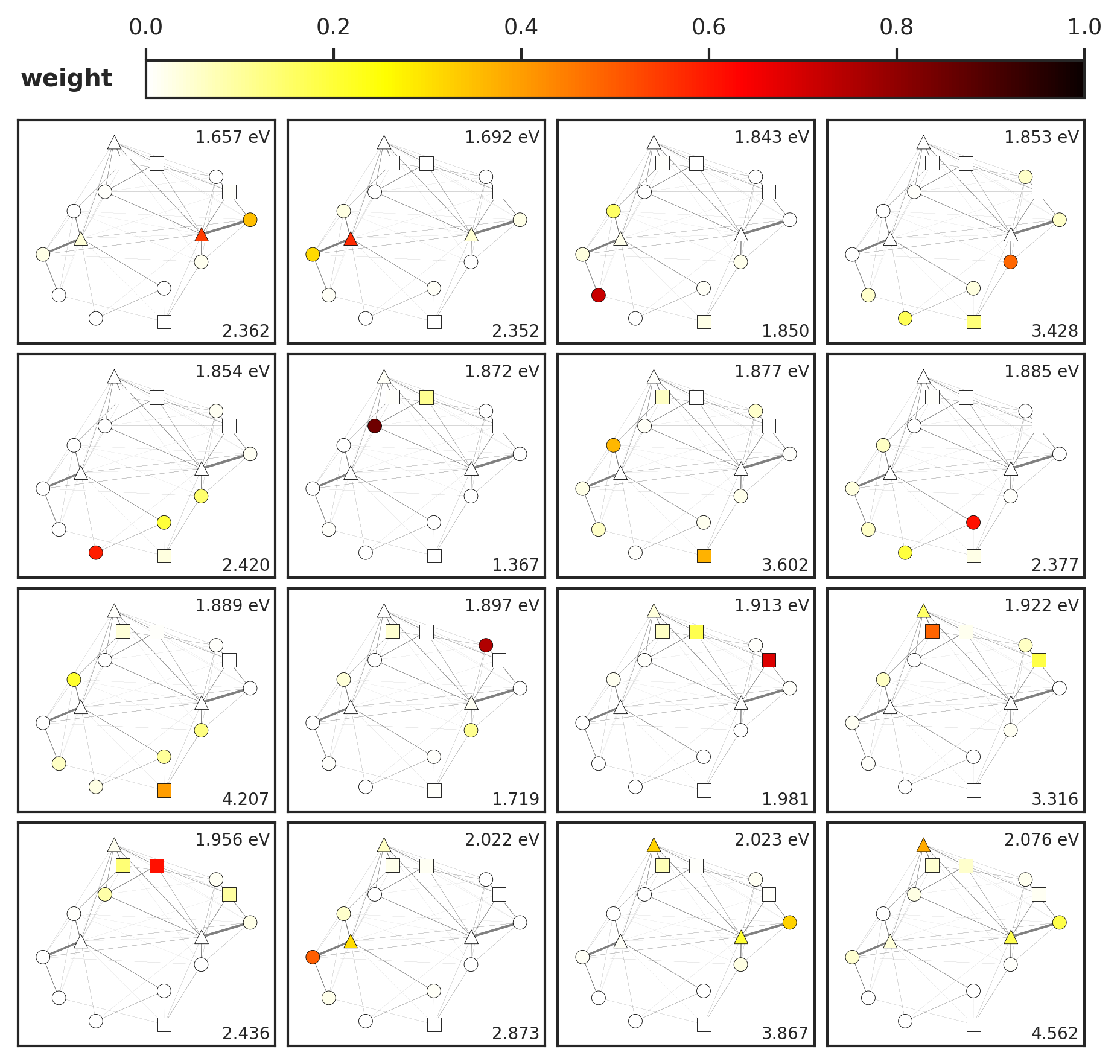

Supplement: Supplementary file 2 [file jp5c02465_si_002.zip › Fig6Analogues/CP26/CP26_WT_Q.png]

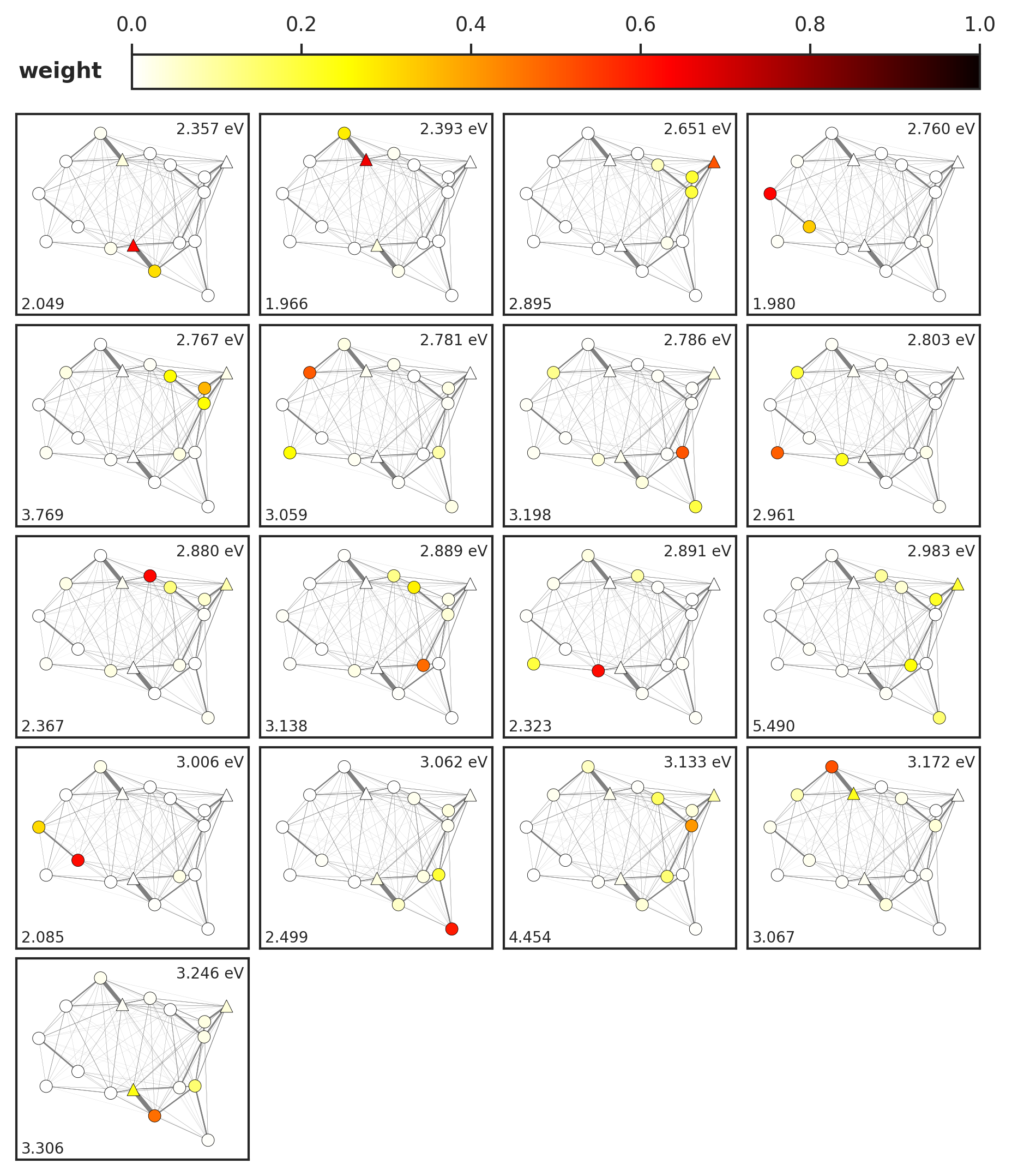

Supplement: Supplementary file 2 [file jp5c02465_si_002.zip › Fig6Analogues/CP29/CP29_ChlbreplacedbyChla_B.png]

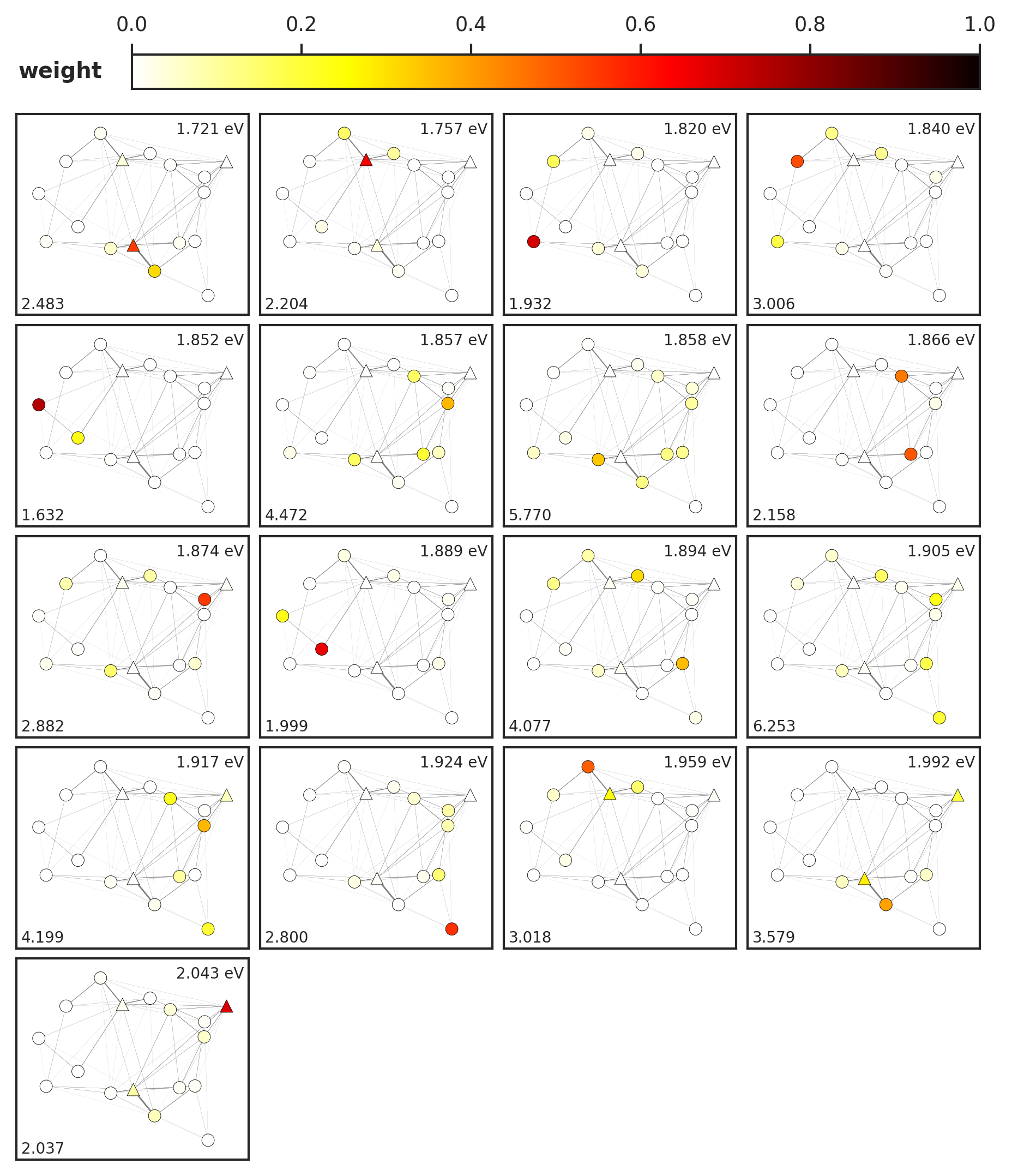

Supplement: Supplementary file 2 [file jp5c02465_si_002.zip › Fig6Analogues/CP29/CP29_ChlbreplacedbyChla_Q.png]

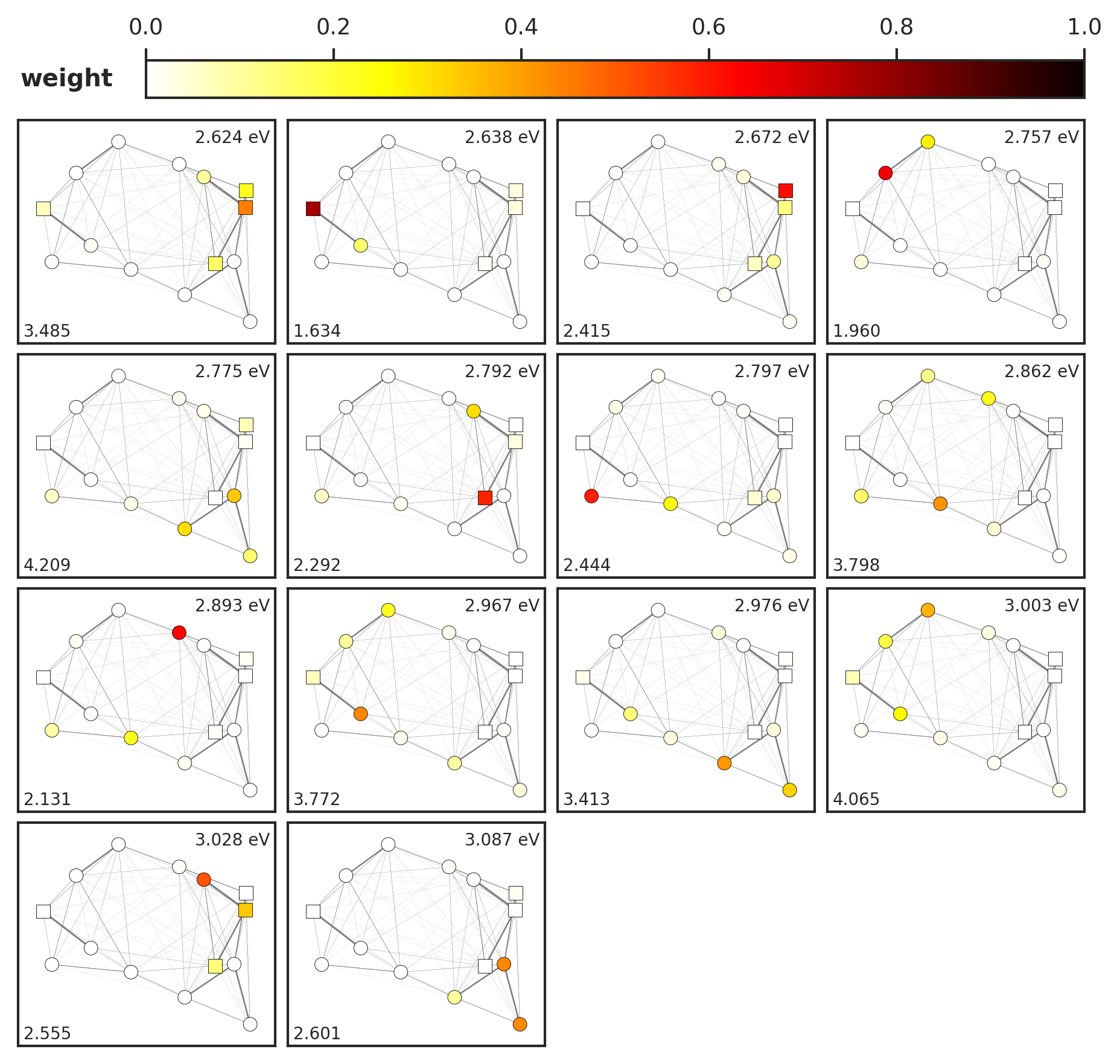

Supplement: Supplementary file 2 [file jp5c02465_si_002.zip › Fig6Analogues/CP29/CP29_noCrts_B.png]

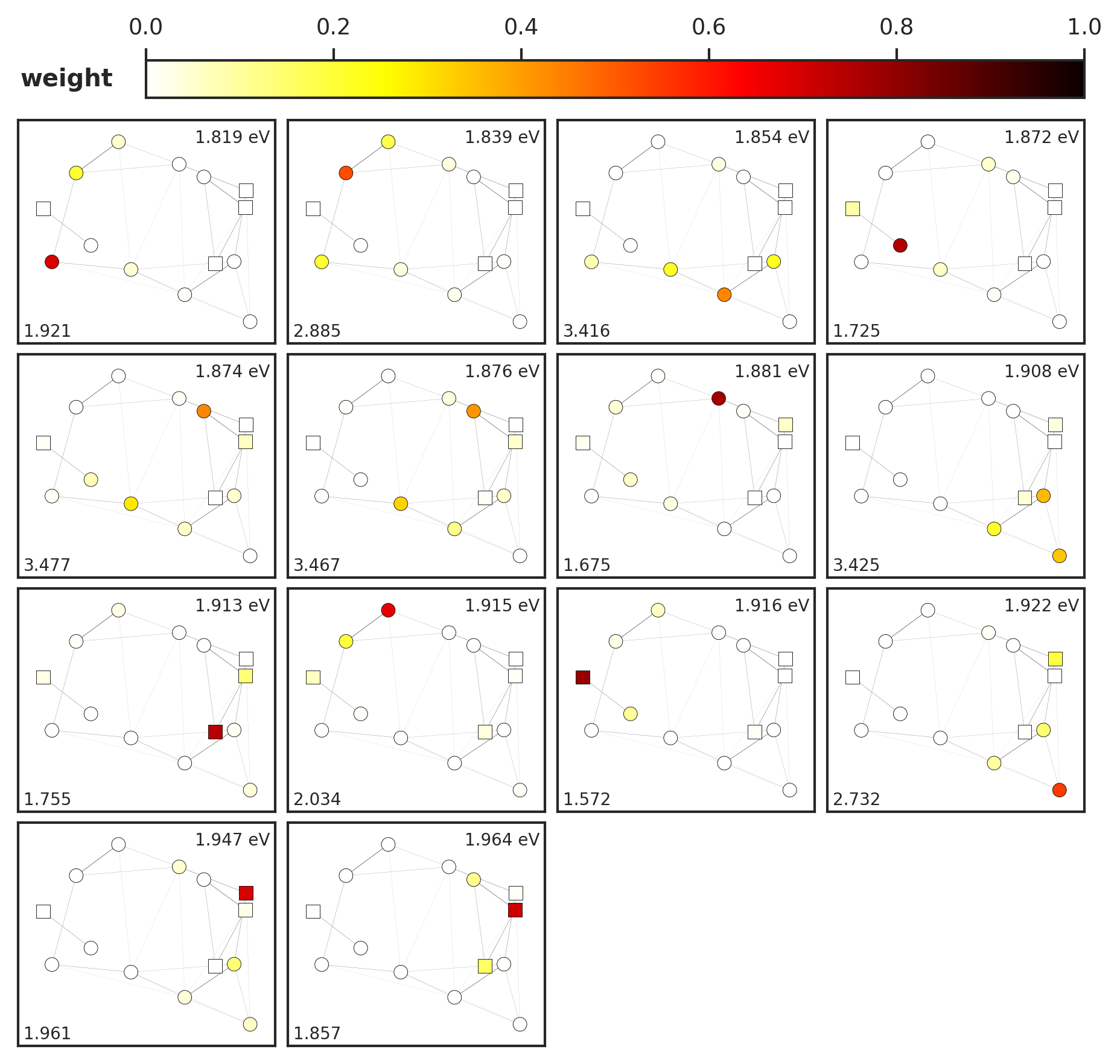

Supplement: Supplementary file 2 [file jp5c02465_si_002.zip › Fig6Analogues/CP29/CP29_noCrts_Q.png]

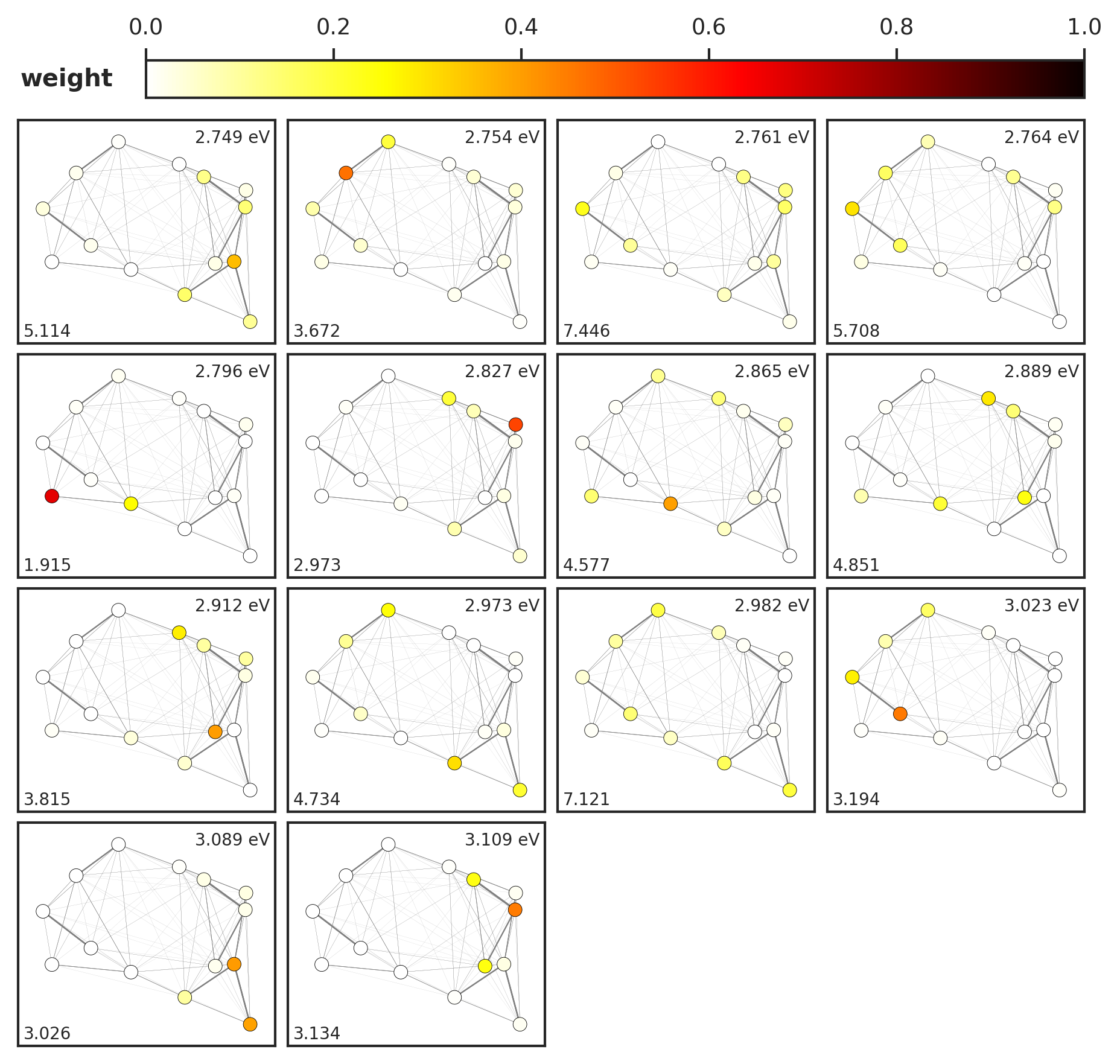

Supplement: Supplementary file 2 [file jp5c02465_si_002.zip › Fig6Analogues/CP29/CP29_onlyChla_B.png]

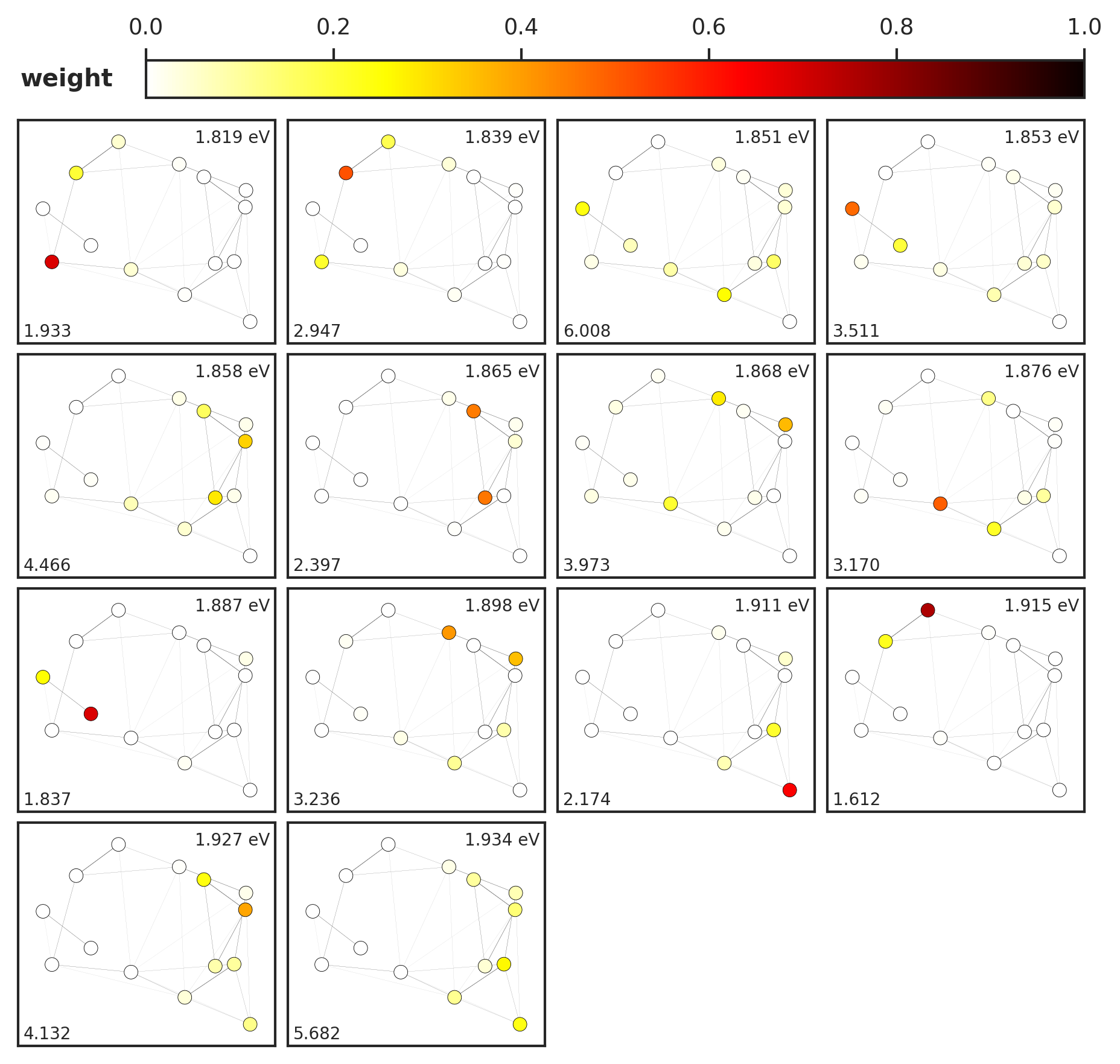

Supplement: Supplementary file 2 [file jp5c02465_si_002.zip › Fig6Analogues/CP29/CP29_onlyChla_Q.png]

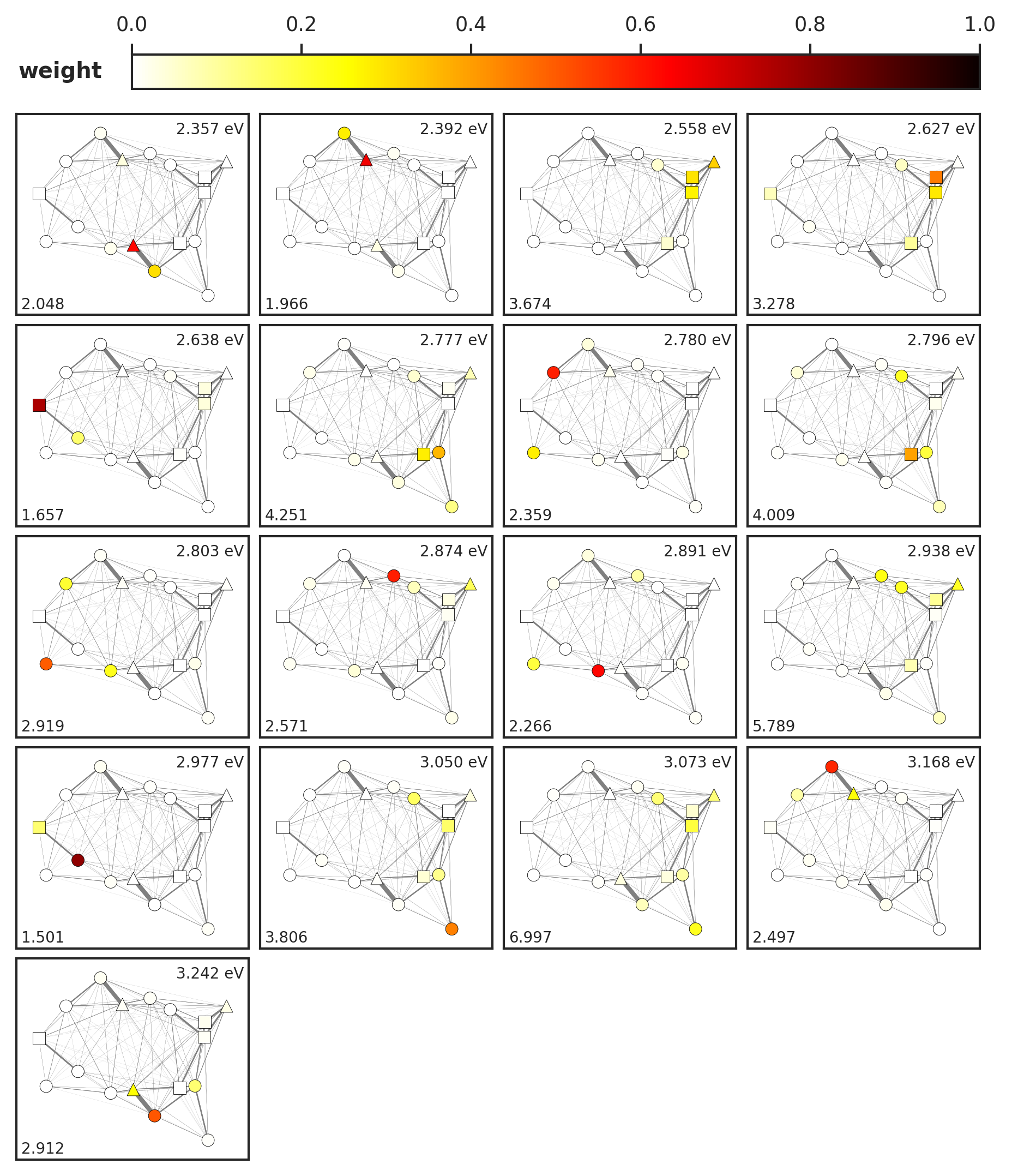

Supplement: Supplementary file 2 [file jp5c02465_si_002.zip › Fig6Analogues/CP29/CP29_WT_B.png]

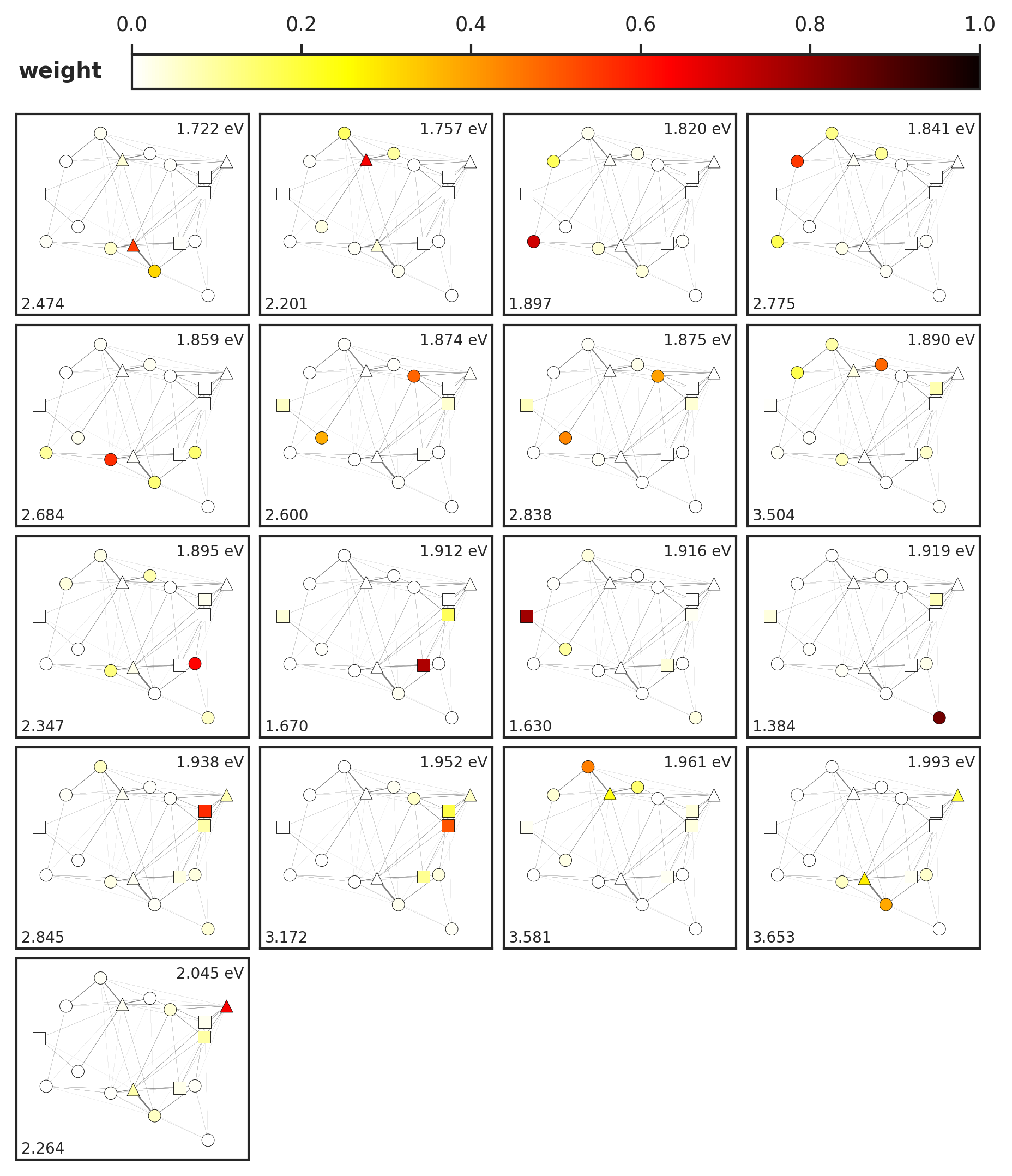

Supplement: Supplementary file 2 [file jp5c02465_si_002.zip › Fig6Analogues/CP29/CP29_WT_Q.png]

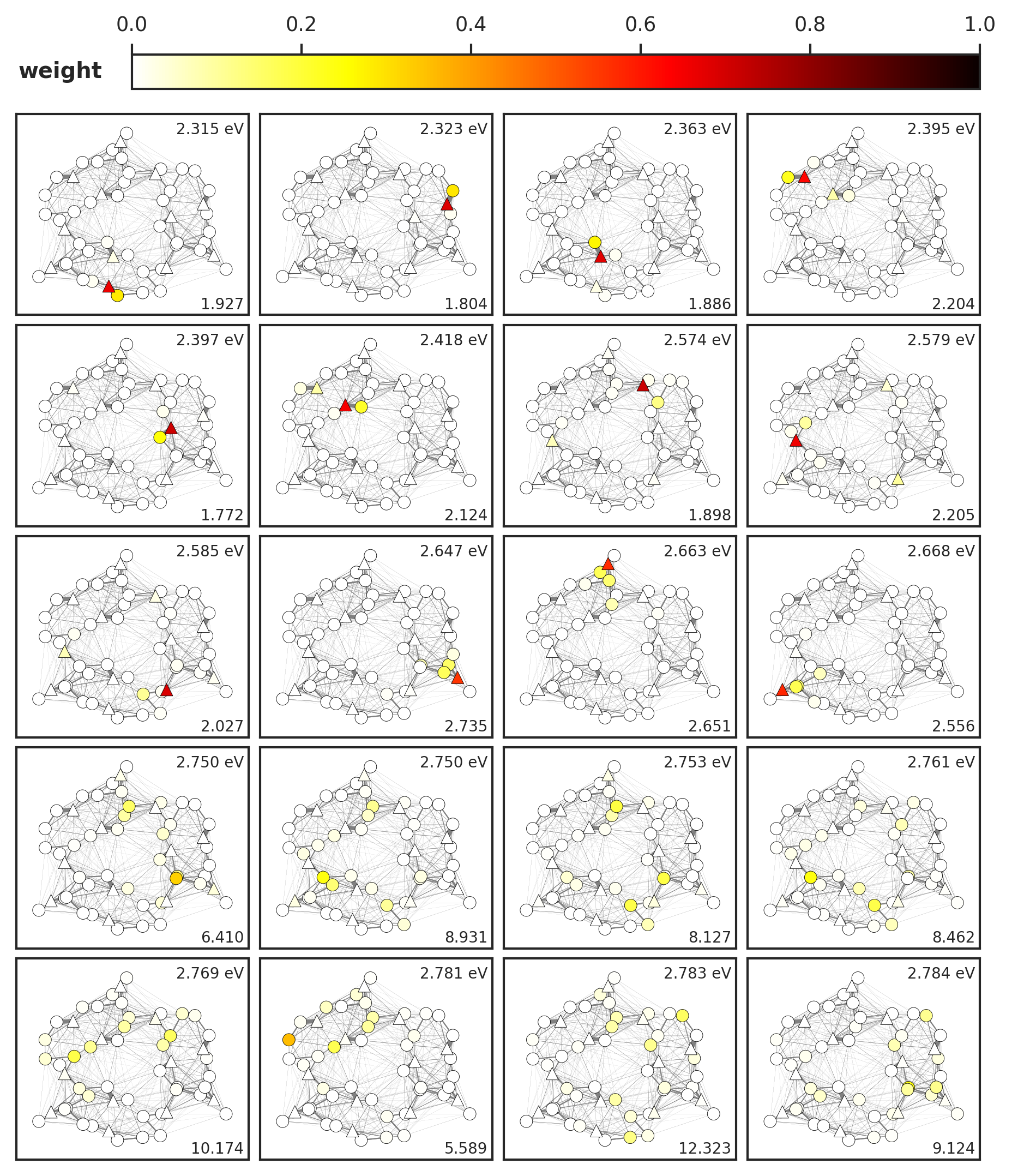

Supplement: Supplementary file 2 [file jp5c02465_si_002.zip › Fig6Analogues/LHCII/LHCII_ChlbreplacedbyChla_B_part1.png]

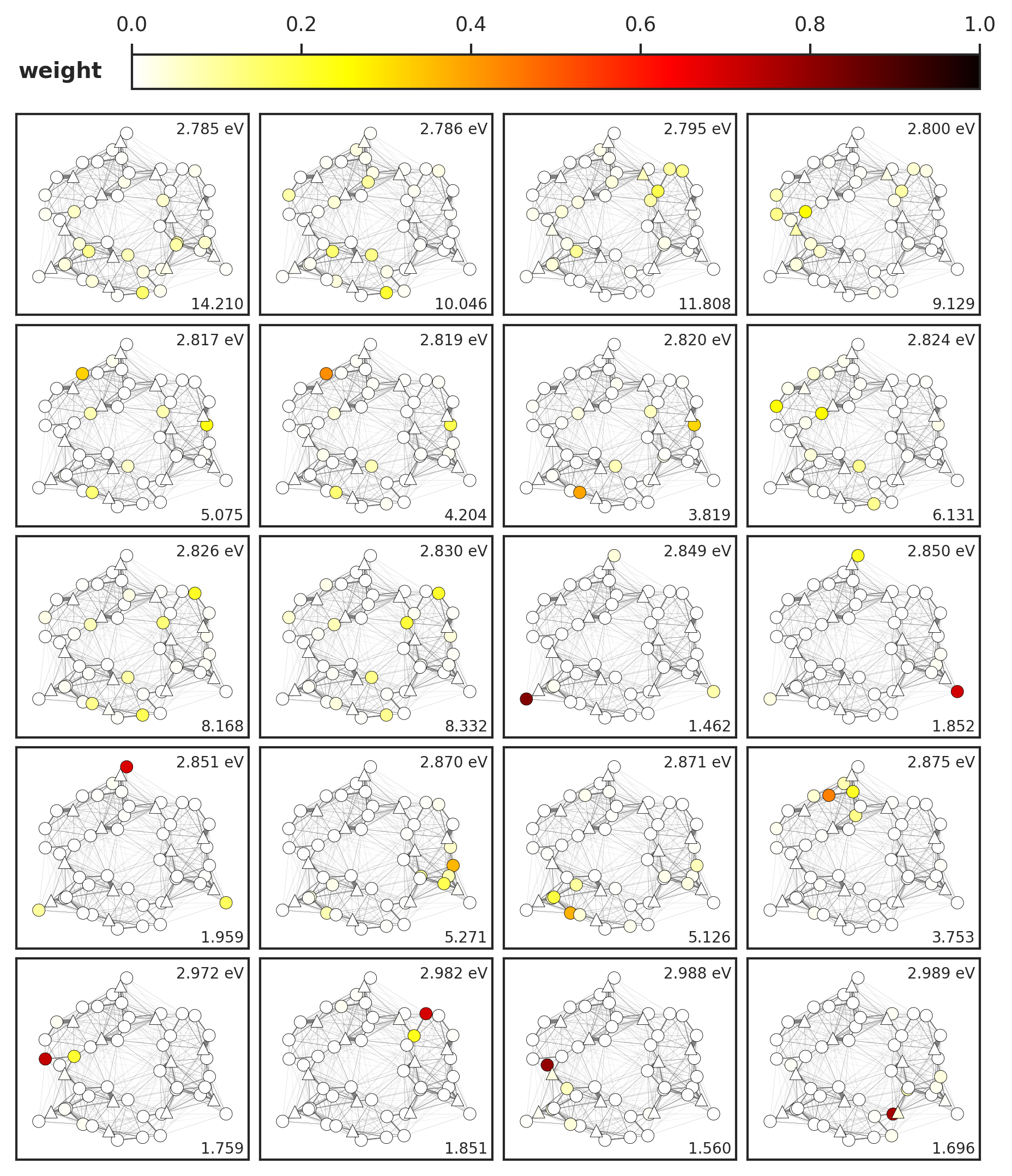

Supplement: Supplementary file 2 [file jp5c02465_si_002.zip › Fig6Analogues/LHCII/LHCII_ChlbreplacedbyChla_B_part2.png]

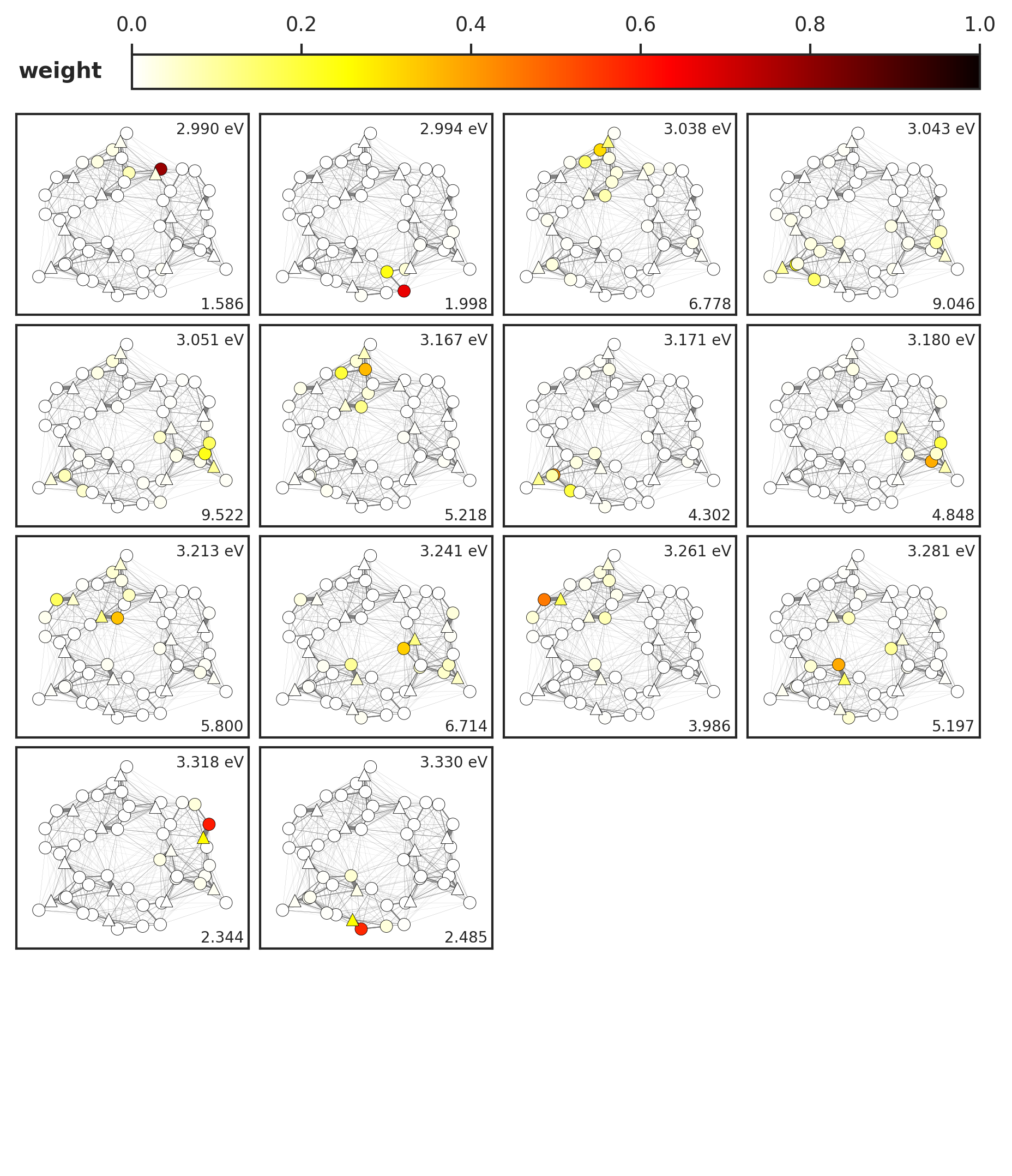

Supplement: Supplementary file 2 [file jp5c02465_si_002.zip › Fig6Analogues/LHCII/LHCII_ChlbreplacedbyChla_B_part3.png]

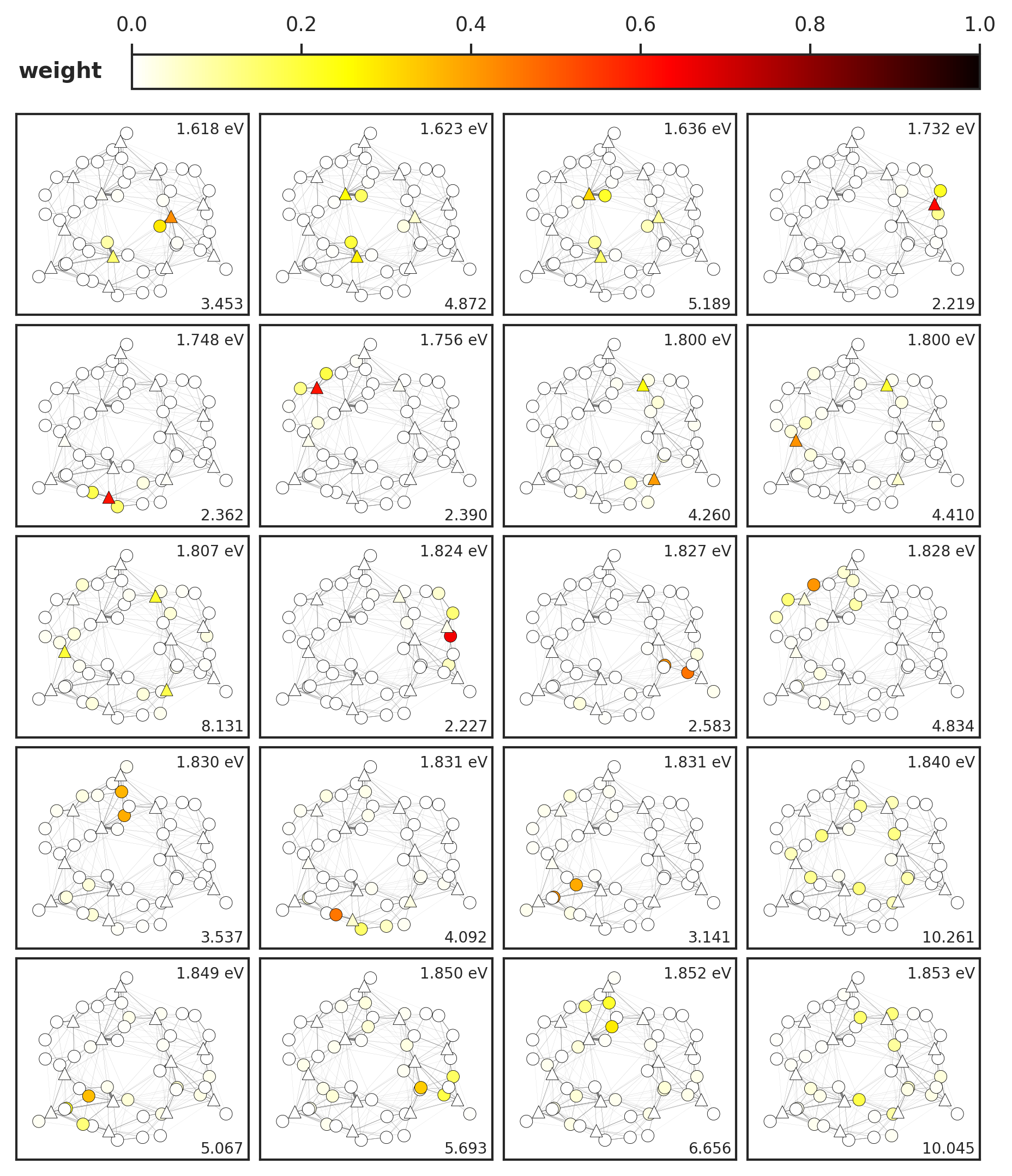

Supplement: Supplementary file 2 [file jp5c02465_si_002.zip › Fig6Analogues/LHCII/LHCII_ChlbreplacedbyChla_Q_part1.png]

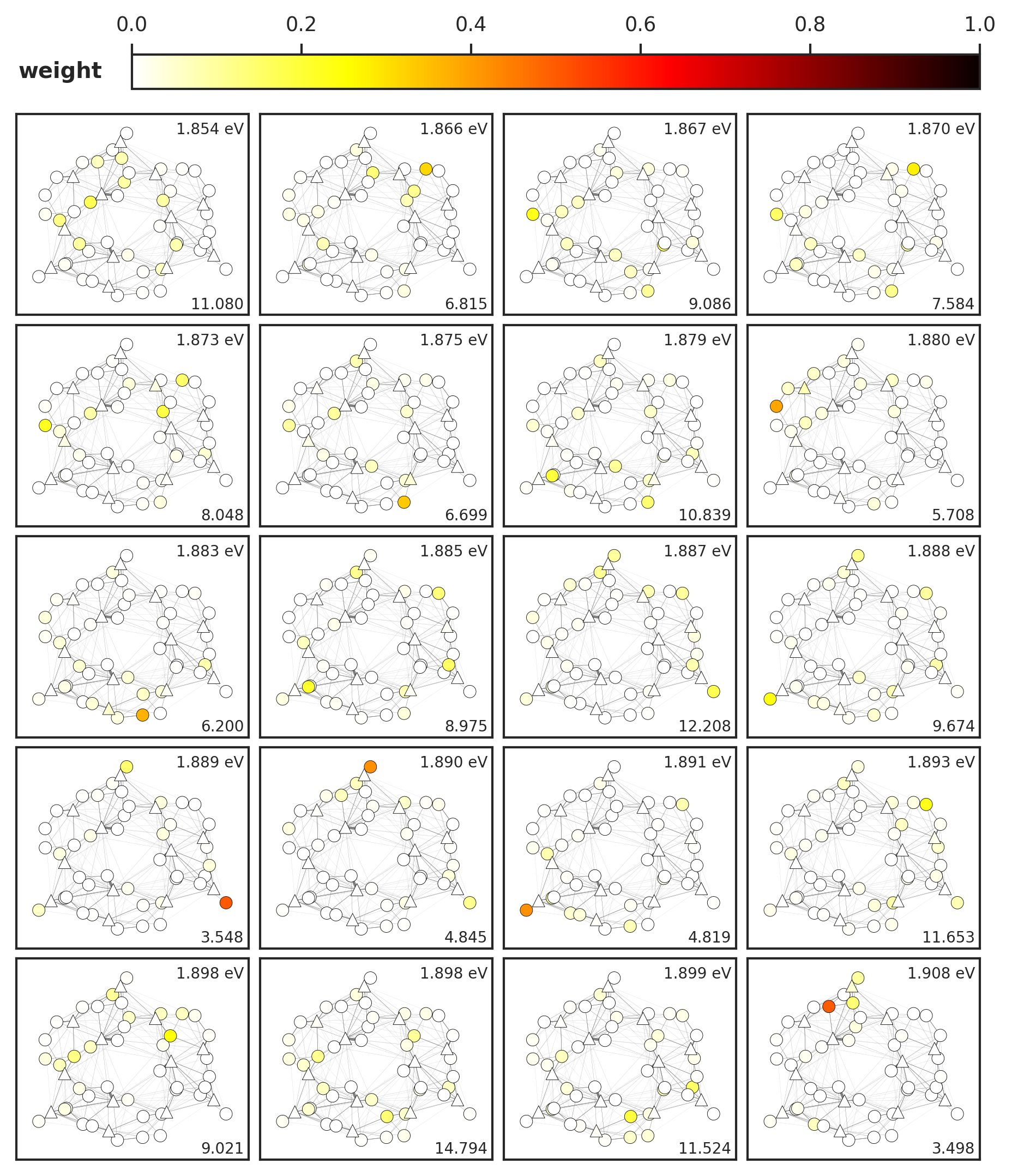

Supplement: Supplementary file 2 [file jp5c02465_si_002.zip › Fig6Analogues/LHCII/LHCII_ChlbreplacedbyChla_Q_part2.png]

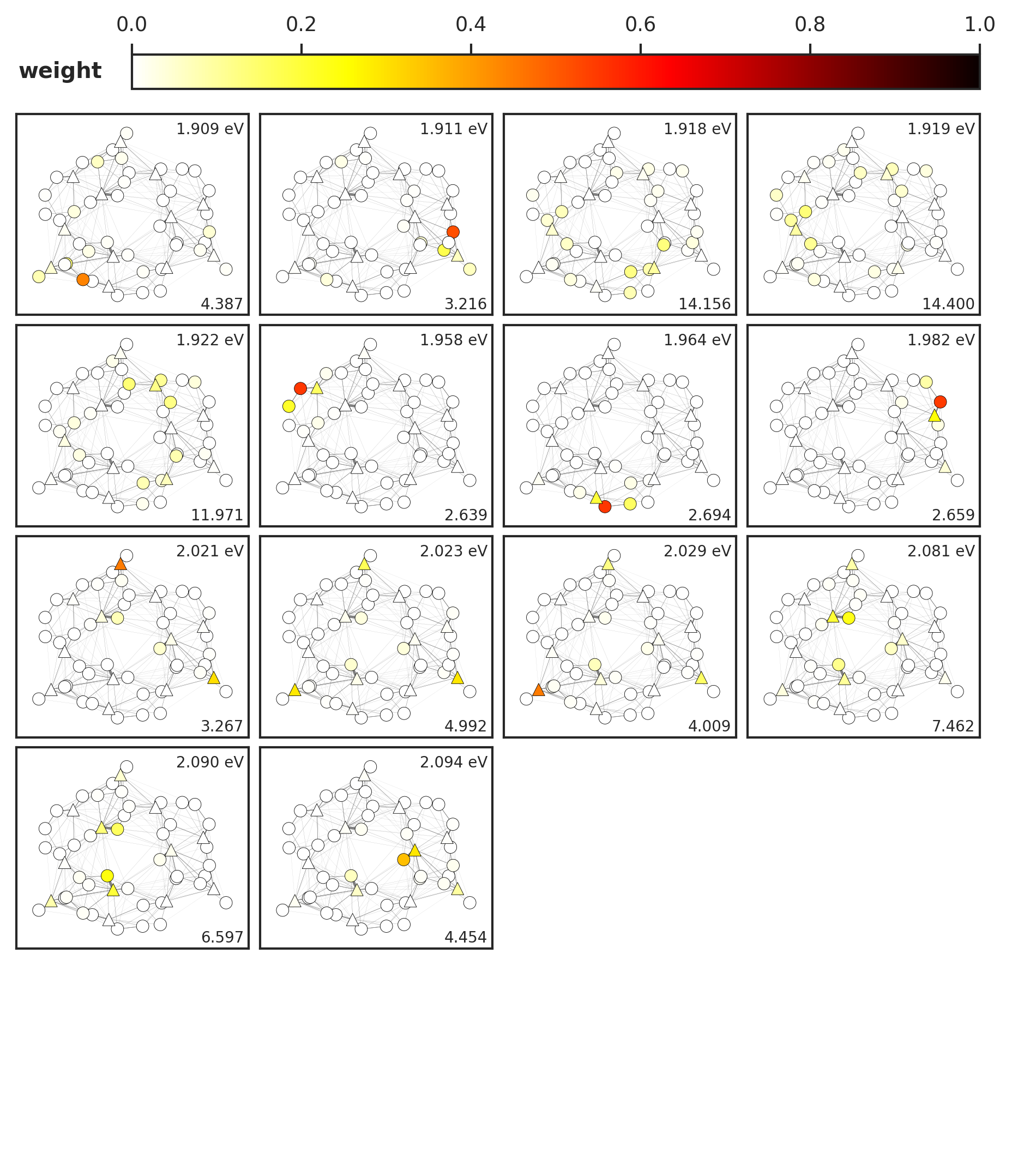

Supplement: Supplementary file 2 [file jp5c02465_si_002.zip › Fig6Analogues/LHCII/LHCII_ChlbreplacedbyChla_Q_part3.png]

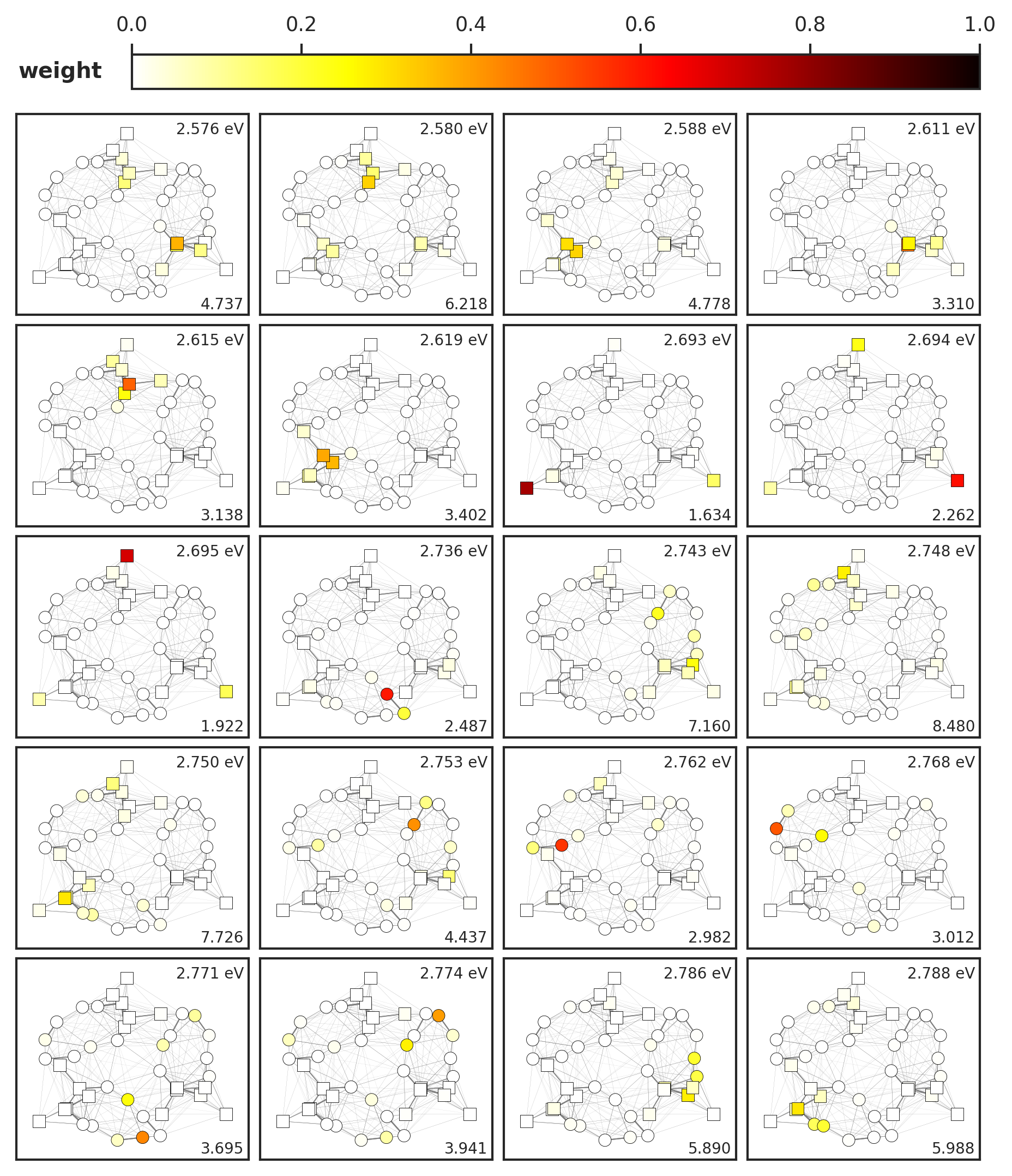

Supplement: Supplementary file 2 [file jp5c02465_si_002.zip › Fig6Analogues/LHCII/LHCII_noCrts_B_part1.png]

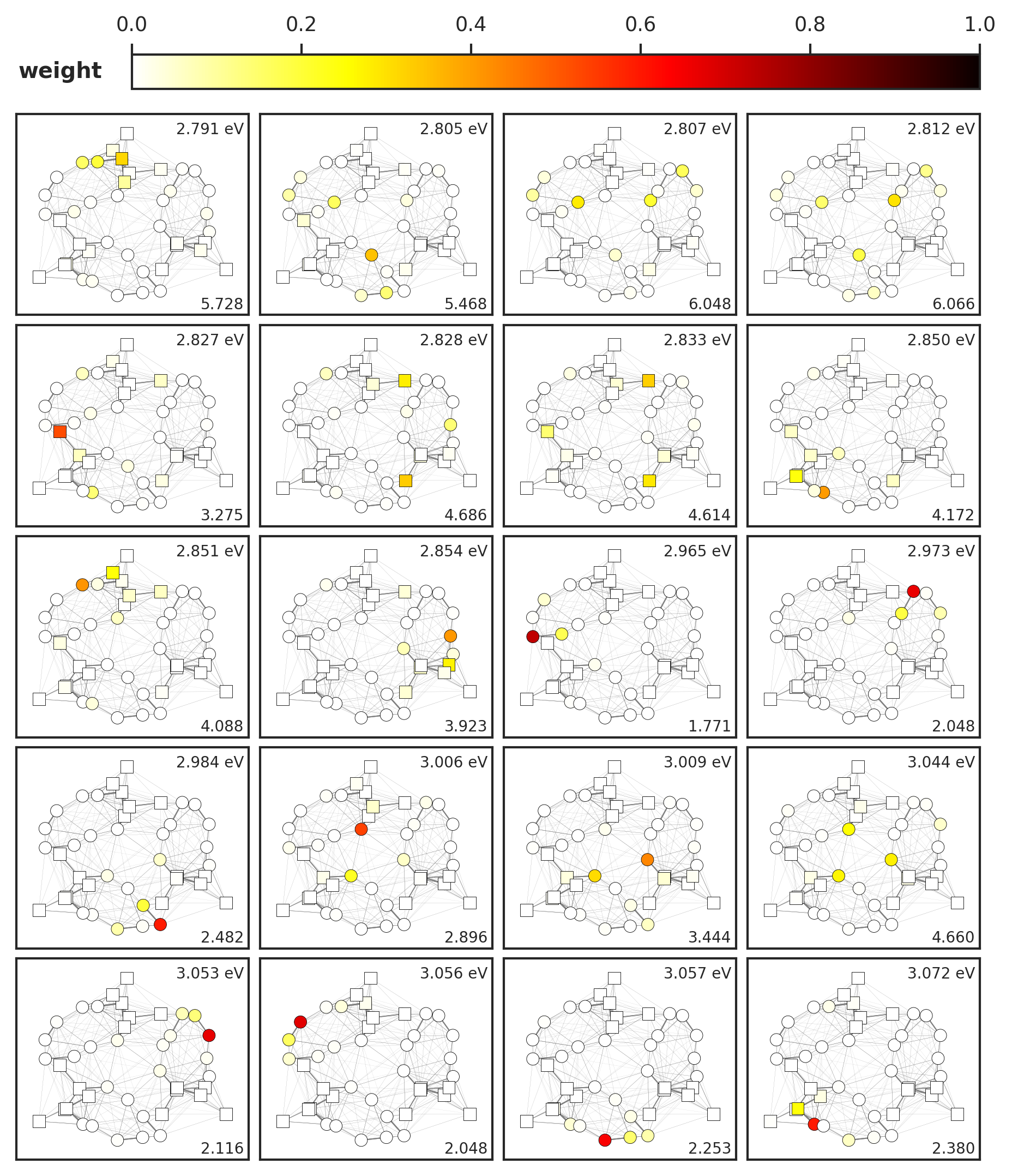

Supplement: Supplementary file 2 [file jp5c02465_si_002.zip › Fig6Analogues/LHCII/LHCII_noCrts_B_part2.png]

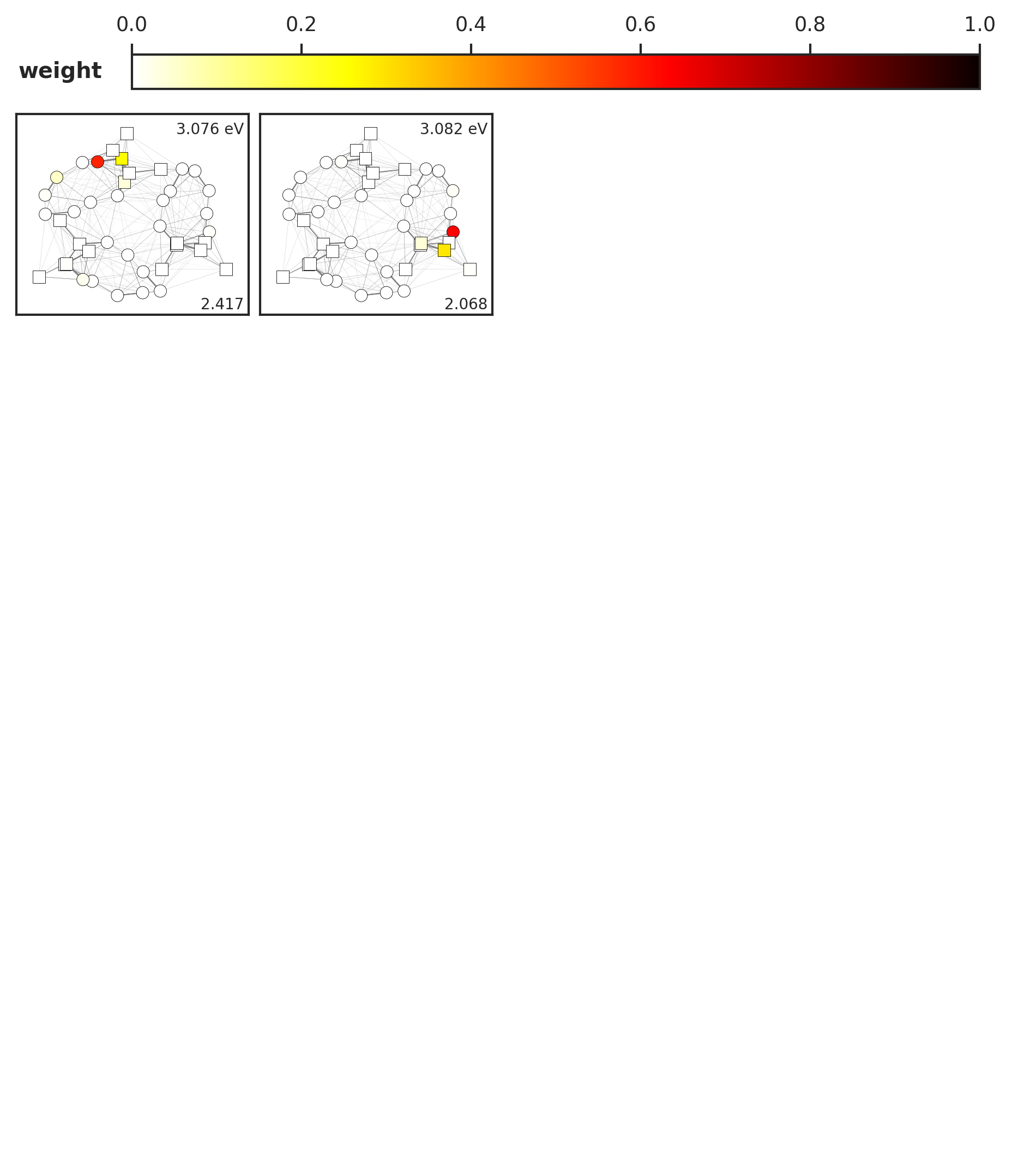

Supplement: Supplementary file 2 [file jp5c02465_si_002.zip › Fig6Analogues/LHCII/LHCII_noCrts_B_part3.png]

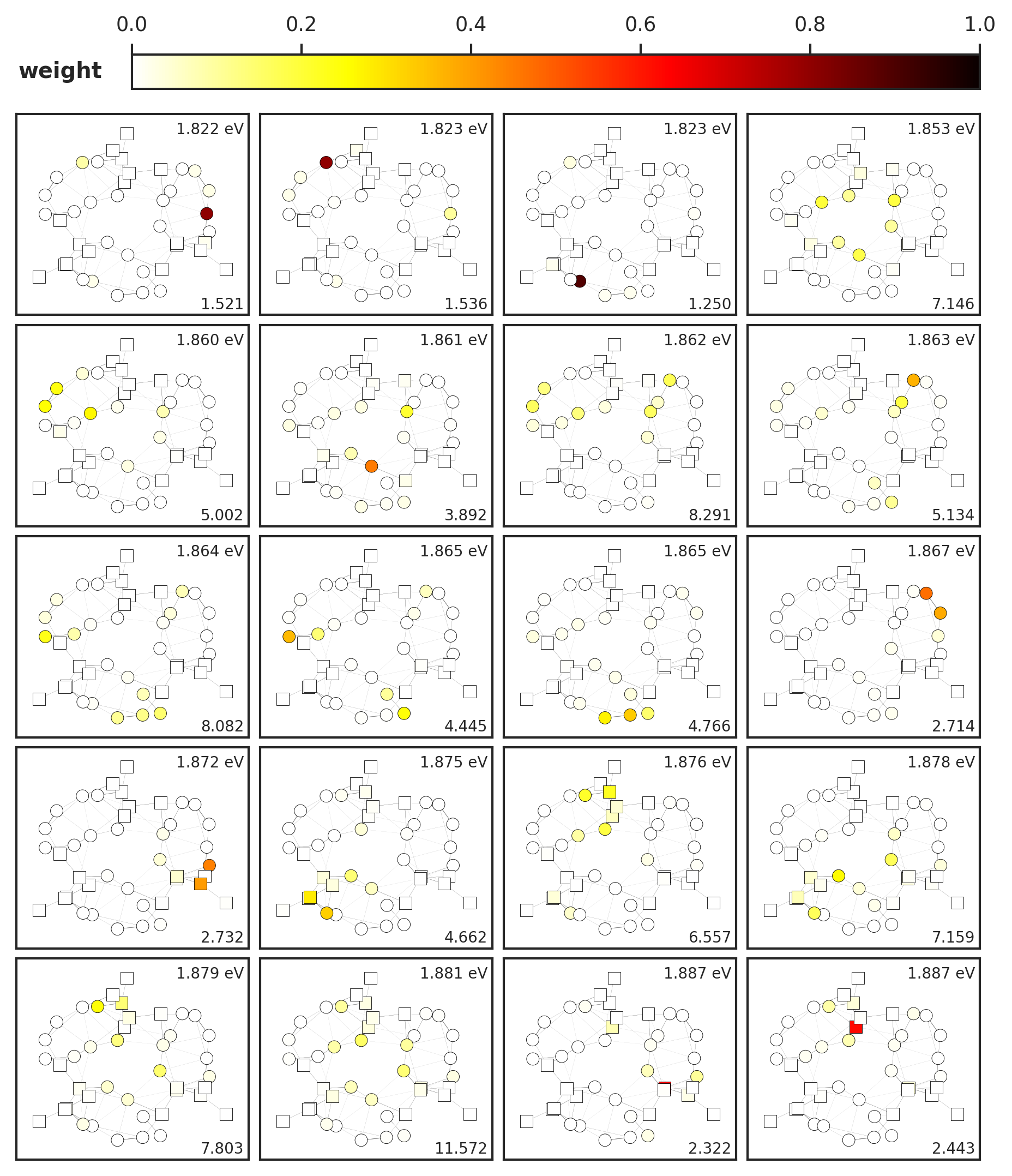

Supplement: Supplementary file 2 [file jp5c02465_si_002.zip › Fig6Analogues/LHCII/LHCII_noCrts_Q_part1.png]

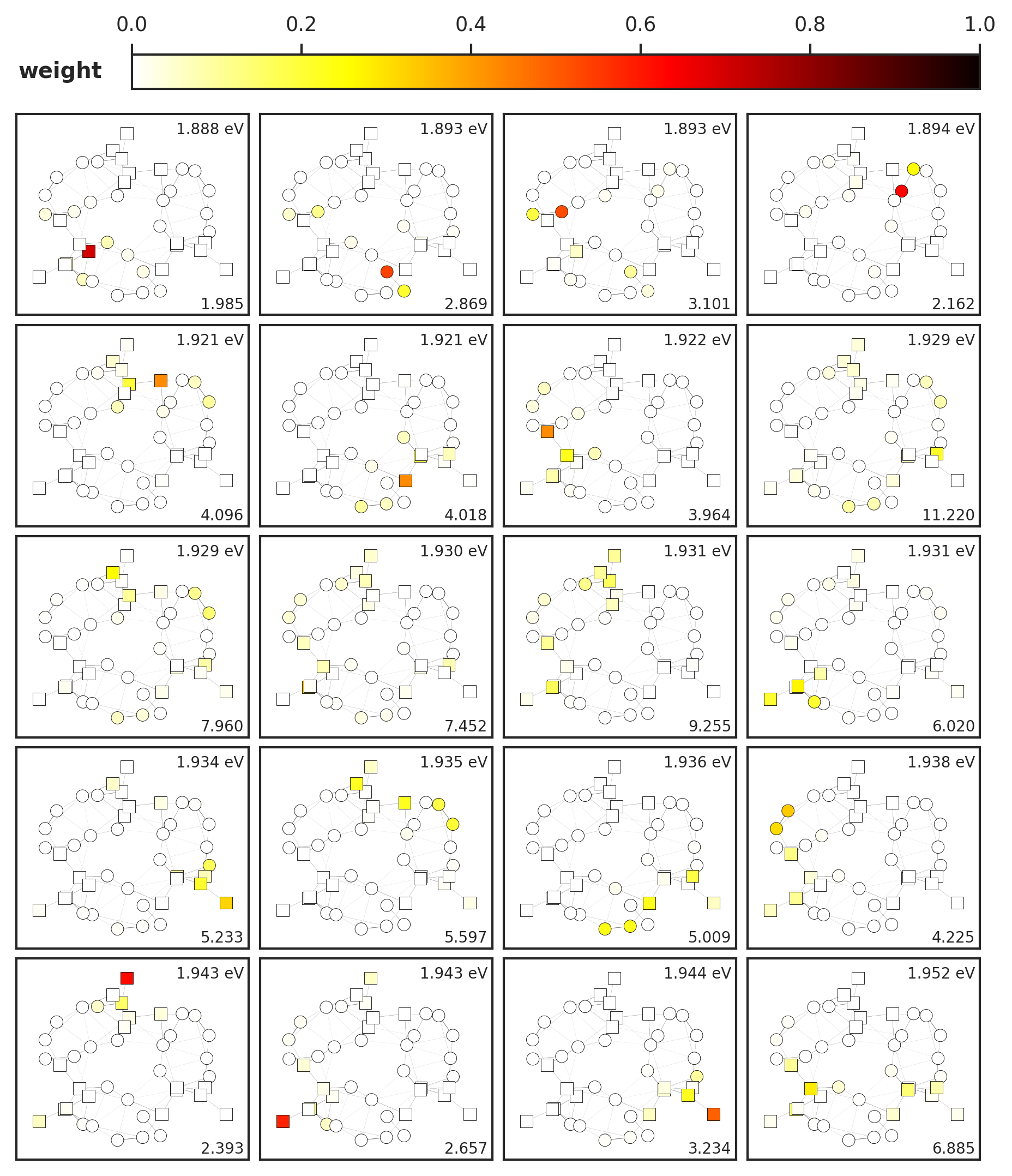

Supplement: Supplementary file 2 [file jp5c02465_si_002.zip › Fig6Analogues/LHCII/LHCII_noCrts_Q_part2.png]

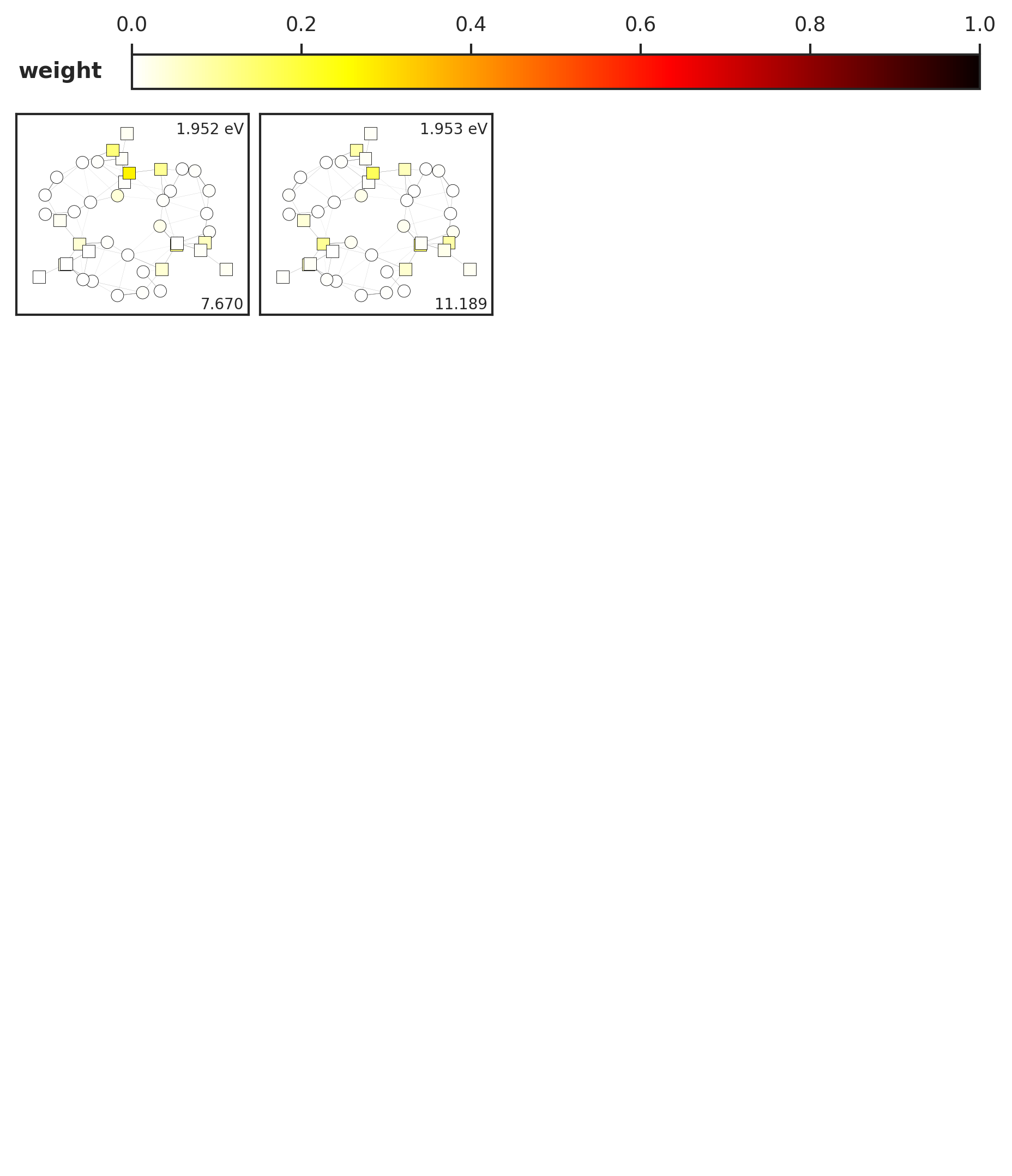

Supplement: Supplementary file 2 [file jp5c02465_si_002.zip › Fig6Analogues/LHCII/LHCII_noCrts_Q_part3.png]

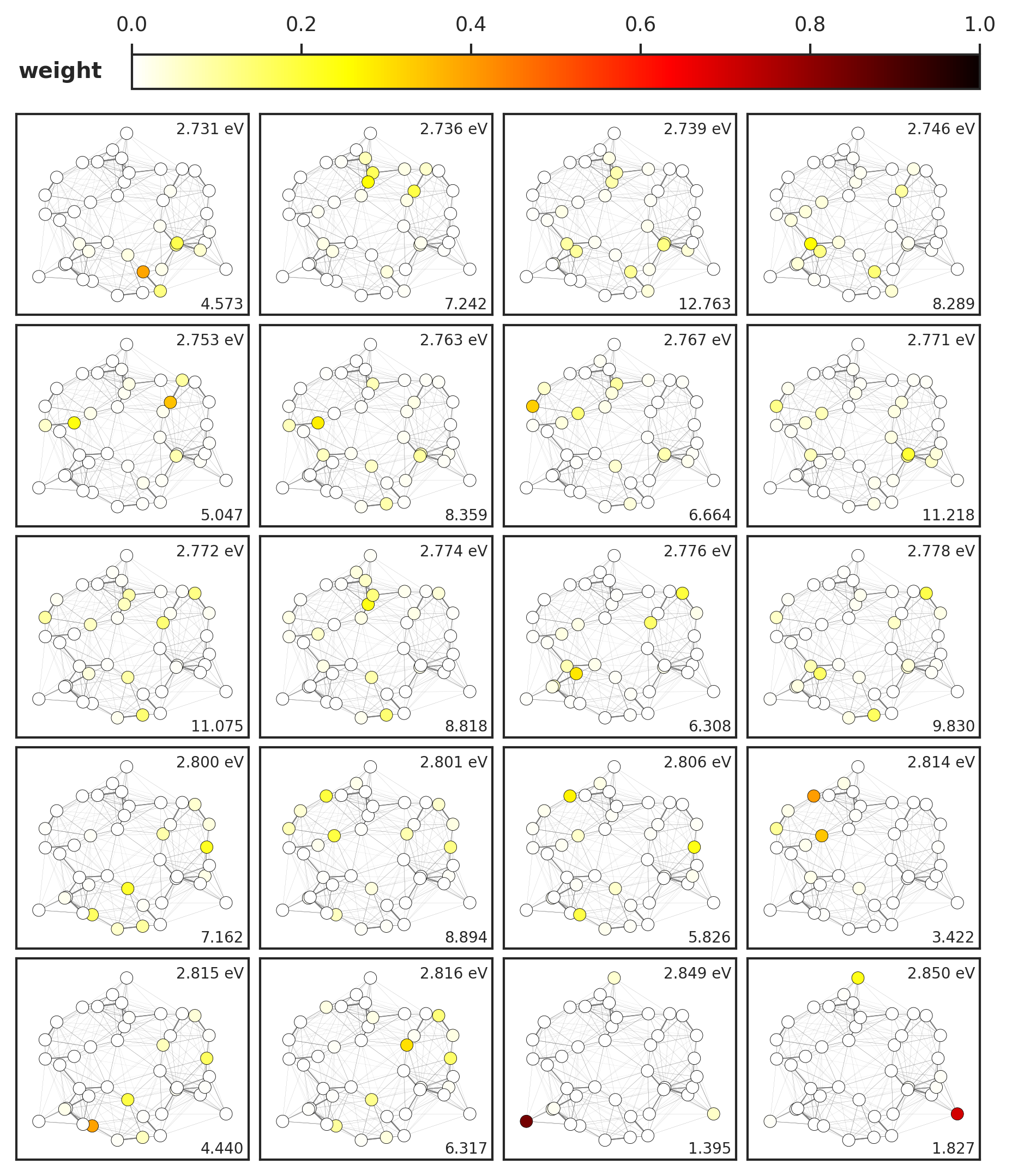

Supplement: Supplementary file 2 [file jp5c02465_si_002.zip › Fig6Analogues/LHCII/LHCII_onlyChla_B_part1.png]

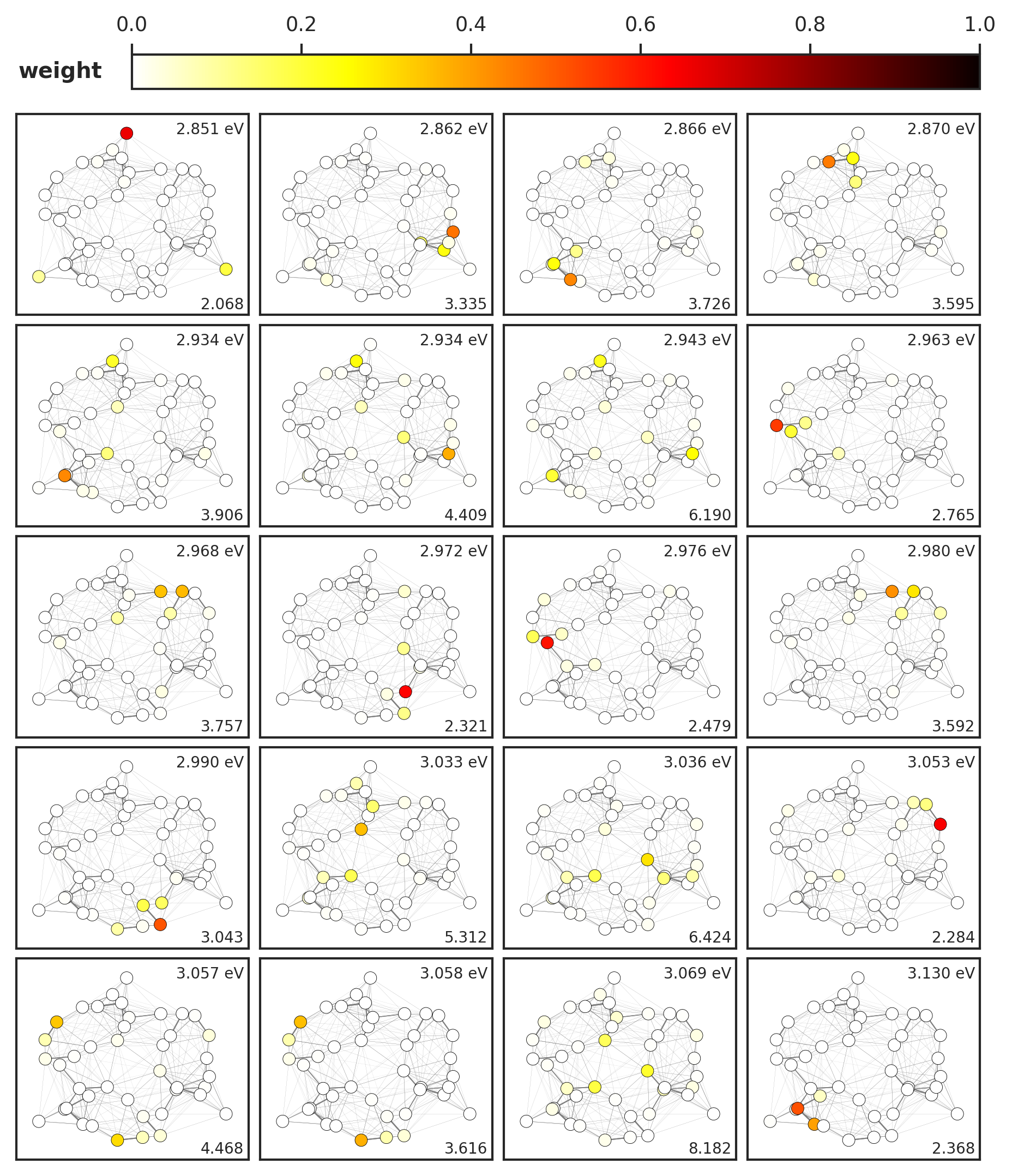

Supplement: Supplementary file 2 [file jp5c02465_si_002.zip › Fig6Analogues/LHCII/LHCII_onlyChla_B_part2.png]

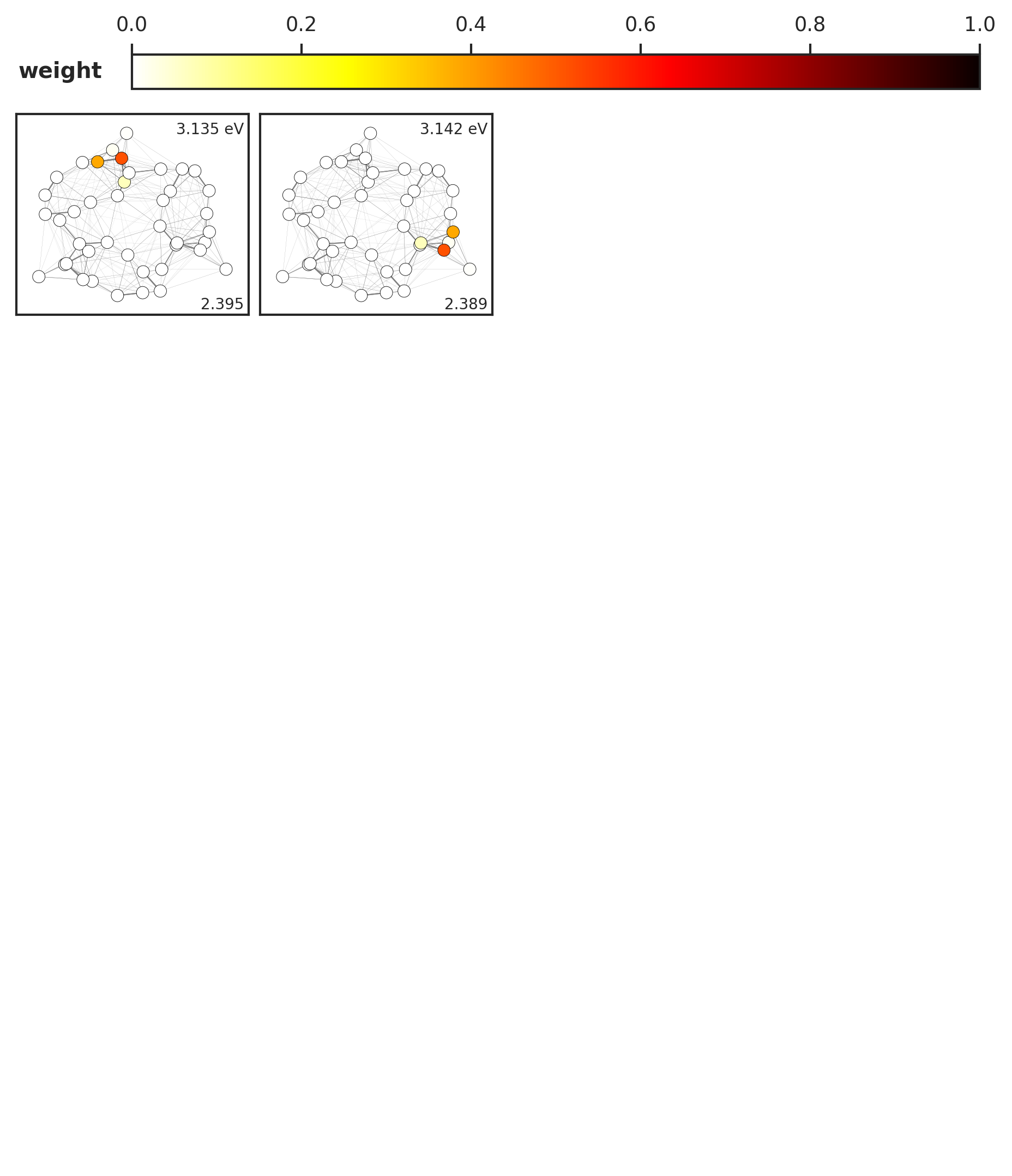

Supplement: Supplementary file 2 [file jp5c02465_si_002.zip › Fig6Analogues/LHCII/LHCII_onlyChla_B_part3.png]

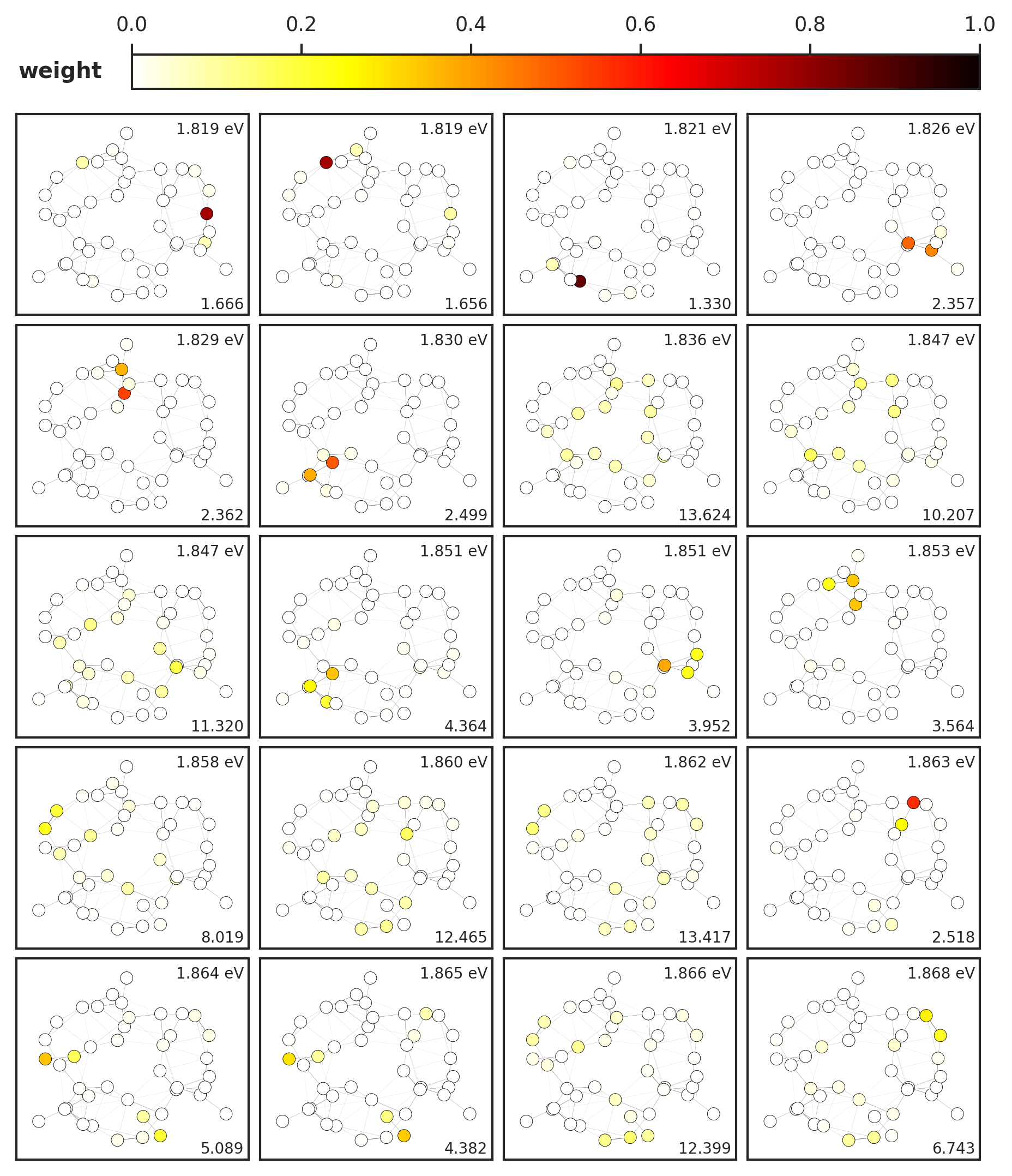

Supplement: Supplementary file 2 [file jp5c02465_si_002.zip › Fig6Analogues/LHCII/LHCII_onlyChla_Q_part1.png]

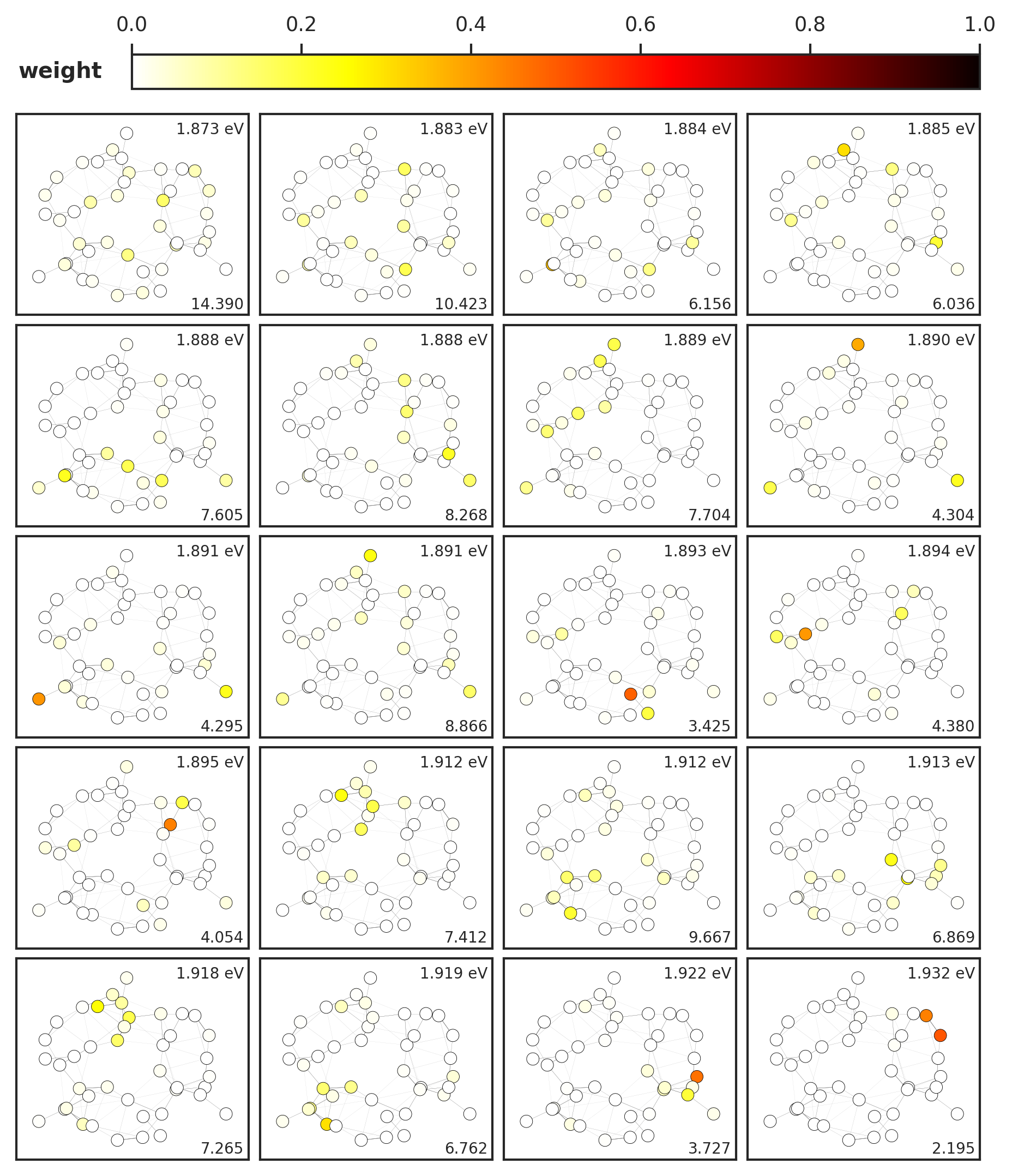

Supplement: Supplementary file 2 [file jp5c02465_si_002.zip › Fig6Analogues/LHCII/LHCII_onlyChla_Q_part2.png]

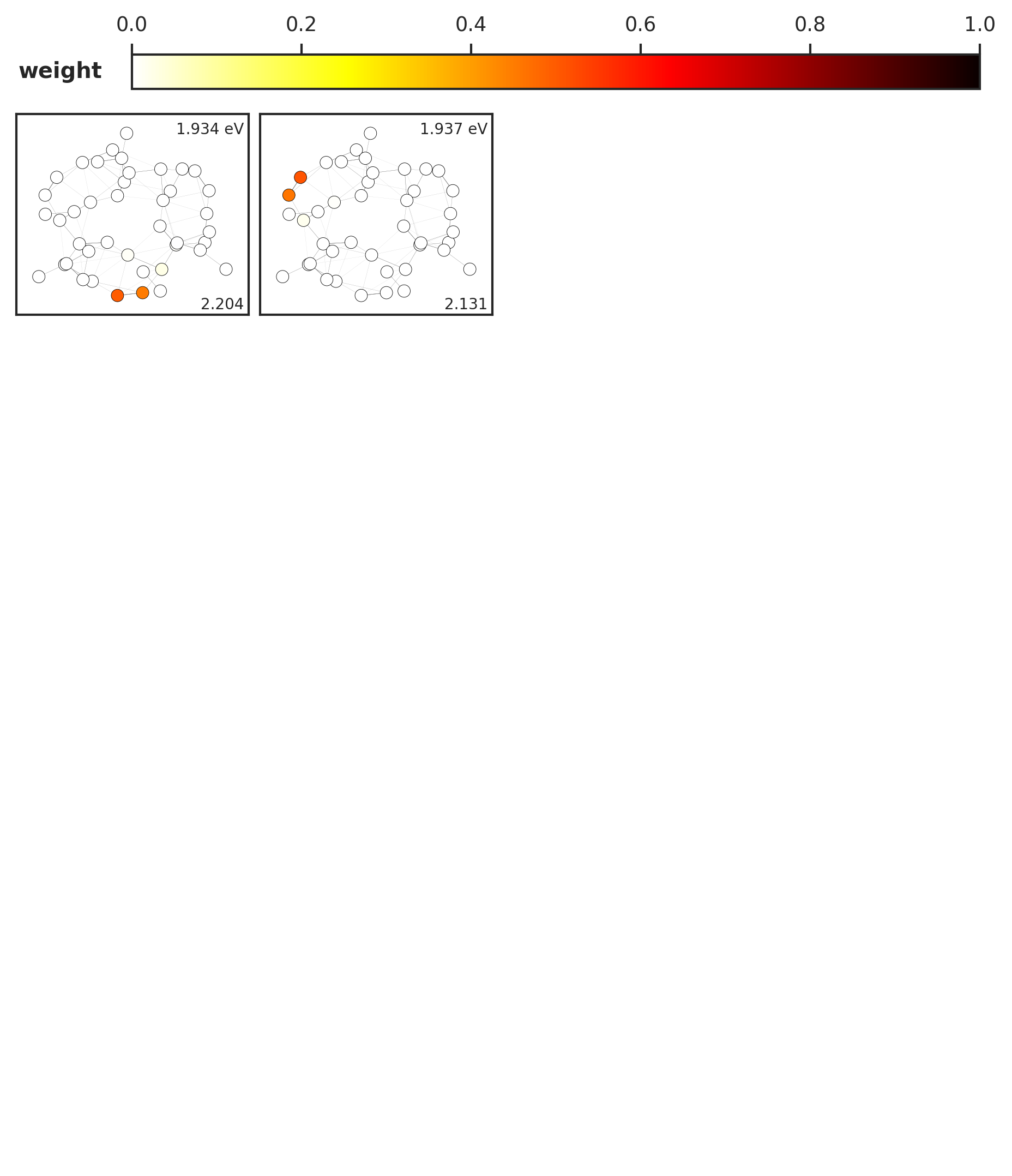

Supplement: Supplementary file 2 [file jp5c02465_si_002.zip › Fig6Analogues/LHCII/LHCII_onlyChla_Q_part3.png]

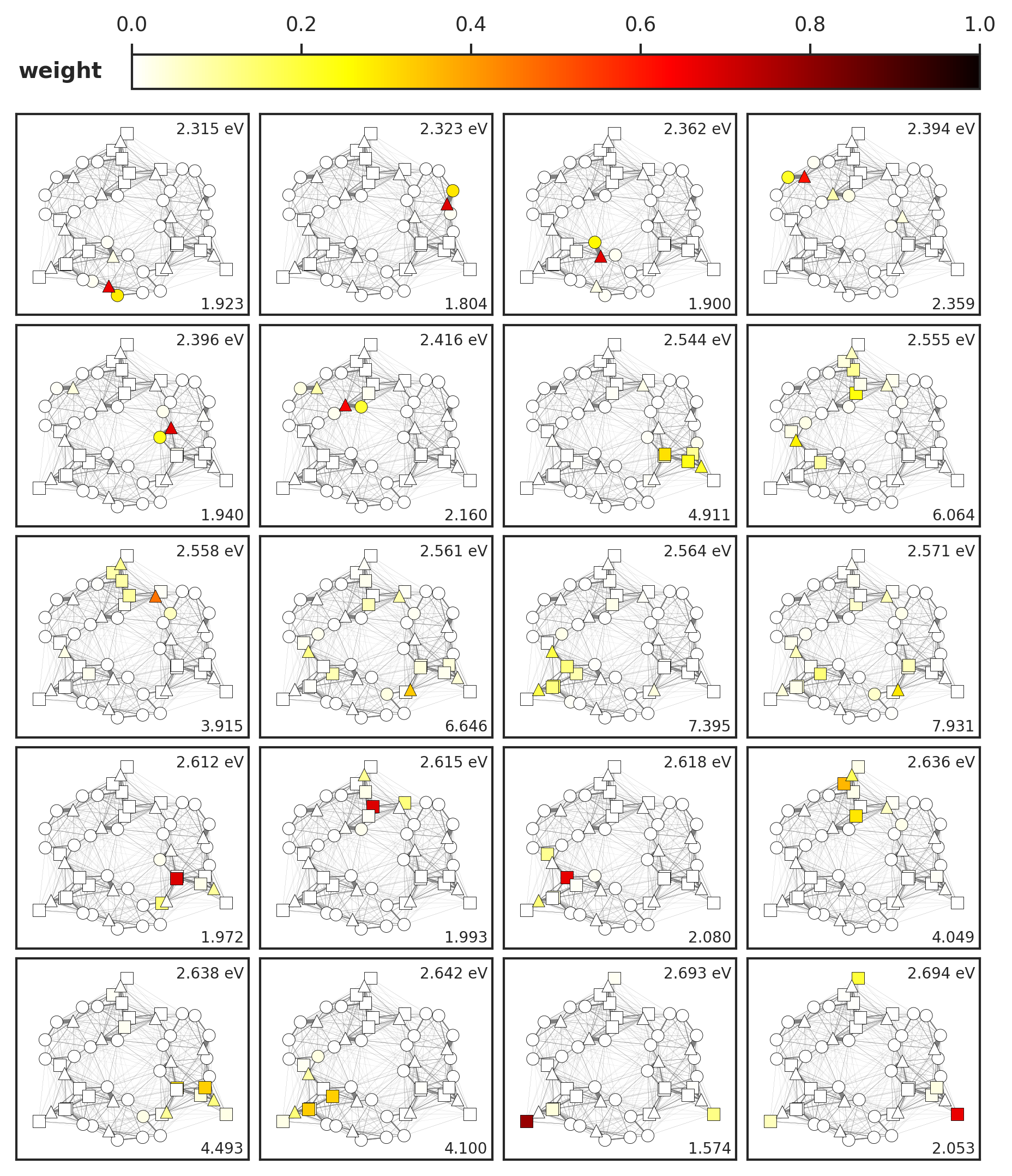

Supplement: Supplementary file 2 [file jp5c02465_si_002.zip › Fig6Analogues/LHCII/LHCII_WT_B_part1.png]

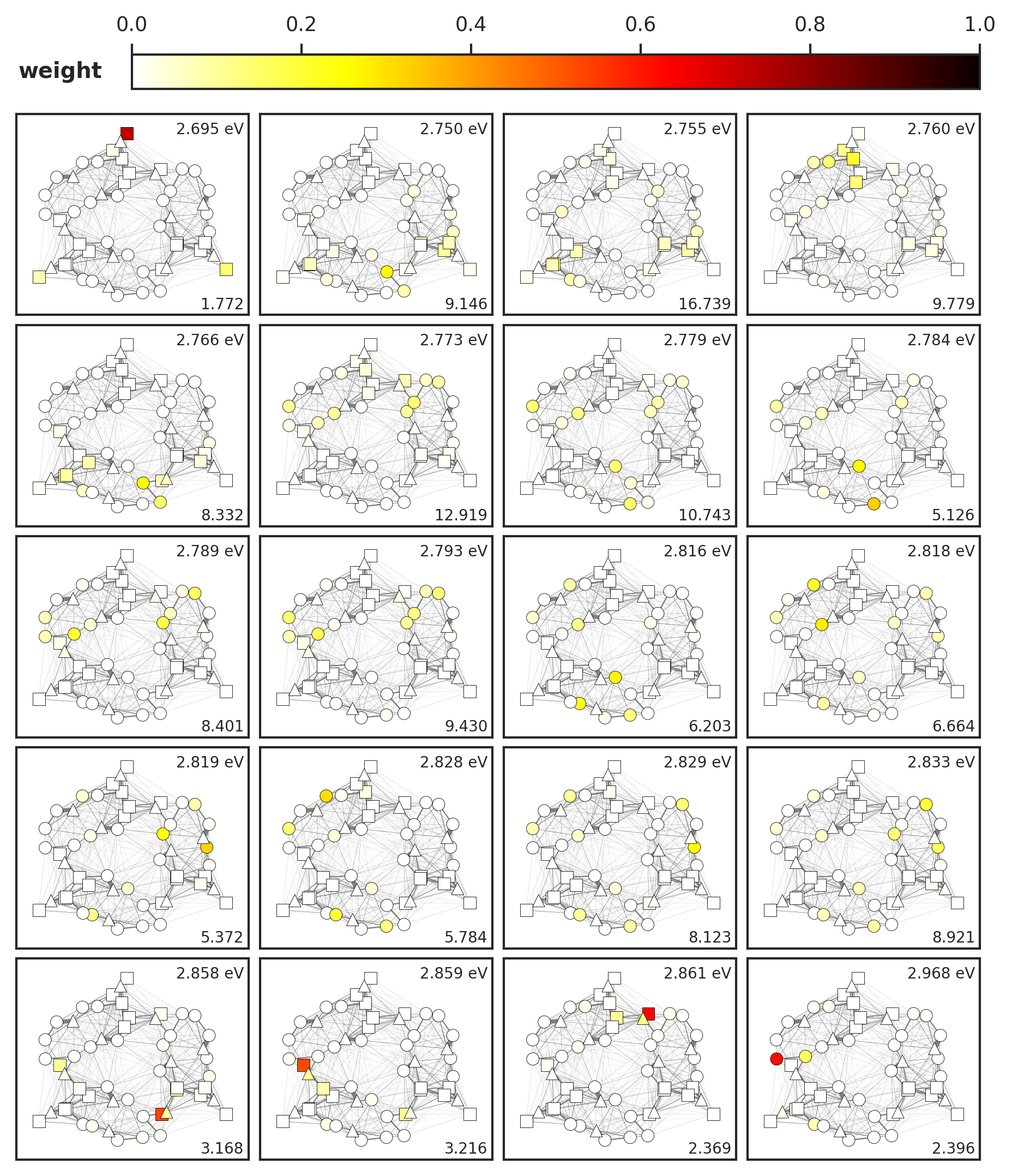

Supplement: Supplementary file 2 [file jp5c02465_si_002.zip › Fig6Analogues/LHCII/LHCII_WT_B_part2.png]

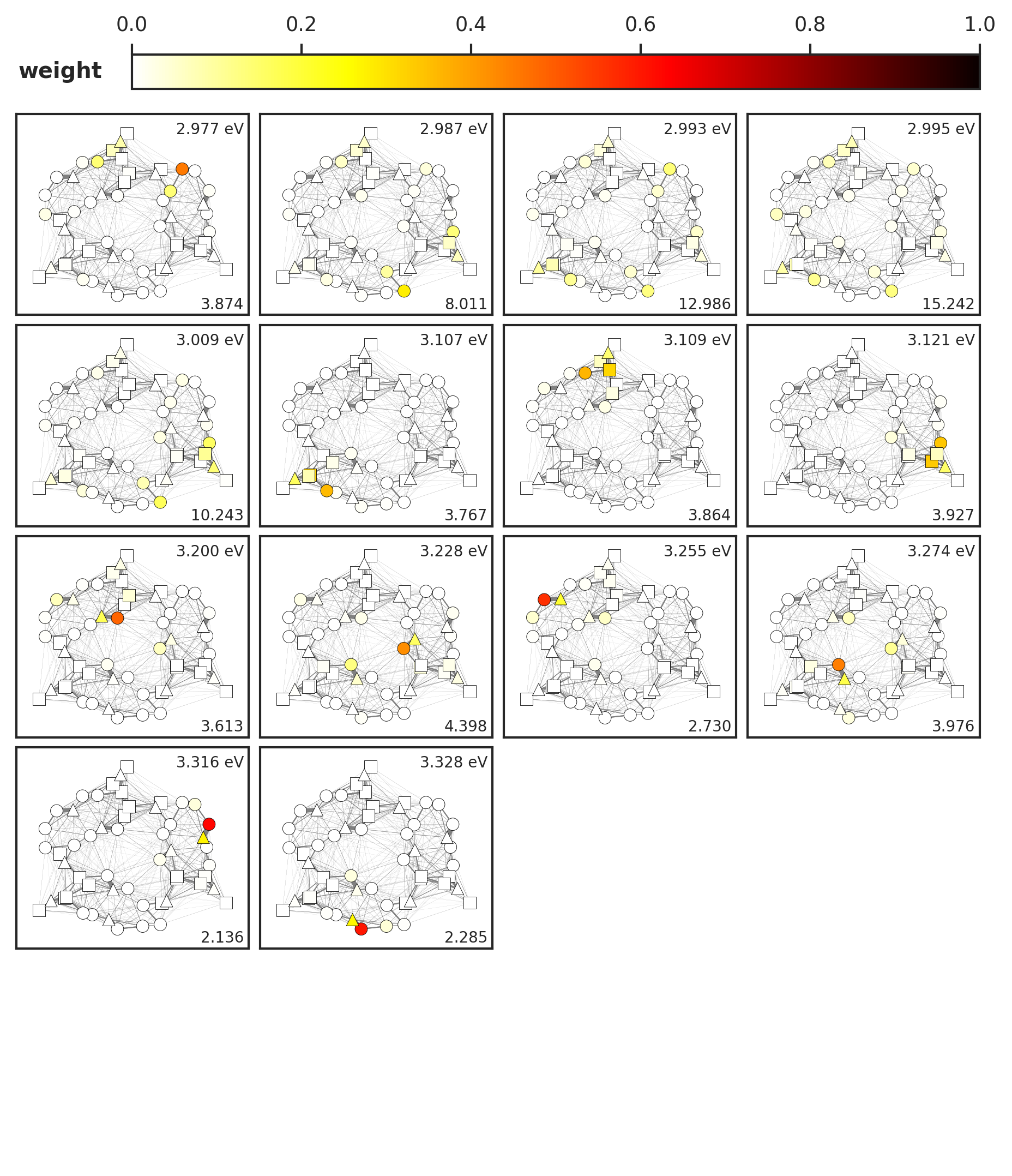

Supplement: Supplementary file 2 [file jp5c02465_si_002.zip › Fig6Analogues/LHCII/LHCII_WT_B_part3.png]

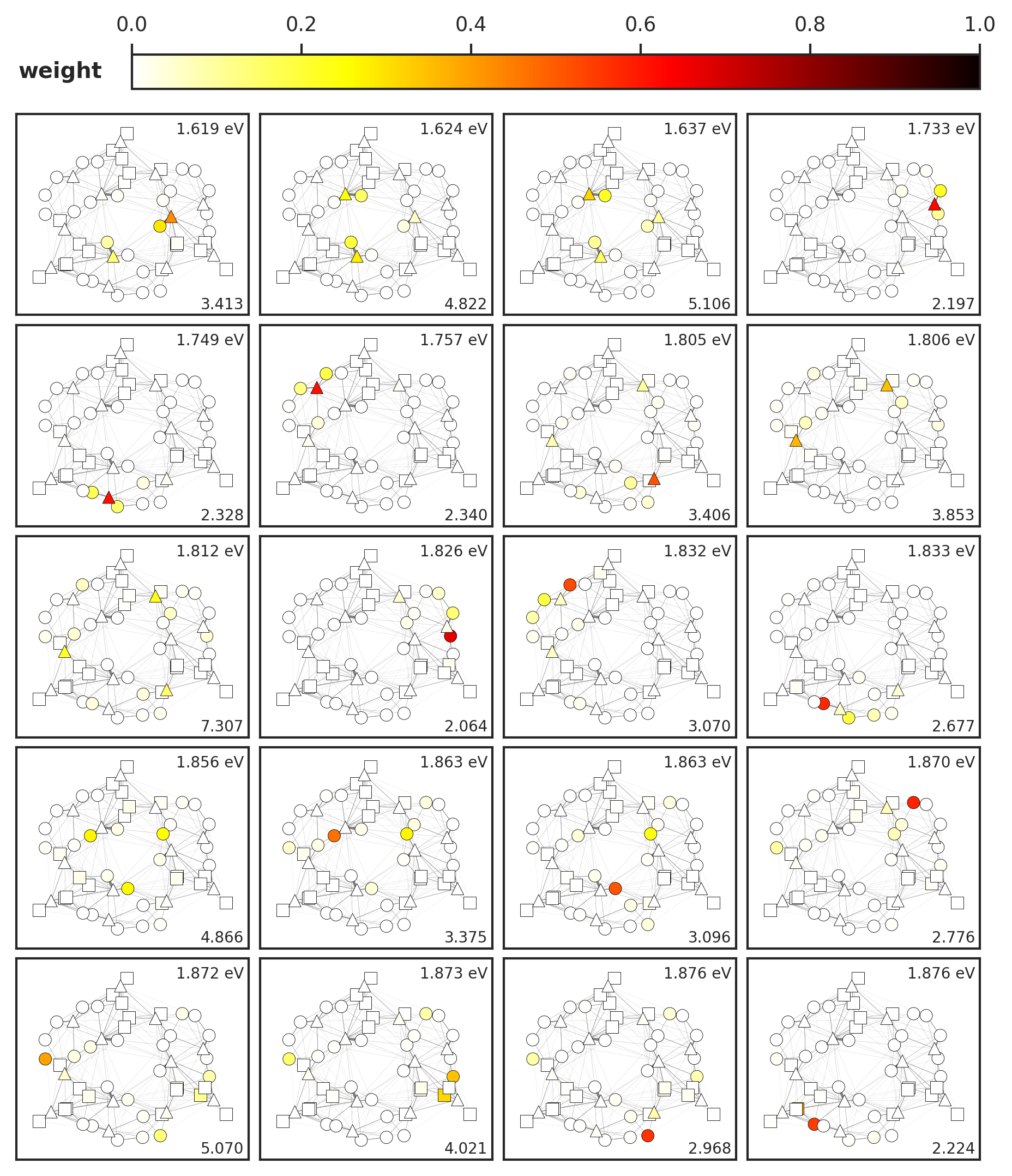

Supplement: Supplementary file 2 [file jp5c02465_si_002.zip › Fig6Analogues/LHCII/LHCII_WT_Q_part1.png]

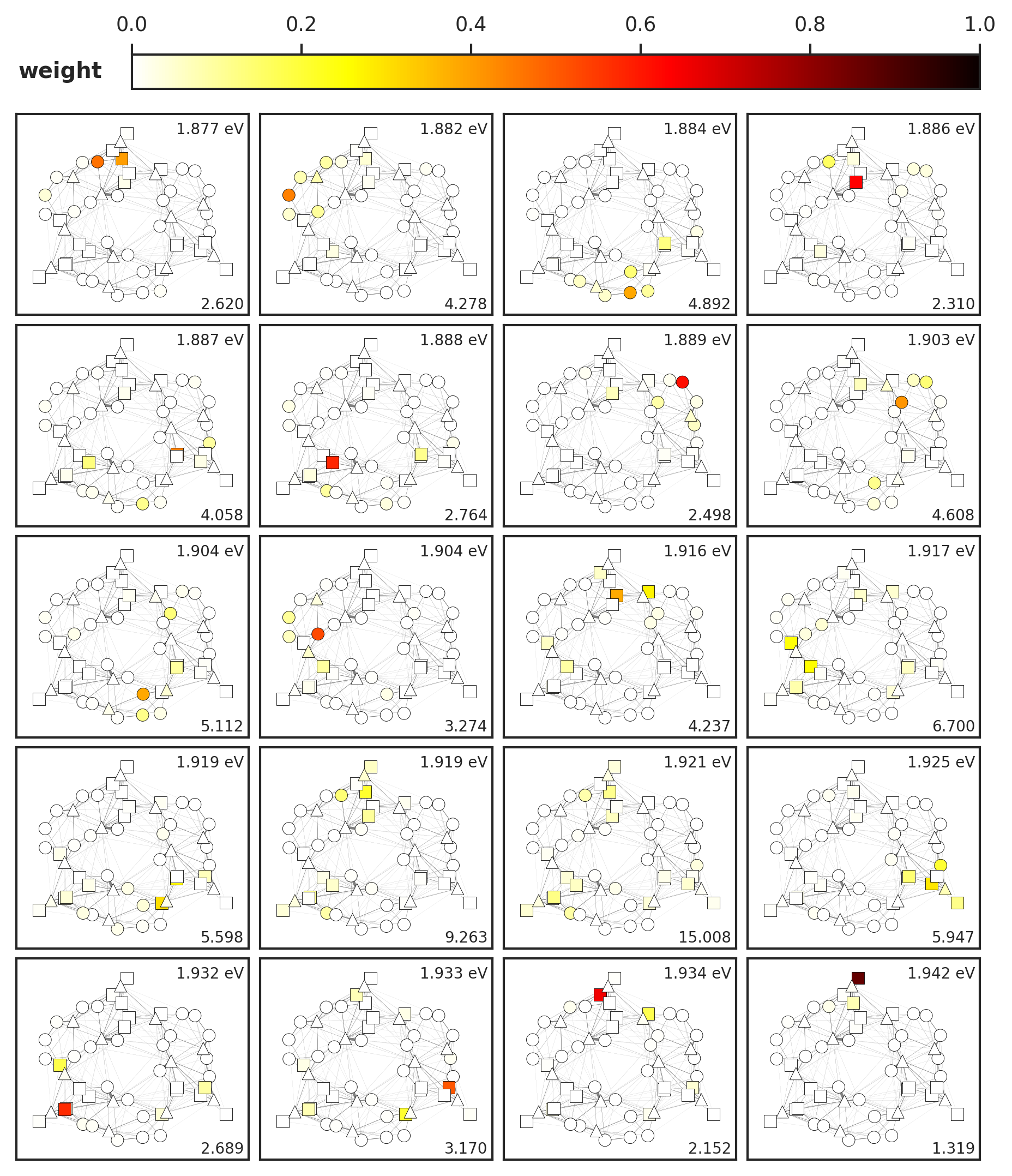

Supplement: Supplementary file 2 [file jp5c02465_si_002.zip › Fig6Analogues/LHCII/LHCII_WT_Q_part2.png]

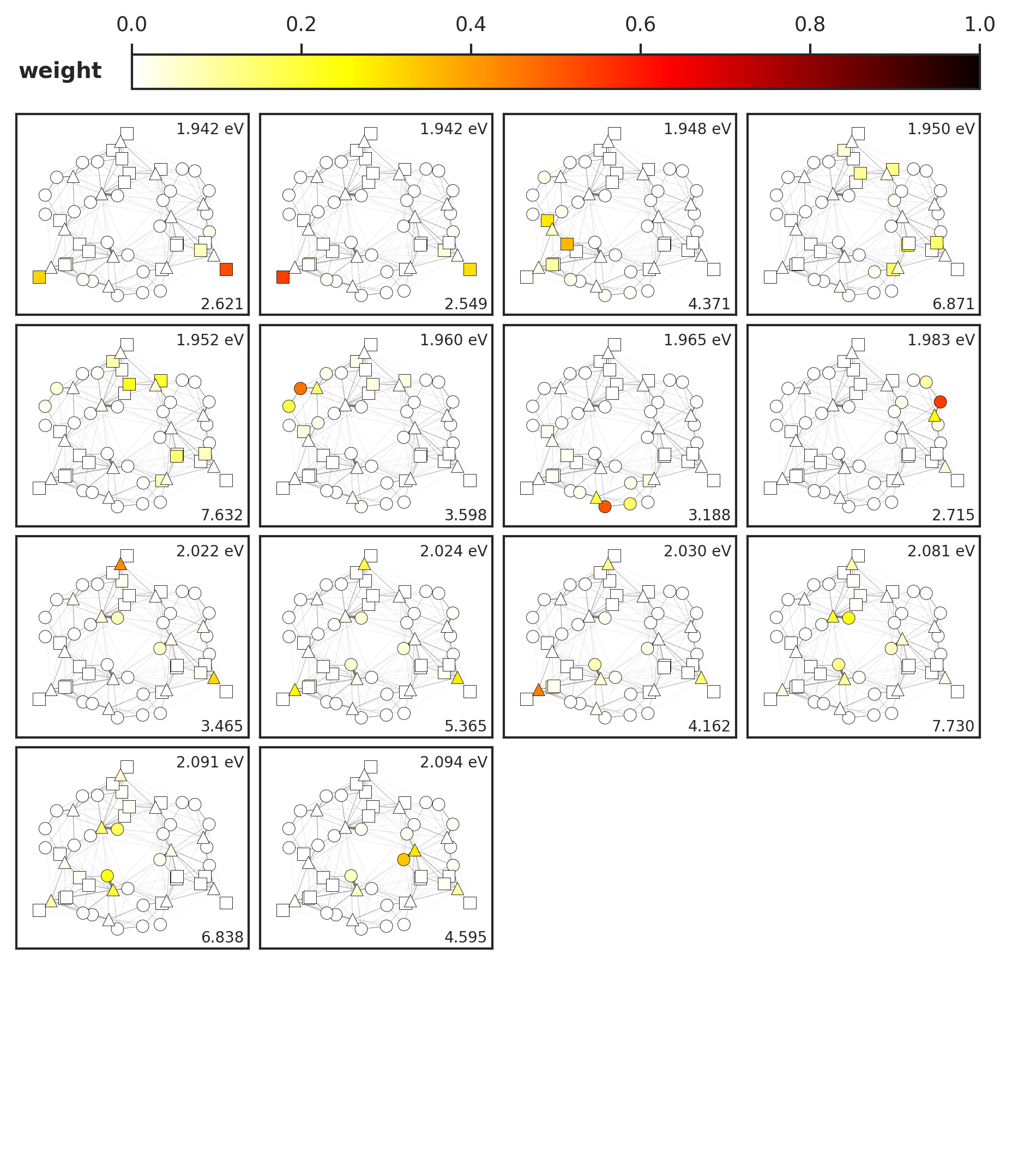

Supplement: Supplementary file 2 [file jp5c02465_si_002.zip › Fig6Analogues/LHCII/LHCII_WT_Q_part3.png]

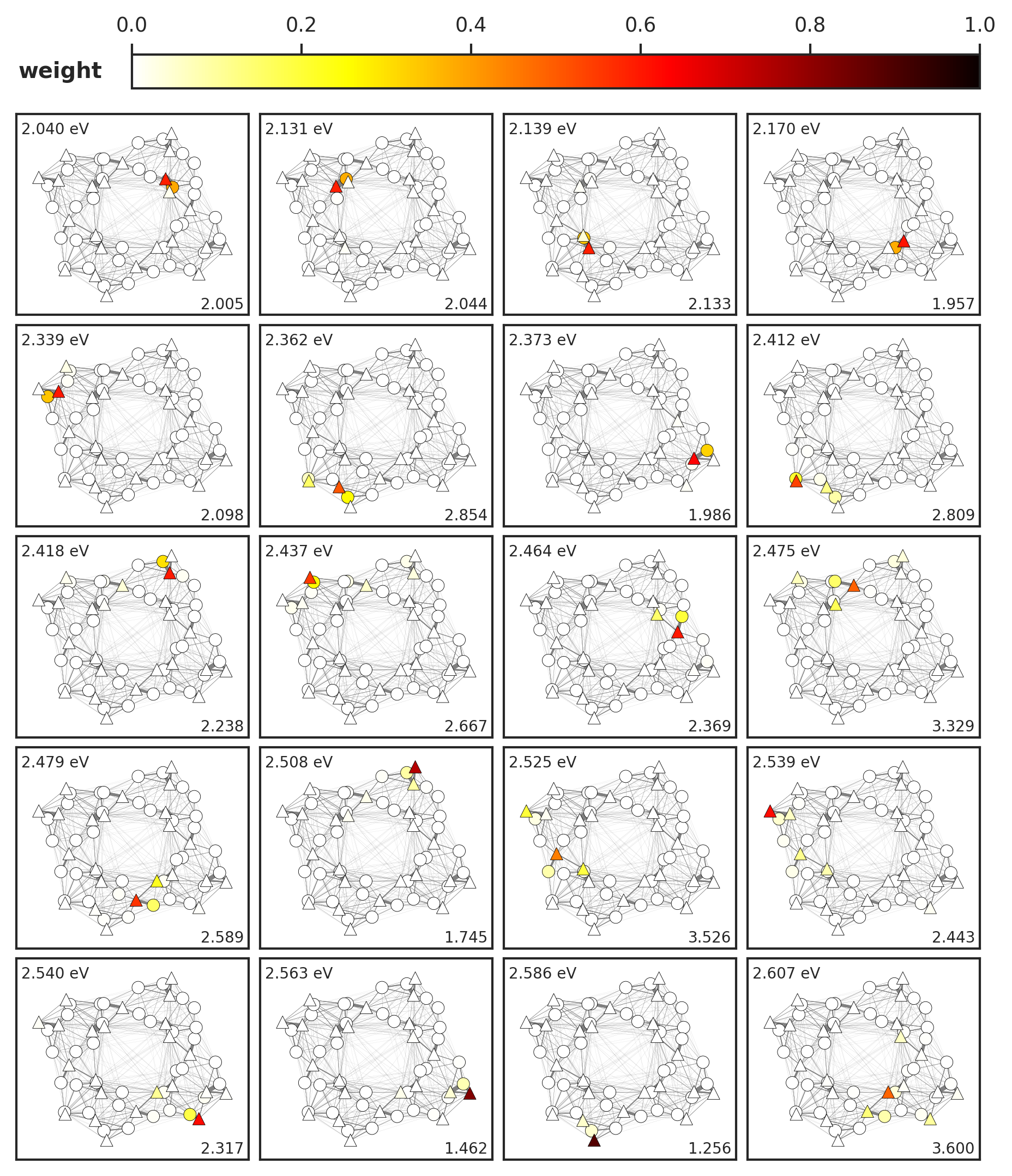

Supplement: Supplementary file 2 [file jp5c02465_si_002.zip › Fig6Analogues/tFCP/tFCP_ChlbreplacedbyChla_B_part1.png]

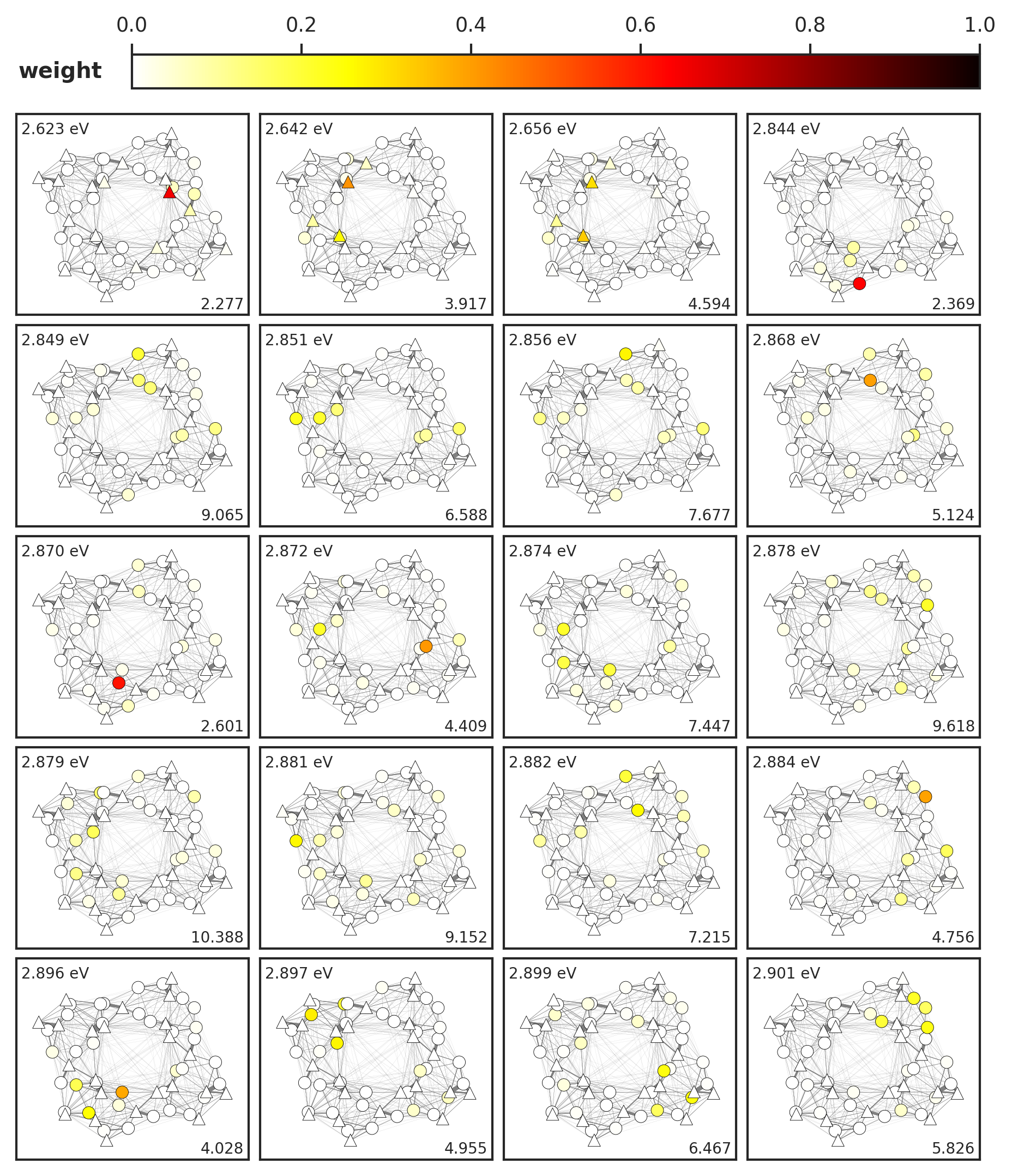

Supplement: Supplementary file 2 [file jp5c02465_si_002.zip › Fig6Analogues/tFCP/tFCP_ChlbreplacedbyChla_B_part2.png]

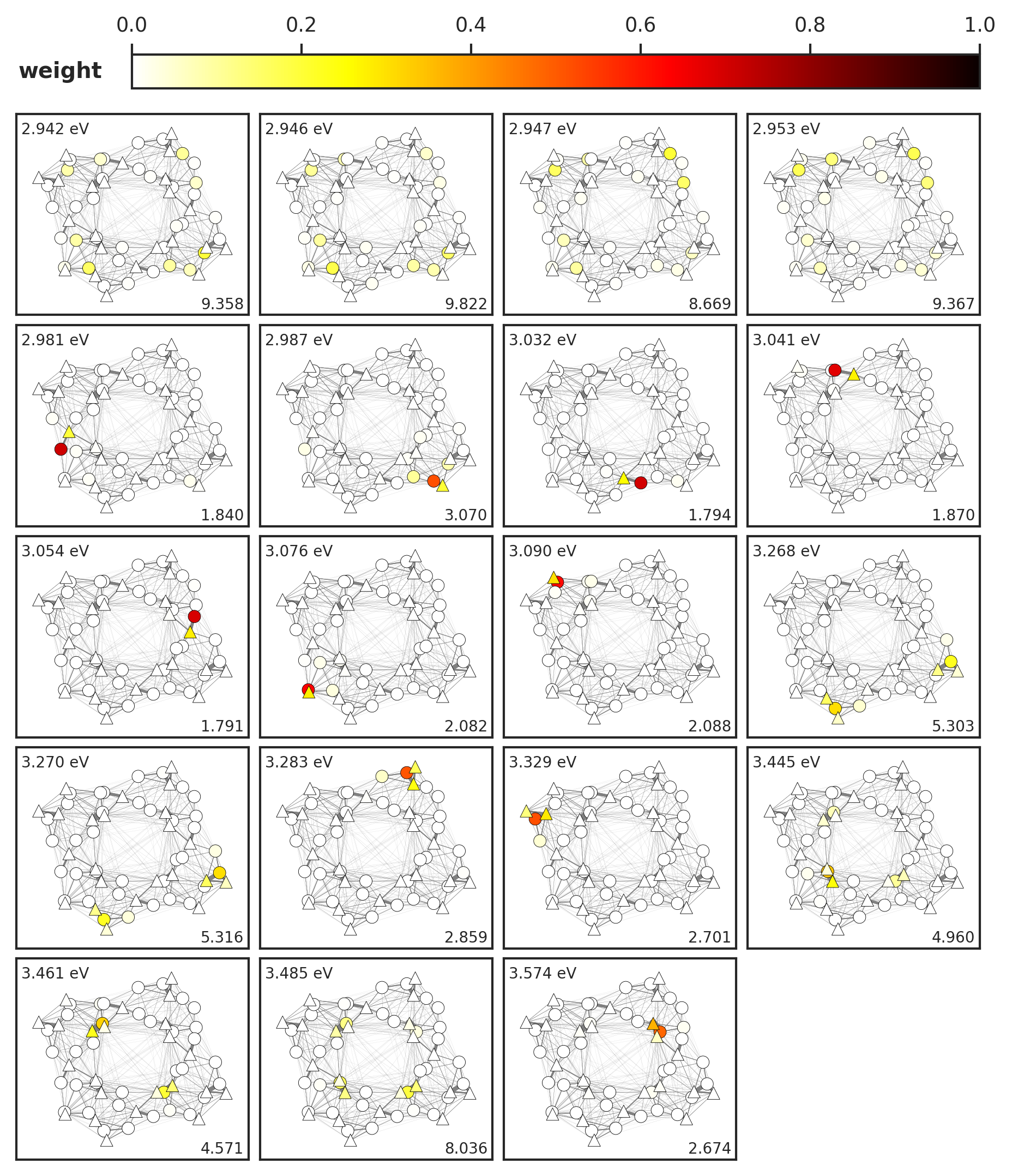

Supplement: Supplementary file 2 [file jp5c02465_si_002.zip › Fig6Analogues/tFCP/tFCP_ChlbreplacedbyChla_B_part3.png]

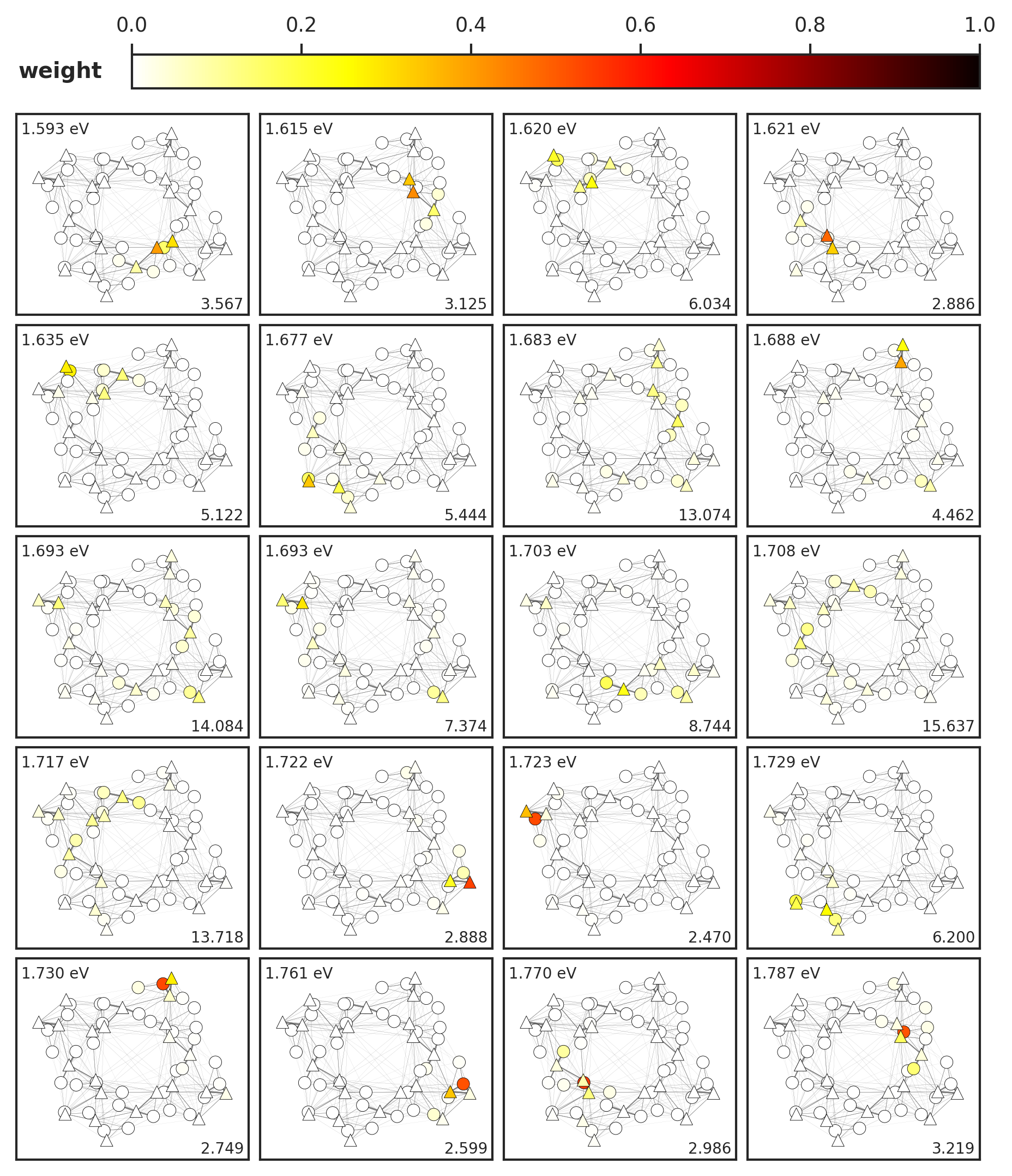

Supplement: Supplementary file 2 [file jp5c02465_si_002.zip › Fig6Analogues/tFCP/tFCP_ChlbreplacedbyChla_Q_part1.png]

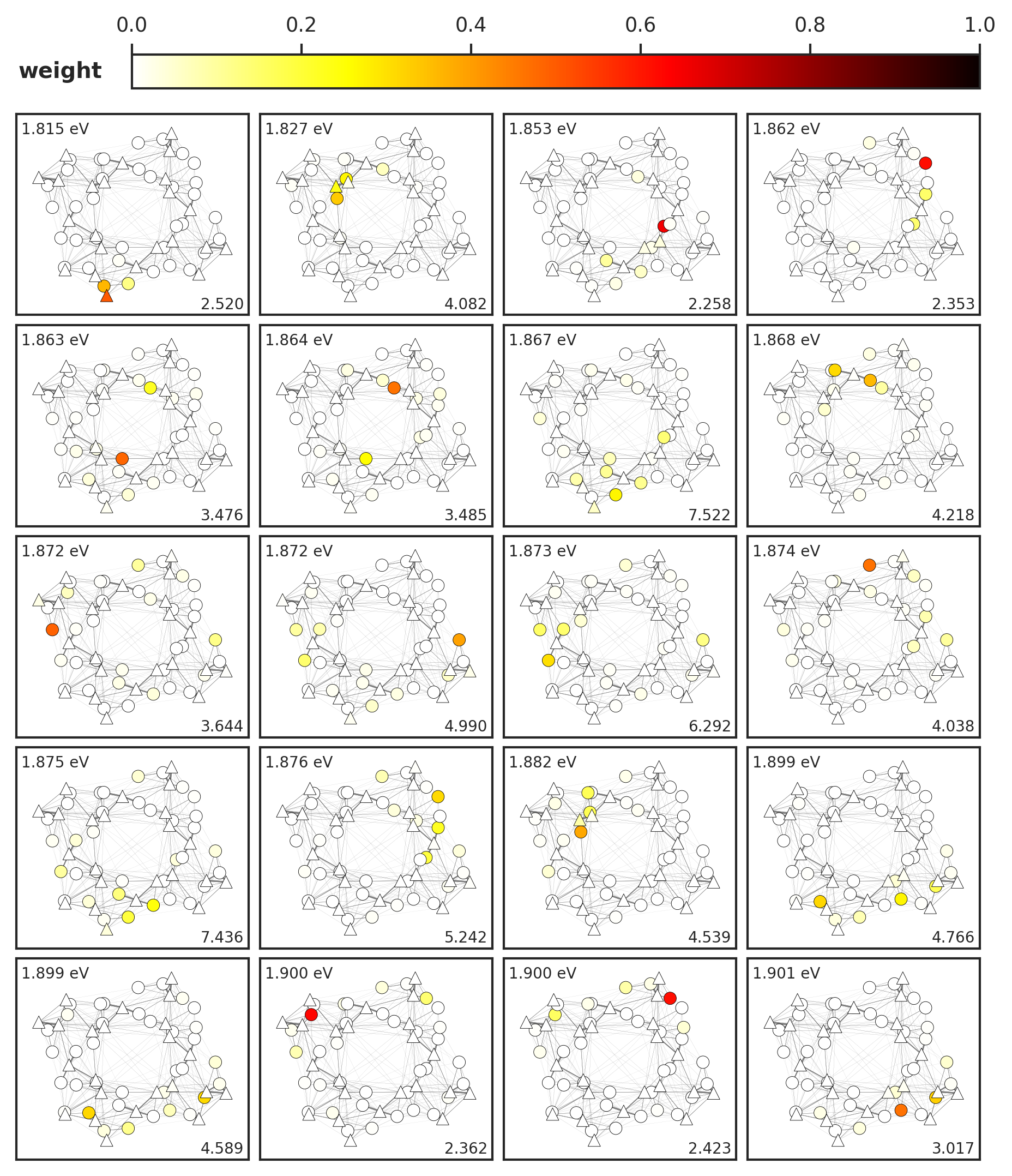

Supplement: Supplementary file 2 [file jp5c02465_si_002.zip › Fig6Analogues/tFCP/tFCP_ChlbreplacedbyChla_Q_part2.png]

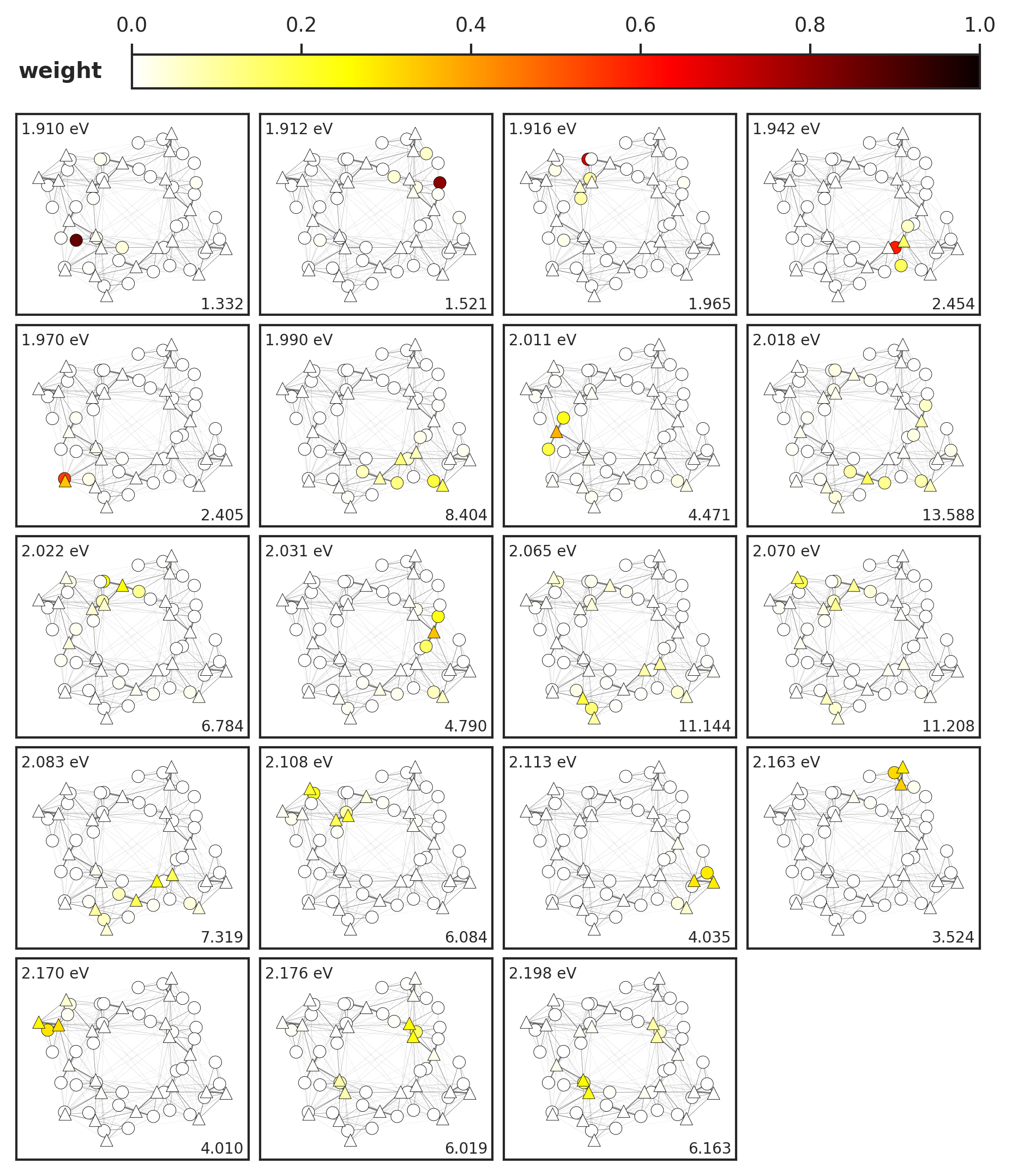

Supplement: Supplementary file 2 [file jp5c02465_si_002.zip › Fig6Analogues/tFCP/tFCP_ChlbreplacedbyChla_Q_part3.png]

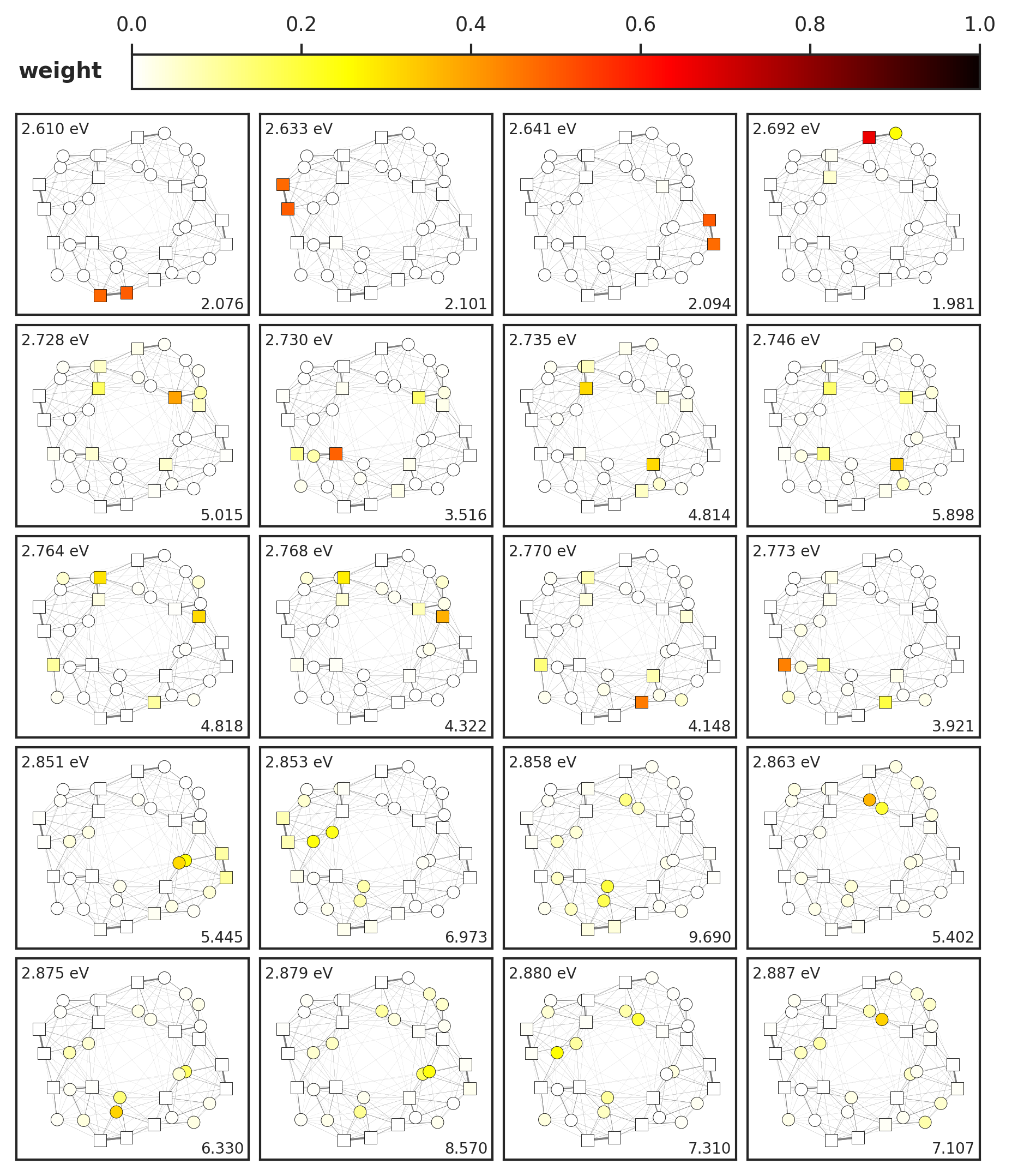

Supplement: Supplementary file 2 [file jp5c02465_si_002.zip › Fig6Analogues/tFCP/tFCP_noCrts_B_part1.png]

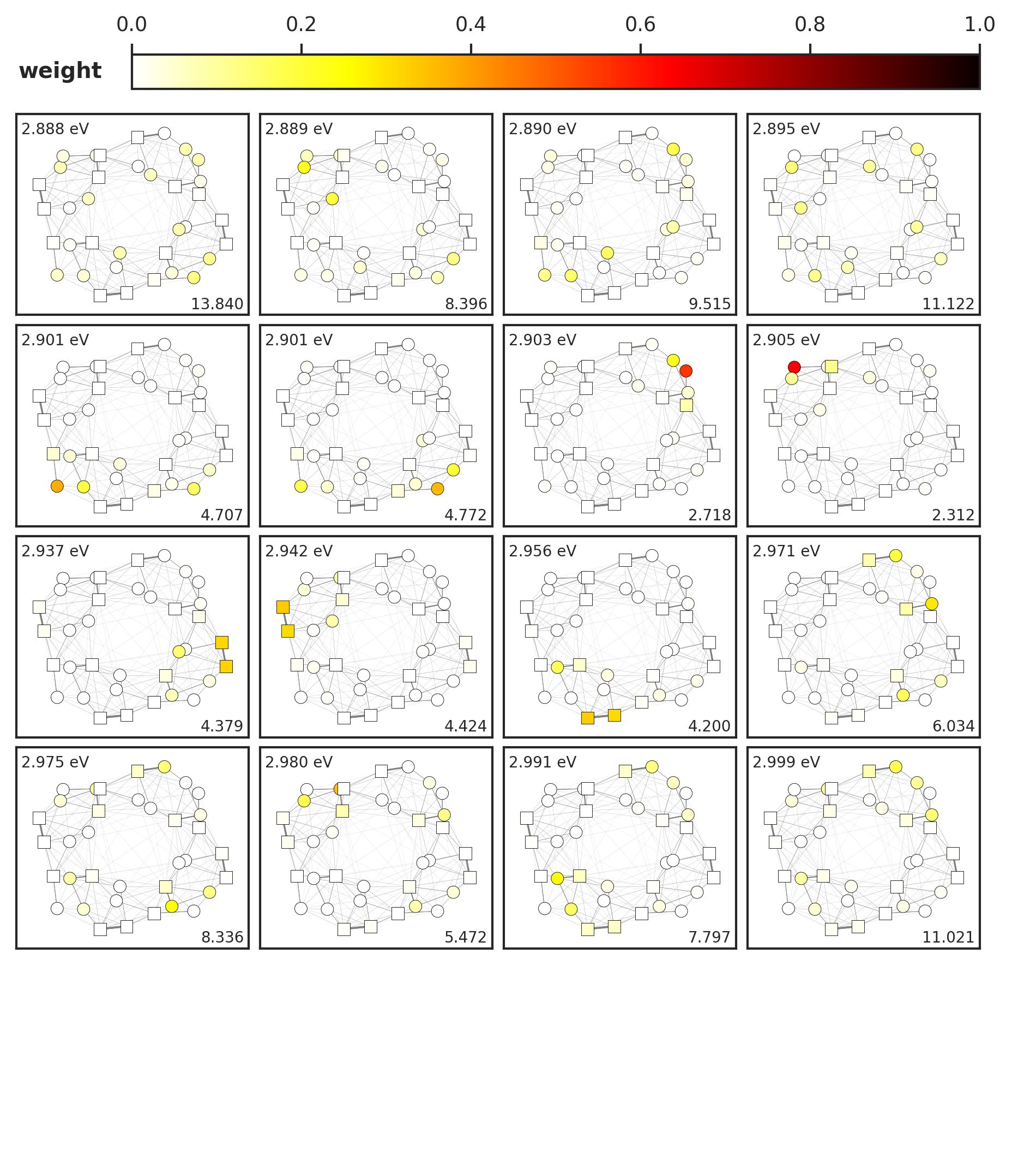

Supplement: Supplementary file 2 [file jp5c02465_si_002.zip › Fig6Analogues/tFCP/tFCP_noCrts_B_part2.png]

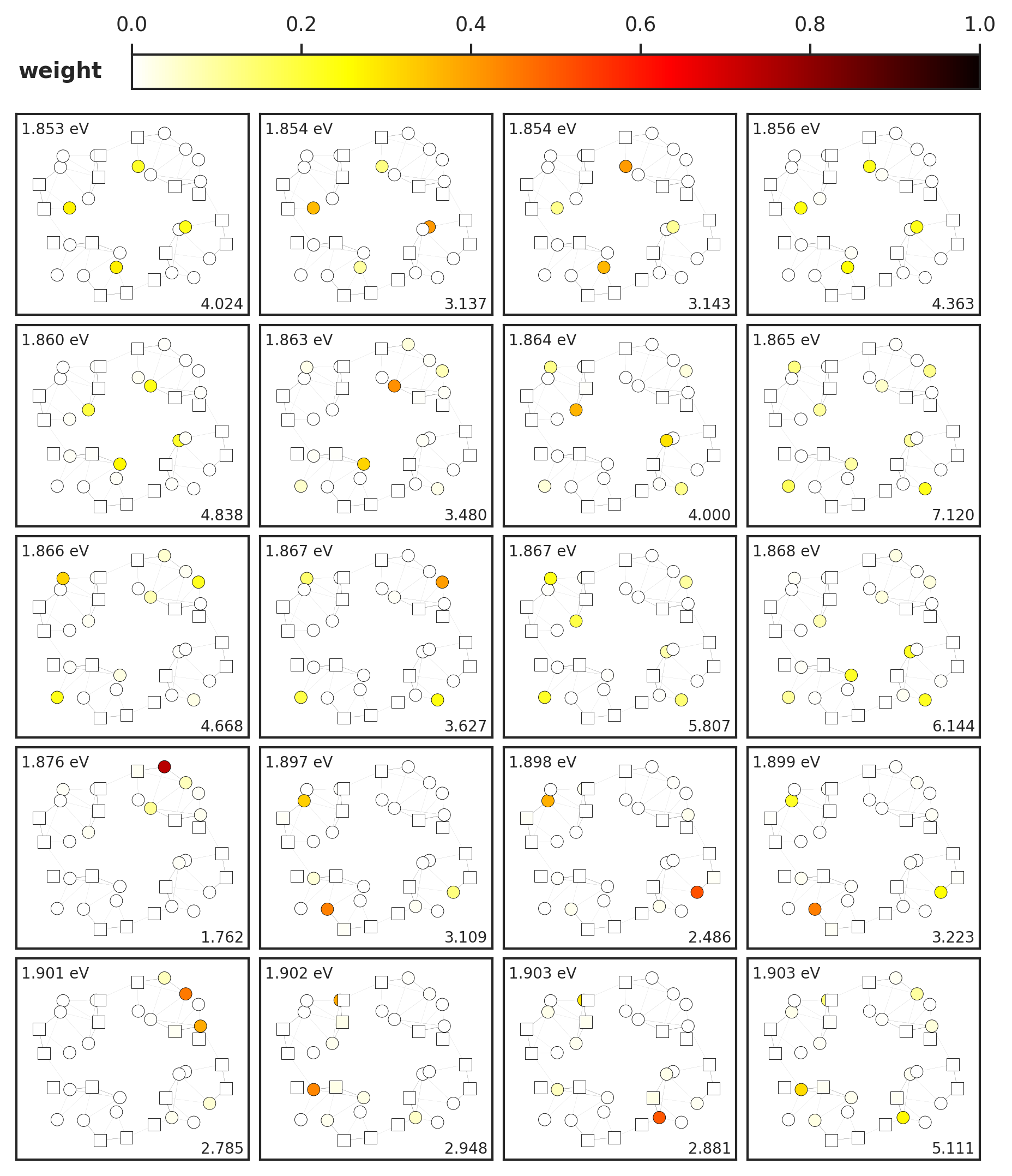

Supplement: Supplementary file 2 [file jp5c02465_si_002.zip › Fig6Analogues/tFCP/tFCP_noCrts_Q_part1.png]

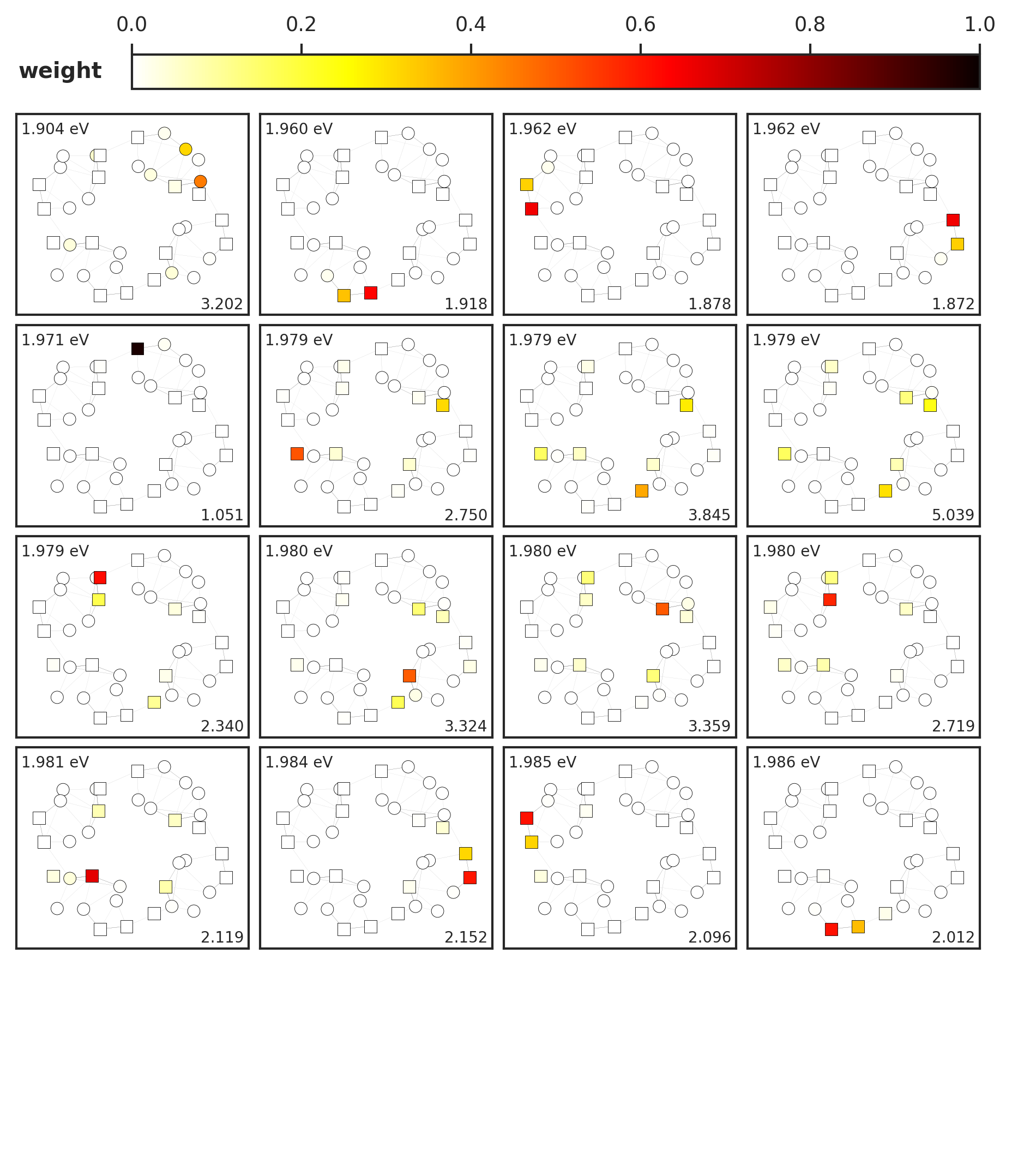

Supplement: Supplementary file 2 [file jp5c02465_si_002.zip › Fig6Analogues/tFCP/tFCP_noCrts_Q_part2.png]

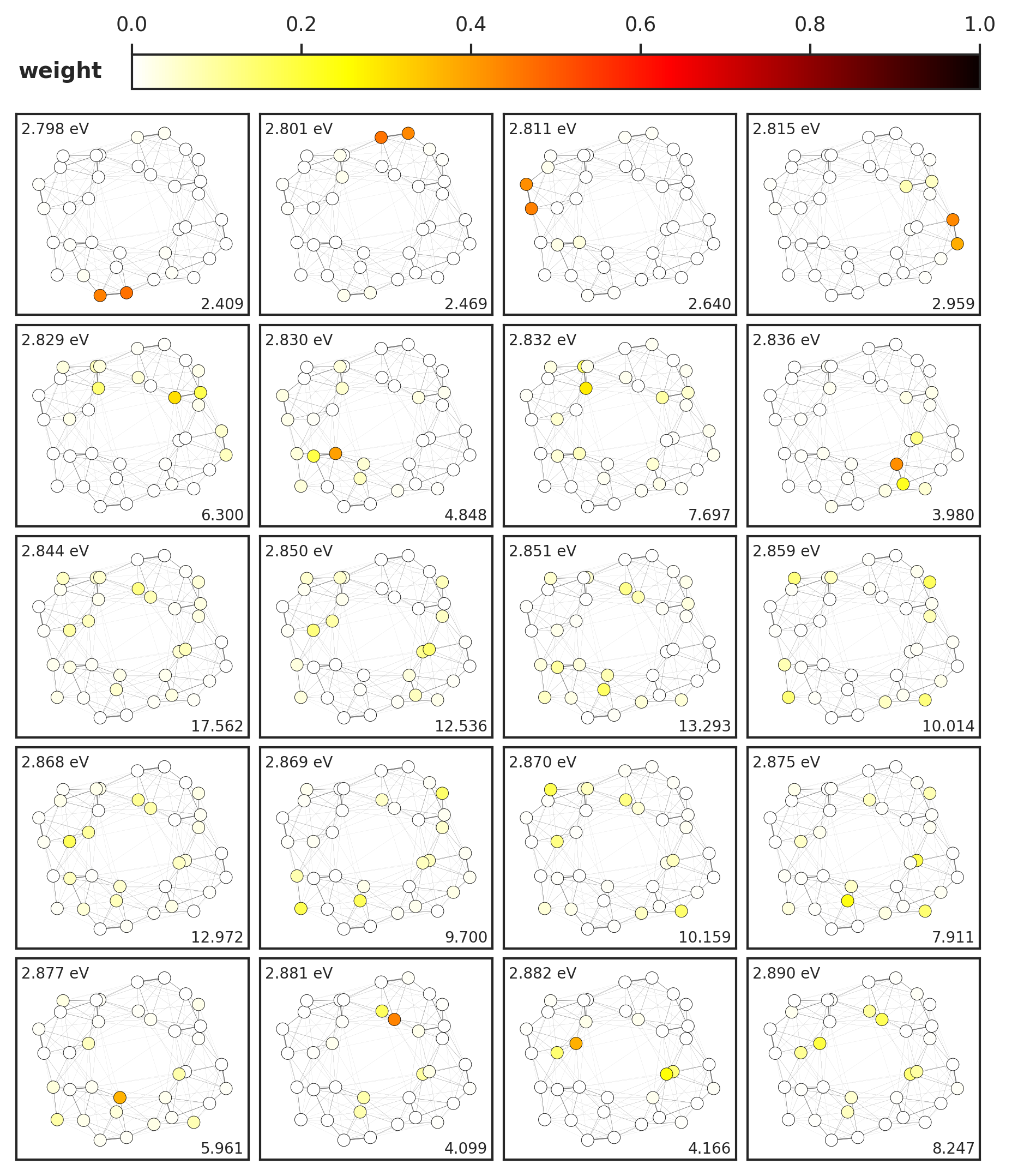

Supplement: Supplementary file 2 [file jp5c02465_si_002.zip › Fig6Analogues/tFCP/tFCP_onlyChla_B_comp_part1.png]

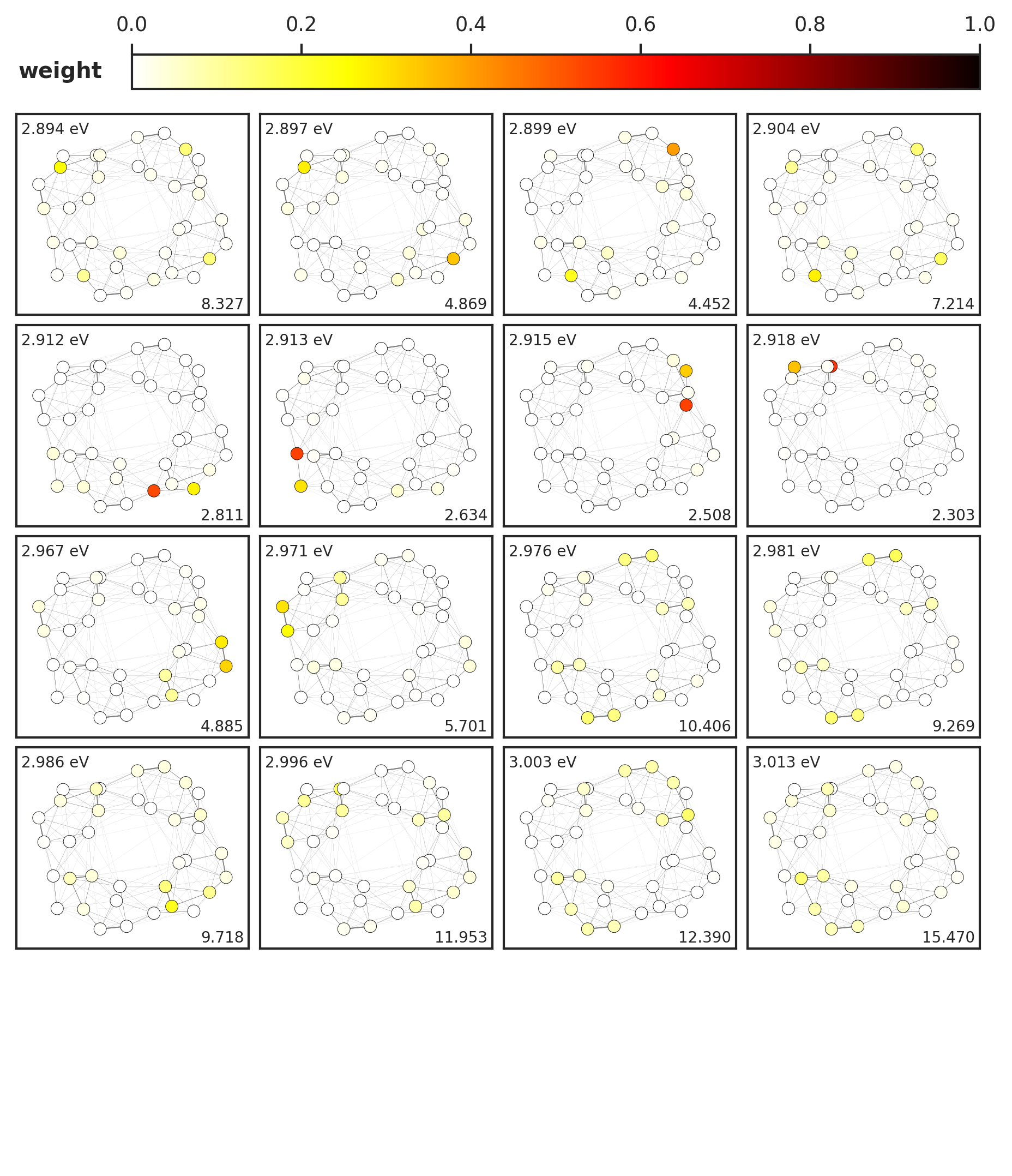

Supplement: Supplementary file 2 [file jp5c02465_si_002.zip › Fig6Analogues/tFCP/tFCP_onlyChla_B_comp_part2.png]

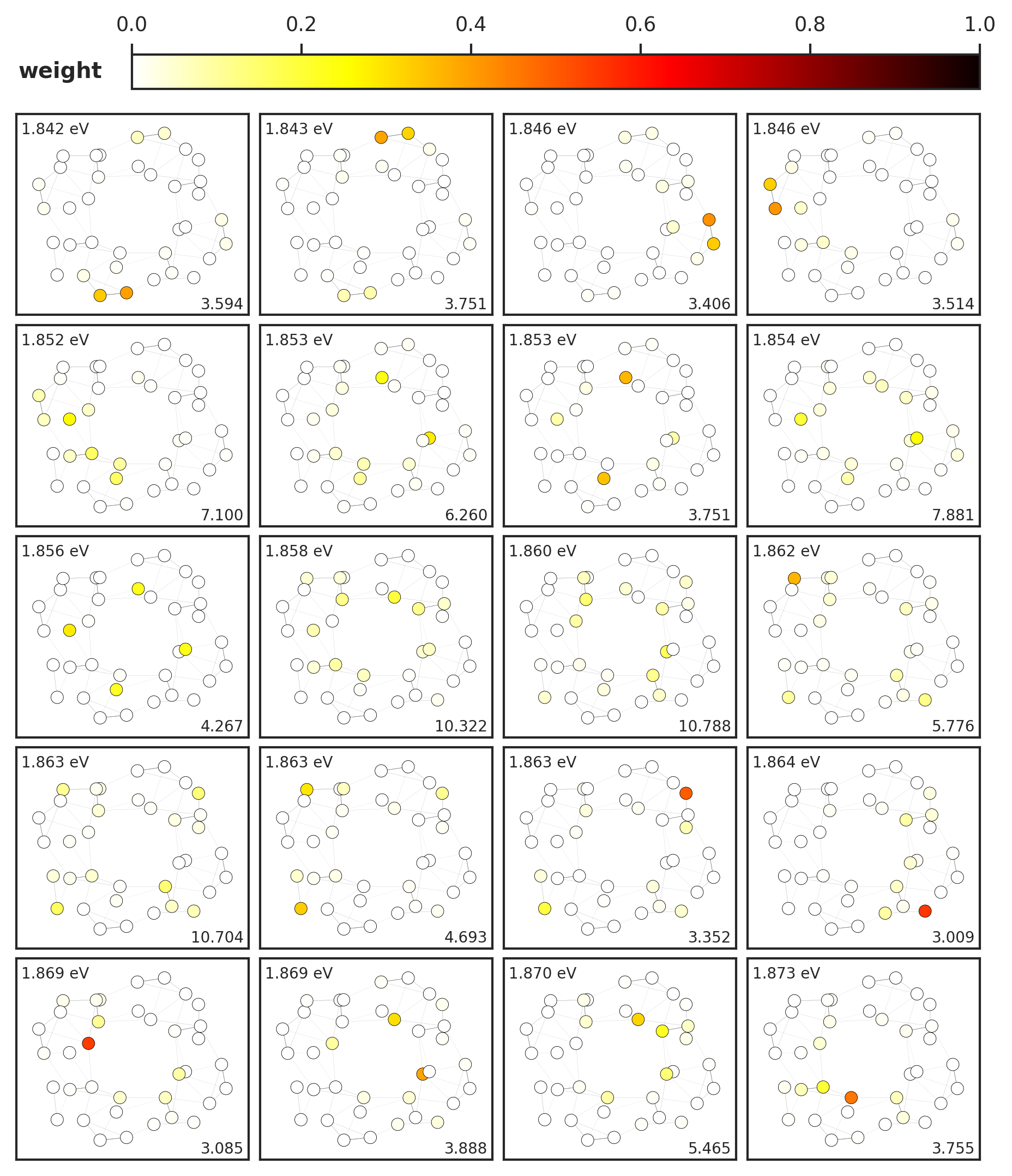

Supplement: Supplementary file 2 [file jp5c02465_si_002.zip › Fig6Analogues/tFCP/tFCP_onlyChla_Q_comp_part1.png]

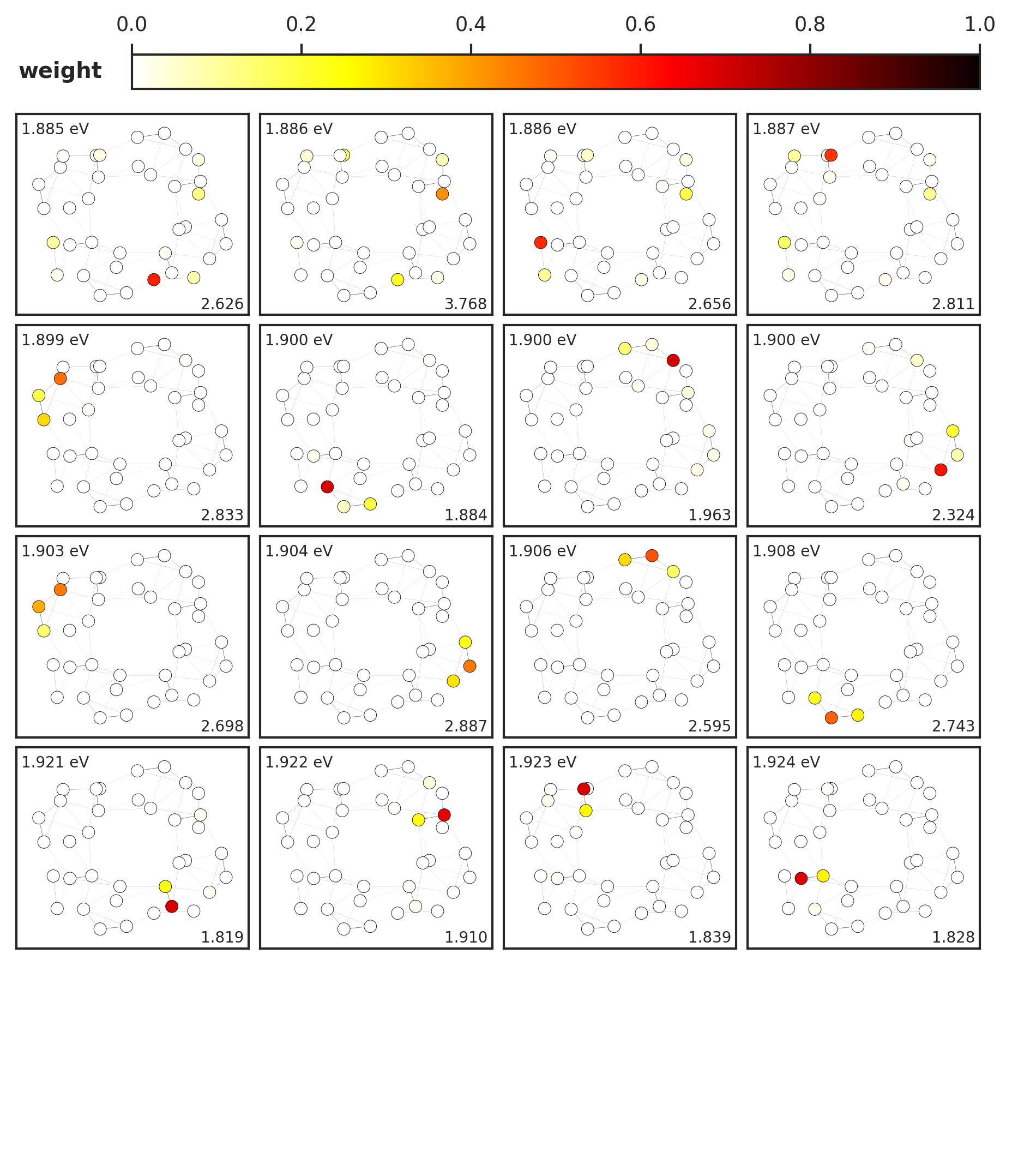

Supplement: Supplementary file 2 [file jp5c02465_si_002.zip › Fig6Analogues/tFCP/tFCP_onlyChla_Q_comp_part2.png]

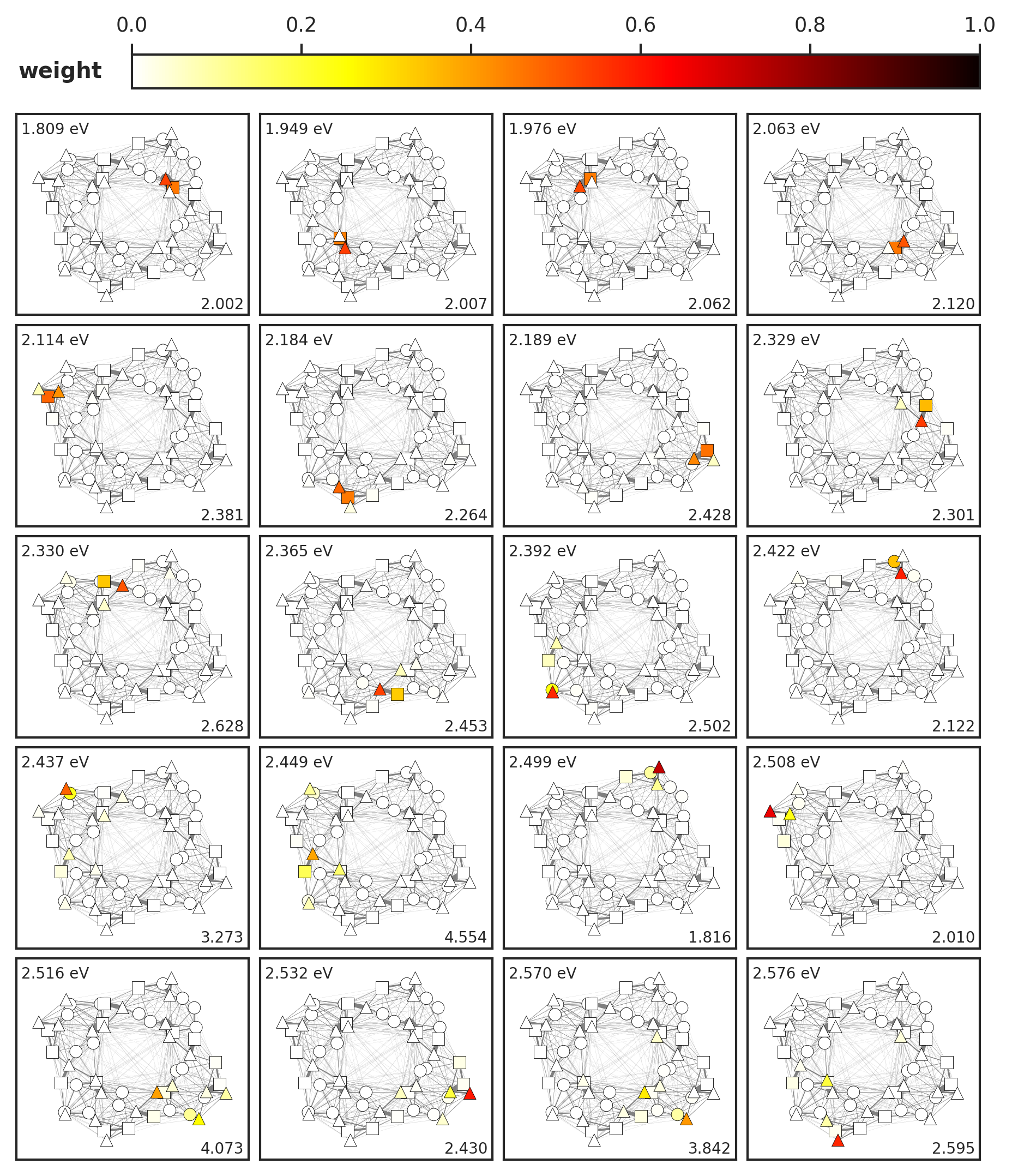

Supplement: Supplementary file 2 [file jp5c02465_si_002.zip › Fig6Analogues/tFCP/tFCP_WT_B_part1.png]

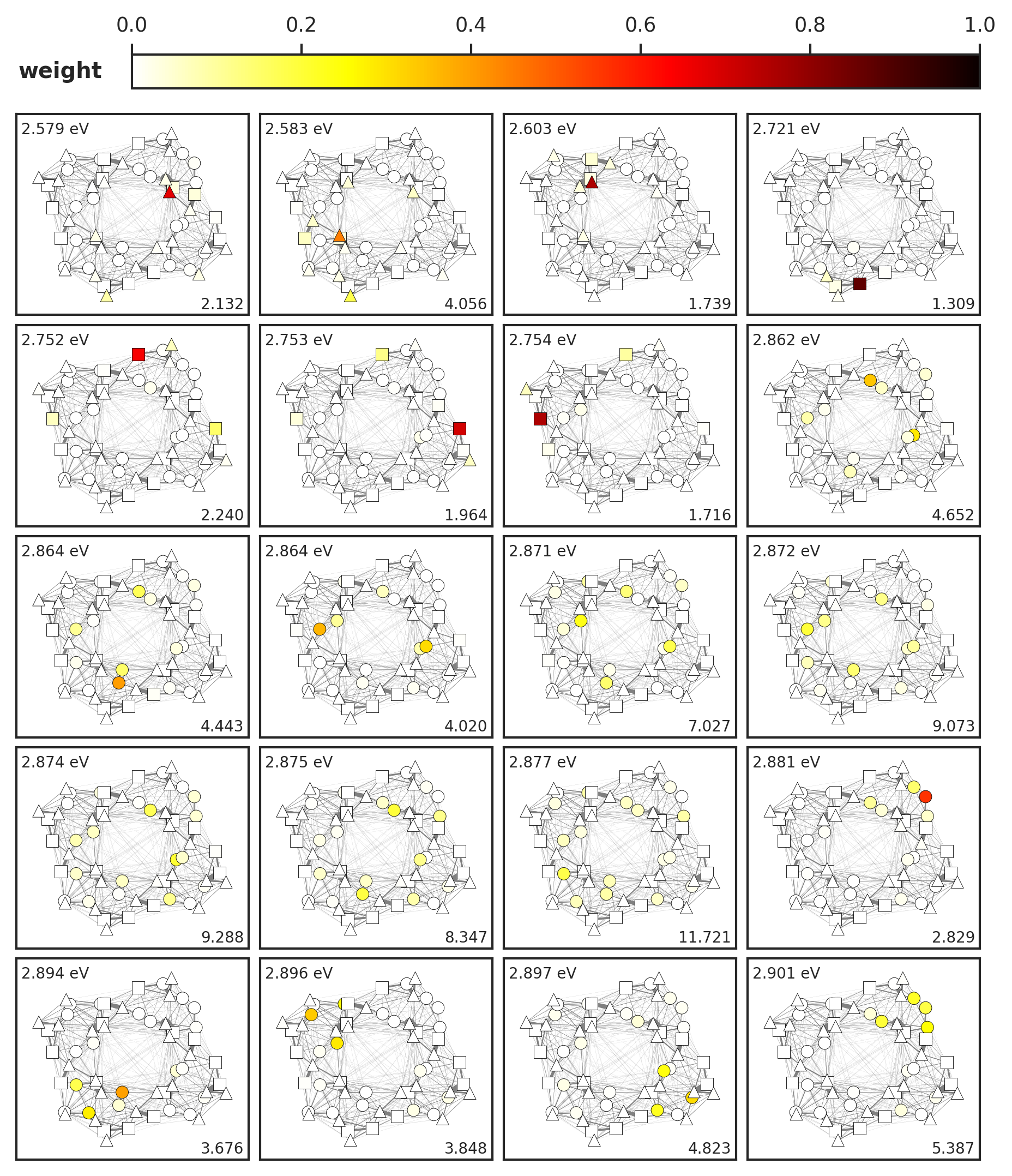

Supplement: Supplementary file 2 [file jp5c02465_si_002.zip › Fig6Analogues/tFCP/tFCP_WT_B_part2.png]

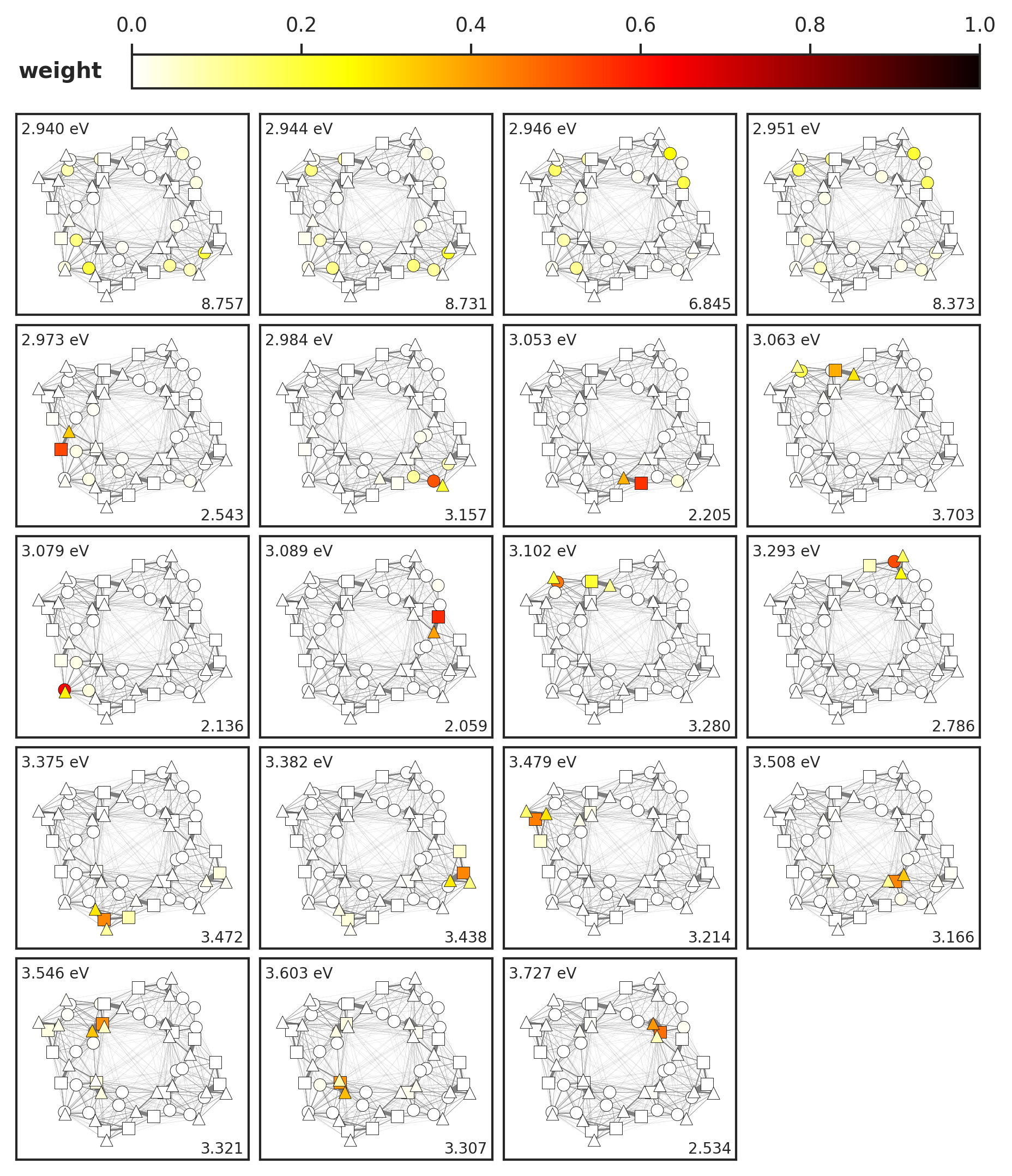

Supplement: Supplementary file 2 [file jp5c02465_si_002.zip › Fig6Analogues/tFCP/tFCP_WT_B_part3.png]

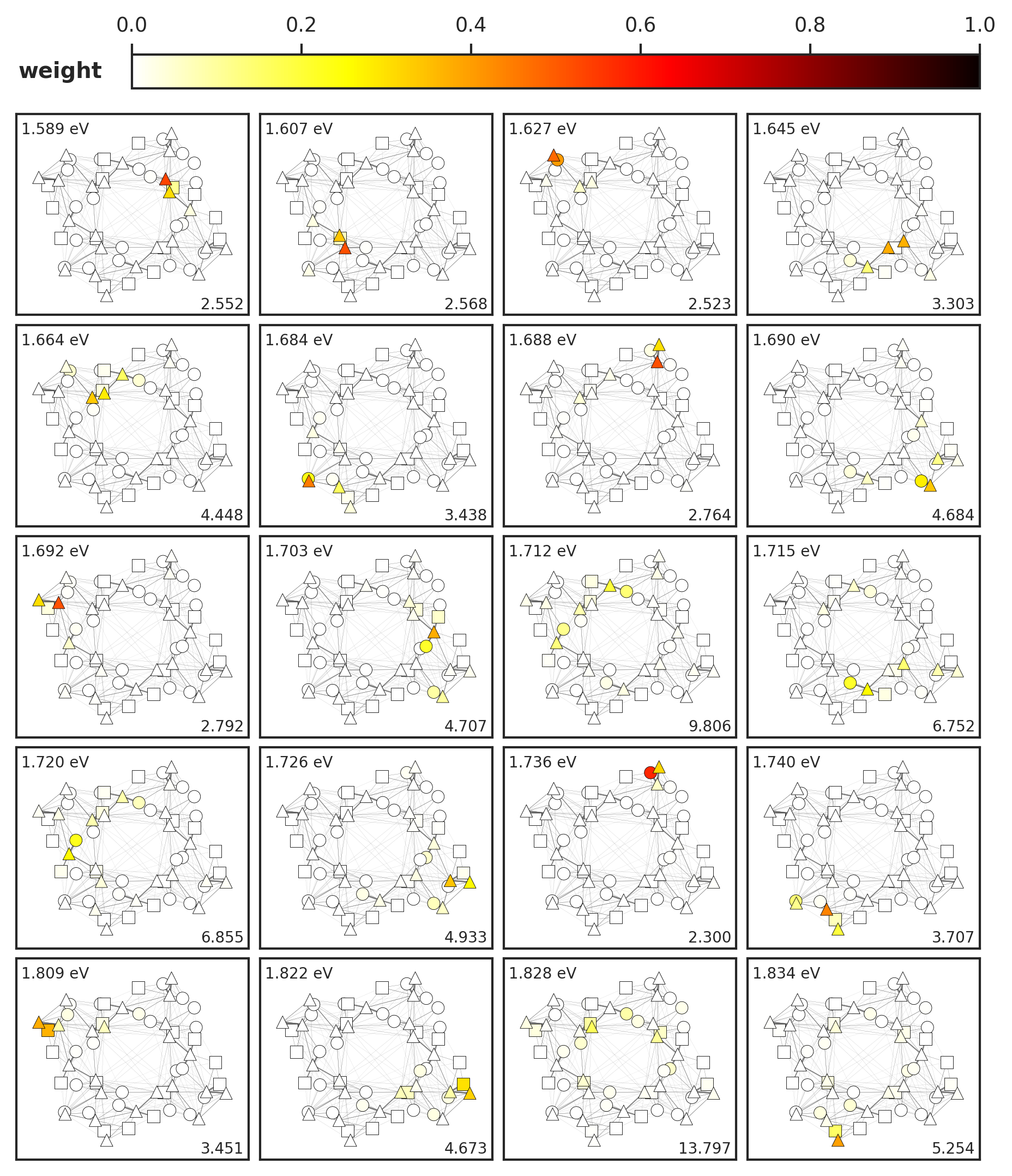

Supplement: Supplementary file 2 [file jp5c02465_si_002.zip › Fig6Analogues/tFCP/tFCP_WT_Q_part1.png]

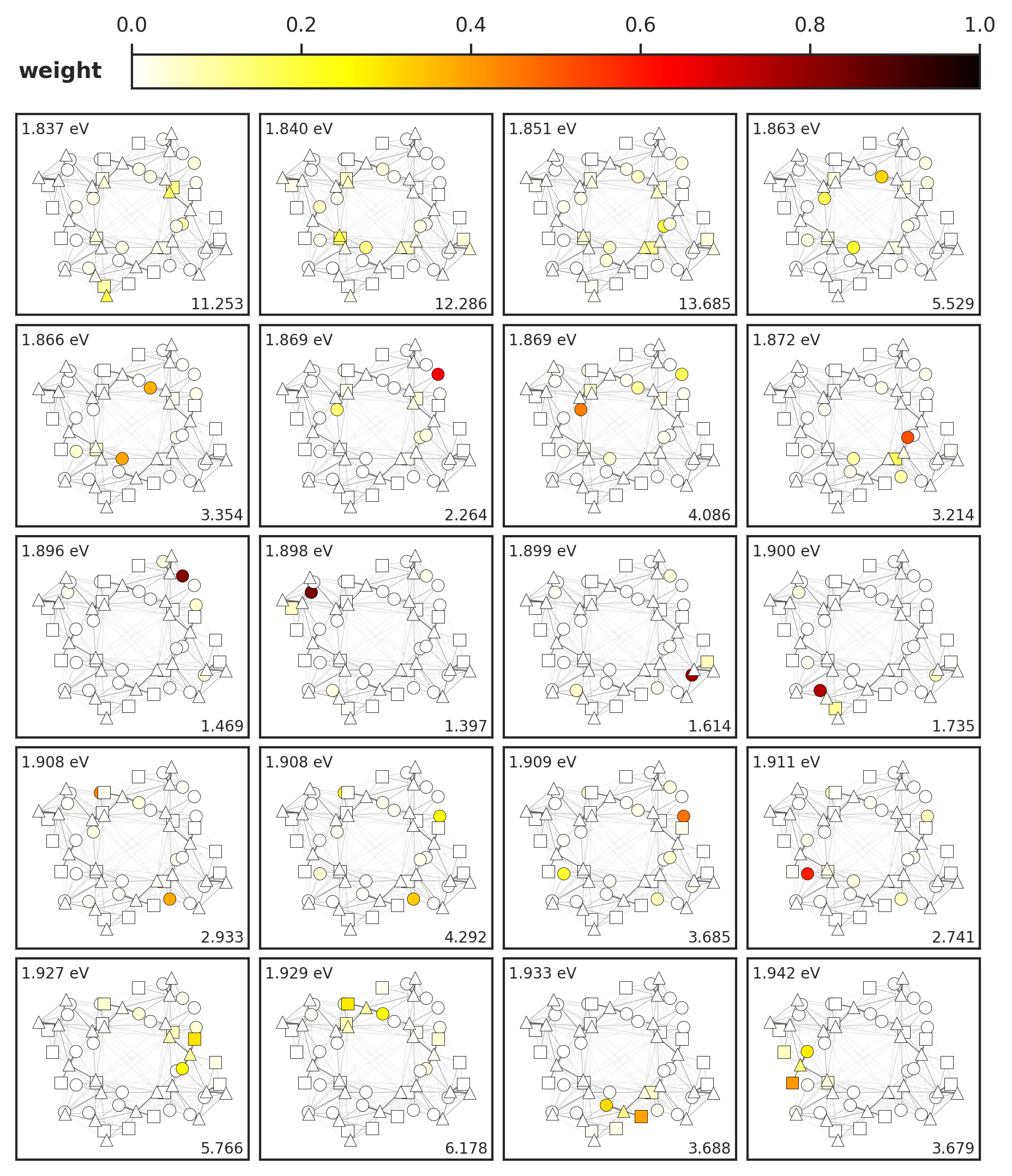

Supplement: Supplementary file 2 [file jp5c02465_si_002.zip › Fig6Analogues/tFCP/tFCP_WT_Q_part2.png]

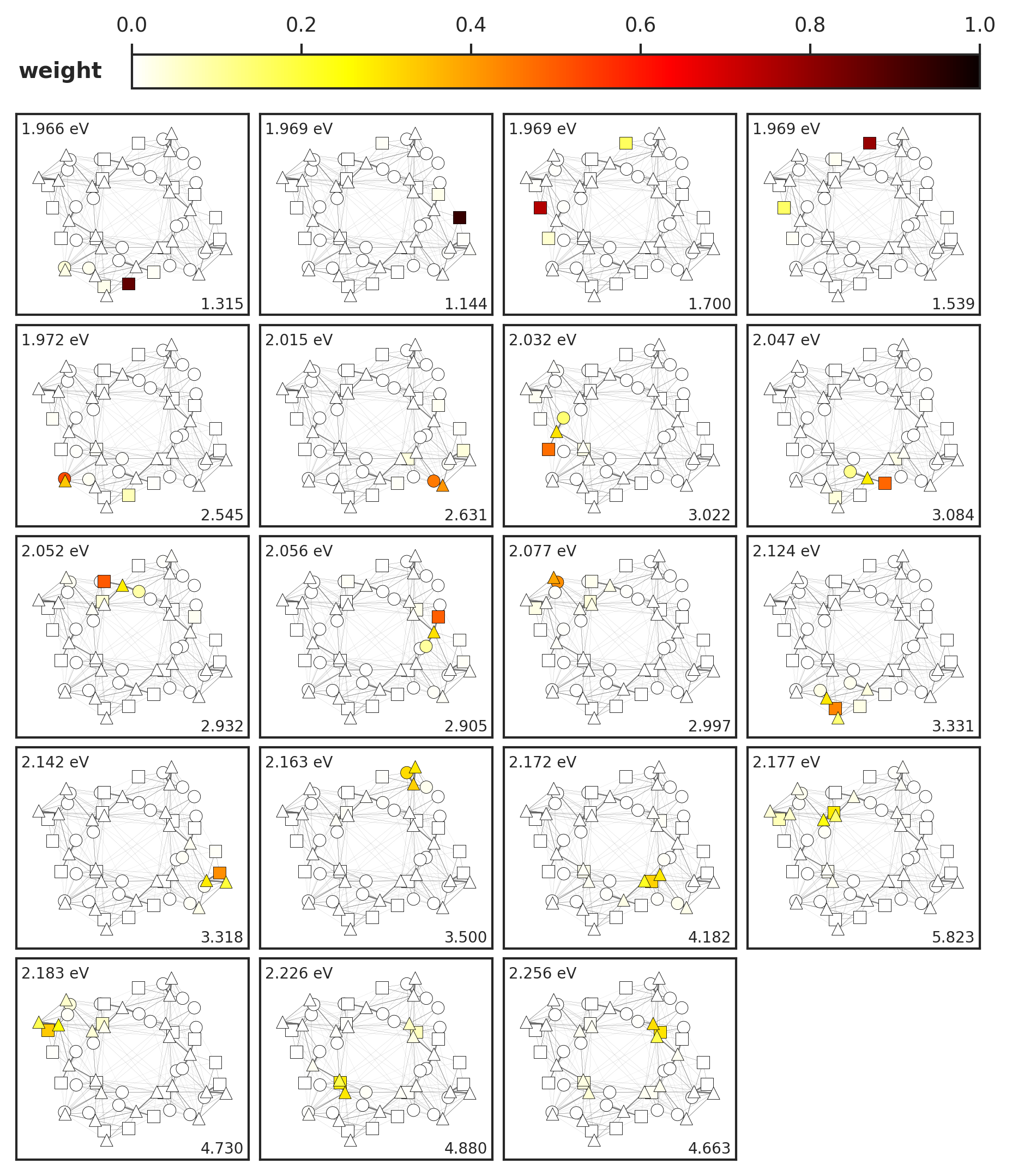

Supplement: Supplementary file 2 [file jp5c02465_si_002.zip › Fig6Analogues/tFCP/tFCP_WT_Q_part3.png]

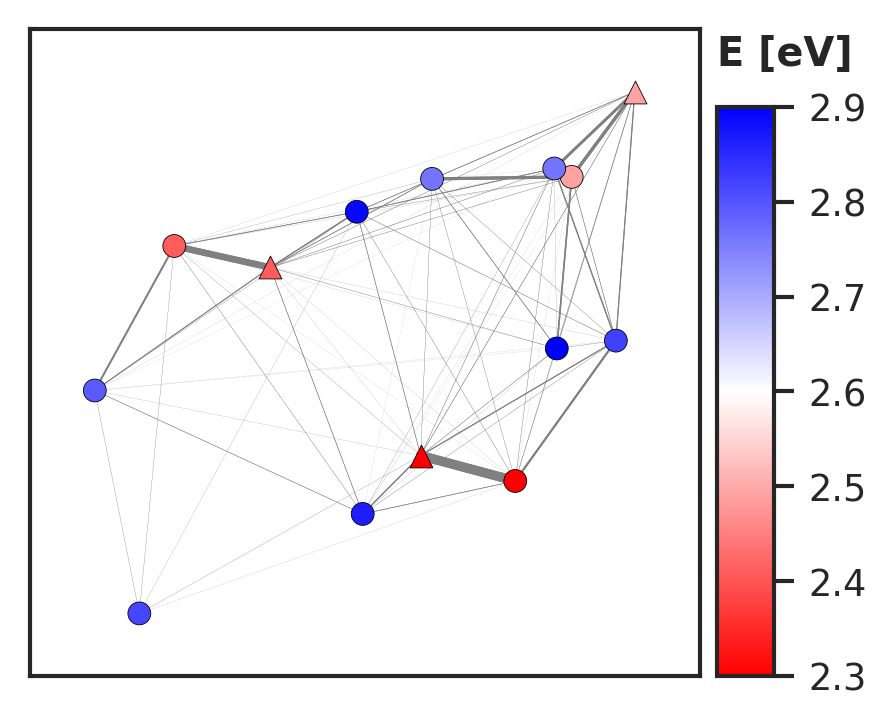

Supplement: Supplementary file 2 [file jp5c02465_si_002.zip › Fig4Analogues/CP24/CP24_ChlbreplacedbyChla_B.png]

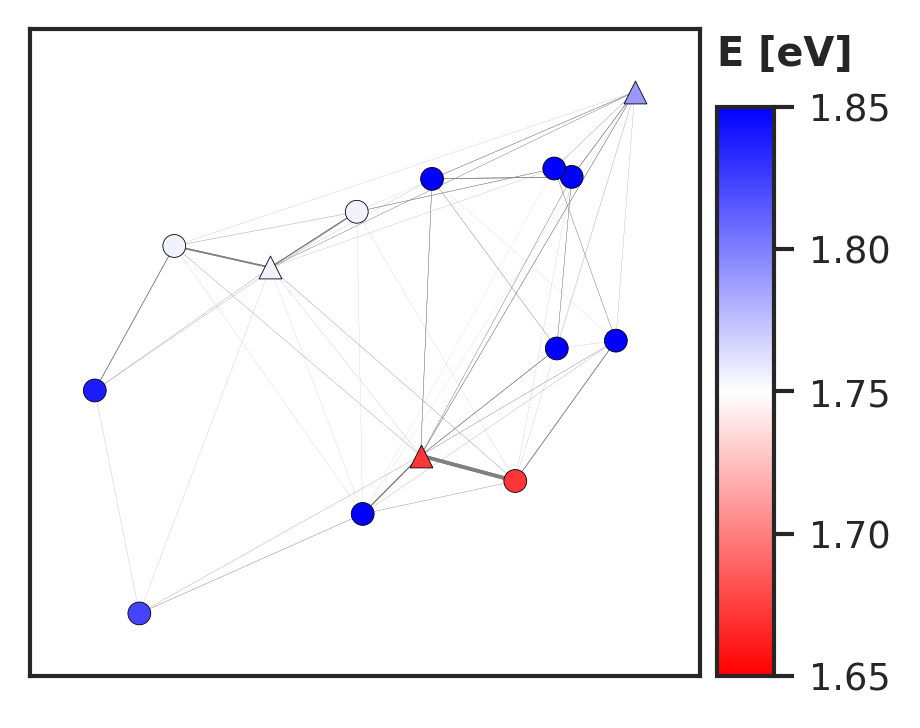

Supplement: Supplementary file 2 [file jp5c02465_si_002.zip › Fig4Analogues/CP24/CP24_ChlbreplacedbyChla_Q.png]

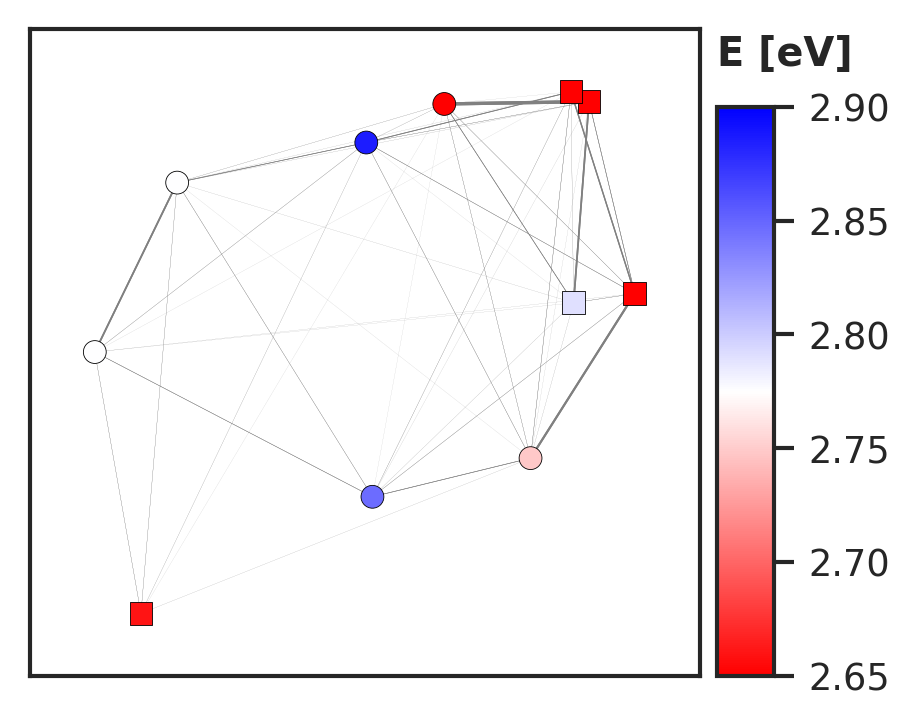

Supplement: Supplementary file 2 [file jp5c02465_si_002.zip › Fig4Analogues/CP24/CP24_noCrts_B.png]

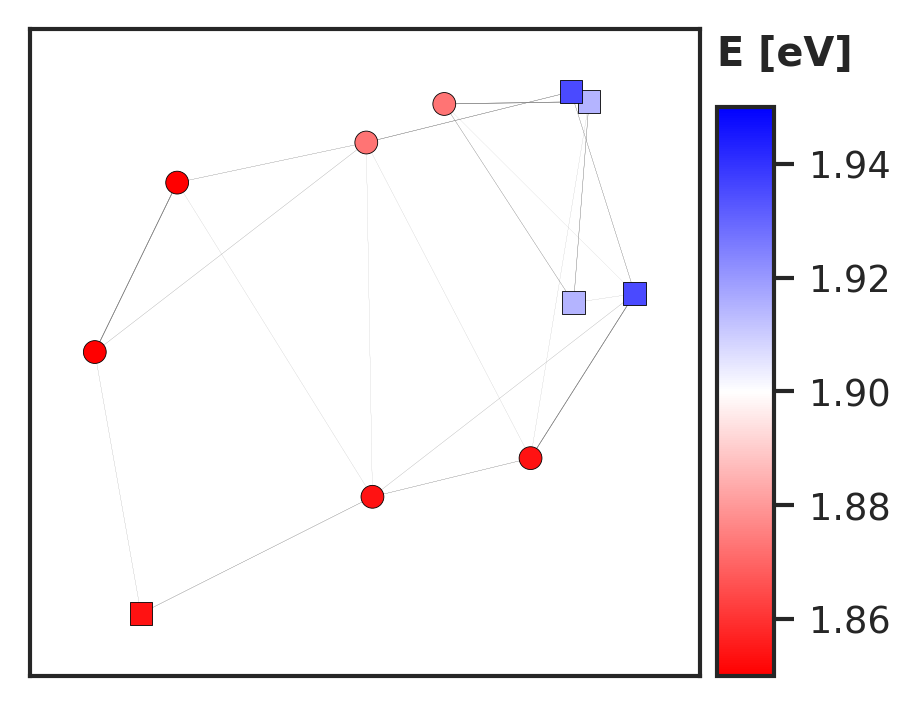

Supplement: Supplementary file 2 [file jp5c02465_si_002.zip › Fig4Analogues/CP24/CP24_noCrts_Q.png]

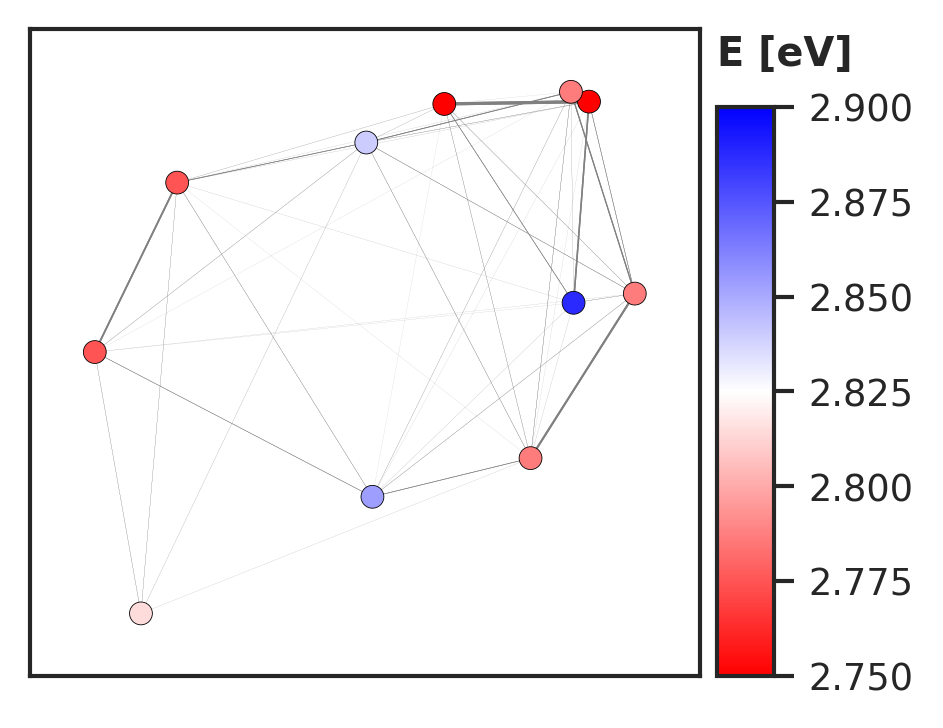

Supplement: Supplementary file 2 [file jp5c02465_si_002.zip › Fig4Analogues/CP24/CP24_onlyChla_B.png]

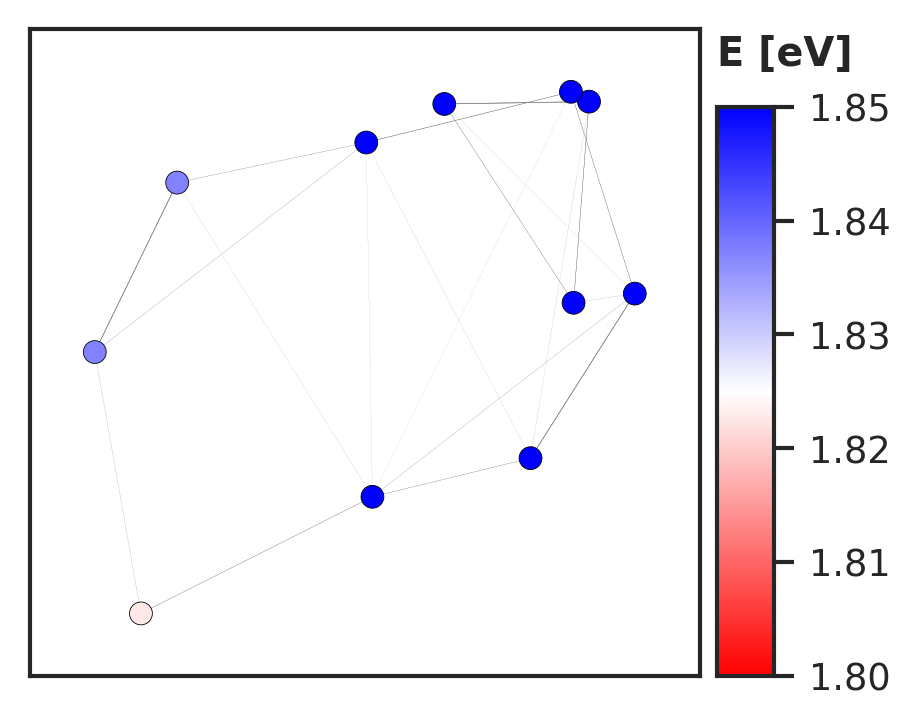

Supplement: Supplementary file 2 [file jp5c02465_si_002.zip › Fig4Analogues/CP24/CP24_onlyChla_Q.png]

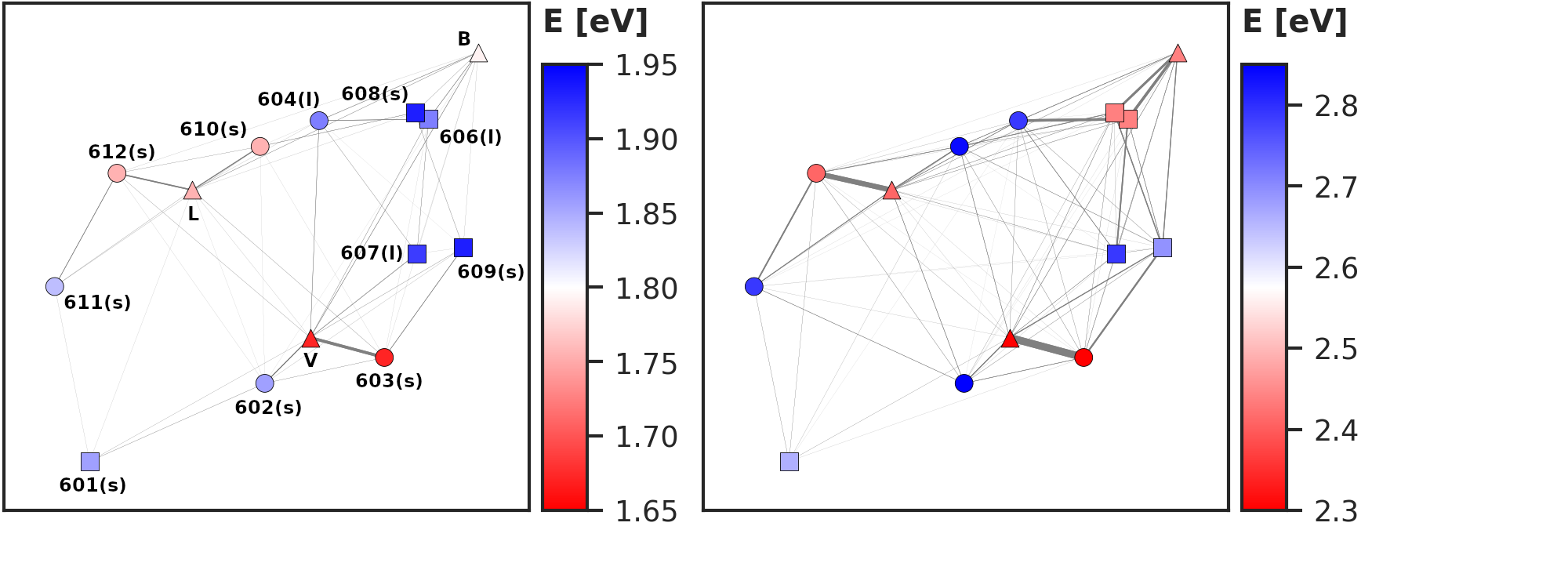

Supplement: Supplementary file 2 [file jp5c02465_si_002.zip › Fig4Analogues/CP24/CP24_WT_QandB.png]

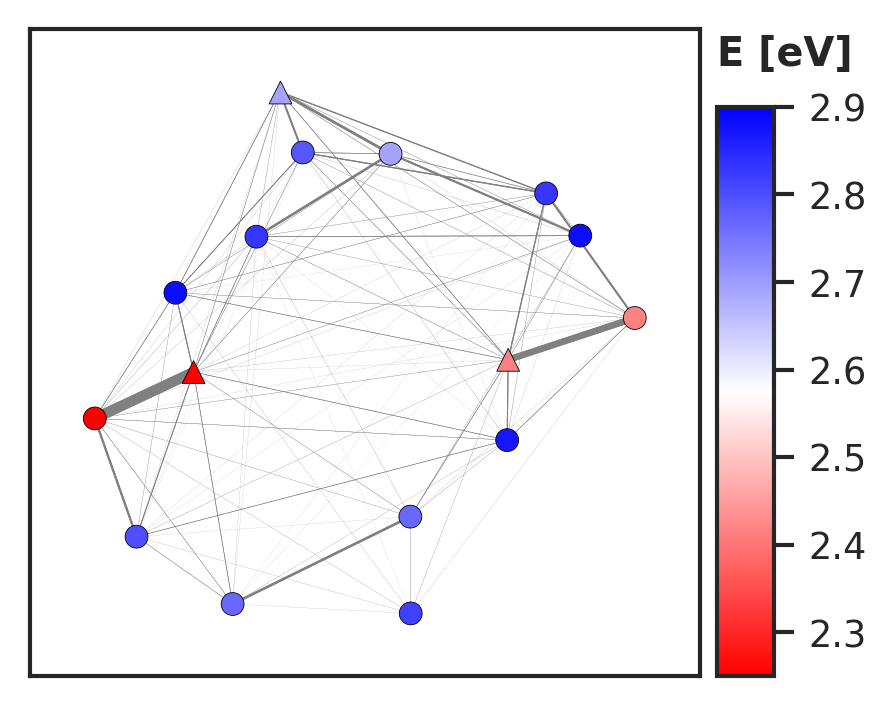

Supplement: Supplementary file 2 [file jp5c02465_si_002.zip › Fig4Analogues/CP26/CP26_ChlbreplacedbyChla_B.png]

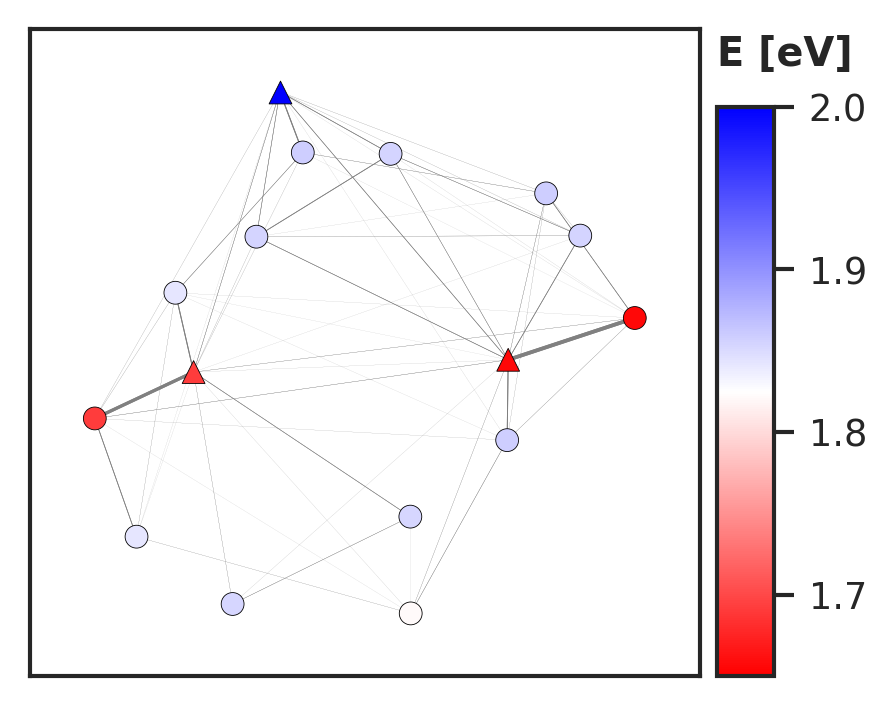

Supplement: Supplementary file 2 [file jp5c02465_si_002.zip › Fig4Analogues/CP26/CP26_ChlbreplacedbyChla_Q.png]

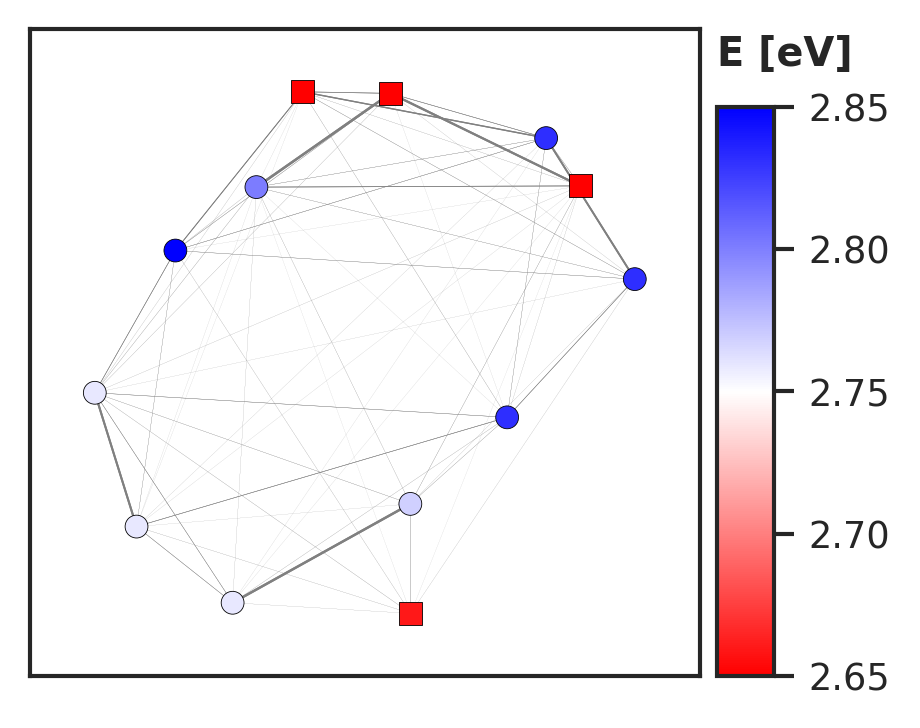

Supplement: Supplementary file 2 [file jp5c02465_si_002.zip › Fig4Analogues/CP26/CP26_noCrts_B.png]

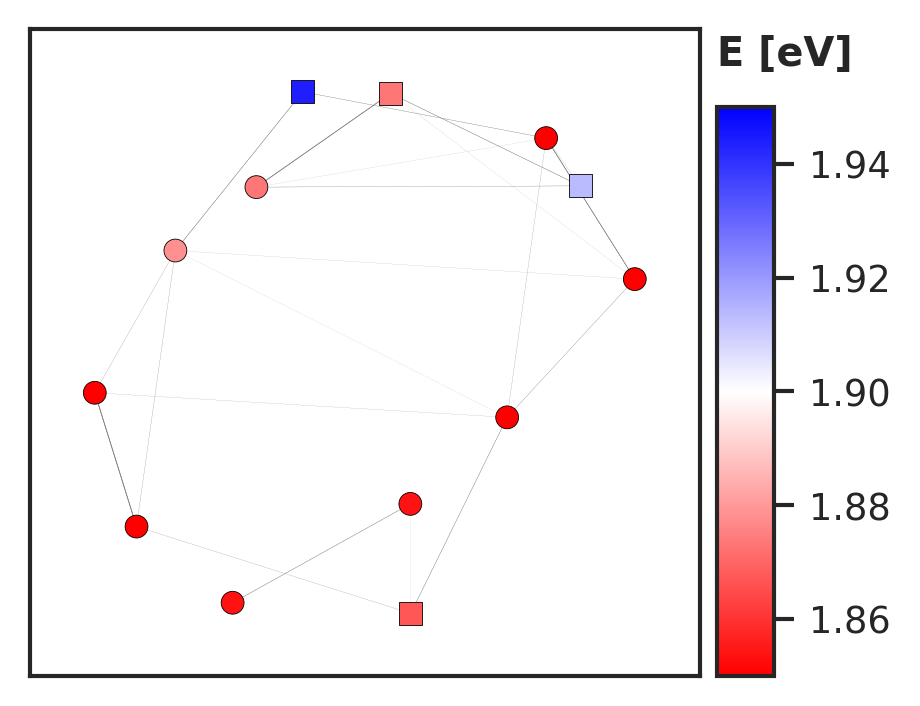

Supplement: Supplementary file 2 [file jp5c02465_si_002.zip › Fig4Analogues/CP26/CP26_noCrts_Q.png]

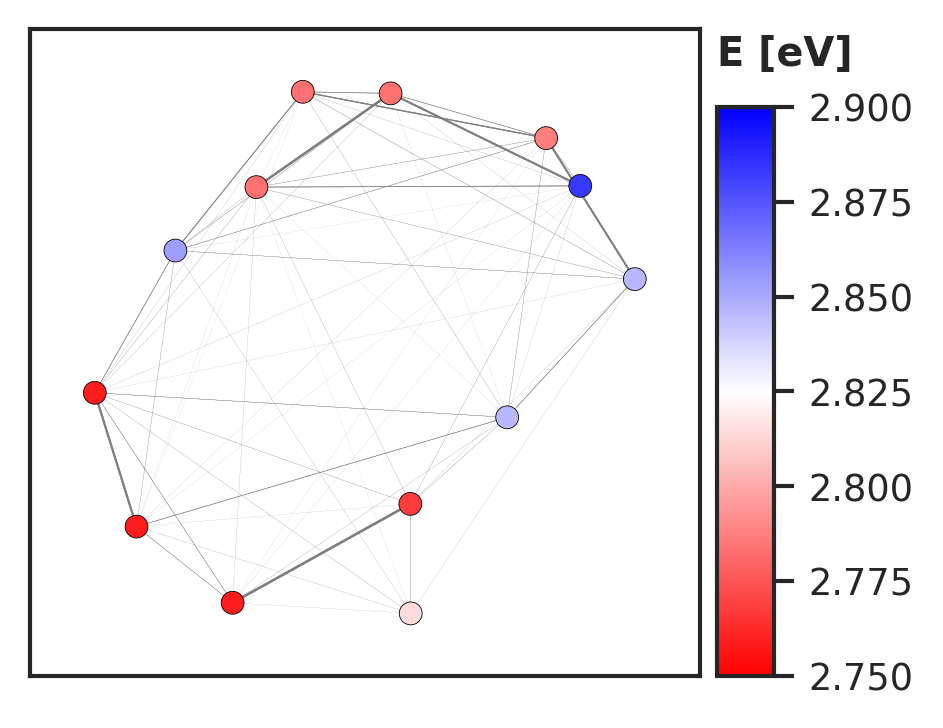

Supplement: Supplementary file 2 [file jp5c02465_si_002.zip › Fig4Analogues/CP26/CP26_onlyChla_B.png]

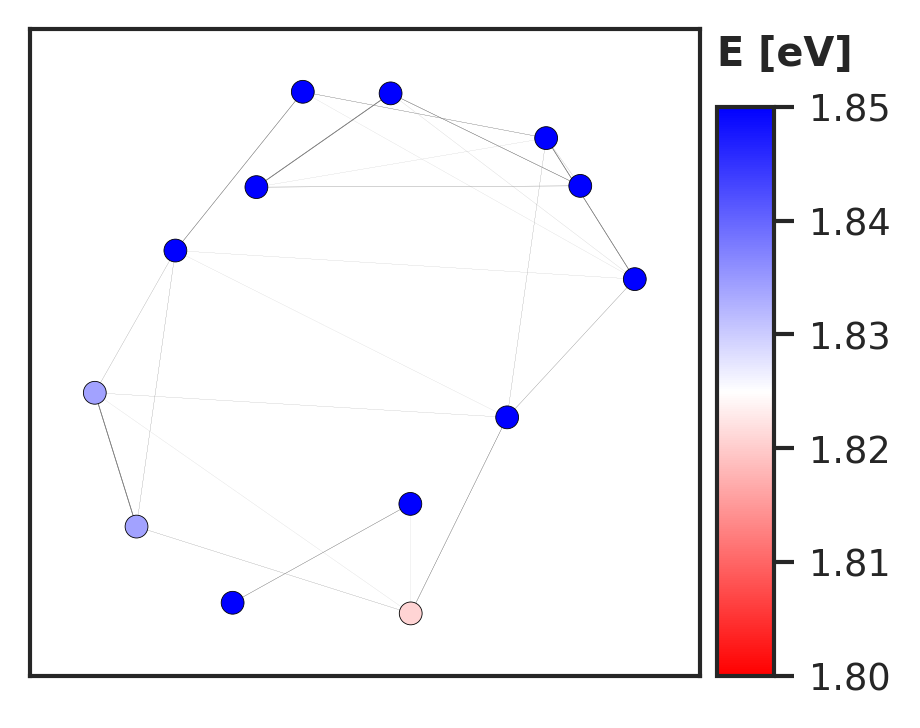

Supplement: Supplementary file 2 [file jp5c02465_si_002.zip › Fig4Analogues/CP26/CP26_onlyChla_Q.png]

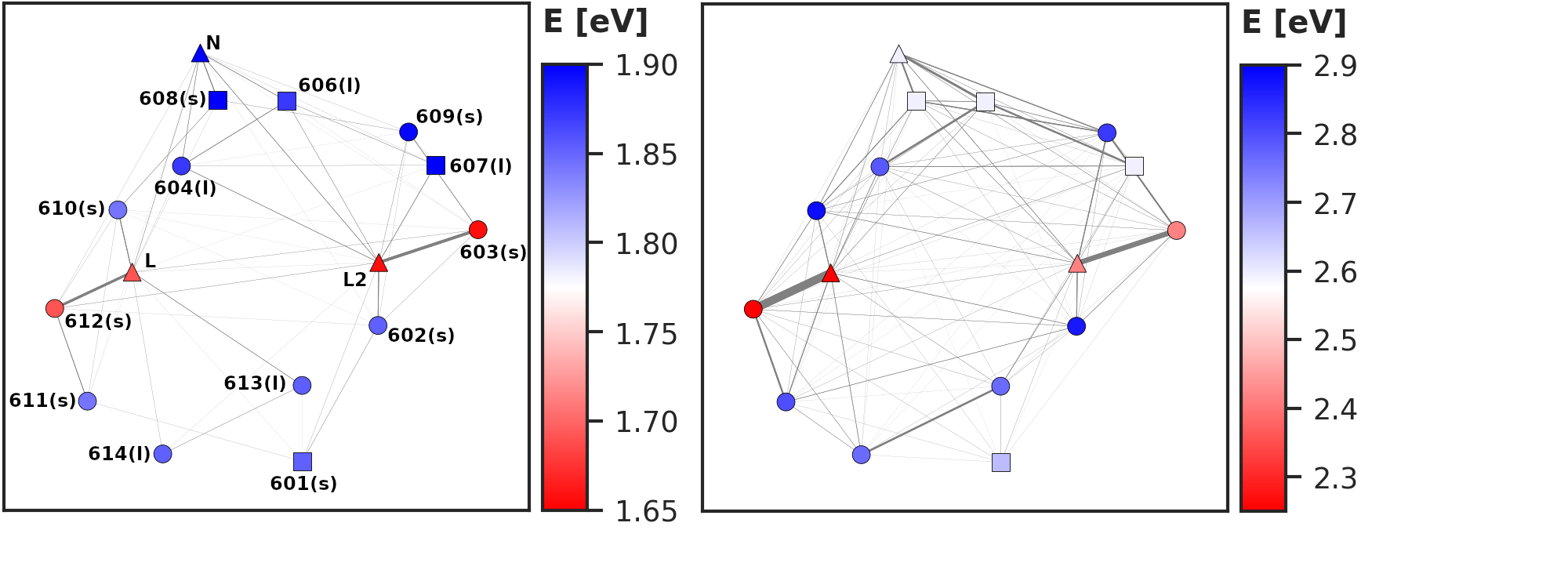

Supplement: Supplementary file 2 [file jp5c02465_si_002.zip › Fig4Analogues/CP26/CP26_WT_QandB.png]

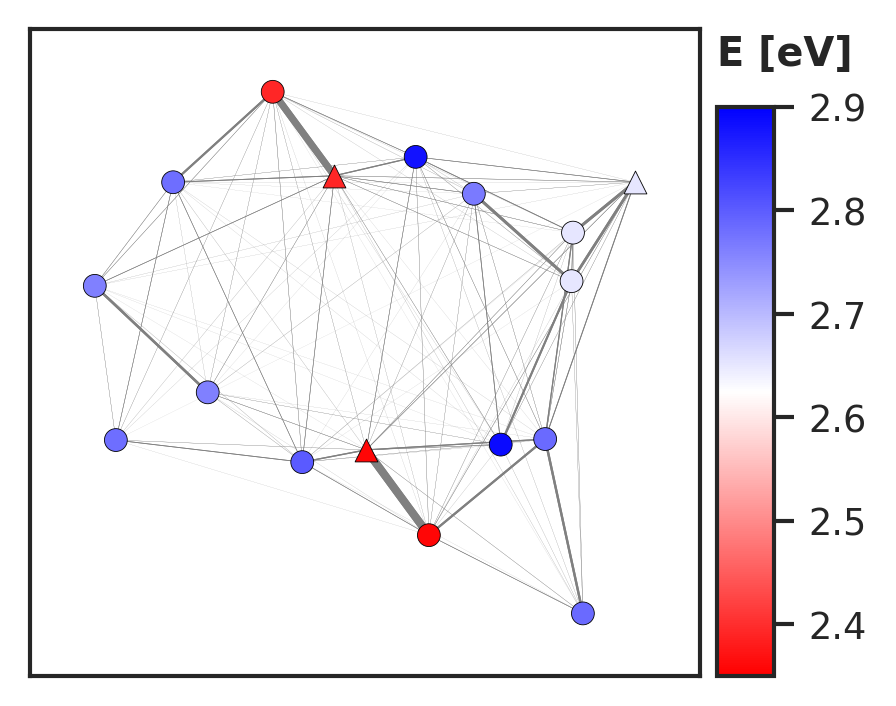

Supplement: Supplementary file 2 [file jp5c02465_si_002.zip › Fig4Analogues/CP29/CP29_ChlbreplacedbyChla_B.png]

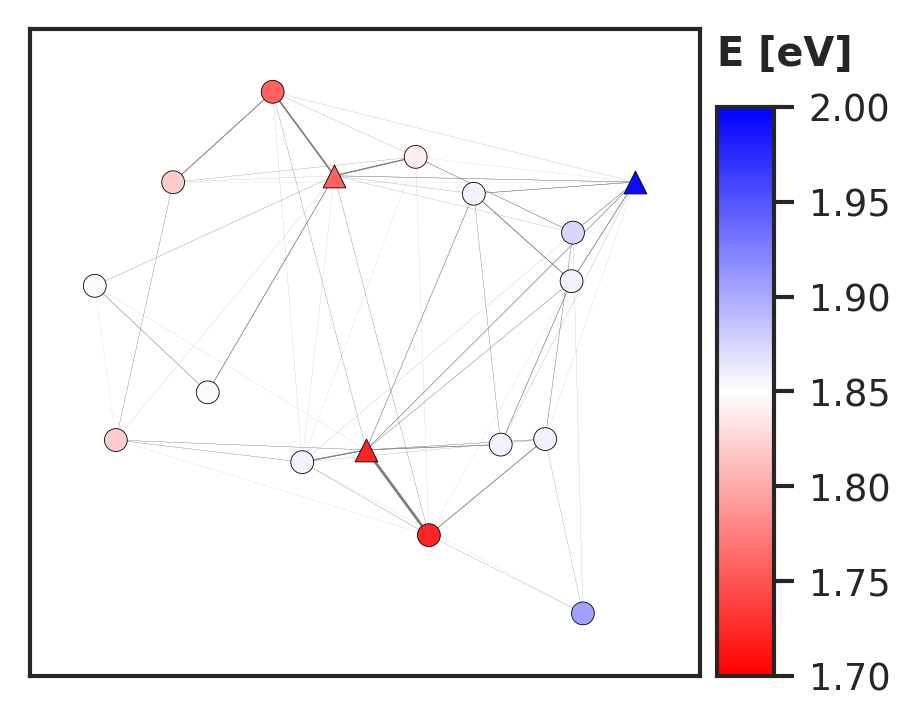

Supplement: Supplementary file 2 [file jp5c02465_si_002.zip › Fig4Analogues/CP29/CP29_ChlbreplacedbyChla_Q.png]

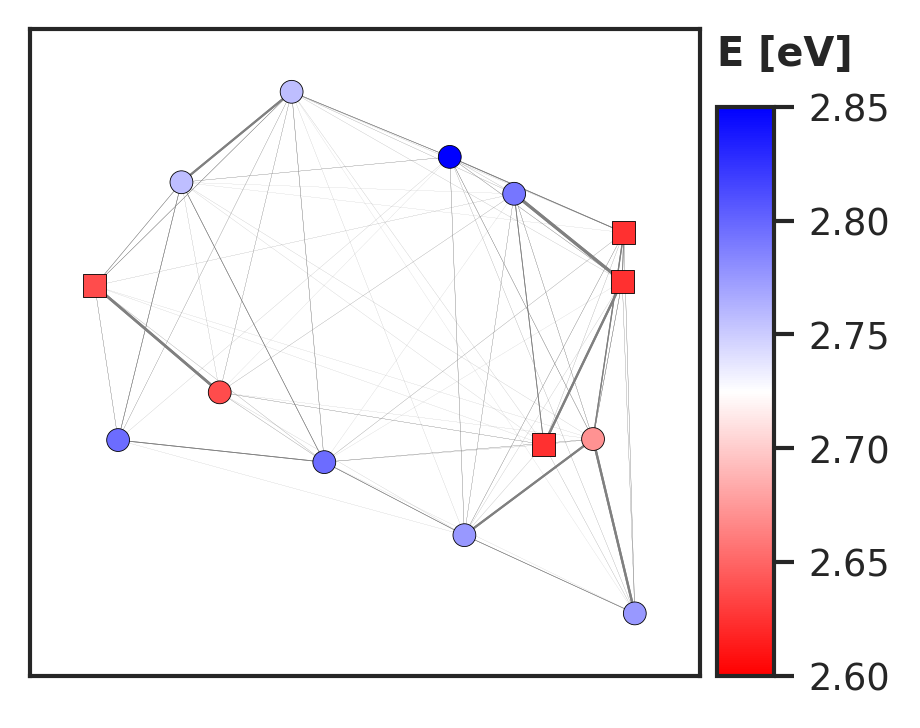

Supplement: Supplementary file 2 [file jp5c02465_si_002.zip › Fig4Analogues/CP29/CP29_noCrts_B.png]

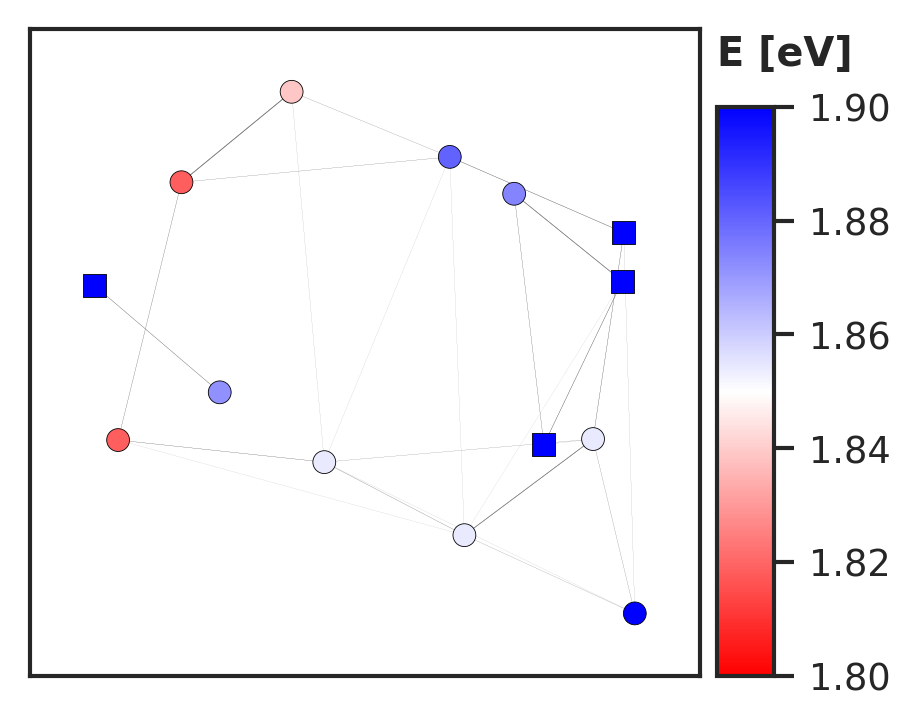

Supplement: Supplementary file 2 [file jp5c02465_si_002.zip › Fig4Analogues/CP29/CP29_noCrts_Q.png]

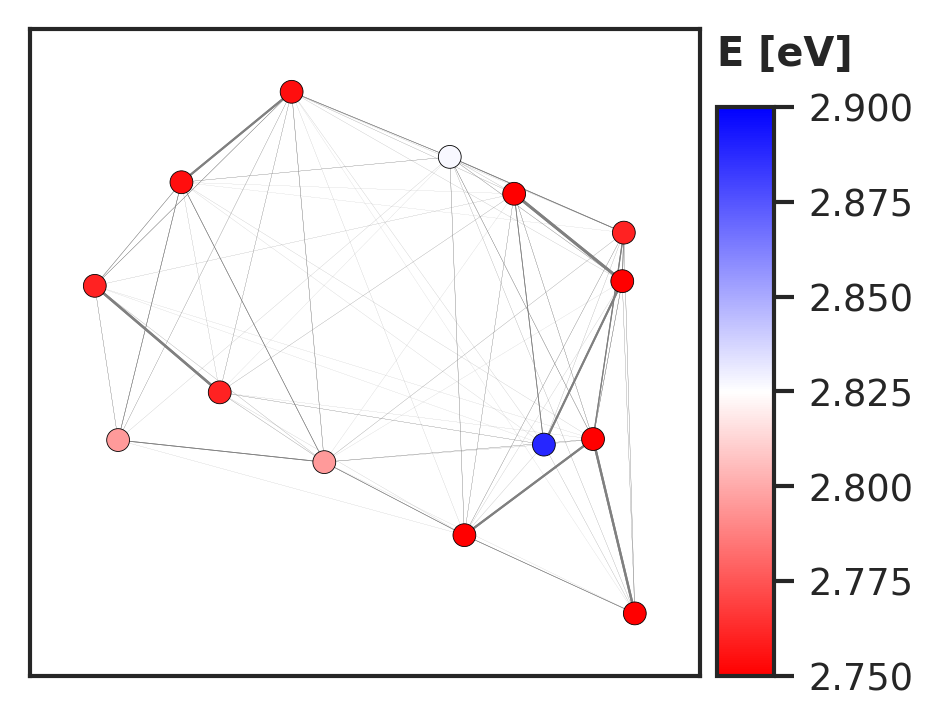

Supplement: Supplementary file 2 [file jp5c02465_si_002.zip › Fig4Analogues/CP29/CP29_onlyChla_B.png]

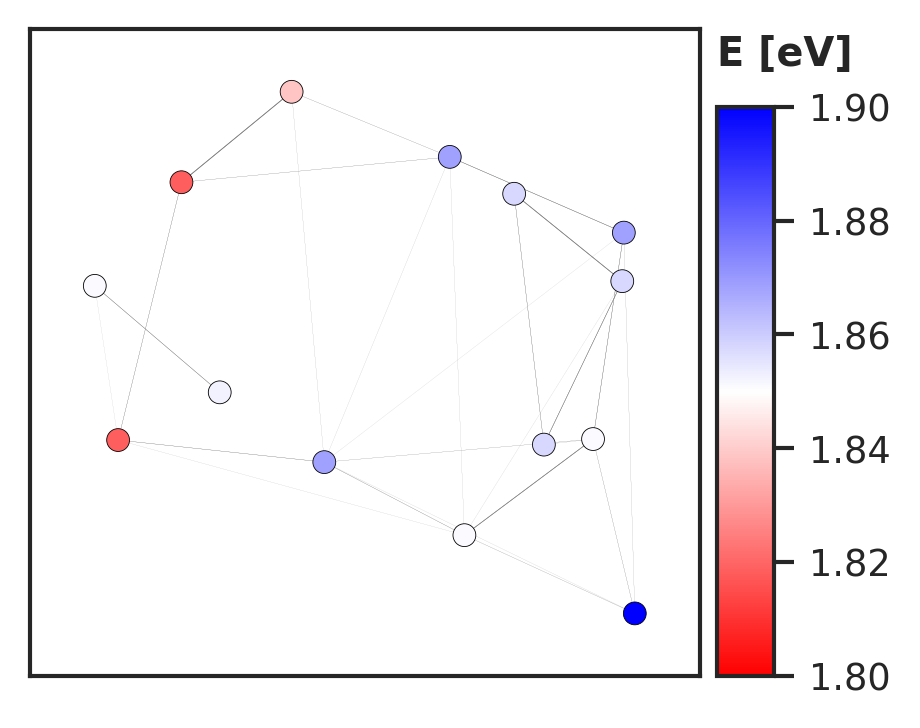

Supplement: Supplementary file 2 [file jp5c02465_si_002.zip › Fig4Analogues/CP29/CP29_onlyChla_Q.png]

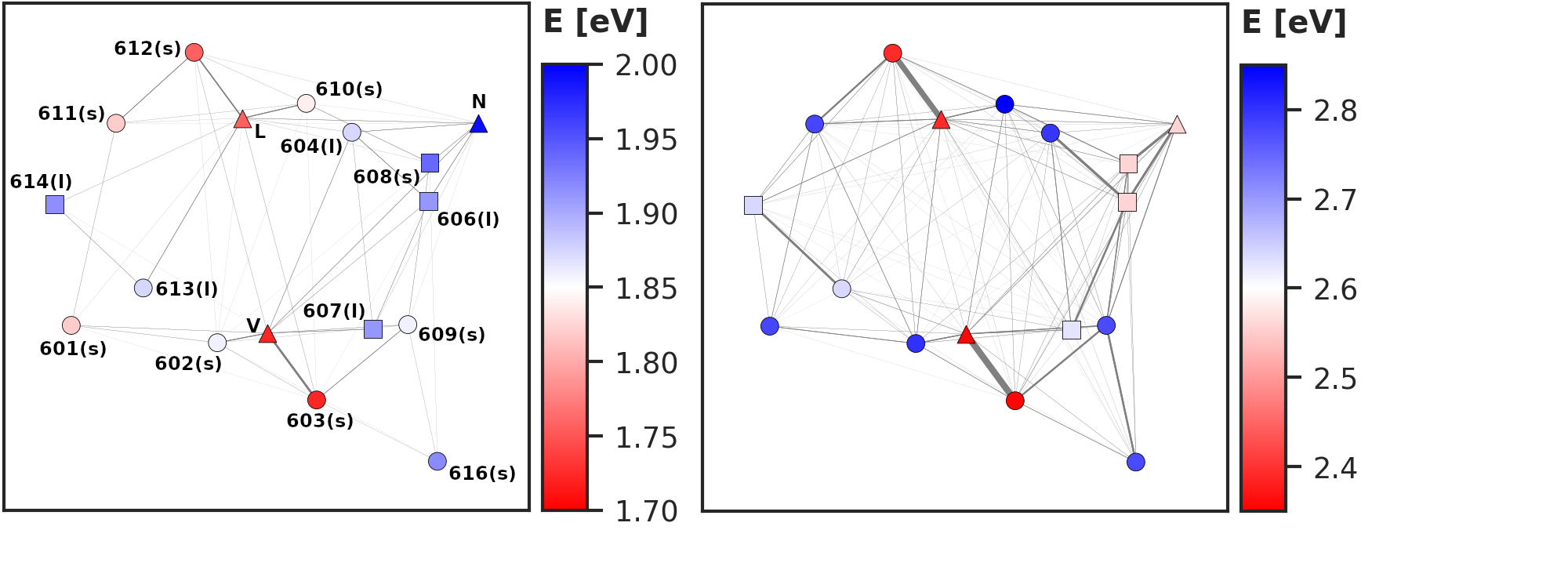

Supplement: Supplementary file 2 [file jp5c02465_si_002.zip › Fig4Analogues/CP29/CP29_WT_QandB.png]

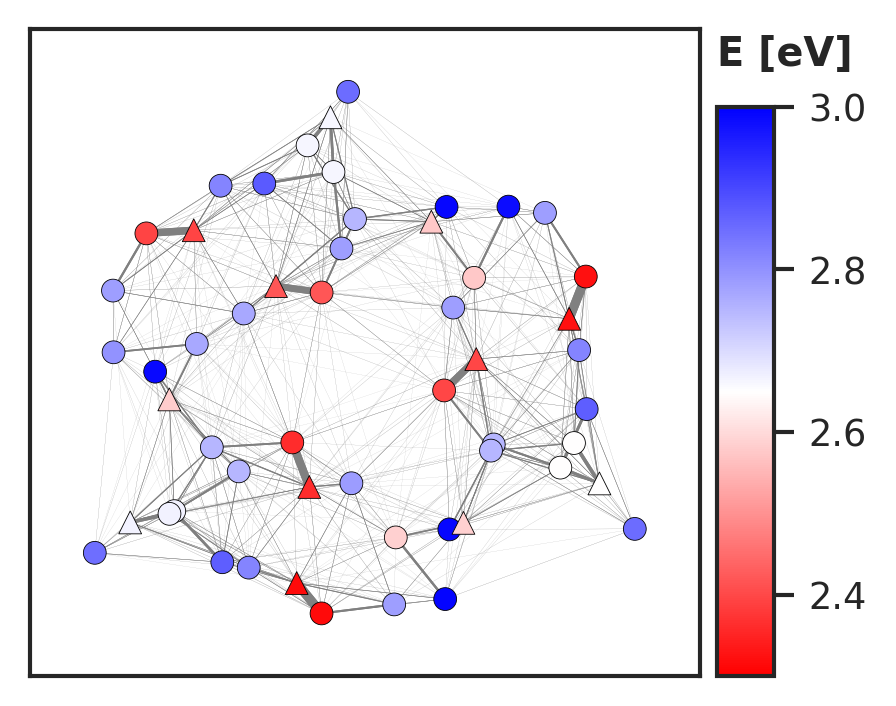

Supplement: Supplementary file 2 [file jp5c02465_si_002.zip › Fig4Analogues/LHCII/LHCII_ChlbreplacedbyChla_B.png]

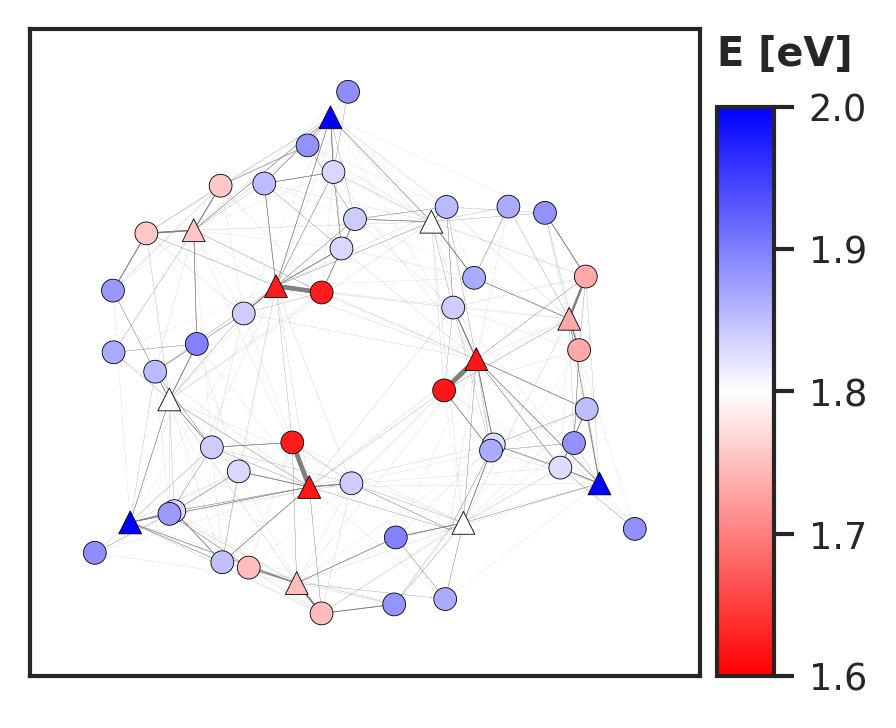

Supplement: Supplementary file 2 [file jp5c02465_si_002.zip › Fig4Analogues/LHCII/LHCII_ChlbreplacedbyChla_Q.png]

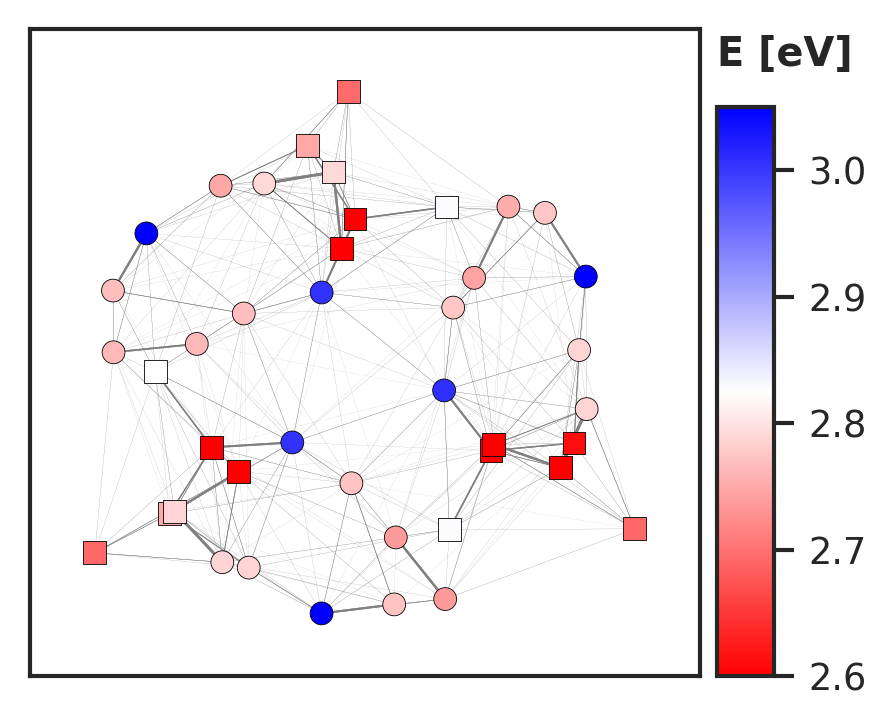

Supplement: Supplementary file 2 [file jp5c02465_si_002.zip › Fig4Analogues/LHCII/LHCII_noCrts_B.png]

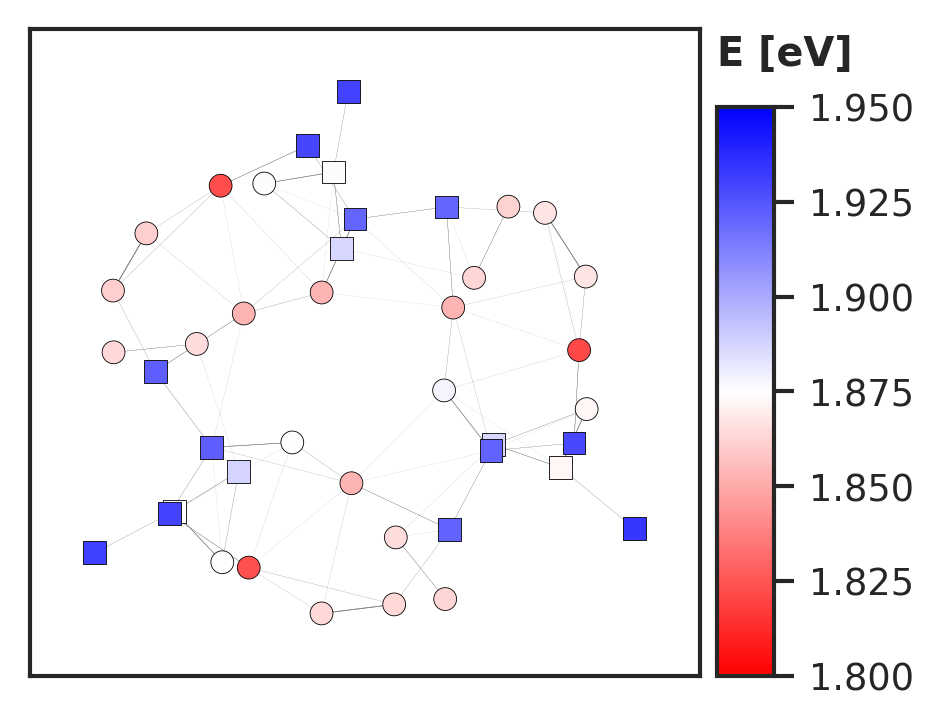

Supplement: Supplementary file 2 [file jp5c02465_si_002.zip › Fig4Analogues/LHCII/LHCII_noCrts_Q.png]

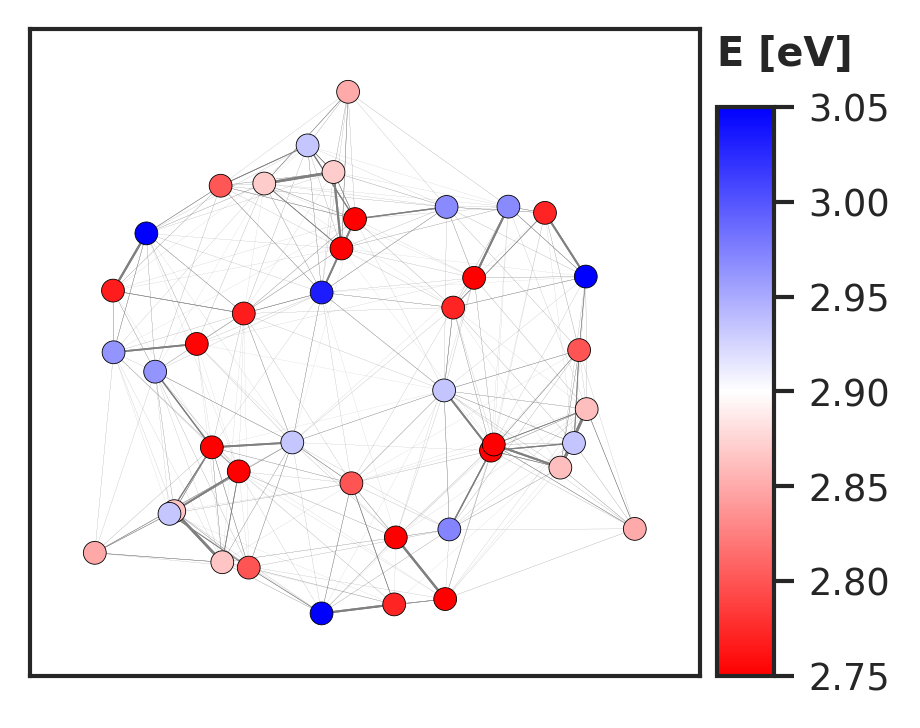

Supplement: Supplementary file 2 [file jp5c02465_si_002.zip › Fig4Analogues/LHCII/LHCII_onlyChla_B.png]

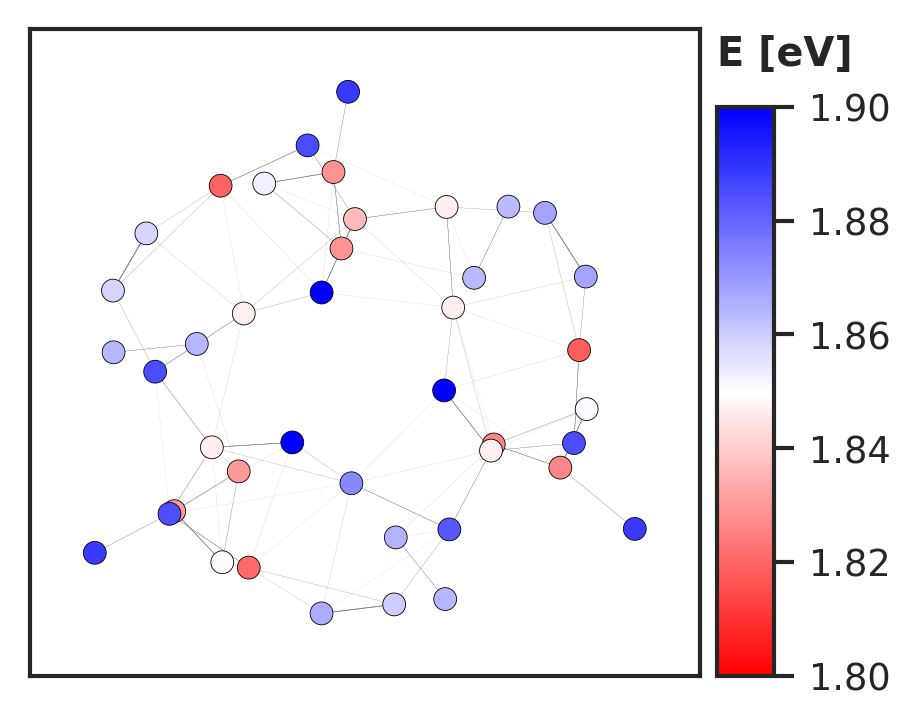

Supplement: Supplementary file 2 [file jp5c02465_si_002.zip › Fig4Analogues/LHCII/LHCII_onlyChla_Q.png]

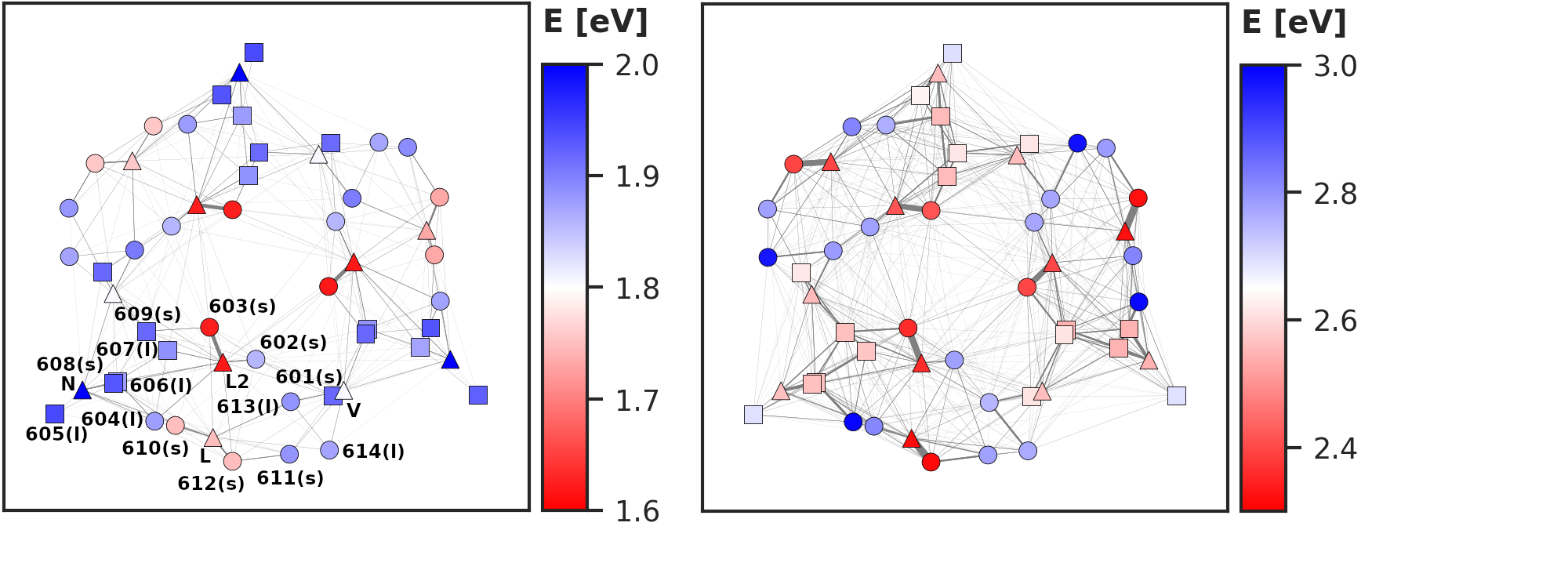

Supplement: Supplementary file 2 [file jp5c02465_si_002.zip › Fig4Analogues/LHCII/LHCII_WT_QandB.png]

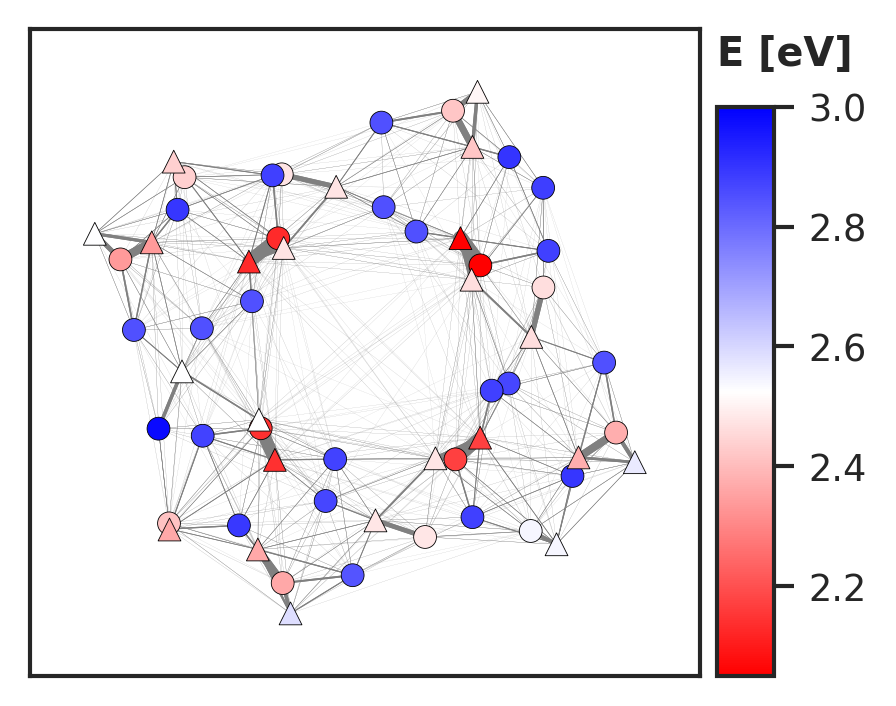

Supplement: Supplementary file 2 [file jp5c02465_si_002.zip › Fig4Analogues/tFCP/tFCP_ChlbreplacedbyChla_B.png]

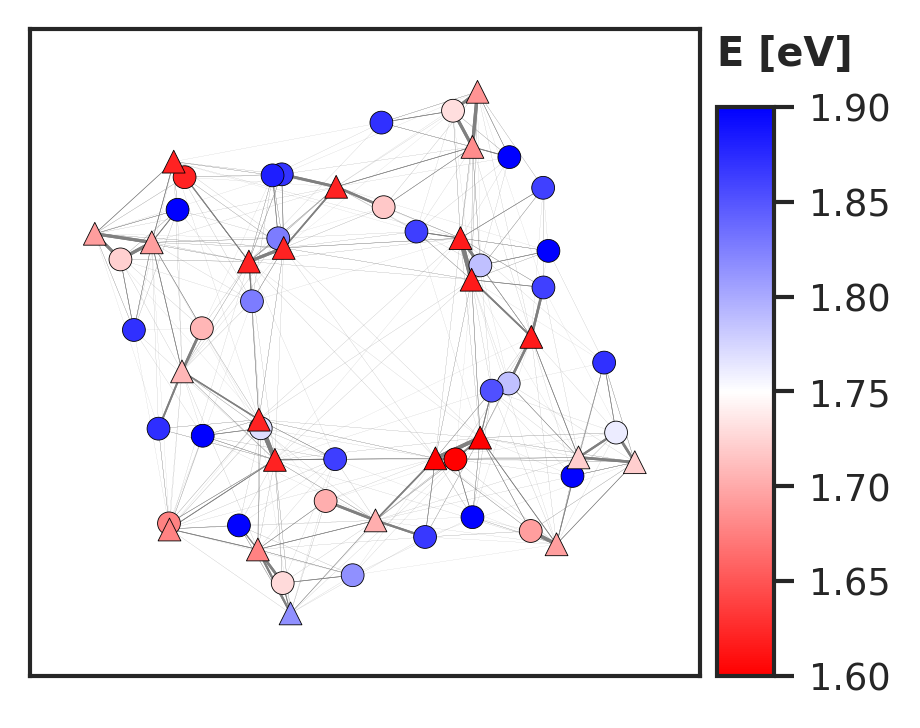

Supplement: Supplementary file 2 [file jp5c02465_si_002.zip › Fig4Analogues/tFCP/tFCP_ChlbreplacedbyChla_Q.png]

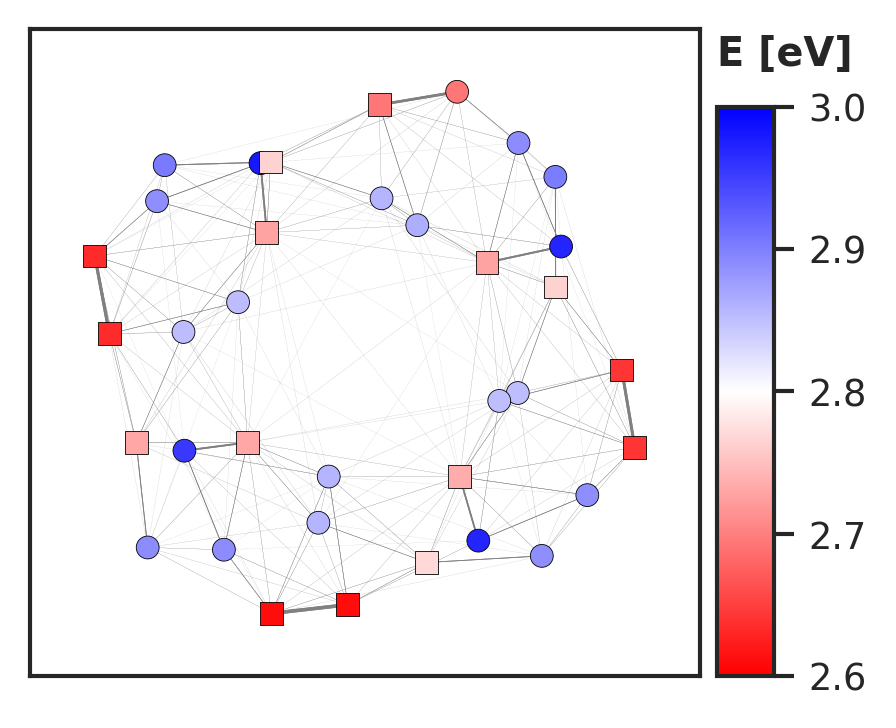

Supplement: Supplementary file 2 [file jp5c02465_si_002.zip › Fig4Analogues/tFCP/tFCP_noCrts_B.png]

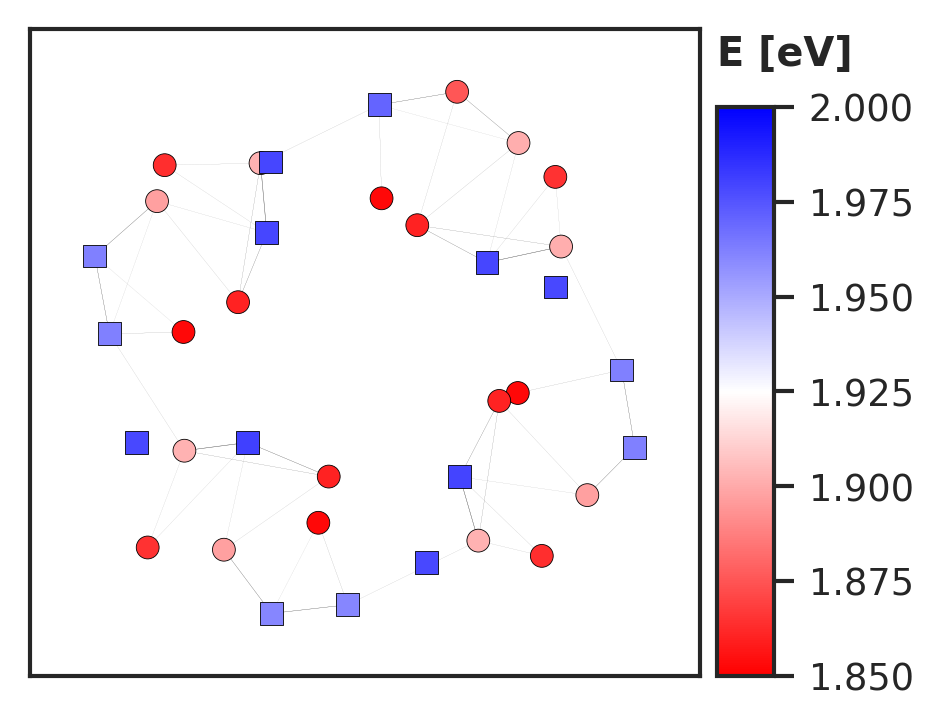

Supplement: Supplementary file 2 [file jp5c02465_si_002.zip › Fig4Analogues/tFCP/tFCP_noCrts_Q.png]
